# Taming *m*‐Quionodimethanes by Dispersion Force: Cyclodimerization of (Trialkylsilyl)Ethynyl‐Substituted Indeno[2,1‐*b*]Fluorenes and Fluoreno[2,3‐*b*]Fluorenes

**DOI:** 10.1002/chem.202502053

**Published:** 2025-08-19

**Authors:** Ching‐En Hung, Hsin‐Chung Lin, Rumi Ozawa, Yogajivan Rout, Hirokazu Miyoshi, Yutaka Ie, Ichiro Hisaki, Yen‐Ju Cheng, Yoshito Tobe

**Affiliations:** ^1^ Department of Applied Chemistry National Yang Ming Chiao Tung University 1001 Ta Hsueh Road Hsinchu 30030 Taiwan; ^2^ Nanoscience and Nanotechnology Center SANKEN The University of Osaka Ibaraki Osaka 567‐0047 Japan; ^3^ Division of Frontier Materials Science Graduate School of Engineering Science The University of Osaka Toyonaka Osaka 560–8531 Japan; ^4^ Division of Chemistry Graduate School of Engineering Science The University of Osaka Toyonaka Osaka 560–8531 Japan

**Keywords:** cyclodimerization, diradical intermediates, dispersion force, open‐shell character, quinodimethanes

## Abstract

We present herein a systematic study on the cyclodimerization of (trialkylsilyl)ethynyl‐substituted derivatives of indeno[2.1‐*b*]fluorene and fluoreno[2,3‐*b*]fluorene, which incorporate an open‐shell *meta*‐quinodimethane (*m*‐QDM) unit. Our investigation focuses on the influence of different alkyl groups (methyl, isopropyl, or cyclohexyl) attached to the silyl moiety. Upon generation from the precursor diols via treatment with SnCl_2_, these *m*‐QDM derivatives yielded cyclodimers with an *anti*‐configuration. The reaction yields varied significantly, ranging from virtually zero to quantitative, depending on the steric bulk of the alkyl group. Furthermore, the regioselectivity of bond formation (*Head* versus *Tail*) with respect to the ethynyl–allenyl radical resonance forms was also notably affected by the choice of alkyl group. These remarkable observations are attributed to attractive London dispersion forces operating between the π‐system and the alkyl groups. Crucially, the degree of size matching between these components dictates both the dimerization efficiency and the regioselectivity of the bond‐forming process.

## Graphical abstract

Indeno[2.1‐*b*]fluorenes and fluoreno[2,3‐*b*]fluorenes having (trialkylsilyl)ethynyl groups dimerize spontaneously to yield the cyclodimers in the yields ranging from virtually zero to quantitative depending on the alkyl group. The regioselectivity of the bond formation (*Head* versus *Tail*) is also dependent on the alkyl group. These remarkable effects are attributed to the attractive London dispersion force between the π‐ system and the alkyl groups.

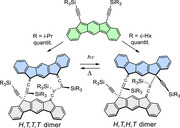

## 1 Introduction

**Figure 1:**
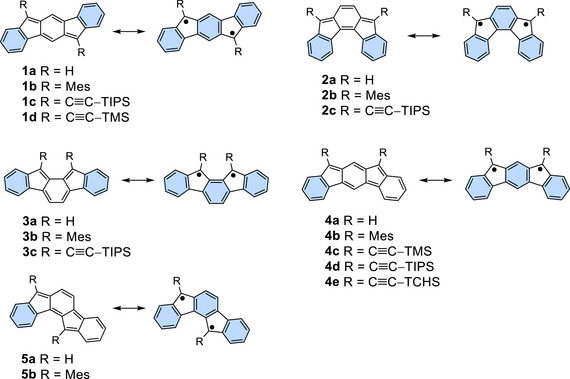
Resonance structures of indenofluorene isomers **1a**–**5a** and their derivatives. The turquoise rings highlight aromatic sextets.

Indenofluorenes are pentacyclic hydrocarbons composed of an s‐indacene or as‐indacene core, with benzene rings fused at different lateral edges of the core. They include five constitutional isomers, **1a**–**5a**, shown in Figure 1 in their closed‐shell quinoidal forms and open‐shell diradical canonical forms.^[^ ^1^, ^2^, ^3^, ^4^, ^5^ ^]^ Their electronic structures are qualitatively explained by the balance between destabilization arising from free valence (radical centers) and stabilization from aromatic sextets (benzene rings). All open‐shell forms contain three benzene rings with aromatic sextets. In the closed‐shell forms, however, the number of aromatic sextets varies depending on the structure. Isomers **1a** and **2a** possess a *para*‐quinodimethane (*p*‐QDM) moiety and two benzene rings. Similarly, **3a** contains an *ortho*‐quinodimethane (*o*‐QDM) and two benzene rings. Thus, the difference in the number of aromatic sextets between the closed‐shell and open‐shell forms is one, which explains the experimentally observed closed‐shell singlet ground states with small open‐shell character for derivatives of **1a**–**3a**.^[^ ^6^, ^7^, ^8^, ^9^ ^]^ In contrast, since the closed‐shell forms of **4a** and **5a** consist of a *meta*‐quinodimethane (*m*‐QDM), one benzene ring, and a cyclohexadienylidene unit, the difference in the number of aromatic sextets is two. This accounts for the larger open‐shell contribution in the electronic configurations of derivatives of **4a** and **5a**, which were shown to adopt open‐shell singlet and triplet ground state configurations, respectively.^[^ ^10^, ^11^ ^]^ Because of their small energy gaps between the highest occupied molecular orbitals (HOMOs) and lowest unoccupied molecular orbitals (LUMOs), as well as their open‐shell character, indenofluorenes, their π‐extended congeners, and heterocyclic analogs^[^ ^12^, ^13^, ^14^ ^]^ have attracted significant interest for applications in molecular electronics and spintronics.^[^ ^7^, ^15^, ^16^, ^17^, ^18^, ^19^, ^20^ ^]^

However, none of the unsubstituted derivatives of **1a**–**5a** have been isolated due to their high reactivity, which arises from their high HOMO levels and/or open‐shell character. The introduction of sterically bulky substituents, such as the 2,4,6‐trimethylphenyl (mesityl, Mes) group, at the most reactive apical positions of the five‐membered rings has been successful in kinetically stabilizing **1b**–**4b** for isolation,^[^ ^7^, ^8^, ^9^, ^10^ ^]^ except for the ground‐state triplet **5b**, which was not fully characterized in the neutral state.^[^ ^11^ ^]^ For **1a** and **2a**, (trialkylsilyl)ethynyl derivatives **1c** and **2c**, with bulky triisopropylsilyl (TIPS) groups, were also prepared not only to modulate electronic properties but also to engineer crystal packing geometries, which play critical roles in charge mobility when these compounds are used as organic field‐effect transistors (OFETs).^[^ ^6^, ^8^, ^15^ ^]^ By contrast, attempts to prepare **3c** were unsuccessful due to sequential intramolecular electrocyclic reactions caused by the proximity of the acetylene terminal carbons.^[^ ^21^ ^]^ These previous results prompted us to examine the preparation of (trialkylsilyl)ethynyl derivatives of **4a**, the last acetylene derivative remaining among the singlet indenofluorenes.

**Scheme 1:**
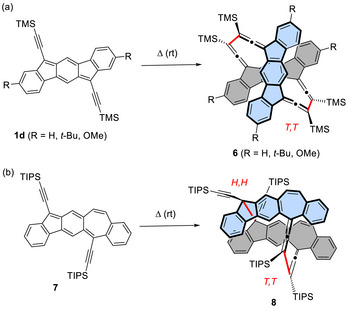
Cyclodimerizations of a) TMS‐substituted indenofluorene **1d** and b) TIPS‐substituted homolog **7**. In the dimers **6** and **8**, the original monomer units are drawn in grey and turquoise and the newly formed bonds are shown in red.

A few previous reports indicate that the stability of (trialkylsilyl)ethynyl‐substituted indenofluorenes depends on the steric bulk of the alkyl group. In contrast to **1c**, the corresponding trimethylsilyl (TMS) derivative **1d** was not isolated because of facile cyclodimerization, which produced dimers **6** in 7% to 20% yield (Scheme 1a), where C─C bonds were formed between the propargyl carbons (hereafter referred to as *Tail*–*Tail*).^[^ ^22^ ^]^ Moreover, in contrast to the stability of **1c**, although the homologous molecule **7** with a TIPS group was isolated once,^[^ ^23^, ^24^ ^]^ it gradually dimerized when left in solution for several days, giving cyclodimer **8** in which C─C bonds were formed between the acetylenic positions (hereafter referred to as *Head*–*Head*) as well as *Tail*–*Tai*l positions, in 25%–30% yield, along with a dioxygen‐incorporated product (10%–20%) (Scheme 1b).^[^ ^24^ ^]^ Note that in both examples, the yields of the dimers are low due to competing oligomer/polymer formation through random radical mechanisms.

**Figure 2:**
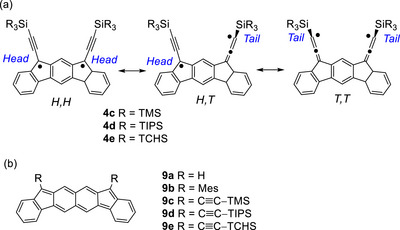
a) Open‐shell resonance structures of indeno[2.1‐*b*]fluorenes **4c**–**4e** showing the reaction centers (*Head* and *Tail*) and b) chemical structures of fluoreno[2,3‐*b*]fluorenes **9a**–**9e**.

We previously reported that indeno[2,1‐*b*]fluorene (**4a**)^[^ ^10^ ^]^ and its homolog fluoreno[2,3‐*b*]fluorene (**9a**)^[^ ^25^ ^]^ adopted open‐shell singlet configurations, with the latter having a larger open‐shell character, and characterized their mesityl derivatives **4b** and **9b** by spectroscopic and crystallographic methods (Figure 2). Herein, we disclose the results of a systematic study on the cyclodimerization of (trialkylsilyl)ethynyl derivatives **4c**–**4e** and **9c**–**9e**, in which the silyl groups are **c**: TMS, **d**: TIPS, **e**: tricyclohexylsilyl (TCHS), focusing on the effect of alkyl substituents on reactivity and stereoselectivity. Although none of **4c**–**4e** and **9c**–**9e** were detected when generated from their corresponding dihydrodiols by treatment with SnCl_2_, their cyclodimers with an *anti*‐configuration were obtained in yields ranging from virtually zero to quantitative, depending on the size of the alkyl groups. The positions of bond formation (*Head* versus *Tail* reactive centers, Figure 2b) were also influenced critically in some cases by the alkyl group. These remarkable effects are attributed to attractive London dispersion forces between the π‐systems and the alkyl groups. Cyclodimers were formed in high yields when the sizes of the interacting groups were well‐matched. These results suggest that, with proper molecular design, dispersion forces can be utilized to control the reactivity of diradicals under kinetically controlled conditions for the construction of macrocyclic structures.

## 2 Results and Discussion

### 2.1 Theoretical Structures and Electronic Properties of (Trialkylsilyl)Ethynyl‐Substituted Indeno[2,1‐*b*]Fluorenes and Fluoreno[2,3‐*b*]Fluorenes

**Figure 3:**
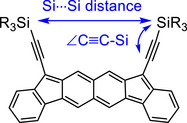
Structural parameters in theoretically optimized structures of **9c**–**9e**.

First, to assess the effect of the (trialkylsilyl)ethynyl groups on the structures and electronic properties, DFT calculations were performed for **4c**–**4e** and **9c**–**9e** using the B3LYP‐D3(BJ)/6–311G(d,p) level of theory. This level of theory has been reported to moderately reproduce dispersion forces.^[^ ^26^ ^]^ The relative energies of the open‐shell singlet state (with open‐shell character *y*_0_) and the triplet state, with respect to the closed‐shell singlet state, along with the structural parameters defined in Figure 3 for **9c**–**9e** (i.e., the angles at the sp carbon atoms of the triple bonds and Si⋯Si distances), are listed in Table 1.

**Table 1:** Theoretical electronic and geometrical properties of **4c**–**4e** and **9c**–**9e**.^[^ ^a^ ^]^

	Geometrical parameters		Relative energy with respect to that of closed‐shell singlet state	
Compound	∠C≡C─Si angle [av],^[^ ^c^ ^]^ deg	Si⋯Si Distance, Å	Open‐shell singlet, kcal/mol, (*y*_0_)^[^ ^b^ ^]^	Open‐shell triplet, kcal/mol
4c	177.9	7.29	−3.34 (0.16)	−2.29
4d	175.9	7.97	−3.36 (0.16)	−2.37
4e	175.9	7.83	−3.32 (0.16)	−2.28
9c	179.7	10.18	−4.79 (0.26)	−3.77
9d	173.1^[^ ^d^ ^]^	8.73	−4.69 (0.25)	−3.61
9e	167.4^[^ ^d^ ^]^	7.75	−4.67 (0.25)	−3.55

^[a]^ B3LYP‐D3(BJ)/6–311G(d,p) level of theory.

^[b]^ Open‐shell character *y*_0_ of the open‐shell singlet state, calculated using the Yamaguchi's scheme,^[^ ^27^ ^]^ is given in parentheses.

^[c]^ Average of the two angles.

^[d]^Distorted inward with respect to the π conjugated core.

As geometrical parameters, the nonbonded Si⋯Si distance and the bond angle of the terminal acetylene carbon (∠C≡C─Si) were evaluated. These parameters are expected to be affected by the intramolecular attractive or repulsive interactions between the alkyl groups in the different (trialkylsilyl)ethynyl substituents. It is worthwhile to note that the Si⋯Si distances of **4d** and **4e** are longer than that of **4c**, likely due to steric repulsion between the bulky alkyl groups confined within the limited space defined by the indenofluorene frame. Conversely, the Si⋯Si distances of **9d** and **9e** are shorter than that of **9c**, suggesting that the trialkylsilyl groups are drawn closer by attractive interactions between the alkyl groups. Correspondingly, the triple bonds are deformed from linearity, as indicated by C≡C─Si angles of 173° in **9d** and 167° in **9e**.

The relative energies of the open‐shell singlet and triplet states are compared with the closed‐shell singlet state. As reported previously for the parent **4a** and **9a**,^[^ ^10^, ^25^ ^]^ all **4c**–**4e** and **9c**–**9e** adopt open‐shell singlet ground state configurations, which are more stable than the closed‐shell states by ca. 3.3 kcal/mol for **4c**–**4e** and 4.6 kcal/mol for **9c**–**9e**. The triplet states are higher in energy than open‐shell singlet by approximately 1 kcal/mol. Despite these geometrical deformations, the relative stabilities of the open‐shell singlet states and the open‐shell indices (*y*_0_ values defined by the Yamaguchi's scheme),^[^ ^27^ ^]^ are virtually identical, indicating little effect of the deformation at the triple bond ends on the electronic properties. Moreover, the frontier molecular orbital levels of the open‐shell singlet state are also unaffected by the structural deformation (Figure S1a,b).

### 2.2 Cyclodimerization of Indenofluorene Derivatives

**Scheme 2:**
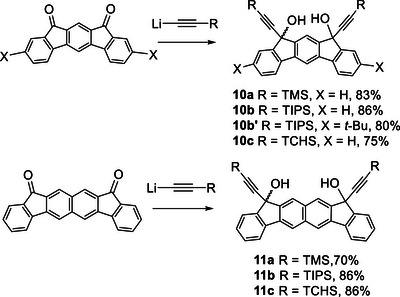
Preparation of precursor diols **10a**–**10d** and **11a**–**11c**.

The precursor diols **10a**–**10c** and **11a**–**11c** for **4c**–**4e** and **9c**–**9e**, respectively, were prepared by the addition of the corresponding acetylide to their respective diketones.^[^ ^10^, ^25^ ^]^ For **10b**, a di‐*tert*‐butyl derivative **10b’** was also synthesized to improve the solubility of the resulting cyclodimers (Scheme 2). Although the presence of two diastereomers was discernible in the ^1^H NMR spectra of **10a**, it appears likely that a single isomer was predominantly formed in the other cases.

The reductive dihydroxylation of the diols was conducted in most cases by treatment with excess anhydrous SnCl_2_ suspended in toluene at room temperature. The expected blue–greenish color development for the **4c**–**4e** and **9c**–**9e** was not observed, suggesting that the initial products reacted immediately upon formation. The ^1^H NMR spectra of the crude products, obtained after removal of the inorganic solid by passing the solution through a short alumina column, exhibited sharp signals corresponding to the cyclodimers dominantly in some cases, while broad signals due to intractable oligomers (or polymers) were observed mainly in other cases (Figure S4). For the cyclodimers, 14 possible diastereomers exist (Figure S2), seven each possessing *anti* or *syn* geometry with respect to the orientation of the aromatic frameworks. Within each *anti* and *syn* isomer group, seven diastereomers are conceivable from the different positions of C─C bond formation positions—*Head* and *Tail* (hereafter abbreviated as *H* and *T*)—as indicated in Figure 2a. Different sites of reactivity have been previously observed in the cyclodimers of the aforementioned **1c** and **7** (Scheme 1). In the present case, *H,H,H,H, H,H,H,T, H,H,T,T, H,T,H,T, H,T,T,H, T,T,T,H*, and *T,T,T,T* diastereomers are conceivable (Figure S2).

**Scheme 3:**
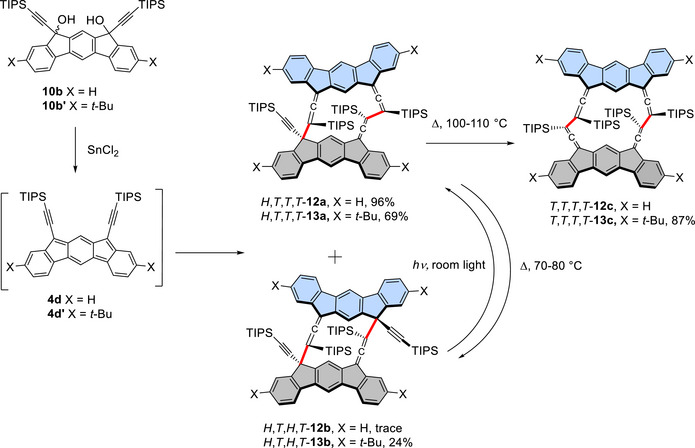
Cyclodimerization of **4d** (**4d’**) and subsequent isomerizations between cyclodimers *H,T,T,T*‐**12a** (**13a**), *H,T,T,T*‐**12b** (**13b**), and *T,T,T,T*‐**12c** (**13c**). The yields of preparative scale reactions are shown. In the cyclodimers, the monomer units are drawn in grey and turquoise and the bonds connecting the monomer units are shown in red.

**Figure 4:**
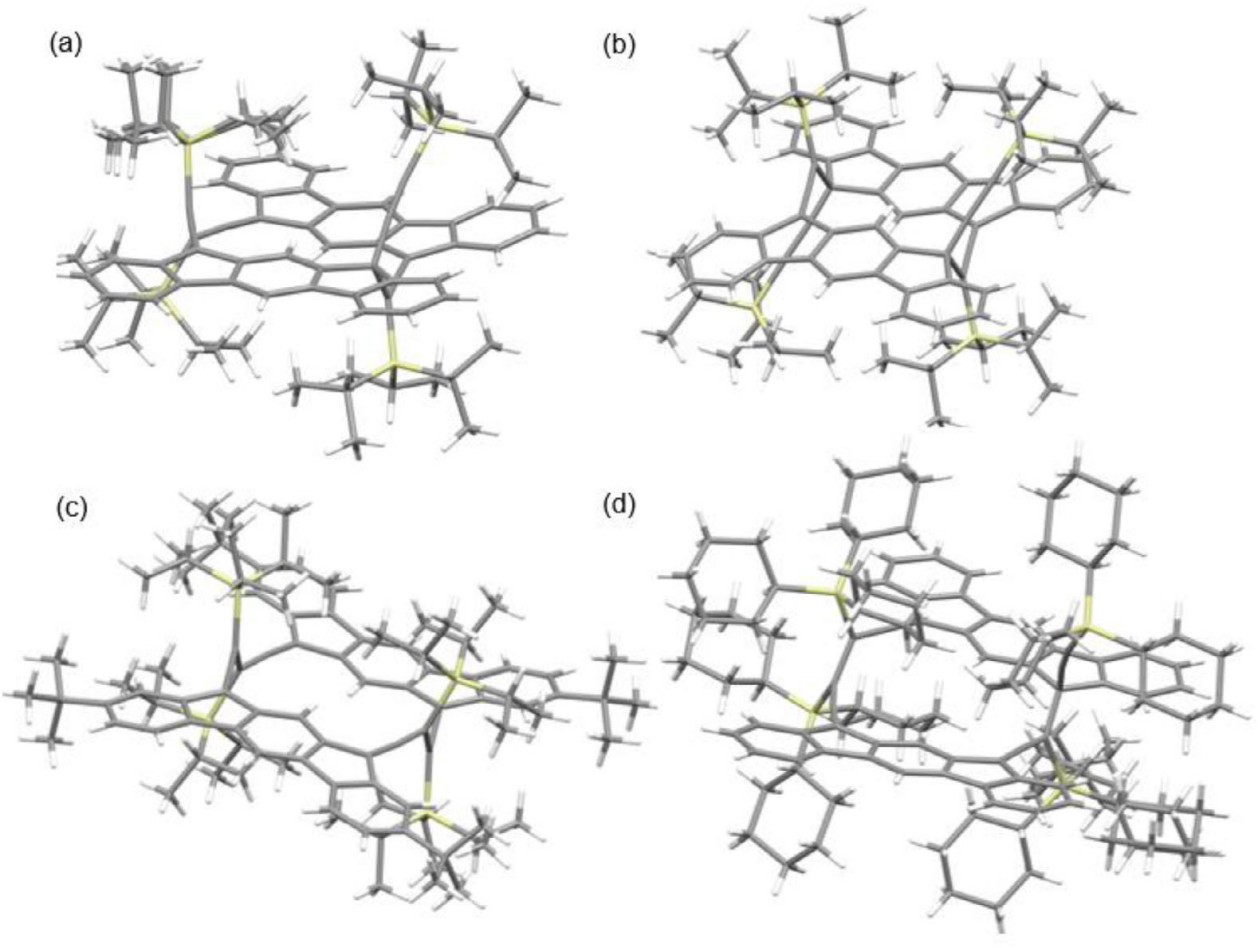
The crystal structures of a) *H,T,T,T*‐**12a**, b) *H,T,H,T*‐**12b**, c) *T,T,T,T*‐**13c**, and d) *H,T,H,T*‐**15b**.

We begin by describing the results of the preparative‐scale cyclodimerization of TIPS derivative **4d**, as this reaction not only yielded a single isomer of the cyclodimer quantitatively but also demonstrated that the initially obtained dimer could isomerize photochemically and thermally to different isomers, thereby showcasing its reactivity and spectroscopic characteristics. First, **4d** generated from the reaction of **10b** with SnCl_2_ afforded cyclodimer *H,T,T,T*‐**12a**, in 96% yield, which contained a small amount of the isomer *H,T,H,T*‐**12b** (Scheme 3). Salient features in the spectroscopic data of the dimer include eight doublet signals arising from the diastereotopic methyl protons in the four inequivalent isopropyl groups and four aromatic singlet protons in the ^1^H NMR spectrum; three signals due to the sp‐hybridized allene carbons, appearing at 205.0, 206.5, and 207.7 ppm in the ^13^C NMR spectrum; and an allenic stretching band at 1907 cm^−1^ in the IR spectrum. These data, together with the high‐resolution mass spectrum, indicate that the dimer possesses an unsymmetrical structure, which was confirmed by X‐ray crystallography (Figures 4a and S6). The crystal structure establishes the *anti*‐orientation between the aromatic frameworks and the connections at three allenic ends and one acetylenic quaternary carbon. Incidentally, we found that exposure of a CDCl_3_ solution of *H,T,T,T*‐**12a** to room light led to its quantitative transformation to *H,T,H,T*‐**12b**, as judged by ^1^H NMR spectroscopy, via a photo‐induced 1,3‐shift of one of the bonds from an allenyl position to a quaternary propargyl end. Owing to its higher symmetry, the resulting *H,T,H,T*‐**12b** exhibits four doublets for the isopropyl groups and two aromatic singlet peaks in the ^1^H NMR spectrum, and one allenic sp carbon signal in the ^13^C NMR spectrum. Its structure was ultimately confirmed by X‐ray crystallographic analysis (Figures 4b and S7). Conversely, heating a C_2_D_2_Cl_4_ solution of *H,T,H,T*‐**12b** at 70 °C resulted in its quantitative reversion to *H,T,T,T*‐**12a** via a thermal 1,3‐shift. Moreover, continued heating of *H,T,T,T*‐**12a** at a higher temperature (100 °C) in C_2_D_2_Cl_4_ formed a new isomer, presumed to be *T,T,T,T*‐**12c**. Although only the ^1^H NMR spectrum could be measured due to its very limited solubility, the structure was tentatively assigned based on the simple spectrum, which showed only two singlets and two doublets for the aromatic and methyl protons, respectively. These results suggest that *H,T,T,T*‐**12a** is initially formed as a kinetically preferred product. It isomerizes photochemically to the less stable *H,T,H,T*‐**12b**, which then thermally reverts to *H,T,T,T*‐**12a**. Ultimately, *H,T,T,T*‐**12a** transforms thermally to the most stable *T,T,T,T*‐**12c**. In contrast to the photochemical 1,3‐shift, which is orbital‐symmetry allowed, the facile occurrence of the thermally forbidden 1,3‐shift in *H,T,H,T*‐**12b** is ascribed to the rigid framework maintained by intramolecular nonbonding interactions involving the TIPS groups. It is worthwhile to note, however, that the most stable *T,T,T,T*‐**12c** was not formed directly from the dimerization of **4d**, indicating that the transition state leading to *T,T,T,T*‐**12c** from *H,T,T,T*‐**12a** differs from that of the dimerization process.

To confirm the structure of *T,T,T,T*‐**12c** by crystal structure analysis, the di‐*tert*‐butyl derivative *T,T,T,T*‐**13c**, which had been expected to exhibit better solubility, was prepared. Similar to the dimerization of **4d** described above, the SnCl_2_‐mediated reduction of **10b’** primarily yielded *H,T,T,T*‐**13a** through *tert*‐butyl‐bearing intermediate **4d’**, although an increased amount of *H,T,H,T*‐**13b** was also formed (Scheme 3). Photochemical isomerization to *H,T,H,T*‐**13b** and its subsequent thermal reversion to *H,T,T,T*‐**13a** were observed, consistent with the results of isomerizations of *H,T,T,T*‐**12a** and *H,T,H,T*‐**12b**. Heating *H,T,T,T*‐**13a** at 110 °C in toluene afforded the desired *T,T,T,T*‐**13c** (87%). Although it displayed the expected IR band at 1904 cm^−1^, its solubility was not as high as anticipated once the solid product was purified, and consequently, only a low‐intensity ^1^H NMR spectrum could be recorded. However, through slow evaporation of the solvent from a dilute toluene solution containing impure products, crystals suitable for X‐ray analysis were obtained. This analysis revealed the highly symmetric framework with *T,T,T,T* connectivity at all allenic ends (Figures 4c and S8).

**Scheme 4:**
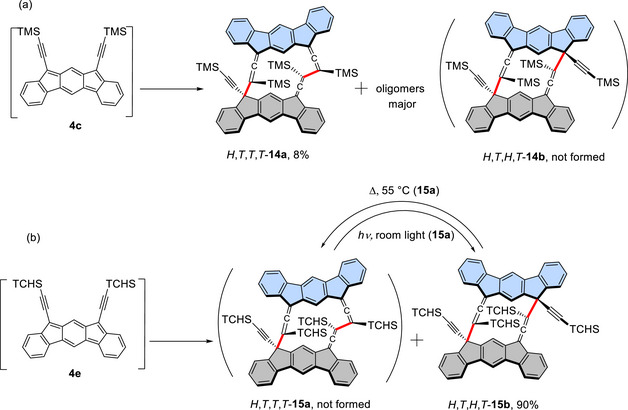
a) Cyclodimerization of **4c** giving oligomers mainly with a small amount of *H,T,T,T*‐**14a** and b) cyclodimerization of **4e** and subsequent isomerizations between cyclodimers *H,T,T,T*‐**15a** and *H,T,T,T*‐**15b**. The yields of preparative scale reactions are shown. In the cyclodimers, the monomer units are drawn in grey and turquoise and the bonds connecting the monomer units are shown in red.

On the other hand, the SnCl_2_‐mediated reduction of TMS‐diol **10a** primarily resulted in the formation of oligomers (Scheme 4a), as indicated by the ^1^H NMR spectrum of the crude product (Figure S4). The LD‐TOF MS analysis of the oligomer fraction, separated by GPC, exhibited fragment peaks with *m/z* values larger than that of the dimer, suggesting that this fraction consists mainly of small oligomers (Figure S5‐1). While the ^1^H NMR spectrum displayed only broad peaks, the IR spectrum showed both allenic and acetylenic stretching bands, indicating that the monomer units are linked at both types of reactive centers. A small amount (ca. 10%) of cyclodimer *H,T,T,T*‐**14a** was also obtained. Its structure was deduced based on the presence of four methyl signals from the TMS groups and the similarity of its aromatic signals to those of *H,T,T,T*‐**12a** in the ^1^H NMR spectrum.

Notably, in the case of TCHS‐substituted **4e**, *H,T,H,T*‐**15b** was obtained as the sole cyclodimer in 90% yield (Scheme 4b). This contrasts with the cyclodimerization of **4e**, which predominantly yielded *H,T,T,T*‐**12a**. The structure of *H,T,H,T*‐**15b** was confirmed based on its similarity to that of *H,T,H,T*‐**12b**, including the presence of two aromatic singlets in the ^1^H NMR spectrum, one allenic carbon signal at 205.1 ppm in the ^13^C NMR spectrum, and an IR stretching band at 1908 cm^−1^. Moreover, heating a toluene solution of *H,T,H,T*‐**15b** at 50 °C resulted in the slow formation of the *H,T,T,T*‐**15a** (88%), accompanied by apparent decomposition. The structure of *H,T,T,T*‐**15a** was elucidated based on spectral properties, including three allenic sp carbon signals at 207.3, 206.1, and 205.0 ppm in the ^13^C NMR spectrum, that share characteristics with *H,T,T*,*T*‐**12a**, and it was further confirmed by X‐ray crystallography (Figures 4d and S9). Photoisomerization of *H,T,T,T*‐**15a** to *H,T,H,T*‐**15b** also took place by exposure to room light at room temperature accompanied by considerable decomposition. To summarize, contrary to **4c**, which mainly polymerizes, **4d** and **4e**, equipped with bulky trialkylsilyl groups, dimerize efficiently, producing dimers but with different selectivity in the sites of bond formation.

### 2.3 Cyclodimerization of Fluorenofluorene Derivatives

**Scheme 5:**
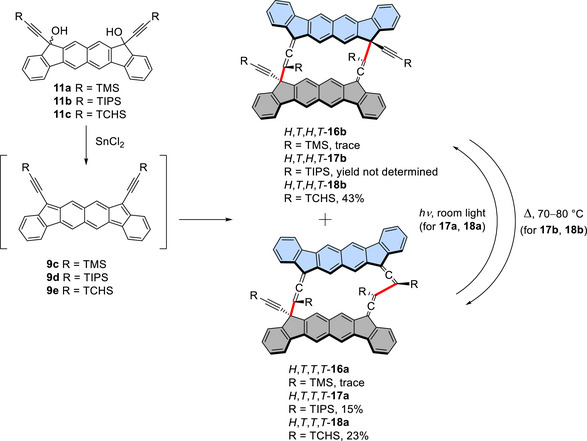
Cyclodimerization of **9d** derivatives and subsequent isomerizations between cyclodimers of *H,T,T,T*‐**17a** (**18a**) and *H,T,H,T*‐**17b** (**18b**). The yields of preparative scale reactions are shown. In the cyclodimers, the monomer units are drawn in grey and turquoise and the bonds connecting the monomer units are shown in red.

Similar alkyl group dependence on cyclodimerization was observed for fluorenofluorene derivatives **9c**–**9e**, despite with lower efficiency compared to the case of indenofluorenes. The reaction of **9c** predominantly yielded oligomers as seen in Figure S4 (Scheme 5). Based on LD‐TOF MS analysis (Figure S5–2) and the IR spectrum, the oligomers were deduced to consist of a few monomer units connected at both acetylenic and allenic ends. A very small amount (1% yield) of the cyclodimer *H,T,H,T*‐**16b** was isolated. Although only the ^1^H NMR spectrum (showing four aromatic and two TMS methyl singlet signals) and mass spectrum could be recorded, these data supported the proposed structure. Other characterization data were not acquired due to the limited quantity of material. Thus, the cyclodimerization was even less efficient in this case compared to that of **4c**.

Cyclodimerization of **9d** was also not as efficient as that of **4d**, as indicated by the ^1^H NMR spectrum of the crude product (Figure S4). The exact yields were not determined because purification of the products was hampered by difficulties in removing oligomers and separating the isomers, as well as by facile photochemical and thermal isomerizations between the *H,T,T,T*, and *H,T,H,T* isomers. However, through fractionation by dissolution in ethyl acetate, a mixture of dimers was obtained in approximately 20% yield, which mainly contained *H,T,T,T*‐**17a** (isolated in 15% yield) along with *H,T,H,T*‐**17b** as a minor component (Scheme 5). To obtain the latter isomer, a CDCl_3_ solution containing the mixture of isomers was exposed to room light for a few hours, resulting in a quantitative conversion to *H,T,T,T*‐**17a**. Conversely, when a toluene‐*d*₈ solution of the *H,T,H,T* isomer was heated at 70 °C for 4 hours in the dark, quantitative reversion to *H,T,H,T*‐**17b** was observed. Pure samples of the isomers were thus obtained by utilizing these photochemical and thermal isomerizations. These dimers exhibit NMR and IR signals consistent with their symmetry. Salient signals of *H,T,T,T*‐**17a** include eight aromatic singlets (two overlapping) and eight methyl doublets in the ^1^H NMR spectrum, three allenic carbon signals in the ^13^C NMR spectrum, and an IR stretching band at 1903 cm^−1^. The corresponding signals of *H,T,H,T*‐**17b** include four aromatic singlets and four methyl doublets in the ^1^H NMR spectrum, one allenic carbon signal in the ^13^C NMR spectrum, and a stretching band at 1909 cm^−1^ in the IR spectrum. Their structures were ultimately confirmed by X‐ray crystallographic analyses (Figure S10 and S11). The lower selectivity observed in the cyclodimerizations of **9c** and **9d**, compared with the corresponding indenofluorene derivatives, is attributed to the larger open‐shell character of the fluorenofluorene system and the greater distance between the reaction sites, which may be entropically disadvantageous for ring closure to form the cyclodimers.

The reaction of **9e**, which possesses the bulkiest TCHS groups, produced cyclodimers more selectively (Figure S3b). Because the *H,T,T,T* and *H,T,H,T* isomers were formed in a comparable ratio (approximately 1:1.7), a chloroform solution of the mixture was exposed to room light to promote the photoisomerization of the former isomer to the latter (Scheme 5). The latter isomer, *H,T,H,T*‐**18b**, was obtained in 64% yield by collecting the solid that precipitated from the solution. Although the ^13^C NMR spectrum could not be recorded due to low solubility once the solid product was purified, the structure was assigned based on the observation of two aromatic singlet peaks in the ^1^H NMR spectrum and an IR band at 1912 cm^−1^. *H,T,T,T*‐**18a** was obtained quantitatively via thermal isomerization of *H,T,H,T*‐**18b** by heating it in toluene at 80 °C. As before, the structure was assigned based on the ^1^H NMR (eight aromatic singlets), ^13^C NMR (three allenic carbon signals), and IR (1895 cm^−1^ band) spectra. This assignment was also confirmed by X‐ray crystallography (Figure S12).

### 2.4 Summary of Cyclodimerization

**Figure 5:**
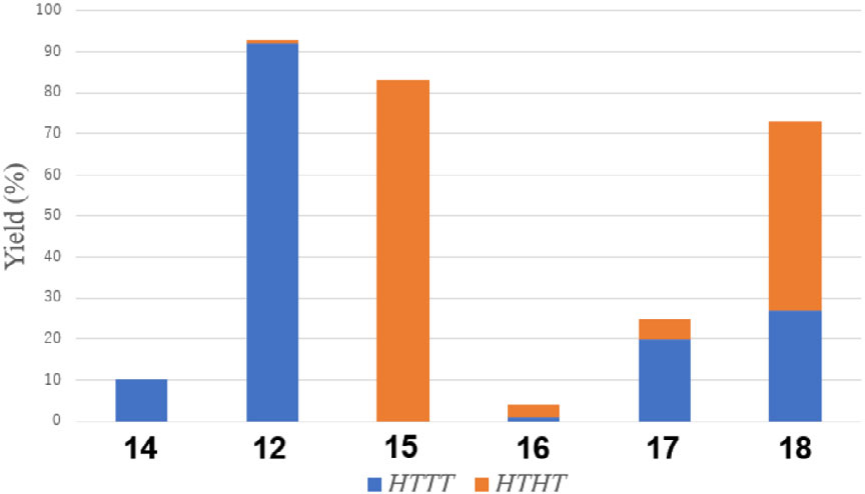
Yields of cyclodimers and their isomer distribution. Blue: *H,T,T,T* isomer, orange: *H,T,H,T* isomer.

Since the yields from preparative‐scale reactions are not consistently reliable due to difficulties in purifying the cyclodimers, the reactions were conducted on a small scale. This allowed for more accurate yield determination by ^1^H NMR spectroscopy using an internal standard, without the need for isolating the cyclodimers, as detailed in the Experimental Section. The results are summarized in Figure 5 and Table 2.

**Table 2:** Cyclodimerization of **4c**–**4d’** and **9c**–**9e**.^[^ ^a^ ^]^

		Dimer, Yield, %		Relative amount
Monomer	Alkyl group	*H,T,T,T*	*H,T,H,T*	Oligomers
**4c**	methyl	**14a**, 10	**14b**, ND^[^ ^b^ ^]^	main
**4d**	*i*‐propyl	**12a**, 92	**12b**, 1	trace
**4d’**	*i*‐propyl	**13a**, 82	**13a**,15	trace
**4e**	cyclohexyl	**15a**, ND^[^ ^b^ ^]^	**15b**, 83	trace
**9c**	methyl	**16a**, (1)^[^ ^c^ ^]^	**16b**, (3)^[^ ^c^ ^]^	main
**9d**	*i*‐propyl	**17a**, 20	**17b**, 5	main
**9e**	cyclohexyl	**18a**, 27	**18b**, 46	minor

^[a]^ The results determined using an internal standard (See Experimental Section for the detail).

^[b]^ Not detected.

^[c]^ Not accurate due to the small integration of the relevant signal in the ^1^H NMR spectrum.

Notably, **4d** (**4d’**), **4e**, and **9e** produced cyclodimers in excellent yields, in contrast to other cases where oligomers were predominantly formed. The effect of the TCHS group in promoting cyclodimerization is particularly remarkable in the more reactive fluorenofluorene system. Moreover, whereas **4d** selectively forms the *H,T,T,T*‐dimer, **4e** exclusively yielded the *H,T,H,T* isomer, and **9e** produced both isomers. From the perspective of higher‐order cycloadditions,^[^ ^28^, ^29^ ^]^ it is worth mentioning that the observed reactions can be classified as formal [14 + 16], [14 + 14], [16 + 18], and [16 + 16] cycloadditions. The resultant macrocyclic architectures possess [4.4]‐ and [4.3]cyclophane frameworks.

### 2.5 Structures and Stabilities of Cyclodimers

**Figure 6:**
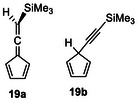
Chemical structures of model compounds **19a** and **19b**.

To assess the relative stabilities of the regioisomers, DFT calculations for the cyclodimers were carried out at the B3LYP‐D3(BJ)/6–311G(d,p) level of theory (Table 3). In all cases, the *H,T,T,T* isomers are considerably more stable than the corresponding *H,T,H,T* isomers by approximately 11–17 kcal/mol. This trend parallels the observed predominance of the *H,T,T,T* isomers, except for the TCHS‐substituted dimers which predominantly yielded the *H,T,H,T* isomers. The general stability trend is attributed to the π‐conjugation in the allenylidenecyclopentadiene substructure formed by the *Tail* bonding mode, compared to that in the ethynylcyclopentadiene substructure formed by the *Head* mode. The model compound **19a** is estimated to be more stable than **19b** by 8.0 kcal/mol, according to calculations at the B3LYP/6–311G(d,p) level of theory (Figure 6). In addition, the destabilization in the *H,T,H,T* isomers due to steric compression around the quaternary carbons increases the relative stability difference, as observed for the TMS‐bearing *H,T,T,T* isomers, which are 12–13 kcal/mol more stable than their corresponding *H,T,H,T* isomers. Moreover, the TCHS‐substituted dimers exhibit the largest energy difference between the *H,T,T,T* and *H,T,H,T* isomers, suggesting increased dispersion stabilization arising from the *T‐T* connection mode, likely due to enhanced dispersion interactions with the cyclohexyl groups.

**Table 3:** Stabilities of *H,T,T,T* and *H,T,H,T* Isomers of Cyclodimers.^[^ ^a^ ^]^

Cyclodimer	Relative energy of *H,T,H,T* isomer,^[^ ^b^ ^]^ kcal/mol
*H,T,T,T*‐**14a** / *H,T,H,T*‐**14b**	13.0
*H,T,T,T*‐**12a** / *H,T,H,T*‐**12b**	12.5
*H,T,T,T*‐**15a** / *H,T,H,T*‐**15b**	14.3
*H,T,T,T*‐**16a** / *H,T,H,T*‐**16b**	12.1
*H,T,T,T*‐**17a** / *H,T,H,T*‐**17b**	10.9
*H,T,T,T*‐**18a** / *H,T,H,T*‐**18b**	16.0

^[a]^ Calculated by the B3LYP‐D3(BJ)/6–311G(d,p) level.

^[b]^ The relative theoretical energy of the *H,T,H,T* isomer with respect to the *H,T,T,T* isomer.

**Figure 7:**
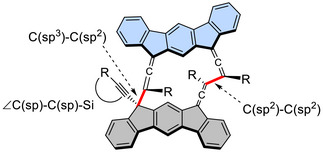
Chemical structures of a *H*,*T*,*T*,*T*‐dimer showing the structural parameters, C(sp^3^)─C(sp^2^), C(sp^2^)─C(sp^2^), and ∠C(sp)─C(sp)─Si, listed in Table 4.

The structures of the cyclodimers *H,T,T,T*‐**12a**, *H,T,H,T*‐**12b**, *T,T,T,T*‐**13c**, *H,T,T,T*‐**15a**, *H,T,T,T*‐**17a**, *H,T,H,T*‐**17b**, and *H,T,T,T*‐**18a** were determined by single‐crystal X‐ray diffraction.^[^ ^30^ ^]^ The molecular structures are depicted in Figure 4 for representative dimers and Figures S5–S11. The most notable common feature is the geometry of the trialkylsilyl groups, which hang over the π‐framework of the counterpart molecule within the dimer. As a result, numerous short intramolecular contacts are observed between the hydrogen atoms of the alkyl groups and the carbon atoms of the π‐framework, as summarized in Figures S13‐1–S13‐7. Additionally, due to steric compression, these dimers exhibit elongated newly formed C─C bond lengths, which are listed in Figure 7 and Table 4. This table also includes the angles of the triple bond at the external C(sp) atom.

**Table 4:** Long C─C Bond Lengths in the Crystal Structures of Cyclodimers *H,T,T,T*‐**12a**, *H,T,H,T*‐**12b**, *T,T,T,T*‐**13c**, *H,T,T,T*‐**15a**, *H,T,T,T*‐**17a**, *H,T,H,T*‐**17b**, and *H,T,T,T*‐**18a**.

Compound	C(sp^3^)─C(sp^2^), Å	C(sp^2^)─C(sp^2^), Å	∠C(sp)─C(sp)─Si, deg
*H,T,T,T*‐**12a**	1.572 (5)	1.525 (5)	174.2
*H,T,H,T*‐**12b**	1.586(2)	–	176.5
*T,T,T,T*‐**13c**‐1^[^ ^a^ ^]^	–	1.507(6)	
*T,T,T,T*‐**13c**‐2^[^ ^a^ ^]^	–	1.526(5)	
*H,T,T,T*‐**15a**	1.568 (4)	1.534 (3)	170.1
*H,T,T,T*‐**17a**‐1^[^ ^a^ ^]^	1.557 (20)	1.525 (10)	173.4
*H,T,T,T*‐**17a**‐2^[^ ^a^ ^]^	1.548 (10)	1.504 (10)	175.0
*H,T,H,T*‐**17b**	1.585(4)	–	171.2
*H,T,T,T*‐**18a**	1.564 (5)	1.509 (5)	177.5

^[a]^ There are two crystallographically independent molecules in a unit cell.

The C(sp^3^)─C(sp^2^) bond lengths are longer than the typical bond length (1.51 Å^[^ ^31^ ^]^ or 1.50 Å^[^ ^32^ ^]^) by 0.06–0.07 Å, due to steric crowding around the quaternary carbon. The C(sp^2^)─C(sp^2^) bond lengths are less elongated from typical lengths (1,48 Å,^[^ ^31^ ^]^ or 1.46 Å,^[^ ^32^ ^]^) by 0.03–0.05 Å, likely because the sp^2^ carbons are further from the core cyclodimer framework. The existence of intramolecular nonbonded C⋯H distances that are shorter by approximately 0.1 Å than the sum of their van der Waals radii (Figures S13‐1–S13‐7) is consistent with the intramolecular dispersion interactions discussed in the next section. Another notable feature is the deformation of the triple bond from linearity, presumably to gain stabilization through dispersion interactions.

### 2.6 Role of Dispersion Forces in Cyclodimerization

**Scheme 6:**
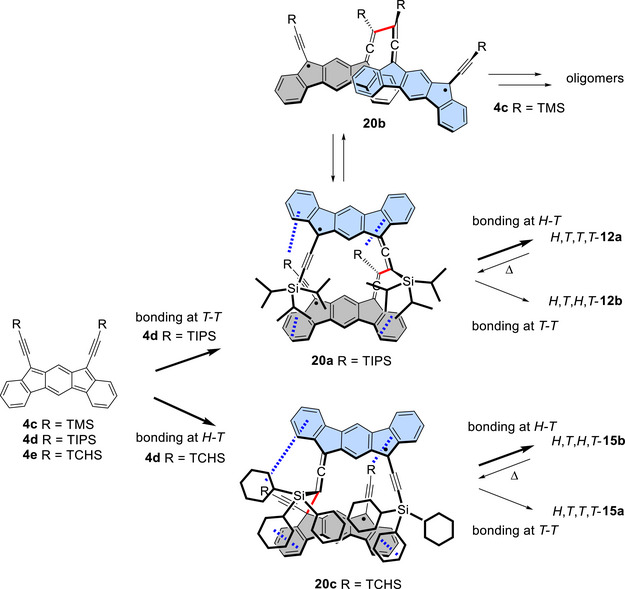
A plausible mechanism of cyclodimerization of **4c**–**4e** through diradical intermediates **20a**–**20c**. In the diradical intermediates, the monomer units are drawn in grey and turquoise and the initially formed bonds are shown in red. In **20a** and **20c**, the attractive noncovalent interactions due to dispersion forces are indicated by blue hashed lines.

Before discussing the effect of the alkyl groups on the observed cyclodimerization vs. oligomerization selectivity and the regioselectivity of dimerization, the reaction mechanism is considered. Given the large open‐shell characters of indenofluorene and fluorenofluorene,^[^ ^10^, ^25^ ^]^ and in view of the reported mechanisms for the cyclodimerizations of the closely related (trialkylsilyl)ethynyl‐substituted **1d** ^[^ ^22^ ^]^ and **7** ^[^ ^24^ ^]^ (Scheme 1), it is reasonable to assume that the present cyclodimerizations proceed via a stepwise mechanism. Recent mechanistic studies on the cyclodimerizations of *para*‐quinodimethane,^[^ ^33^ ^]^ an extended *ortho*‐quinodimethane termed sigmarene,^[^ ^34^ ^]^ and a cyclooctatetraene homologue of **1a** ^[^ ^35^ ^]^ also support this view. Therefore, assuming that the initial C─C bond is formed at the *T* and *T* positions (indicated by the red thick bond, Scheme 6), diradical intermediates such as closed form **20a** and open form **20b** are conceivable. **20a** and **20b** are conformers arising from rotation around the red bond, exhibiting negligible and significant overlap, respectively, between the trialkylsilyl groups and the π‐frameworks. Though sterically encumbered, the *T*‐*T* bond formation would be favored in view of the allenylcyclopentadiene moiety discussed above. Considering the small singlet‐triplet energy gap in the open‐shell states of indenofluorene and fluorenofluorene (Table 1), the diradical intermediates may adopt either singlet or triplet configurations. However, the spin multiplicity is unlikely to affect the position of the bond formation. We hypothesize that, in the case of R = *i*‐Pr, **20a** experiences stabilization due to dispersion force by the *i*‐Pr groups, undergoing the second bond formation at the less crowded *H*‐*T* positions, producing the *H*,*T*,*T*,*T* dimer. At high temperature, the dimer rearranges through the cleavage of the *H*‐*T* bond which recombine at the *T*‐*T* positions. In the case of R = Me, however, because the dispersion stabilization in **20a** is not sufficient to maintain the closed conformation, oligomers are formed through the open form **20b**. The different regioselectivity observed in the case of R = *c*‐Hx can be interpreted as follows. The initial bond formation occurs at the *H‐T* ends owing to the large steric hindrance due to the sterically demanding cyclohexyl groups, forming intermediate **20c**. Subsequent ring closure also takes place at the *H‐T* positions to produce the *H,T,H,T* dimer to avoid steric hindrance. Similar to *H,T,H,T*‐**12b**, the *H,T,H,T* dimer rearranges to the *H,T,T,T* isomer through the cleavage of the *H*‐*T* bond which recombine at the *T*‐*T* positions.

Recently, the significant role of London dispersion forces has been reported in controlling the reactivity and selectivity of organic reactions.^[^ ^36^, ^37^, ^38^, ^39^ ^]^ While the majority of these reactions involve aryl‐aryl interactions, the critical roles of alkyl–alkyl and/or alkyl–aryl interactions are also appreciated in reactions such as the association of triarylmethyl radicals,^[^ ^40^, ^41^ ^]^ catalytic hydroamination,^[^ ^42^ ^]^ and benzyne cycloaddition.^[^ ^43^, ^44^ ^]^ We attribute the remarkable effects of alkyl groups on the reaction selectivity and regioselectivity of the cyclodimerizations to attractive dispersion forces between the hydrogen atoms of the alkyl groups and the π‐system of the indenofluorene or fluorenofluorene framework in diradical intermediates **20a** and **20c** (indicated by blue hashed lines shown in Scheme 6). It is expected that these attractive interactions bring the radical centers close to each other, thereby facilitating the second C─C bond formation. Otherwise, rotation around the first C─C bond separates the radical centers, as in **20b**, leading to the formation of oligomers through reaction with another monomer molecule. Note that the matching of the sizes of the alkyl groups and the π‐system is important for attaining efficient interactions, which in turn leads to the formation of different regioisomers of the dimers. In most cases, the *H,T,T,T* isomers, which are deduced to be more stable than the *H,T,H,T* isomers (Table 3), are formed predominantly, with two exceptions where the less stable *H,T,H,T* isomers predominate in the case of TCHS‐derivatives. The different regioselectivity can be understood by assuming the different positions of the initial bond formation (Scheme 6), which depend on the steric bulkiness of the cyclohexyl groups.

**Figure 8:**
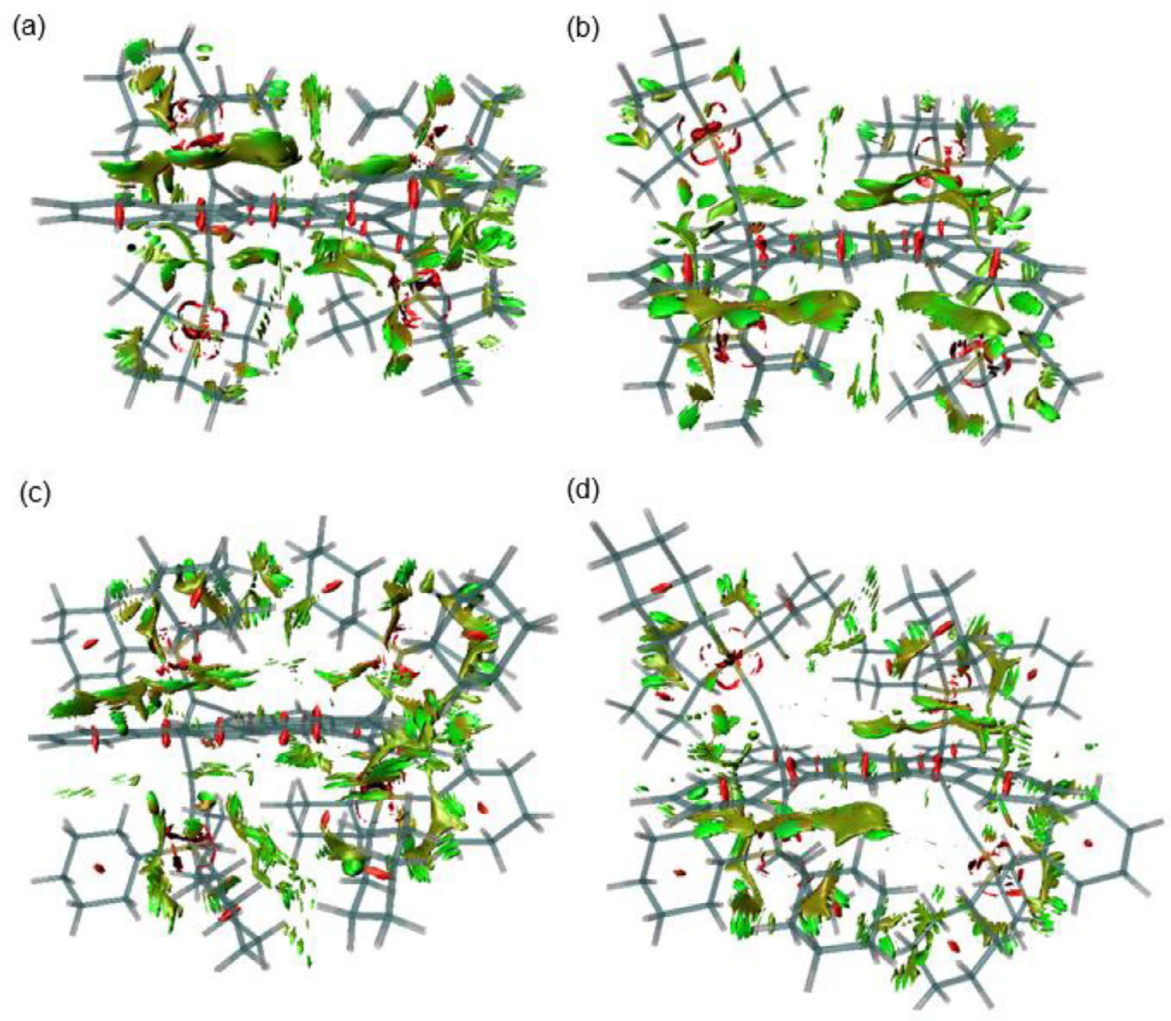
NCI plots for the theoretical optimized structures of the cyclodimers. a) *H,T,T,T*‐**12a**, b) *H,T,H,T*‐**12b**, c) *H,T,T,T*‐**16a**, and d) *H,T,H,T*‐**18b**. The regions showing van der Waals interactions are represented by green isosurfaces, whereas the regions showing attractive or repulsive forces are displayed as blue or red isosurfaces, respectively, (isosurface values 0.50 a.u.).

Although the transition state geometries were not examined due to the complexity arising from numerous possible conformations with similar energies, we estimated the stabilization from dispersion forces in the cyclodimers using noncovalent interaction region plots (NCI plots) for the theoretically optimized structures.^[^ ^45^ ^]^ Figure 8 displays the 3D NCI plots for *H,T,T,T*‐**12a**, *H,T,H,T*‐**12b**, *H,T,T,T*‐**16a**, and *H,T,H,T*‐**18b** as representative examples. Figures S14 and S15 compile the 3D and corresponding 2D plots for all cyclodimers. As shown in the NCI plots, attractive noncovalent interactions are observed between the alkyl groups and the π‐conjugated framework of the counterpart molecule, in addition to interactions between and within the trialkylsilyl groups. These results, combined with the short intramolecular contacts observed in the crystal structures of the cyclodimers, strongly indicate that the dimers bearing TIPS and TCHS groups are stabilized by dispersion forces. The 2D plots (Figure S15) also indicate increasing stabilization due to noncovalent interactions with increasing size of the alkyl groups. Although significant stabilization is indicated in the dimers bearing TIPS and TCHS groups, the difference between the *H,T,T,T* and *H,T,H,T* isomers is not apparent from these plots.

Finally, it should be pointed out that the present cyclodimerizations take place under kinetic control and are distinct from the dynamic covalent chemistry (DCC) mechanism, in which error corrections occur through bond dissociation‐recombination processes leading to thermodynamically favored products.^[^ ^46^, ^47^, ^48^ ^]^ Recent advances have established control over the cyclo‐oligomerization of *m*‐QDM derivatives,^[^ ^49^, ^50^, ^51^, ^52^, ^53^ ^]^ which is otherwise challenging because of their high reactivity due to open‐shell character.^[^ ^54^, ^55^ ^]^ The present results consequently shed light on controlling regio‐ and stereoselective cycloaddition reactions that proceed under kinetic control conditions through diradical intermediates, a feat that is generally difficult to achieve.

## 3 Conclusion

A remarkable effect of alkyl groups (methyl, isopropyl, or cyclohexyl) was observed on the reactivity and regioselectivity of the cyclodimerization of (trialkylsilyl)ethynyl derivatives of indeno[2.1‐*b*]fluorene and fluoreno[2,3‐*b*]fluorene, which are open‐shell singlet molecules incorporating an *m*‐quinodimethane substructure. Cyclodimers with an *anti*‐configuration were obtained in yields ranging from virtually zero to quantitative, depending on the balance between the sizes of the diradicaloid frameworks and the alkyl groups. The positions of bond formation (*Head* versus *Tail*) arising from the ethynyl–allenyl radical resonance were also affected by this size balance. Cyclodimers were formed in high yields only when an optimal size balance was achieved; otherwise, intractable oligomers were formed. The crystal structures of some of the dimers and theoretical calculations for the cyclodimers indicate the existence of strong intramolecular noncovalent interactions between the alkyl groups and the π‐framework of the counterpart molecule within the cyclodimers. These results indicate that the reaction pathways of cyclodimerization, including the regioselectivity of bond formation, are controlled by London dispersion forces. Therefore, dispersion forces were shown to be a powerful tool for controlling the reactivity of diradicals under kinetic control.

## 4 Experimental Section

### Cyclodimerization under standardized condition

The preparative‐scale cyclodimerizations of the **2,1‐IF** and **2,3‐FF** derivatives are described in the Supporting Information. To determine the yields of the cyclodimerization reactions under identical conditions, small‐scale reactions were undertaken using the following procedure. A dilute solution of the diol (0.03 mmol) in toluene (20 mL) was added dropwise to a suspension of SnCl_2_ (0.14 mmol) in toluene (5 mL) over 20 minutes at room temperature. During the reaction and subsequent workup, all apparatus were covered with aluminum foil to protect the products from exposure to room light. After stirring for 150 minutes, the mixture was passed through a short alumina column (eluted with toluene). After solvent evaporation, a weighed portion of the crude product was dissolved in CDCl_3_ containing a known amount of bromoform. The relative amounts of the oligomers were judged by comparing the apparent intensities of the broad oligomer signals with respect to the sharp signals of the cyclodimers in the ^1^H NMR spectra. Based on the relative integration ratios of the signals corresponding to a representative aromatic proton of the cyclodimers and that of CHBr_3_, the yields of the dimers were determined and are listed in Table 2.

## Conflict of Interest

The authors declare no conflict of interest.

## Supporting information



Supporting Information

Supporting Information

Supporting Information

## Data Availability

The data that support the findings of this study are available in the supplementary material of this article.
