# Supplementary material for: Taming m‐Quionodimethanes by Dispersion Force: Cyclodimerization of (Trialkylsilyl)Ethynyl‐Substituted Indeno[2,1‐b]Fluorenes and Fluoreno[2,3‐b]Fluorenes
Source: Chemistry. 2025 Aug 19;31(54):e02053. doi: 10.1002/chem.202502053 (PMC12462229; doi:10.1002/chem.202502053)
Supplement: Supplementary file 1 — Supporting Information [file CHEM-31-e02053-s001.docx]

**Supporting Information for**

**Taming *m*-Quionodimethanes by Dispersion Force: Cyclodimerization of (Trialkylsilyl)Ethynyl-Substituted Indeno[2.1-*b*]Fluorenes and Fluoreno[2.3-*b*]Fluorenes**

Ching-En Hung,*^a^* Hsin-Chung Lin,*^a^* Rumi Ozawa,*^b^* Yogajivan Rout,*^a^* Hirokazu Miyoshi,*^c^* Yutaka Ie,*^b^* Ichiro Hisaki,*^d^* Yen-Ju Cheng,*^a^* Yoshito Tobe*^a,b,c^*^*^

*a* Department of Applied Chemistry, National Yang Ming Chiao Tung University, 1001 Ta Hsueh Road, Hsinchu 30010, Taiwan.

*b* Nanoscience and Nanotechnology Center, The Institute of Scientific and Industrial Research (SANKEN), The University of Osaka, Ibaraki, Osaka 567-0047, Japan.

*c* Division of Frontier Materials Science, Graduate School of Engineering Science, The University of Osaka, Toyonaka, Osaka 560-8531, Japan.

*d* Division of Chemistry, Graduate School of Engineering Science, The University of Osaka, Toyonaka, Osaka 560-8531, Japan.

**Contents**

1. Molecule Orbital Diagrams Possible Structures of Cyclodimers

2. Possible Structures of Cyclodimers

3. Experimental Details

4. ^1^H NMR Spectra of Crude Products of Cyclodimerization

5. MALDI-TOF Mass Spectra of Oligomers of **4c**, **9c**, **9d** and **9e**

6. X-Ray Crystallographic Analyses of Cyclodimers

7. Short Intramolecular Contacts in Crystal Structures of Cyclodimers

8. Theoretical Geometries of Cyclodimers

9. NCI Plots for Cyclodimers

10. NMR Spectra of New Compounds

11. References

12. Atomic Coordinates of Theoretically Optimized Structures (separate file)

**1.** **Molecule Orbital Diagrams**

**
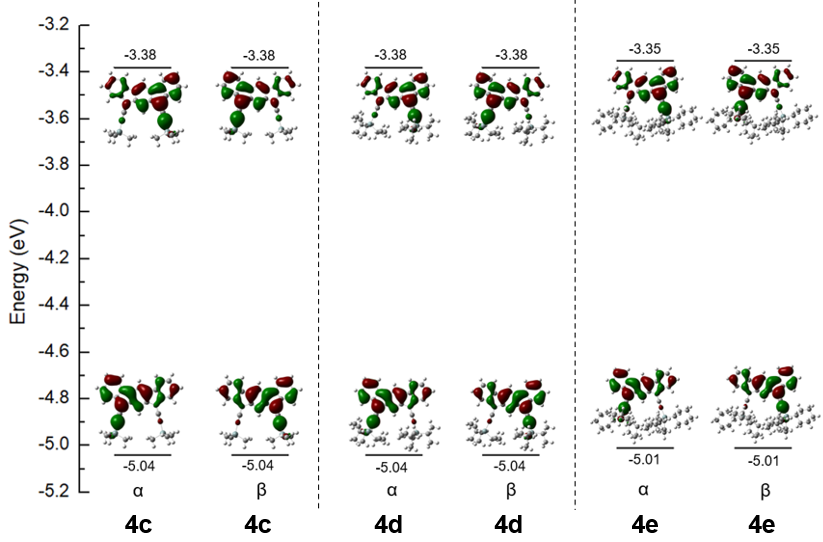
**
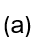


**
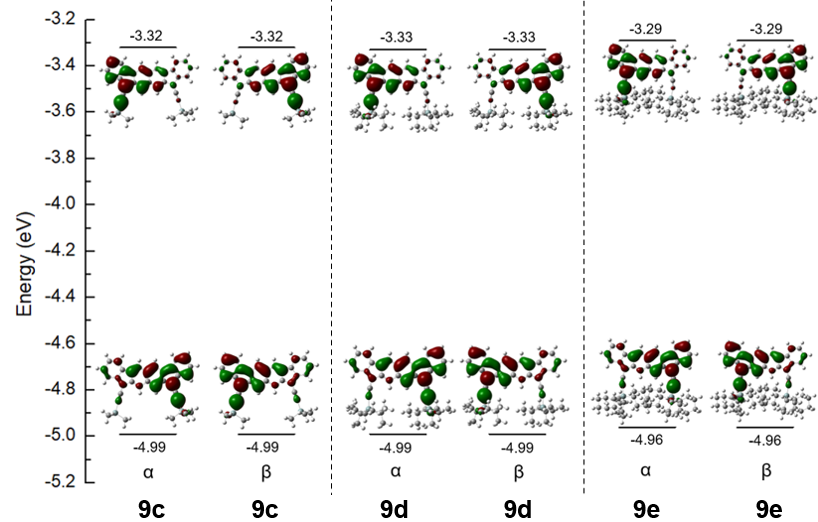
**
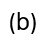


**Figure S1.** Frontier molecular orbital diagrams for closed-shell singlet states of (a) indeno[2,1-*b*]fluorene derivatives **4c**–**4d** and (b) fluoreno[2,3-*b*]fluorene derivatives **9c**–**9d**. Calculations were done by the B3LYP-D3(BJ)/6-311G(d,p) level of theory.

**2. Possible Structures of Cyclodimers**

(a)

(b)

**Figure S2.** Possible structures of cyclodimers of (trialkylsilyl)ethynyl-substituted indeno[2,1-*b*]fluorene. (a) *Anti*-isomers, (b) *syn*-isomers

**3. Experimental Details**

**General Experimental Procedures**

Melting points were measured with a MANDARIN SCIENTIFIC MP-2D apparatus and are uncorrected. ^1^H and ^13^C NMR spectra were measured with a Aligent 400-MR DD2, Bruker AVANCEIII-400, JEOL JNM-ECZ500R/S1 or JEOL ECS-400 spectrometer at 30 °C unless otherwise stated in chloroform-*d*, toluene-*d*_8_ (at 55 °C), 1,2-dichlorobenzene-*d*_4_ (at 100 °C), tetrachloroethane-*d*_2_ (at 100 °C). TMS was used as an internal standard in CDCl_3_, C_2_D_2_Cl_4_ and C_6_D_5_CD_3_. For the ^1^H NMR measurements, the residual solvent signals were used as references for C_2_D_2_Cl_4_ (6.00 ppm), C_6_D_5_CD_3_ (7.10, 7.01, 6.98, 2.09 ppm) and C_6_D_4_Cl_2_ (7.37, 7.10 ppm). IR spectra were recorded with Perkin Elmer Spectrum One FT-IR spectrophotometer. Mass spectrometry was conducted with a JEOL JMS-T200GC AccuTOF GCx and Thermo Scientific Orbitrap XL mass spectrometer in FD (Field Desorption) mode and with Shimadzu/KRATOS AXIMA-PERFORMANCE for laser desorption ionization−time-of-flight (LD-TOF). Column chromatography and TLC were performed with UNI-WARD SiliaFlash Irregular Silica Gel G60, 60-200 µm, 60 Å (R10040B) (70–230 mesh ASTM) and Merck silica gel 60 F254, respectively. For chromatography on aluminum oxide gel, Macherey-Nagel Aluminum Oxide 90 Neutral was used. 1,5-dibromo-2,4-dimethylbenzene,^1^ 2-(4-(tert-butyl)phenyl)-4,4,5,5-tetramethyl-1,3,2-dioxaborolane,^2^ 4,4''-di-tert-butyl-4',6'-dimethyl-1,1':3',1''-terphenyl,^3^ 3,6-dibromonaphthalene-2,7-diol,^4^ 2,7-Diacetoxy-3,6-dibromonaphthalene,^4^ 2,7-Diacetoxy-3,6-diphenylnaphthalene,^4^ 2,7-Dihydroxy-3,6-diphenylnaphthalene,^4^ 2,7-Bis(trifluoromethylsulfonyl)-3,6-diphenylnaphthalene,^4^ 2,7-Dicyano-3,6-diphenylnaphthalene,^4^ tricyclohexylsilane,^5^ (tricyclohexylsilyl)acetylene^6^ were prepared according to the literature methods. Other reagents were used as supplied. Tetrahydrofuran (THF), toluene, and dichloromethane were dried using a solvent purification system SD-300 (Asia Wong Enterprise). The diketones **IFDN**^7^ and **FFDN**^4^ were prepared according to the reported methods.

**Preparation of 2,8-Di-*tert*-butyl-10,12-dihydroindeno[2,1-*b*]fluorene-10,12-dione (di-*t*-Bu-indenofluorenedione)**

4,4''-Di-*tert*-butyl-4',6'-dimethyl-1,1':3',1''-terphenyl was prepared as follows. A two-neck flask was charged with 4,6-dibromo-*m*-xylene (1.40 g, 5.24 mmol), 2-[4-(*tert*-butyl)phenyl]-4,4,5,5-tetramethyl-1,3,2-dioxaborolane^2^ (3.00 g, 11.5 mmol), potassium carbonate (3.62 g, 26.2 mmol), tetrakis(triphenylphosphine)palladium (0) (620 mg, 0.50 mmol) and one drop of Aliquat 336. A mixture of THF (80.0 mL) and water (20.0 mL), degassed by bubbling nitrogen for 30 minutes, was added to dissolve the reagents. The resulting reaction mixture was refluxed overnight under a nitrogen atmosphere, then cooled to ambient temperature. The solution was extracted with dichloromethane and the organic layer was washed with brine. After drying over magnesium sulfate, the solvent was removed under reduced pressure. The product was purified by column chromatography on silica gel (hexane) to afford the terphenyl (1.82 g, 94%) as a white solid. The ^1^H NMR spectrum was consistent with literature values.^3^

A solution of the above product (1.21 g, 3.30 mmol) dissolved in pyridine (25.0 mL) was added potassium permanganate (3.00 g, 19.0 mmol) and the mixture was heated under refluxing for 3 h. Additional potassium permanganate (each 1.51 g, 9.55 mmol) in water (2.3 mL) was added three time, and then the mixture was heated for 3 h, 2 h, and 12 h before cooling to room temperature. The mixture was filtered through Celite and the filter was washed with a 10% aqueous sodium hydroxide solution. The combined filtrate was then acidified with 2M hydrochloric acid, resulting in the formation of precipitates. This solid was collected by suction filtration and dried under vacuum to give a crude diacid (1.02 g). This material was dissolved in sulfuric acid (25.0 mL) and the solution was stirred at 0 ℃ for 2 h. After the mixture was poured into ice, the precipitate was collected and washed with water followed by acetone to give di-*t*-Bu-indenofluorenedione (732 mg, 80%) as a yellow solid. mp 244.3–245.9℃; ^1^H NMR (CDCl_3_) δ 7.91 (s, 1H), 7.77 (br s, 2H), 7.66 (s, 1H), 7.59 (dd, *J* = 1.6, 8.0 Hz, 2H), 7.57 (d, *J* = 7.2 Hz, 2H), 1.37 (s, 18H); ^13^C NMR (CDCl_3_) δ 192.3, 154.4, 151.8, 140.2, 135.1, 134.5, 131.5, 121.8, 120.8, 120.1, 111.9, 35.3, 31.1; FT-IR *ṽ* 3057 (m), 2967 (w), 2962 (s), 2870 (s), 1681 (w), 1604 (w), 1472 (m), 1460 (w), 1369 (m), 1278 (w), 1186 (w), 1155 (w), 1094 (s), 1067 (s), 923 (s), 732 (m) cm^–1^; HRMS (FD) *m/z* calcd C_28_H_26_O_2_ [M^+^] 394.1927, found 394.1924.

**Preparation of (Trialylsilyl)ethynyl-substituted Dihydroindenofluorenediols 10a, 10b, 10b’, and 10c and (Trialylsilyl)ethynyl-substituted Dihydrofluorenofluorenediols 11a, 11b, and 11c.**

As a representative experiment, the preparation of compound **10b’** is described. A flame-dried three-neck flask was charged with (triisopropylsilyl)acetylene (217 mg, 1.20 mmol) and THF (6.0 mL) and the solution was cooled to 0 ˚C under a nitrogen atmosphere. A solution of *n*-BuLi in hexane (2.5 M, 0.5 mL, 1.25 mmol, 5.0 equiv) was added dropwise and the mixture was stirred for 20 min. Then the solution was transferred dropwise via a canula to a two-neck flask charged with di-*t*-Bu-indenofluorenedione (100.0 mg, 0.25 mmol) and in THF (6.0 mL) at room temperature, and the solution was stirred for 2 h. The reaction was quenched by addition of 10% aqueous solution of ammonium chloride and the mixture was extracted with dichloromethane. The organic layer was dried with magnesium sulfate and concentrated. The resulting solid was washed with pentane to give compound **10b’** (154 mg, 80%) as a colorless solid. mp 248.4–249.2 ℃; ^1^H NMR (CDCl_3_) δ 7.95 (s, 1H), 7.77 (s, 1H), 7.77–7.76 (m, 2H), 7.60 (d, *J* = 8.0 Hz, 2H), 7.45 (dd, *J* = 2.0, 8.0 Hz, 2H), 2.59 (s, 2H), 1.37 (s, 18H), 1.06–1.03 (m, 42H); ^13^C NMR (CDCl_3_) δ 152.4, 148.1, 147.3, 141.3, 136.0, 126.7, 121.5, 120.1, 119.8, 111.4, 107.3, 75.0, 85.0, 35.2, 31.5, 18.7, 11.2; FT-IR *ṽ* 3528 (w), 2942 (w), 2858 (w), 2322 (w), 2172 (w), 1603 (w), 1490 (w), 1461 (w), 1364 (w), 1264 (s), 1222 (s), 1196 (s), 1054 (s), 924 (s), 870 (s), 837 (s), 744 (m), 661 (m) cm^–1^; HRMS (FD) *m/z* calcd for C_50_H_70_O_2_Si_2_ [M^+^] 758.4909, found 758.4909.

By the identical procedure, compounds **10a**, **10b**, **10c**, and **11a**, **11b**, and **11c** were prepared from indenofluorenedione and fluorenofluorenedione, respectively and (trialkylsilyl)acetylenes. Diastereomer were estimated by ^1^H NMR: **10a** (4:1), **10b** (Estimation is not possible due to the overlap of peaks), **10b’** (Estimation is not possible due to the overlap of peaks), **10c** (1:10), **11a** (5:4), **11b** (2:3), **11c** (4:5).

**10a**: Using indenofluorenedione (250 mg, 0.90 mmol), *n*-BuLi (2.5 M, 3.2 mL), (trimethylsilyl)acetylene (873 mg, 8.90 mmol) and THF (25.0 mL), compound **10a** (357 mg, 83%) was obtained after washing with pentane as a colorless solid. decomp temp 235.4 ℃; ^1^H NMR (CDCl_3_, 400 MHz) δ 8.02, 8.01 (2s, 1H, 4:1), 7.82, 7.81 (2s, 1H, 1:4), 7.71 (d, *J* = 6.4 Hz, 2H), 7.68 (d, *J* = 7.2 Hz, 2H), 7.44 (t, *J* = 6.4 Hz, 2H), 7.38 (t, *J* = 8.4 Hz, 2H), 2.63 (s, 2H), 0.16 (2s, 18H); ^13^C NMR (CD_2_Cl_2_, 100 MHz) δ 147.6, 141.0, 138.6, 129.8, 128.9, 124.3, 120.4, 111.8, 104.7, 88.5, 74.6, 0.52; FT-IR *ṽ* 3502 (w), 2967 (w), 2920 (w), 2150 (w), 1611 (w), 1443 (w), 1238 (s), 1184 (s), 1096 (m), 1001 (s), 837 (s), 766 (s), 728 (s), 644 (s) cm^–1^; HRMS (FD) *m/z* calcd for C_30_H_30_O_2_Si_2_ [M^+^] 478.1784, found 478.1789.

**10b**: Using indenofluorenedione (150 mg, 0.53 mmol), *n*-BuLi (2.5 M, 1.1 mL, 2.7 mmol), (triisopropylsilyl)acetylene (601 mg, 3.30 mmol) and THF (15.0 mL), compound **10b** (295 mg, 86%) was obtained after washing with pentane as a colorless solid. ^1^H NMR (CDCl_3_, 400 MHz): δ = 7.92 (s, 1H), 7.65 (s, 1H), 7.64 (d, *J* = 7.4 Hz, 2H), 7.57 (d, *J* = 7.4 Hz, 2H), 7.35 (t, *J* = 7.4 Hz, 2H), 7.29 (t, *J* = 7.4 Hz, 2H), 2.88 (s, 2H), 1.06 (br s, 42H); ^13^C NMR (CDCl_3_, 100 MHz) δ = 148.0, 147.5, 141.1, 138.5, 129.6, 128.8, 124.3, 120.2, 120.1, 111.8, 106.9, 85.1, 74.8. 18.7, 11.2; FT-IR *ṽ* 3521, 2941, 2890, 2863, 2170, 1460, 1444, 1055, 989, 881, 785, 764, 748, 737, 727, 667, 643 cm^–1^; HRMS (FD) calcd for C_42_H_54_O_2_Si_2_ 646.3662, found 646.3653.

**10c**: Using indenofluorenedione (100 mg, 0.35 mmol), *n*-BuLi (2.5 M, 0.7 mL, 1.75 mmol), (tricyclohexylsilyl)acetylene (656 mg, 2.17 mmol) and THF (23.0 mL), compound **10c** (233 mg, 75%) was obtained after washing with pentane as a colorless solid. decomp temp 253.0 ℃; ^1^H NMR (CDCl_3_, 400 MHz) δ 7.96 (s, 1H), 7.84 (s, 1H), 7.70 (d, *J* = 7.2 Hz, 2H), 7.67 (d, *J* = 7.2 Hz, 2H), 7.42 (td, *J* = 7.6, 1.2 Hz, 2H), 7.37 (td, *J* = 7.2, 1.2 Hz, 2H), 2.62 (s, 2H), 1.77–1.68 (m, 30H), 1.30–1.20 (m, 30H), 0.88–0.82 (m, 6H); ^13^C NMR (CDCl_3_, 100 MHz) δ 148.3, 147.6, 141.3, 138.6, 129.7, 128.8, 124.4, 120.3, 120.2, 111.9, 107.0, 86.2, 75.0, 28.5, 28.4, 27.0, 23.1; FT-IR *ṽ* 3553 (w), 2917 (w), 2837 (w), 2155 (w), 2012 (s), 1698 (m), 1439 (w), 1309 (m), 1188 (w), 1154 (w), 1105 (m), 1041 (w), 996 (w), 874 (w), 841 (m), 732 (w), 694 (w) cm^–1^; HRMS (FD) *m/z* calcd for C_60_H_78_O_2_Si_2_ [M^+^] 886.5535, found 886.5535.

**11a**: Using fluorenofluorenedione (50.0 mg, 0.15 mmol), *n*-BuLi (2.5 M, 0.5 mL, 1.35 mmol), (trimethylsilyl)acetylene (150 mg, 1.53 mmol) and THF (10.0 mL), compound **11a** (55.4 mg, 70%) was obtained after washing with pentane as a colorless solid. decomp temp 146.4 ℃; ^1^H NMR (CDCl_3_, 400 MHz) δ 8.21, 8.19 (2s, 2H, 5:4), 8.07 (s, 2H), 7.79 (d, *J* = 8.0Hz, 2H); 7.77 (d, *J* = 8.0Hz, 2H); 7.47 (td, *J* = 7.6, 1.2 Hz, 2H), 7.42 (td, *J* = 7.6, 1.2 Hz, 2H), 2.70, 2.68 (2s, 2H, 5:4), 0.17 (2s, 18H, 5:4); ^13^C NMR (CDCl_3_, 100 MHz) δ 147.7, 147.6, 145.5, 145.4, 138.7, 138.6, 138.1, 138.0, 135.9, 134.4, 130.0, 129.4, 124.8, 124.7, 120.9, 120.8, 119.0, 105.1, 105.0, 89.18, 89.17, 74.8, 74.7, 0.0; FT-IR *ṽ* 3369 (w), 2962 (w), 2896 (w), 2163 (w), 1636 (w), 1242 (s), 1063 (w), 837 (s), 749 (s), 694 (w) cm^–1^; HRMS (FD) *m/z* calcd for C_34_H_32_O_2_Si_2_ [M^+^] 528.1935, found 528.1935.

**11b**: Using fluorenofluorenedione (50.0 mg, 0.15 mmol,), *n*-BuLi (2.5 M, 0.3 mL, 0.75 mmol), (triisopropylsilyl)acetylene (188 mg, 0.98 mmol) and THF (10.0 mL), compound **11b** (88.8 mg, 86%) was obtained after washing with pentane as a colorless solid. ^1^H NMR (CDCl_3_, 400 MHz): δ = 8.17 (2s, 2H, 1:1), 8.07 (br s, 2H), 7.79 (d, *J* = 7.4 Hz, 2H), 7.75 (d, *J* = 7.4 Hz, 2H), 7.46 (t, *J* = 7.4 Hz, 2H), 7.41 (t, *J* = 7.4 Hz, 2H), 2.72, 2.69 (2s, 2H, 1:1), 1.07–1.03 (br, 42H); ^13^C NMR (CDCl_3_, 100 MHz): δ = 147.9, 145.6, 138.7, 138.2, 135.9, 134.6, 130.0, 129.4, 124,6, 124.5, 120.9, 119.0, 107.3, 85.75, 74.8, 18.8, 11.3; FT-IR *ṽ* 3538, 3447, 2940, 2925, 2889, 2155, 1634, 1491, 1456, 1421, 1382, 1366, 1312, 1290, 1252, 1240, 1222, 1175, 1152, 1128, 1102, 1064, 1039, 1010, 994, 968, 946, 920, 902, 882, 776, 767, 749, 730, 712, 676, 662, 645, 621 cm^–1^; HRMS (FD) calcd for C_46_H_56_O_2_Si_2_ 696.3813, found 696.3812.

**11c**: Using fluorenofluorenedione (50.0 mg, 0.15 mmol), *n*-BuLi (2.5 M, 0.3 mL, 0.75 mmol), (tricyclohexylsilyl)acetylene (281 mg, 0.93 mmol) and THF (5.0 mL), compound **11c** (121 mg, 86%) was obtained after washing with pentane as a colorless solid. decomp temp 260.0 ℃; ^1^H NMR (CDCl_3_, 400 MHz) δ 8.17, 8.15 (2s, 2H, 4:5), 8.08 (s, 2H), 7.79 (d, *J* = 8.0 Hz, 2H), 7.76 (d, *J* = 7.6 Hz, 2H), 7.47 (td, *J* = 7.2, 1.2 Hz, 2H), 7.41 (td, *J* = 7.6, 1.2 Hz, 2H), 2.70, 2.69 (2s, 2H, 4:5), 1.75–1.69 (m, 30H), 1.31–1.14 (m, 30H), 0.86 (m, 6H); ^13^C NMR (CDCl_3_, 100 MHz) δ 147.9, 147.8, 145.6, 138.7, 138.1, 135.8, 134.5, 129.9, 129.3, 124.7, 124.5, 124.4, 120.8, 119.0, 107.4, 86.3, 74.8, 28.5, 28.3, 27.0, 23.0; FT-IR *ṽ* 3373 (w), 2913 (w), 2841 (w), 2159 (w), 1632 (w), 1439 (s), 1313 (w), 1083 (m), 1038 (w), 992 (s), 887 (w), 744 (s), 682 (s) cm^–1^; HRMS (FD) *m/z* calcd for C_64_H_80_O_2_Si_2_ [M^+^] 936.5691, found 936.5691.

**Formation of *H,T,T,T*-14a by Reductive Deoxygenation of Diol 10a_._** A solution of **10a** (100 mg, 0.21 mmol) in toluene (120 mL) was deaerated by bubbling nitrogen gas. This solution was added dropwise during a 20 min period to a suspension of SnCl_2_ (171 mg, 0.89 mmol) in toluene (30.0 mL), and the mixture was stirred at room temperature for 2.5 h. During the reaction and subsequent workup, all apparatuses were covered with aluminum foil. The resulting solution was filtered through a short column of alumina and the solvent was removed under reduced pressure to yield a brown solid (98.2 mg). separation with GPC (toluene as eluent) after a few cycles gave an oligomer fraction (49.4 mg) as a brown solid and *H,T,T,T***-14a** (8.3 mg, 8%) as a colorless solid. decomp temp 312.7 ℃; ^1^H NMR (CDCl_3_, 400 MHz) δ 8.25 (s, 1H), 8.17 (s, 1H), 7.96–7.94 (m, 3H), 7.87 (s, 2H), 7.80–7.79 (m, 2H), 7.74–7.71 (m, 2H), 7.54–7.37 (m, 9H) 0.23 (s, 9H), 0.16 (s, 9H), −0.50 (s, 9H), −0.54 (s, 9H); ^13^C NMR (CDCl_3_, 100 MHz) δ 204.7, 204.5, 203.5, 147.0, 146.8, 140.8, 140.0 139.7, 139.0, 138.8, 138.7, 138.5, 138.4, 138.2, 138.0, 137.9, 137.7, 137.5, 137.2, 128.7, 127.9, 127.3, 127.2, 127.14, 127.11, 126.7, 126.6, 126.1, 123.4, 123.3, 122.5, 120.4, 120.1, 120.0, 119.0, 115.3, 112.2, 111.9, 111.8, 105.5, 104.8, 104.7, 104.3, 103.3, 103.0, 87.8, 54.2, −0.01, −0.1, −0.4, −0.5, −0.9; FT-IR *ṽ* 2924 (w), 2846 (w), 2163 (w), 1906 (w), 1450 (w),1239 (s), 840 (s), 767 (s), 735 (s) cm^–1^; HRMS (FD) *m/z* calcd for C_60_H_56_Si_4_ [M^+^] 888.3459, found 888.3453.

**Formation of *H,T,T,T*-12a by Reductive Deoxygenation of Diol 10b.** A solution of **10b** (130 mg, 201 μmol) in toluene (140 mL) was deaerated by bubbling nitrogen gas. This solution was added to solid SnCl_2_ (170 mg, 0.89 mmol) placed in another flask, and the mixture was stirred under a nitrogen atmosphere at room temperature for 4 h. After addition of magnesium sulfate, the mixture was filtered. The filtrate was evaporated under reduced pressure using methanol as azeotropic co-solvent and the residue was passed through a column of alumina to give *H,T,T,T***-12a** as a pink solid (118 mg, 96%). An analytical sample was obtained by repeated recrystallization from chloroform to remove trace mount of the *H,T,H,T* isomer: decomp temp 343˚C; ^1^H NMR (CDCl_3_, 400 MHz): δ = 8.21 (s, 1H), 8.14 (s, 1H), 7.99 (s, 1H), 7.98 (s, 1H), 7.96–7.81 (m, 6H), 7.65–7.60 (m, 2H), 7.51–7.31 (m, 8H), 1.45–1.36 (m, 3H), 1.24–1.19 (m, 3H), 1.17 (d, *J* = 7.4 Hz, 9H), 1.10 (d, *J* = 7.4 Hz, 9H), 1.02 (d, *J* = 7.4 Hz, 9H), 0.93 (d, *J* = 7.4 Hz, 9H), 0.85 (d, *J* = 7.4 Hz, 9H), 0.72–0.65 (m, including d, 12H), 0.61 (d, *J* = 7.4 Hz, 9H), 0.54 (d, *J* = 7.4 Hz, 9H), 0.51–0.39 (m, 3H); ^13^C NMR (CDCl_3_, 100 MHz): δ = 207.7, 206.5, 205.0, 148.6, 147.4, 141.0, 140.1, 139.9, 139.5, 139.0, 138.7, 138.61, 138.57, 138.5, 138.4, 138.3, 138.2, 137.6, 137.5, 128.8, 127.3, 127.2, 127.1, 127.0, 126.6, 126.5, 123.7, 123.6, 123.5, 120.6, 120.3, 120.1, 119.9, 119.7, 118.4, 111.8, 111.7, 108.0, 107.7, 105.5, 103.6, 103.5, 99.6, 99.4, 82.3, 56.3, 19.9, 19.39, 19.38, 19.24, 19.16, 19.1, 18.5, 12.6, 12.5, 12.4, 11.0; IR 2923, 2863, 2853, 1907, 1734, 1463, 1443, 1378, 1364, 1278, 1252, 1234, 1218, 1075, 1055, 1032, 1019, 996, 883, 736 cm^–1^; HRMS (FD) calcd for C_84_H_104_Si_4_ 1224.7215, found 1224.7219.

**Photoisomerization of *H,T,T,T*-12a to *H,T,H,T*-12b**. A solution of *H,T,T,T***-12a** in CDCl_3_ in an NMR tube was left under room light at room temperature. The course of isomerization to *H,T,H,T***-12b** was monitored by ^1^H NMR spectroscopy, which showed complete transformation after several hours depending on the concentration. Alternatively, a solution of the *H,T,T,T* isomer in toluene-*d*_8_ was irradiated with a LED lamp (excitation maxima at 450 and 550 nm). After 10 min, the ^1^H NMR spectrum showed complete conversion to the *H,T,H,T* isomer. An analytical sample was obtained by recrystallization from chloroform: decomp temp 343˚C; ^1^H NMR (CDCl_3_, 400 MHz): δ = 8.43 (s, 2H), 8.05 (s, 2H), 7.89 (t, *J* = 8.0 Hz, 4H), 7.80 (dd, *J* = 3.2, 6.8 Hz, 4H), 7.47 (t, *J* = 12.8 Hz, 2H), 7.40–7.32 (m, 6H), 0.93 (d, *J* = 7.6 Hz, 36H), 0.79–0.77 (m, 12H), 0.66 (d, *J* = 7.2 Hz, 36H overlap with impurity signals); ^13^C NMR (CDCl_3_, 100 MHz): δ = 209.7, 149.1, 147.3, 142.1, 140.0, 139.0, 138.9, 137.8, 127.1, 127.0, 126.9, 119.9, 119.6, 112.5, 111.0, 108.7, 105.3, 80.6, 54.7, 30.8, 29.7, 19.9, 19.0, 18.3, 18.2, 11.2; IR 2941, 2879, 2863, 2154, 1908, 1461, 1442, 1422, 1341, 1278, 1193, 1076, 1018, 919, 881, 737, 675, 603 cm^–1^; HRMS (FD) calcd for C_84_H_104_Si_4_ 1224.7215, found 1224.7219.

**Thermal Isomerization of *H,T,H,T*-12b to *H,T,T,T*-12a.** By heating the *H,T,H,T* isomer in C_2_D_2_Cl_4_ in an NMR tubes at 70 °C, complete isomerization to the *H,T,T,T* isomer was observed by ^1^H NMR spectroscopy after 48 hours, showing identical ^1^H NMR spectrum with its authentic sample.

**Thermal Isomerization of *H,T,T,T*-12a to *T,T,T,T*-12c**. Heating the *H,T,T,T* isomer in toluene-*d*_8_ at 100 °C resulted in the formation of precipitates on the surface of the solution. The ^1^H NMR spectrum of the solution indicate the formation of the *T,T,T,T* isomer together with unidentified products. The solid product was collected by filtration and washed with CHCl_3_. Due to the very limited solubility of the product even at high temperature, only ^1^H NMR spectrum was measured, which showed high symmetry consistent with the *T,T,T,T* isomer. decomp temp 342˚C; ^1^H NMR (C_2_D_2_Cl_4_, 400 MHz): δ = 8.15 (s, 2H), 7.87 (s, 2H), 7.86 (d, *J* = 6.0 Hz, 4H), 7.58 (d, *J* = 7.2 Hz, 4H), 7.36 (t, *J* = 6.4 Hz, 10H), 1.22–1.19 (m, overlap with impurity signals), 1.05 (d, *J* = 6.8 Hz, 36H), 0.98 (d, *J* = 7.2 Hz, 36H); FT-IR *ṽ* 2945, 2864, 1902, 1452, 1440, 1384, 1278, 1233, 1071, 1018, 997, 920, 887, 777, 731, 671 cm^–1^; HRMS (FD) calcd for C_84_H_104_Si_4_ 1224.7215, found 1224.7219.

**Formation of *H,T,T,T*-13a by Reductive Deoxygenation of Diol 10b’.** A solution of **10b’** (22.7 mg, 29.9 μmol) in toluene (20.0 mL) was deaerated by bubbling nitrogen gas. This solution was slowly added dropwise during a 20 min period to a suspension of SnCl_2_ (25.6 mg, 0.14 mmol) in toluene (5.0 mL), and the mixture was stirred under a nitrogen atmosphere at room temperature for 2.5 h. During the reaction and subsequent workup, all apparatuses were covered with aluminum foil. The solution was filtered through a short column of alumina and the solvent was removed under reduced pressure to yield a yellow solid (21.2 mg). ^1^H NMR analysis of this crude product revealed it comprised predominantly the *H,T,T,T***-13a** together with a minor mount of *H,T,H,T***-13b**. The solid was washed with MeOH, and after solvent evaporation, pure *H,T,T,T***-13a** (15.6 mg, 69%) was obtained as a yellow solid. decomp temp 342.4 ℃; ^1^H NMR (CDCl_3_, 400 MHz) δ 8.12 (s, 1H), 8.05 (s, 1H), 7.96–7.94 (m, 4H), 7.83–7.8 (m, 3H), 7.76–7.73 (m, 3H), 7.51 (dd, *J* = 1.6, 8.0 Hz, 1H), 7.48–7.44 (m, 3H), 1.43 (2s, 36H, 25:11), 1.29–1.22 (m, 3H), 1.18 (d, *J* = 7.6 Hz, 9H), 1.11 (d, *J* = 7.6 Hz, 9H), 1.04 (d, *J* = 7.6 Hz, 9H), 0.95 (d, *J* = 7.6 Hz, 9H), 0.84 (d, *J* = 7.2 Hz, 9H), 0.70 (d, *J* = 6.8 Hz, 9H), 0.64–0.59 (m, 12H), 0.53–0.49 (m, 12H); ^13^C NMR (CDCl_3_, 100 MHz) δ 207.4, 206.3, 204.8, 150.4, 150.1, 149.7, 149.5, 148.3, 147.2, 139.8, 139.7, 139.4, 138.9, 138.5, 138.3, 138.1, 138.0, 137.9, 137.3, 136.1, 135.9, 135.2, 126.2, 124.8, 124.3, 124.1, 123.4, 120.8, 120.7, 120.6, 119.8, 119.5, 119.4, 119.2, 118.3, 111.2, 107.9, 107.7, 105.6, 103.8, 103.6, 99.2, 99.0, 81.8, 56.2, 35.1, 35.0, 34.9, 31.7, 31.5, 31.4, 31.3, 20.0, 19.8, 19.3, 19.2, 19.0, 18.8, 18.5, 18.4, 18.2, 12.9, 12.4, 12.2, 12.1, 11.2, 10.9; FT-IR *ṽ* 2949 (w), 2861 (w), 2166 (w), 1907 (w), 1610 (w), 1459 (s), 1358 (s), 1028 (m), 927 (s), 882 (s), 814 (s), 747 (m), 659 (m) cm^–1^; HRMS (FD) *m/z* calcd for C_100_H_136_Si_4_ [M^+^] 1448.9714, found 1448.9714.

**Photoisomerization of *H,T,T,T*-13a to *H,T,H,T*-13b.** *H,T,T,T***-13a** (10.0 mg, 6.90 μmol) was dissolved in degassed pentane (15.0 mL) and the solution was exposed to ambient light at room temperature for 72 h. The resulting precipitate was collected by filtration and washed with pentane to afford pure *H,T,H,T***-13b** (8.9 mg, 89%) as a colorless solid. decomp temp 334.6 ℃; ^1^H NMR (CDCl_3_, 400 MHz) δ 8.42 (s, 2H), 7.97 (s, 2H), 7.95 (d, *J* = 1.6 Hz, 2H), 7.83 (d, *J* = 0.9 Hz, 2H), 7.79 (d, *J* = 8.0 Hz, 2H), 7.70 (d, *J* = 8.4 Hz, 2H), 7.48 (dd, *J* = 1.6, 8.0 Hz, 2H), 7.43 (dd, *J* = 1.6, 8.0 Hz, 2H), 1.42 (s, 36H), 0.95–0.94 (m, 18H), 0.80 (d, *J* = 6.8 Hz, 18H), 0.71–0.66 (m, 30H), 0.62 (d, *J* = 6.4 Hz, 18H); ^13^C NMR (CDCl_3_, 100 MHz) δ 209.2, 150.3, 150.0, 148.8, 147.3, 140.2, 139.7, 139.3, 138.9, 138.6, 135.6, 126.0, 124.8, 124.2, 121.7, 120.3, 119.7, 119.3, 112.3, 110.6, 109.2, 105.6, 80.1, 54.9, 35.2, 35.1, 31.7, 31.6, 20.0, 19.0, 18.5, 18.3, 13.0, 11.4; FT-IR *ṽ* 2950 (w), 2862 (w), 2150 (w), 1904 (w), 1711 (w), 1611 (w), 1452 (w), 1364 (s), 1255 (w), 1067 (w), 1021 (w), 874 (s), 811 (s), 669 (w) cm^–1^; HRMS (FD) *m/z* calcd for C_100_H_136_Si_4_ [M^+^] 1448.9714, found 1448.9714.

**Thermal Isomerization of** ***H,T,H,T*-13b to *H,T,T,T*-13a.** *H,T,H,T***-13b** (10.0 mg, 6.90 μmol) was dissolved in degassed toluene (8.0 mL) and the solution was heated at 80 °C for 24 h. The solid obtained after evaporation of the solvent was revealed to be pure *H,T,T,T***-13a** (9.9 mg, 99%), showing identical ^1^H NMR spectrum with its authentic sample.

**Thermal Isomerization of** ***H,T,T,T*-13a to *T,T,T,T*-13c**. *H,T,T,T***-13a** (10.0 mg, 6.90 μmol) was dissolved in degassed toluene (15.0 mL) and refluxed at 110 °C for 16 h. After evaporation of the solvent, the residue was washed with chloroform to give the pure *T,T,T,T***-13c** (8.7 mg, 87%) as colorless solid. Due to the very limited solubility, the ^13^C spectrum of *T,T,T,T***-13c** could not be measured due to low solubility (ca. 0.5 mg / 1.0 ml 1,2-dichlorobenzene-*d*_4_ at 100 °C or lower). decomp temp 343.4 ℃. ^1^H NMR (C_6_D_4_Cl_2_, 500 MHz) δ 8.25 (s, 2H), 8.22 (s, 2H), 8.02–8.01 (m, 4H), 7.87 (d, *J* = 8.0 Hz, 4H), 7.56 (dd, J = 8.0, 2.0 Hz, 4H); FT-IR *ṽ* 2946 (w), 2862 (w), 1904 (w), 1866 (w), 1456 (w), 1360 (m), 1247 (s), 1063 (s), 1012 (s), 874 (s), 820 (s), 736 (m), 682 (s), 631 (s) cm^–1^; HRMS (FD) *m/z* calcd for C_100_H_136_Si_4_ [M^+^] 1448.9714, found 1448.9714.

**Formation of *H,T,H,T*-15b by Reductive Deoxygenation of Diol 10c.** A solution of **10c** (50.0 mg, 56.3 μmol) was deaerated by bubbling nitrogen gas. This solution was slowly added dropwise during a 20 min period to a suspension of SnCl_2_ (47.4 mg, 0.25 mmol) in toluene (10.0 mL), and the mixture was stirred under a nitrogen atmosphere at room temperature for 2.5 h. During the reaction and subsequent workup, all apparatuses were covered with aluminum foil. The solution was filtered through a short column of alumina and the solvent was removed under reduced pressure to yield *H,T,H,T***-15b** as a yellow solid (45 mg, 90%). decomp temp 318.4 ℃; ^1^H NMR (CDCl_3_, 400 MHz) δ 8.09 (s, 2H), 7.89–7.87 (m, 2H), 7.78 (d, *J* = 8.0 Hz, 2H), 7.69–7.66 (m, 4H), 7.46 (t, *J* = 6.8 Hz, 2H), 7.42–7.39 (m, 4H), 7.32 (d, *J* = 7.6 Hz, 2H), 7.23 (t, *J* = 7.6 Hz, 2H), 2.03 (m, 6H), 1.84–1.63 (m, 20H), 1.50–1.38 (m, 20H), 1.35–1.22 (m, 20H), 1.16–1.10 (m, 20H), 1.07–0.96 (m, 20H), 0.93–0.84 (m, 20H), 0.47 (t, *J* = 13.2 Hz, 6H); ^13^C NMR (CDCl_3_, 100 MHz) δ 205.1, 146.9, 145.8, 142.2, 142.1, 139.5, 138.4, 136.7, 129.6, 128.5, 126.9, 126.7, 124.1, 123.7, 121.0, 120.0, 119.0, 110.9, 105.1, 103.1, 98.1, 94.9, 85.9, 58.9, 28.6, 28.5, 28.45, 28.44, 28.4, 28.3, 28.25, 28.2, 28.0, 27.7, 27.0, 26.9, 23.3, 22.9, 22.8; FT-IR *ṽ* 2920 (w), 2841 (w), 2155 (w), 1908 (w), 1435 (w), 1096 (s), 1000 (s), 883 (s), 728 (s) cm^–1^; HRMS (FD) *m/z* calcd for C_120_H_152_Si_4_ [M^+^] 1705.0967, found 1705.0966.

**Thermal Isomerization of** ***H,T,H,T*-15b to *H,T,T,T*-15a_._** *H,T,H,T***-15b** (10.0 mg, 5.86 μmol) was dissolved in degassed toluene (7.5 mL) and the solution was heated at 55 °C for 120 h. The solid obtained after evaporation of the solvent was washed with cold pentane to give pure *H,T,T,T***-15a** as a yellow solid (8.8 mg, 88%). decomp temp 321.1 ℃; ^1^H NMR (CDCl_3_, 400 MHz) δ 8.23 (s, 1H), 8.18 (s, 1H), 7.99 (s, 1H), 7.95–7.93 (m, 4H), 7.90 (d, *J* = 6.8 Hz, 1H), 7.87 (d, *J* = 2.8 Hz, 1H), 7.85 (s, 1H), 7.71 (d, *J* = 6.4 Hz, 1H), 7.63 (d, *J* = 7.6 Hz, 1H), 7.48 (t, *J* = 7.2 Hz, 1H), 7.43–7.32 (m, 7H), 2.03–1.86 (m, 12H), 1.72–1.66 (m, 5H), 1.58–1.41 (m, 30H), 1.39–1.24 (m, 30H), 1.20–0.98 (m, 20H), 0.90–0.81 (m, 20H), 0.71–0.45 (m, 9H), 0.42–0.39 (m, 6H); ^13^C NMR (CDCl_3_, 100 MHz) δ 207.3, 206.1, 205.0, 149.6, 147.0, 140.8, 139.83, 139.76, 139.6, 139.5, 138.6, 138.4, 138.30, 138.29, 138.2, 138.1, 137.9, 137.4, 137.3, 128.5, 127.3, 127.1, 126.9, 126.8, 126.7, 126.2, 126.1, 124.2, 124.1, 124.0, 120.1,120.0, 119.8, 119.6, 118.4, 111.6, 111.3, 109.3, 106.2, 105.5, 103.3,103.2, 99.0, 98.3, 81.8, 56.2, 29.8, 29.3, 29.2, 29.0, 28.7, 28.7, 28.6, 28.5, 28.4, 28.2, 28.0, 27.8, 27.6, 26.9, 24.5, 24.2, 23.8, 22.8; FT-IR *ṽ* 2934 (w), 2850 (w), 2160 (w), 1907 (w), 1450 (w), 1030(s), 930 (s), 832 (m), 771 (m) cm^–1^; atHRMS (FD) *m/z* calcd for C_120_H_152_Si_4_ [M^+^] 1705.0967, found 1705.0966.

**Photoisomerization of** ***H,T,T,T*-15a to *H,T,H,T*-15b_._** A solution of *H,T,T,T***-15a**  in CDCl_3_ was exposed to room light at 30 ℃ for 72 h. The ^1^H NMR spectrum (Figure S3a) showed nearly complete conversion to *H,T,H,T***-15b** but with substantial formation of oligomers/polymers.


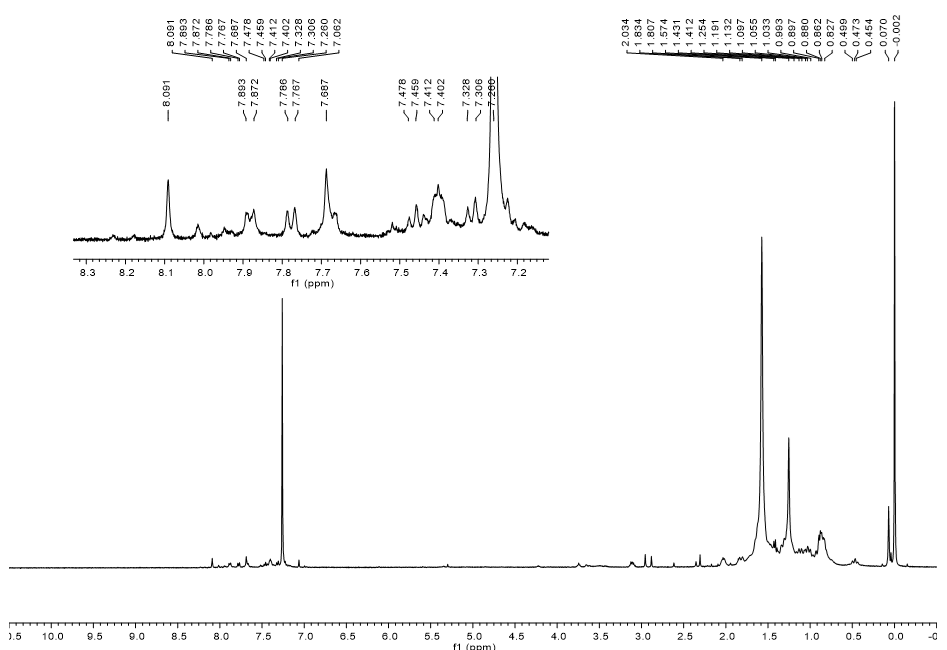


**Figure S3a.** ^1^H NMR spectrum (400 MHz, CDCl_3_, rt) obtained after photoirradiation (exposure to room light) of *H,T,T,T***-15a**. The main aromatic proton signals are identical with those of *H,T,H,T***-15b**.

**Formation of *H,T,H,T*-16b by Reductive Deoxygenation of Diol 11a.** A solution of **11a** (100 mg, 0.19 mmol) in toluene (120 mL) was deaerated by bubbling nitrogen gas. The solution was added slowly during a 20 min period to a suspension of SnCl_2_ (170 mg, 0.88 mmol) in toluene (30.0 mL), and the mixture was stirred at room temperature for 2.5 h. During the reaction and subsequent workup, all apparatuses were covered with aluminum foil. The mixture was filtered through a short column of alumina and the solvent was removed under reduced pressure to yield a brown solid (83.2 mg). Separation by GPC (toluene as eluent) after a few cycles gave oligomers (38.1 mg) as a brown solid and a yellow solid which was assumed to be *H,T,H,T***-16b** (0.6 mg, 1%). Due to the small amount of the cyclodimer, only the ^1^H NMR spectrum and HRMS cold be measured. ^1^H NMR (CDCl_3_, 400 MHz) δ 8.48 (s, 1H), 8.38 (s, 1H), 8.33 (s, 1H), 8.26 (s, 1H), 8.02–8.00 (m, 1H), 7.94 (d, *J* = 7.6 Hz, 1H), 7.90–7.88 (m, 2H), 7.57–7.53 (m, 1H), 7.52–7.50 (m, 1H), 7.47–7.44 (m, 2H), 0.19 (s, 9H), 0.34 (s, 9H). HRMS (FD) *m/z* calcd for C_68_H_60_Si_4_ [M^+^] 988.3767, found 988.3774.

**Formation of *H,T,T,T*-17a by Reductive Deoxygenation of Diol 11b.** A solution of **11b** (697 mg, 1.0 mmol) in toluene (350 mL) was deaerated by bubbling nitrogen gas. This solution was transferred to a two-neck flask containing solid SnCl_2_·2H_2_O (1015 mg, 4.5 mmol), and the mixture was stirred under a nitrogen atmosphere at room temperature for 4 h. After addition of magnesium sulfate, the mixture was filtered. The filtrate was evaporated under reduced pressure using methanol as azeotropic co-solvent and the residue was washed by hexane giving a yellow solid of the mixture of *H,T,T,T***-17a** containing the *H*,*T*,*H*,*T* isomer (34 mg, 5%). The hexane washing was evaporated, the residue was washed with ethyl acetate to give *H,T,T,T***-17a** as a yellow solid (103 mg, 15%): decomp temp 343˚C; ^1^H NMR (CDCl_3_, 400 MHz): δ = 8.55 (s, 1H), 8.47 (s, 1H), 8.37 (s, 2H), 8.35 (s, 1H), 8.27 (s, 1H), 8.24 (s, 1H), 8.19 (s, 1H), 8.04–7.98 (m, 3H), 7.92 (d, *J* = 8.5 Hz, 2H), 7.89–7.84 (m, 1H), 7.72–7.65 (m, 2H), 7.54–7.40 (m, 8H), 1.43–1.35 (m, 3H), 1.30–1.24 (m, 3H), 1.19 (d, *J* = 7.4 Hz, 9H), 1.11 (d, *J* = 7.5 Hz, 9H), 1.07 (d, *J* = 7.4 Hz, 9H), 0.97 (d, *J* = 7.5 Hz, 9H), 0.79 (d, *J* = 6.9 Hz, 9H), 0.66 (d, *J* = 6.4 Hz, 9H), 0.64 – 0.58 (m, 3H), 0.51 (d, *J* = 6.9 Hz, 9H), 0.42 (d, *J* = 6.5 Hz, 9H), 0.40–0.33 (m, 3H); ^13^C NMR (CDCl_3_, 100 MHz): δ = 206.6, 206.4, 205.6, 148.2 146.5, 140.7 139.8, 139.0 138.7, 138.6, 138.3, 138.2, 138.1, 137.9, 137.8, 137.24, 137.23, 136.6, 133.8, 133.5, 133.2, 132.9, 129.0, 128.4, 127.9, 127.7, 127.5, 127.4, 127.3, 127.0, 126.0 124.3, 124.1, 124.0, 123.0, 122.7, 122.1, 121.0, 120.8, 120.7, 119.0, 118.79, 118.78, 118.7, 108.8, 108.1, 105.3, 103.13 103.09, 100.81, 100.80, 83.0, 55.7, 19.5, 19.4, 19.3 19.24, 19.20, 19.1, 18.3, 18.0, 12.9, 12.6, 12.5, 11.2; IR 2940, 2920, 2862, 2160, 1903, 1455, 1380, 1364, 989, 902, 880, 749, 682, 659, 634 cm^–1^; HRMS (FD) calcd for C_92_H_104_Si_4_ 1324.7215, found 1324.7219.

**Photoisomerization of *H,T,T,T*-17a to *H,T,H,T*-17b**. A solution of *H,T,T,T***-17a** in CDCl_3_ in an NMR tube was left under room light at room temperature. The course of isomerization to *H,T,H,T***-17b** was monitored by ^1^H NMR spectroscopy, which showed complete transformation after several hours depending on the concentration. Alternatively, a solution of the *H,T,T,T* isomer in toluene-*d*_8_ was irradiated with a LED lamp (excitation maxima at 450 and 550 nm). After 10 min, the ^1^H NMR spectrum showed complete conversion to the *H,T,H,T* isomer. An analytical sample was obtained by recrystallization from chloroform: decomp temp 343˚C; ^1^H NMR (CDCl_3_, 400 MHz): δ = 8.55 (s, 2H), 8.40 (s, 2H), 8.36 (s, 2H), 8.22 (s, 2H), 7.98–7.97 (m, 2H), 7.95 (d, *J* = 7.2 Hz, 2H), 7.90 (d, *J* = 7.6 Hz, 2H), 7.84–7.82 (m, 2H), 7.52 (t, *J* = 8.0 Hz, 2H), 7.45–7.41 (m, 6H), 1.25 (s, 3H), 0.90 (d, *J* = 7.6 Hz, 16H), 0.73 (d, *J* = 6.8 Hz, 16H), 0.68 (d, *J* = 6.8 Hz, 16H), 0.60 (d, *J* = 6.0 Hz, 16H), 0.57–0.51 (m, 17H); IR 2942, 2864, 2162, 1909, 1460, 1381, 1068, 984, 902, 882, 751, 676, 661 cm^–1^; HRMS (FD) calcd for C_84_H_104_Si_4_ 1324.7215, found 1324.7501.

**Formation of *H,T,H,T*-18b by Reductive Deoxygenation of Diol 11c**. A solution of **11c** (28.1 mg, 30.1 μmol) in toluene (20.0 mL) was deaerated by bubbling nitrogen gas. This solution was added slowly during a 20 min period to a suspension of SnCl_2_ (25.6 mg, 13.5 μmol) in toluene (5.0 mL), and the mixture was stirred under a nitrogen atmosphere at room temperature for 2.5 h. During the reaction and subsequent workup, all apparatuses were covered with aluminum foil, except for the intended photoisomerization. The mixture was filtered through a short column of alumina and the solvent was removed under reduced pressure to leave a brown solid (27.2 mg). ^1^H NMR spectrum of the crude product revealed it contained mainly (Figure S3b.) *H,T,T,T***-18a** and *H,T,H,T***-18b** and the ratio is approximately 1:1.7**_,_** and a minor amount of oligomers. The crude product was dissolved in chloroform (10 mL) and the solution was exposed to ambient light at room temperature for 48 h. The resulting precipitate was collected by filtration and washed with chloroform to afford pure *H,T,H,T***-18b** (18.0 mg, 64%) as a colorless solid. Due to the very limited solubility, (ca. 1.0 mg / 1.0 ml toluene-*d*_8_ at 55 °C or lower) the ^13^C spectrum of *H,T,H,T***-18b** could not be measured. decomp temp 309.0 ℃; ^1^H NMR (C_6_D_5_CD_3_, 500 MHz) : δ = 8.01 (s, 2H), 7.97 (d, *J* = 7.5 Hz, 2H), 7.91 (s, 2H), 7.77 (d, *J* = 7.5 Hz, 2H), 7.66 (d, *J* = 7.5 Hz, 2H), 7.63 (d, *J* = 8.0 Hz, 2H), 7.36–7.29 (m, 8H), 7.25–7.22 (m, 4H), 1.74–1.64 (m, 40H), 1.56–1.51 (m, 30H), 1.40–1.32 (m, 6H), 1.20–1.00 (m, 50H), 0.65–0.62 (m, 6H); FT-IR *ṽ* 2917 (w), 2837 (w), 2155 (w), 1912 (w), 1448 (w), 992 (s), 887 (s), 732 (m) cm^–1^; HRMS
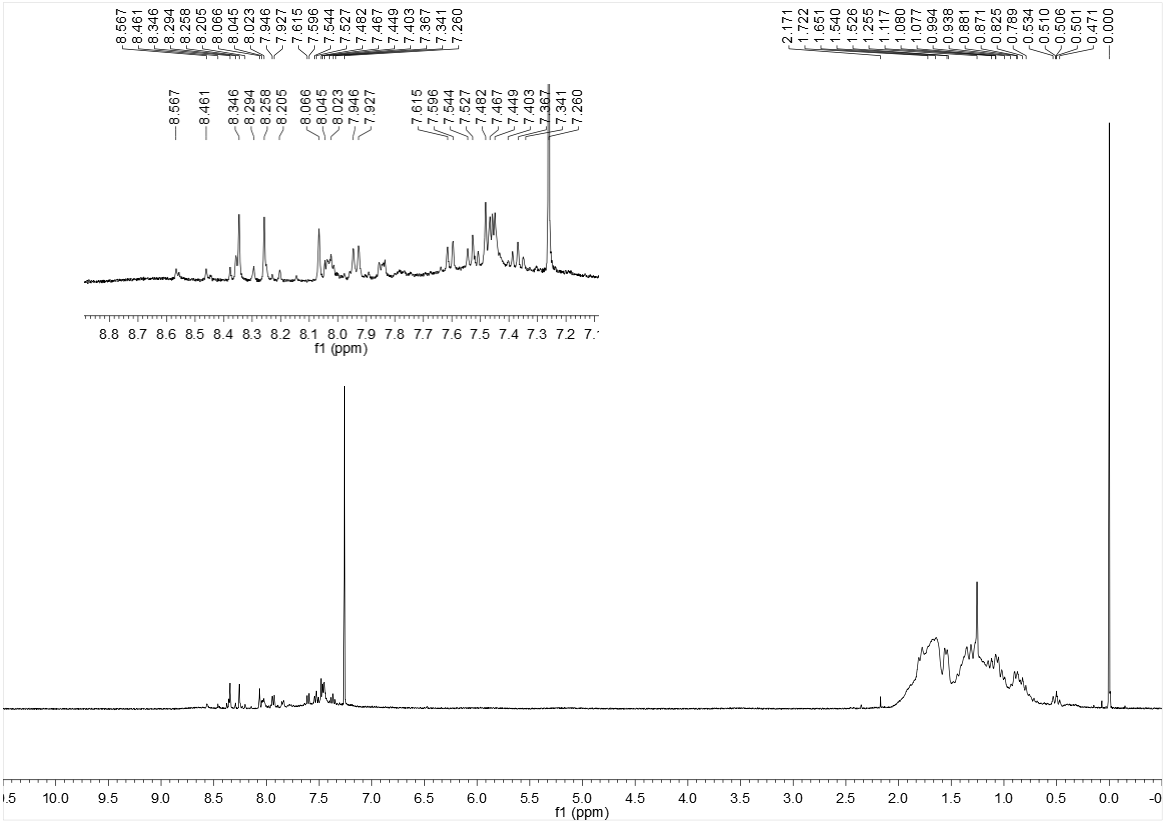
(FD) *m/z* calcd for C_128_H_156_Si_4_ [M^+^] 1805.1279, found 1805.1279.

**Figure S3b.** ^1^H NMR spectrum of crude products including cyclodimers **18a** and **18b** (400 MHz, CDCl_3_, rt).

**Thermal Isomerization of** ***H,T,H,T*-18b to *H,T,T,T*-18a**. *H,T,H,T***-18b** (20.0 mg, 0.011 mmol) was dissolved in degassed toluene (15.0 mL) and the solution was heated at 80 °C for 20 h. Evaporation of the solvent gave eventually pure *H,T,T,T***-18a** (19.7 mg, 99%) as colorless solid. decomp temp 315.3 ℃; ^1^H NMR (CDCl_3_, 400 MHz) δ 8.56 (s, 1H), 8.46 (s, 1H), 8.37 (s, 1H), 8.35 (s, 1H), 8.29 (s, 1H), 8.25 (s, 1H), 8.2 (s, 1H), 8.03–8.00 (m, 3H), 7.98–7.93 (m, 3H), 7.79–7.77 (m, 2H), 7.54 (t, *J* = 8.0Hz, 2H), 7.47–7.40 (m, 6H), 1.92–1.61 (m, 24H), 1.48–1.23 (m, 45H), 1.16–0.93 (m, 25H), 0.90–0.58 (m, 30H), 0.43–0.29 (m, 8H); ^13^C NMR (CDCl_3_, 100 MHz) δ 206.8, 206.3, 205.5, 148.5, 146.2, 140.8, 140.0, 139.2, 138.5, 138.3, 138.2, 138.1, 137.8, 137.7, 137.4, 137.3, 137.1, 137.0, 136.6, 133.8, 133.6, 133.1, 133.0, 130.1, 129.8, 128.7, 128.1, 127.6, 127.4, 127.3, 127.2, 125.0, 124.6, 124.5, 124.3, 122.5, 122.4, 122.0, 120.8, 120.7, 120.6, 119.0, 118.9, 118.8, 118.7, 109.1, 108.1, 105.1, 102.9, 102.7, 100.6, 100.4, 82.3, 55.6, 37.2, 32.8, 32.0, 30.1, 29.8, 29.6, 29.4, 29.2, 29.1 29.0, 28.9, 28.8, 28.6, 28.4, 28.24, 28.20, 28.1, 28.07, 28.02, 28.0, 27.2, 27.0, 26.97, 26.89, 24.7, 24.6, 24.4, 22.7, 19.8, 14.2, 1.1, 0.1; FT-IR *ṽ* 2917 (w), 2845 (w), 2159 (w), 1895 (w), 1439 (w), 1264 (s), 1109 (w), 996 (s), 899 (s), 728 (s), 675 (m), 615 (s) cm^–1^; HRMS (FD) *m/z* calcd for C_128_H_156_Si_4_ [M^+^] 1805.1279, found 1805.1279.

**
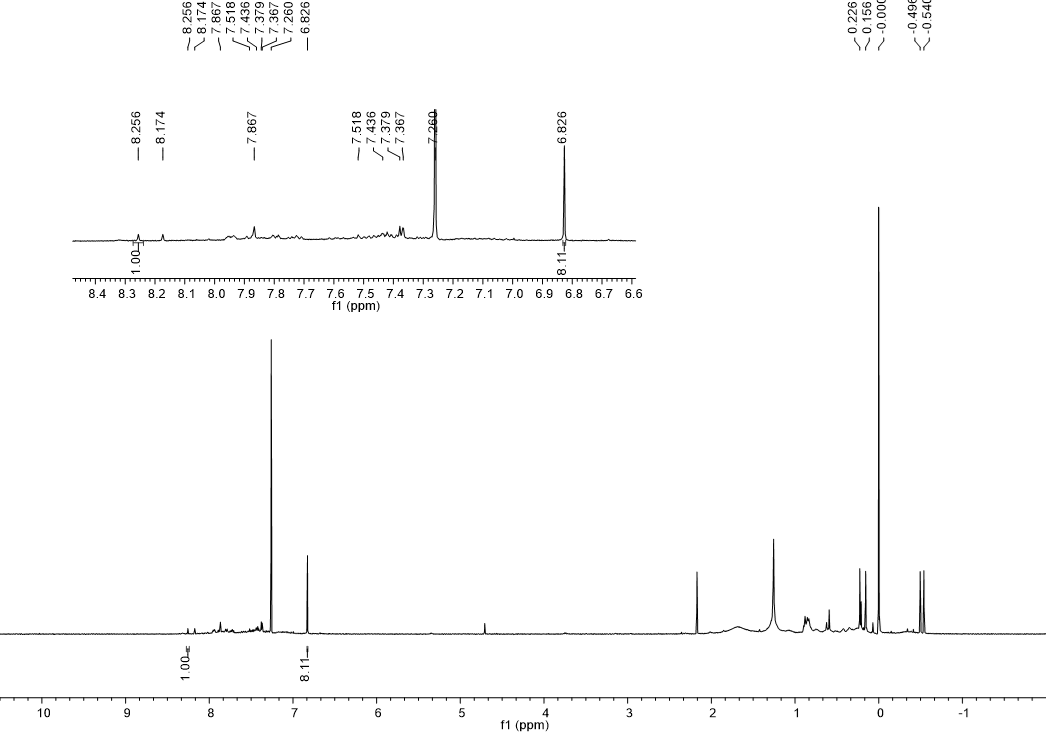
**
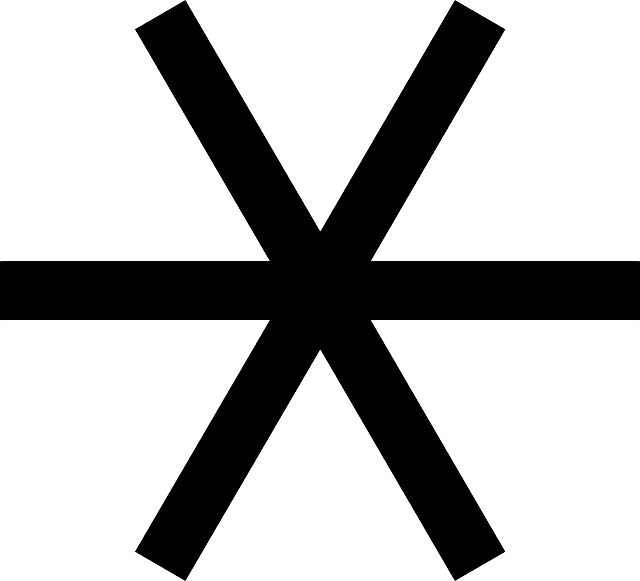
**4. ^1^H NMR Spectra of Crude Products of Cyclodimerization**

**
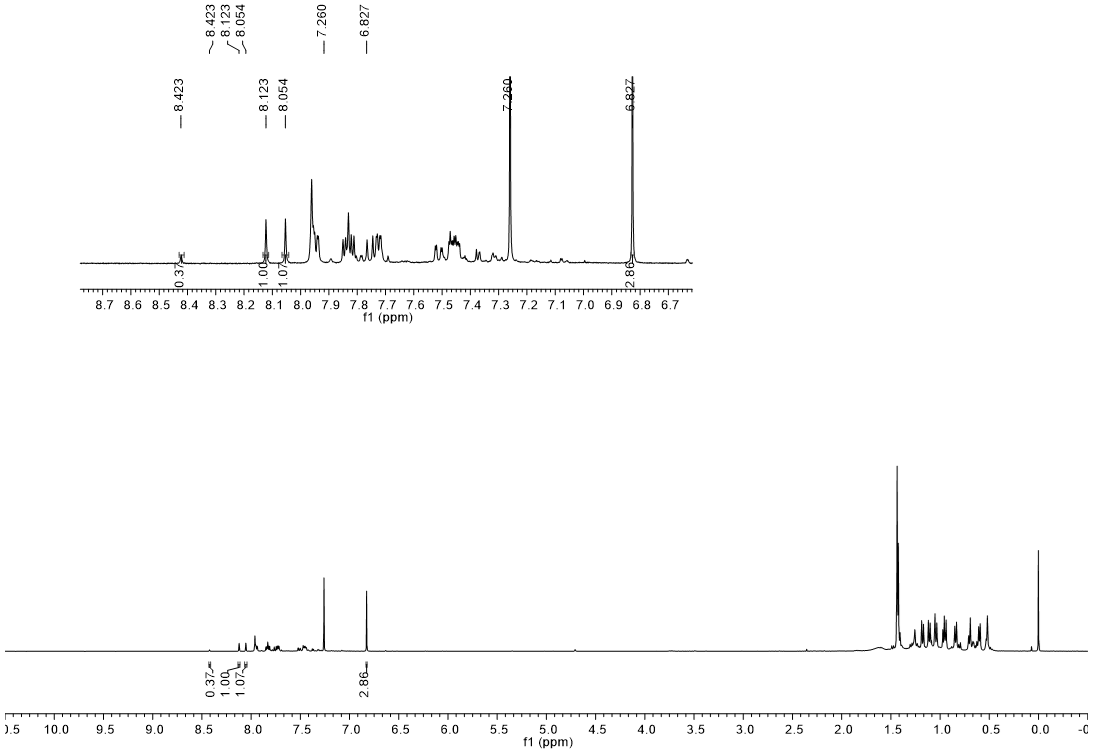
**
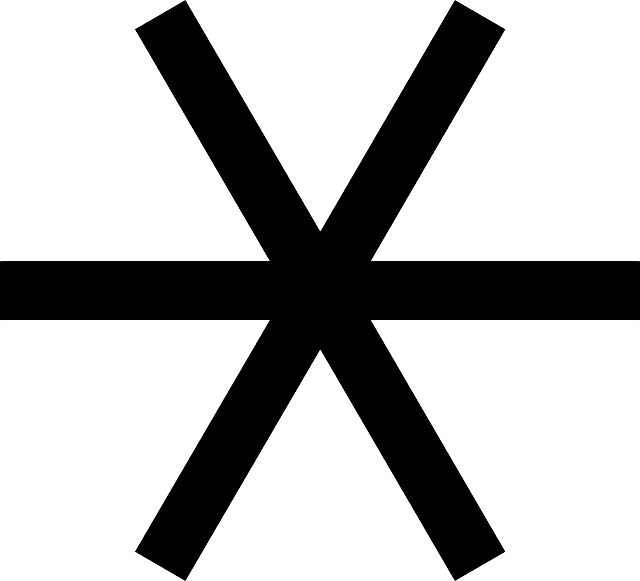
**Figure S4-1.** ^1^H NMR spectrum of crude products including oligomers with a small amount of cyclodimer **14a** (400 MHz, CDCl_3_, rt). The asterisk indicates the signal due to internal standard bromoform.

**Figure S4-2.** ^1^H NMR spectrum of crude products including mainly cyclodimer **13a** (400 MHz, CDCl_3_, rt) The asterisk indicates the signal due to internal standard bromoform.


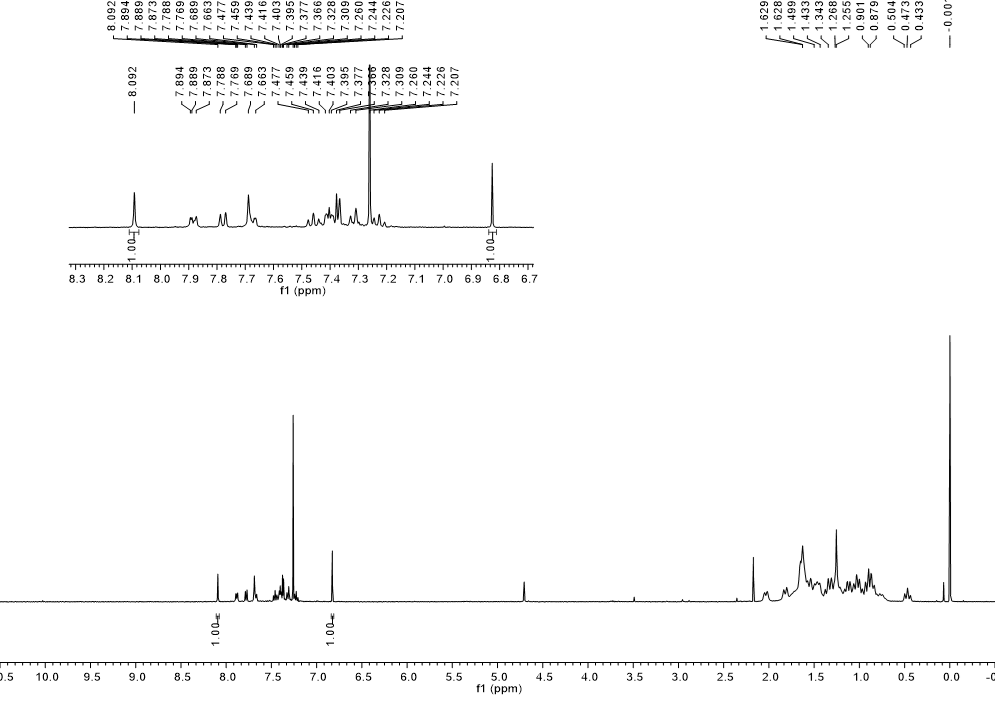

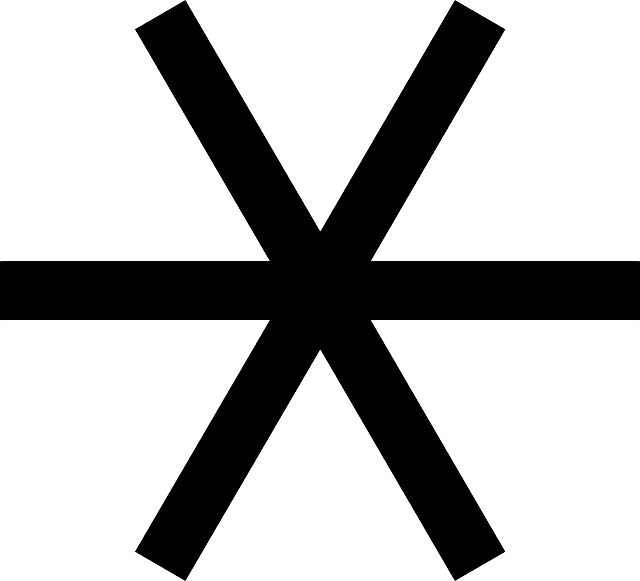

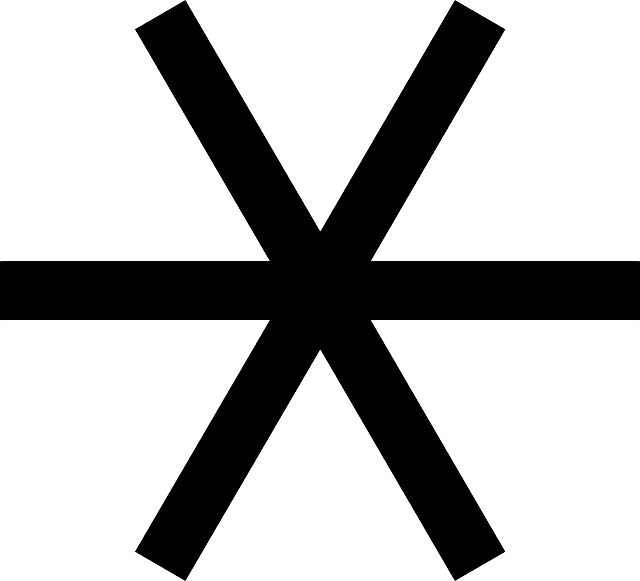
**
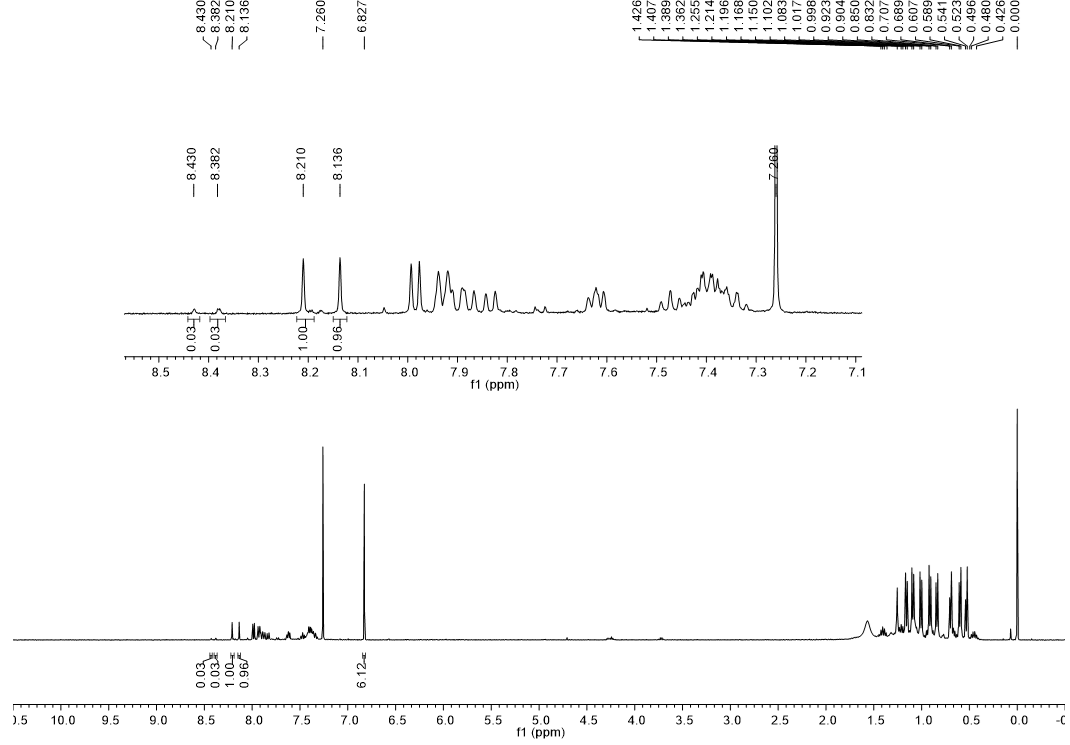
Figure S4-3.** ^1^H NMR spectrum of crude products including mainly cyclodimer **12a** (400 MHz, CDCl_3_, rt). The asterisk indicates the signal due to internal standard bromoform.

**Figure S4-4.** ^1^H NMR spectrum of crude products including mainly cyclodimer **15b** (400 MHz, CDCl_3_, rt). The asterisk indicates the signal due to internal standard bromoform.


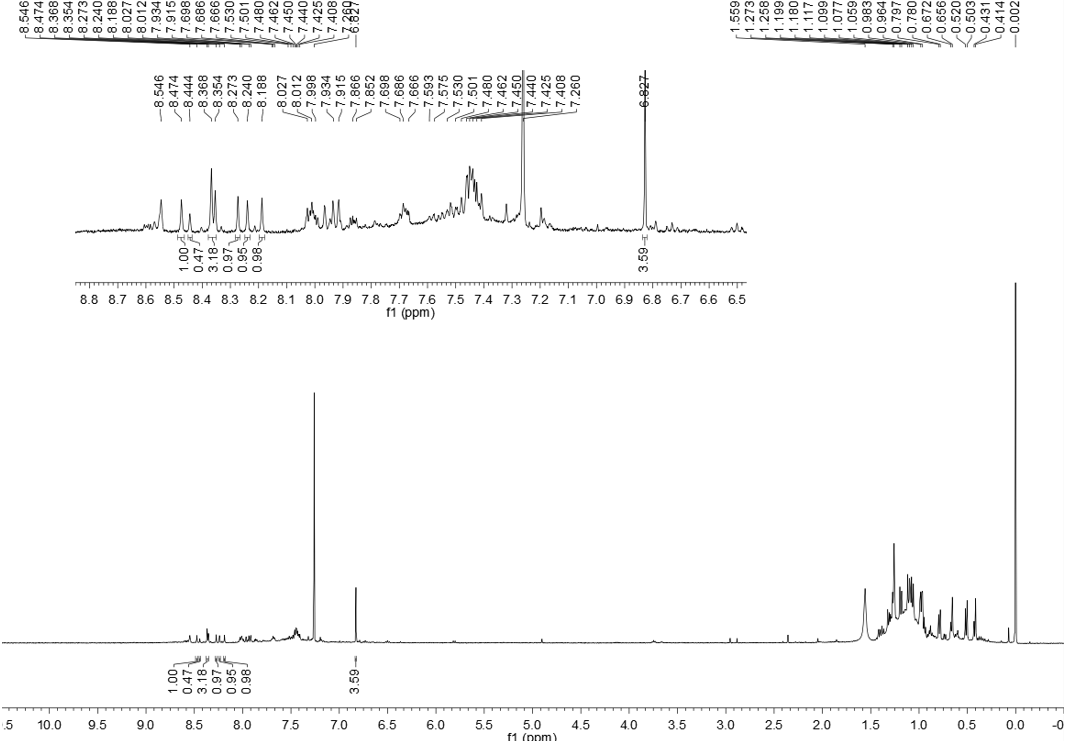

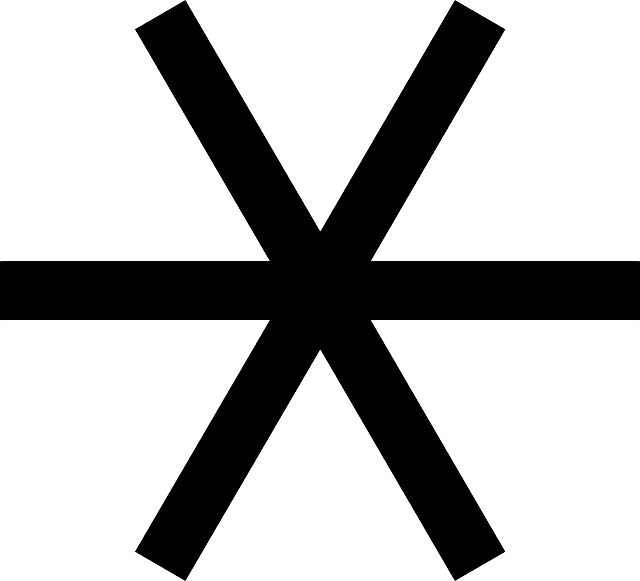

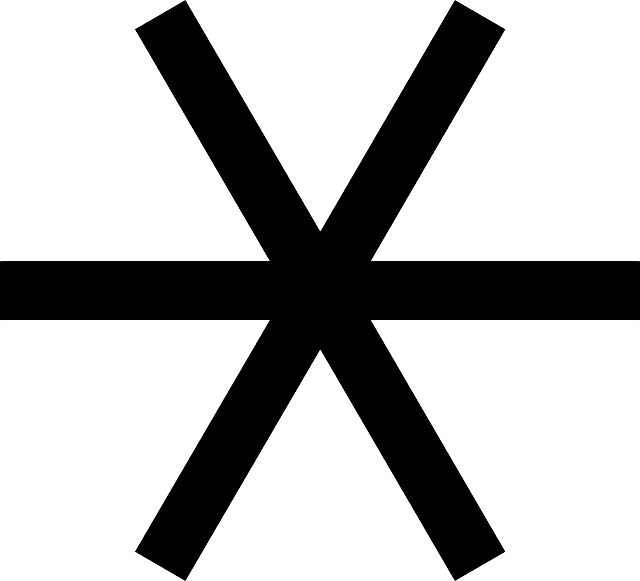
**Figure S4-5.
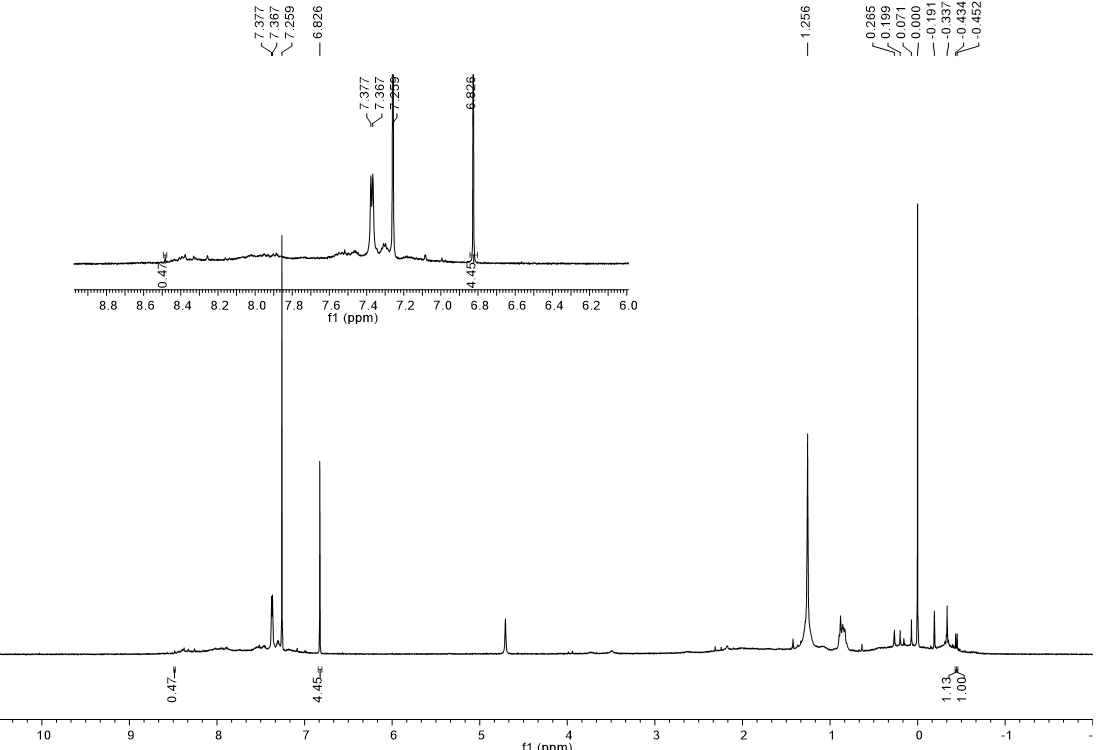
**^1^H NMR spectrum of crude products including oligomers with small amounts of cyclodimers **16a** and **16b** (400 MHz, CDCl_3_, rt). The asterisk indicates the signal due to internal standard bromoform.

**Figure S4-6.** ^1^H NMR spectrum of crude products including oligomers and cyclodimer **17a** (400 MHz, CDCl_3_, rt). The asterisk indicates the signal due to internal standard bromoform.


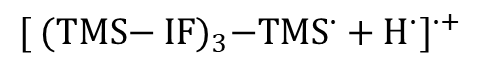

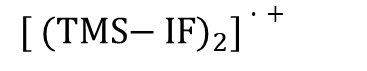

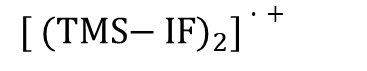

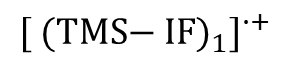

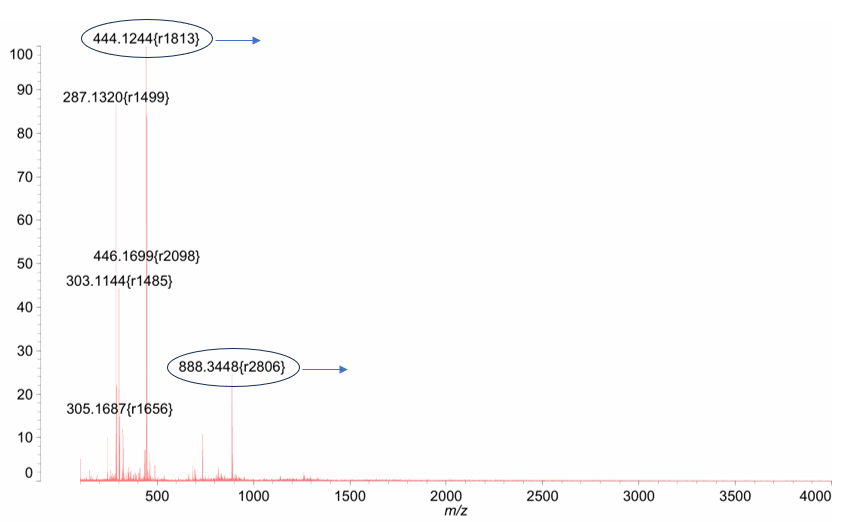

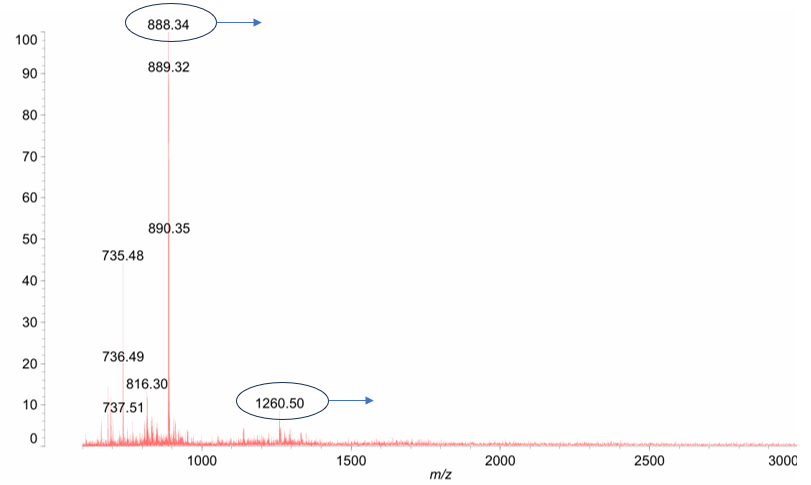
**5. MALDI-TOF Mass Spectra of Oligomers of Compound 4c**, **9c, 9d, and 9e**

[**Compound 14**]^•+^

[**Compound 14**]^•+^

[**Compound 4c**]^•+^

[(**Compound 4c)_3_** -TMS^•^ + H^•^]^•+^

**Figure S5-1.** MALDI-TOF mass spectra of oligomers of compound **4c**. Top: HR, bottom: LR.


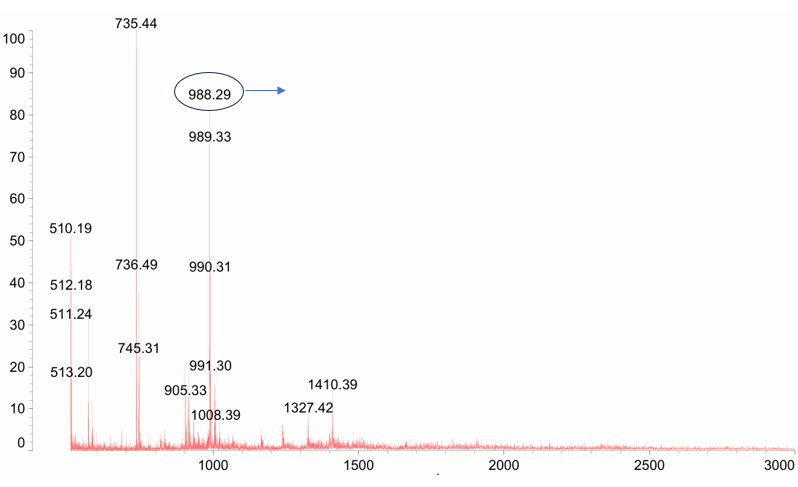

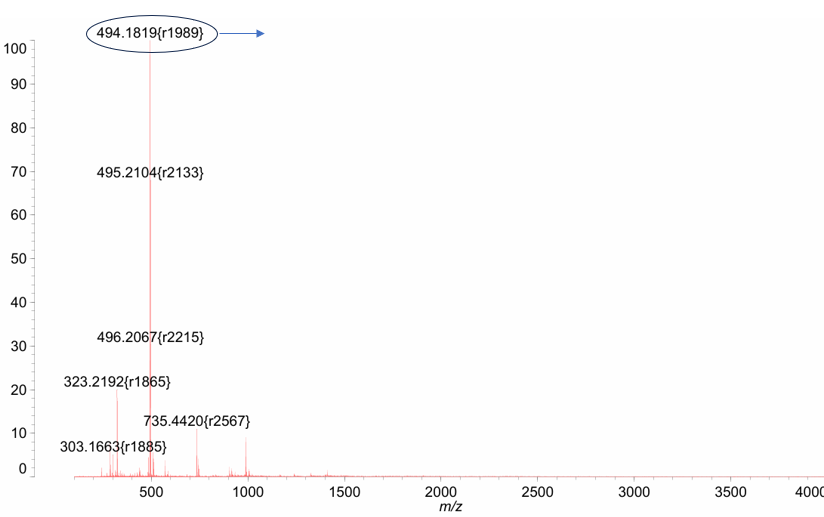
**Figure S5-2.** MALDI-TOF mass spectra of oligomers of compound **9c**. Top: HR, bottom: LR.

[**Compound 16**]^•+^

[**Compound 9c**]^•+^

[**Compound 9d**]^•+^

[**Compound 17** + **2H**^•^]^•+^


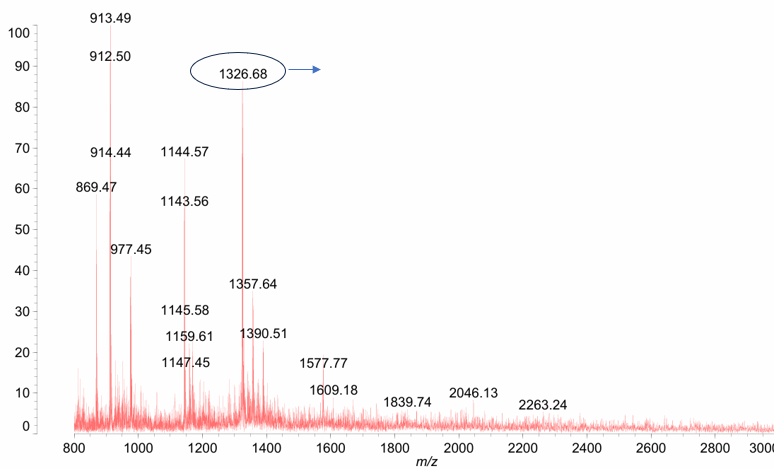

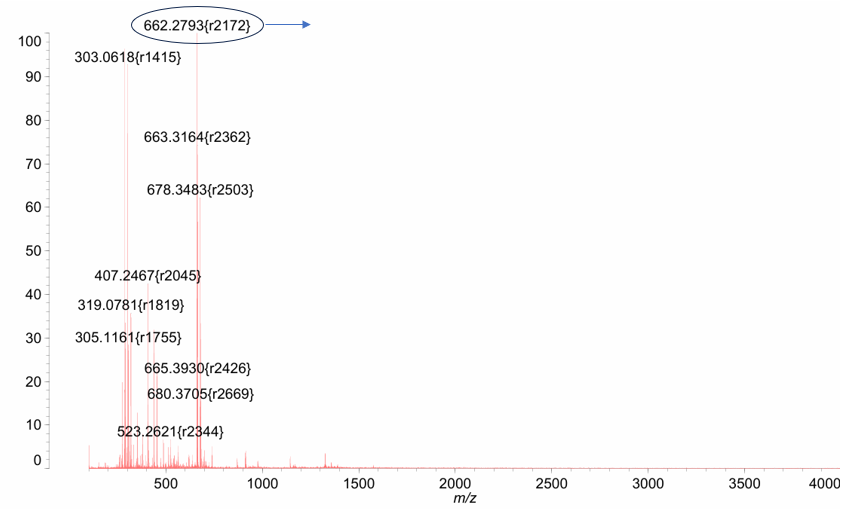
**Figure S5-3.** MALDI-TOF mass spectra of oligomers of compound **9d**. Top: HR, bottom: LR.


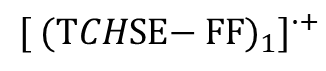
**Figure S5-4.** MALDI-TOF Mass spectra of oligomers of compound
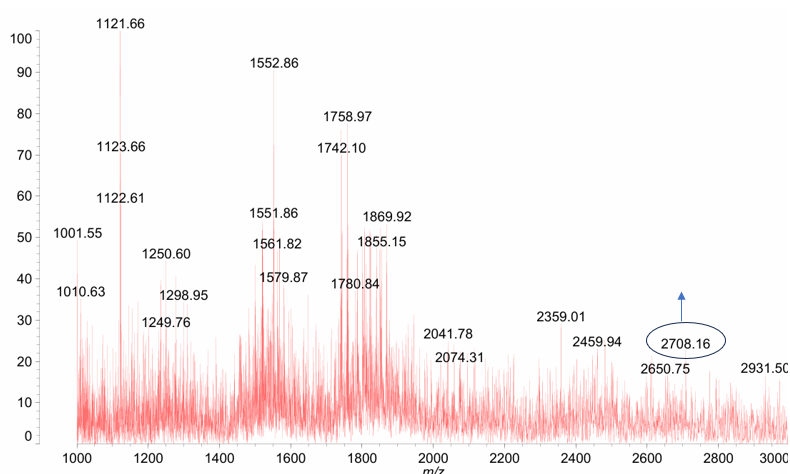

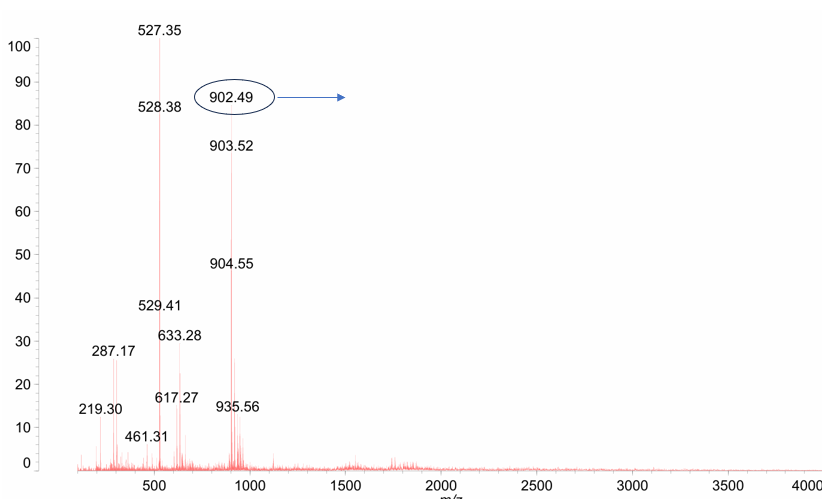
**9e**. Top: HR, bottom: LR.

[(**Compound 9e**)_3_]^•+^

[**Compound 9e**]^•+^

**6. X-Ray Cr**ystallographic Analyses of Cyclodi**mers**

For the X-ray crystallographic analysis, the data collections were performed on a Rigaku XtaLAB Synergy DW system and Bruker D8 Venture Dual X-ray Single Crystal at low temperatures with a monochromated Cu-Kα radiation (λ = 1.54184 Å). Data reduction and integration were performed with the software SAINT (v8.40A)^8^ and SADABS (v2008/1).^9^ The structure was solved by SHELXL (Sheldrick, 2014)^10^ using intrinsic phasing and refined by full-matrix least-squares procedures using SHELXL (Sheldrick, 2014)^10^ software package. All non-hydrogen atoms were refined anisotropically. Hydrogen atoms were placed in idealized positions and refined as rigid atoms with the relative isotropic displacement parameters.

**X-ray Crystallographic Structure of *H,T,T,T*-12a**

Crystals suitable for X-ray analysis were obtained by slow diffusion of acetonitrile to a dichloromethane solution of *H,T,T,T***-12a** under dark.

**Table S1.** Crystal Data for *H,T,T,T***-12a**.

| Compound | [(triisopropylsilyl)ethynyl]indeno[2,1-*b*]fluorene *H*,*T*,*T*,*T* dimer |
| --- | --- |
| Empirical formula | C_84.5_ H_105_ Si_4_ |
| Formula weight | 1268.49 |
| Temperature / *K* | 238(2) |
| Crystal system | Triclinic |
| Space group | *P*-1 |
| *a* / Å | 12.641(5) |
| *b* / Å | 17.707(7) |
| *c* / Å | 18.115(7) |
| *α* / ° | 110.463(9) |
| *β* / ° | 100.351(9) |
| *γ* / ° | 90.781(10) |
| *V* / Å^3^ | 3723(2) |
| *Z* | 2 |
| *ρ*_calc_ (g/cm^3^) | 1.132 |
| *μ* / mm^‑1^ | 0.159 |
| *F* (000) | 1370 |
| Crystal size / mm | 0.24 × 0.13 × 0.10 |
| 2*θ* range for data collection / ° | 2.045 to 25.095 |
| Index ranges | -15 ≤ h ≤ 15, -21 ≤k ≤ 21, -21≤ l ≤ 21 |
| Reflections collected | 26223 |
| Independent reflections | 13174 [*R*(int) = 0.0553] |
| Data/restraints/parameters | 13174 / 64 / 854 |
| Goodness-of-fit on *F*^2 a^ | 1.168 |
| Final *R* indexes [*I* >2*σ* (*I*)] ^b^ | *R*1 = 0.0822, *wR*2 = 0.1926 |
| Final *R* indexes [all data] ^b^ | *R*1 = 0.1257, *wR*2 = 0.2111 |
| Largest diff. peak/hole / e Å^-3^ | 1.674/ -1.054 |
| CCDC No. | 2451664 |

^a^ Goodness-of-fit = [Σ[*w*(*F*_o_^2^-*F*_c_^2^)^2^]/(*N*_obs_-*N*_params_)]^½^, based on all data.

^b^*R*1 = Σ||*F*_o_|-|*F*_c_||/Σ|*F*_o_|. *wR*2 = [Σ[*w*(*F*_o_^2^-*F*_c_^2^)^2^]/Σ[*w*(*F*_o_^2^)^2^]].


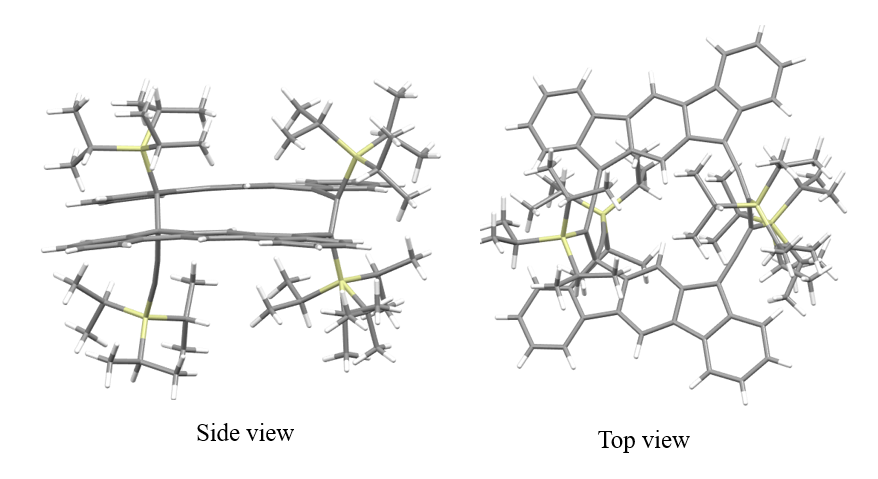


**Figure S6.** Crystal structure of *H,T,T,T***-12a**.

**X-ray Crystallographic Structure of *H,T,H,T*-12b**

Crystals suitable for X-ray analysis was obtained by slow diffusion of acetonitrile to a dichloromethane solution of *H,T,H,T***-12b** under dark.

**Table S2.** Crystal Data for *H,T,H,T***-12b**.

| Compound | [(triisopropylsilyl)ethynyl]indeno[2,1-*b*]fluorene *H*,*T*,*H*,*T* dimer |
| --- | --- |
| Empirical formula | C_84_H_104_Si_4_ |
| Formula weight | 1226.03 |
| Temperature / *K* | 100.00(10) |
| Crystal system | triclinic |
| Space group | *P*-1 |
| *a* / Å | 9.29211(13) |
| *b* / Å | 12.5707(3) |
| *c* / Å | 16.1891(4) |
| *α* / ° | 69.345(2) |
| *β* / ° | 88.8030(15) |
| *γ* / ° | 87.0870(15) |
| *V* / Å^3^ | 1767.19(7) |
| *Z* | 1 |
| *ρ_calc_* (*g*/cm^3^) | 1.152 |
| *μ* / mm^‑1^ | 1.103 |
| *F* (000) | 664.0 |
| Crystal size/mm^3^ | 0.05 × 0.04 × 0.04 |
| Radiation | Cu Kα (λ = 1.54184) |
| 2*θ* range for data collection / ° | 5.834 to 149.652 |
| Index ranges | -11 ≤ h ≤ 8, -15 ≤ k ≤ 15, -19 ≤ l ≤ 19 |
| Reflections collected | 25992 |
| Independent reflections | 6877 [*R*_int_ = 0.0260, *R*_sigma_ = 0.0274] |
| Data/restraints/parameters | 6877 / 0 / 410 |
| Goodness-of-fit on *F*^2 a^ | 1.051 |
| Final *R* indexes [*I* ≥ 2σ (*I*)]^b^ | *R*_1_ = 0.0347, *wR*_2_ = 0.0863 |
| Final *R* indexes [all data]^b^ | *R*_1_ = 0.0394, *wR*_2_ = 0.0889 |
| Largest diff. peak/hole / e Å^-3^ | 0.31/ -0.30 |
| CCDC No. | 2451683 |

^a^ Goodness-of-fit = [Σ[*w*(*F*_o_^2^-*F*_c_^2^)^2^]/(*N*_obs_-*N*_params_)]^½^, based on all data.

^b^*R*1 = Σ||*F*_o_|-|*F*_c_||/Σ|*F*_o_|. *wR*2 = [Σ[*w*(*F*_o_^2^-*F*_c_^2^)^2^]/Σ[*w*(*F*_o_^2^)^2^]].

**
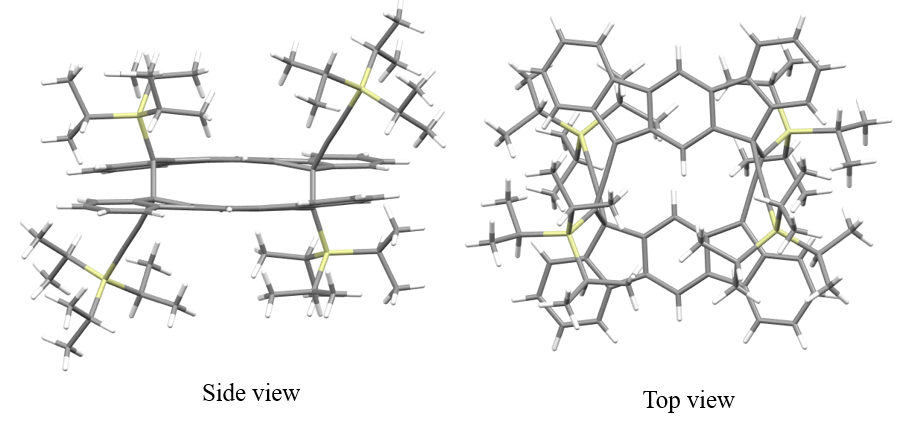
**

**Figure S7.** Crystal structures of *H,T,H,T***-12b**.

**X-ray Crystallographic Structure of *T,T,T,T*-13c**

Crystals suitable for X-ray analysis was obtained by slow evaporation of toluene solution of *T,T,T,T***-13c** under dark.

**Table S3.** Crystal Data for *T,T,T,T***-13c**.

| Compound | [(triisopropylsilyl)ethynyl]-2,8-di-*tert*-butyl indeno[2,1-*b*]fluorene *T,T,T,T* dimer |
| --- | --- |
| Empirical formula | C_100_H_136_Si_4_ |
| Formula weight | 1450.44 |
| Temperature / *K* | 115(20) |
| Crystal system | triclinic |
| Space group | *P*-1 |
| *a* / Å | 12.5027(2) |
| *b* / Å | 16.2353(3) |
| *c* / Å | 22.1077(3) |
| *α* / ° | 87.2710(10) |
| *β* / ° | 83.6070(10) |
| *γ* / ° | 78.6930(10) |
| *V* / Å^3^ | 4371.54(12) |
| *Z* | 2 |
| *ρ*_calc_ (g/cm^3^) | 1.102 |
| *μ* / mm^‑1^ | 0.959 |
| *F* (000) | 1584.0 |
| Crystal size/mm^3^ | 0.06 × 0.04 × 0.03 |
| Radiation | Cu Kα (λ = 1.54184) |
| 2*θ* range for data collection / ° | 6.772 to 134.16 |
| Index ranges | -14 ≤ h ≤ 10, -19 ≤ k ≤ 19, -26 ≤ l ≤ 26 |
| Reflections collected | 56512 |
| Independent reflections | 15439 [*R*_int_ = 0.0859, *R*_sigma_ = 0.0729] |
| Data/restraints/parameters | 15439 / 39 / 1004 |
| Goodness-of-fit on *F*^2 a^ | 1.074 |
| Final *R* indexes [*I* ≥ 2σ (*I*)]^b^ | *R*_1_ = 0.0696, *wR*_2_ = 0.2003 |
| Final *R* indexes [all data]^b^ | *R*_1_ = 0.1166, *wR*_2_ = 0.2854 |
| Largest diff. peak/hole / e Å^-3^ | 1.24/ -0.76 |
| CCDC No. | 2451681 |

^a^ Goodness-of-fit = [Σ[*w*(*F*_o_^2^-*F*_c_^2^)^2^]/(*N*_obs_-*N*_params_)]^½^, based on all data.

^b^*R*1 = Σ||*F*_o_|-|*F*_c_||/Σ|*F*_o_|. *wR*2 = [Σ[*w*(*F*_o_^2^-*F*_c_^2^)^2^]/Σ[*w*(*F*_o_^2^)^2^]].

**
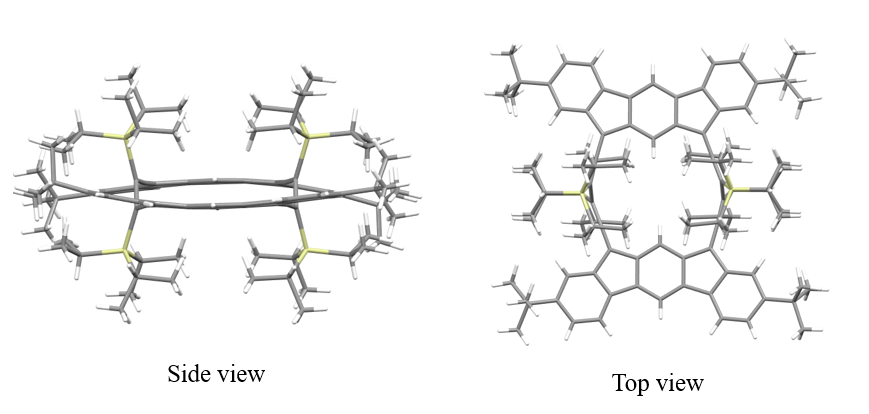
**

**Figure S8a.** Crystal structures of *T,T,T,T***-13c** (Molecule A).

**
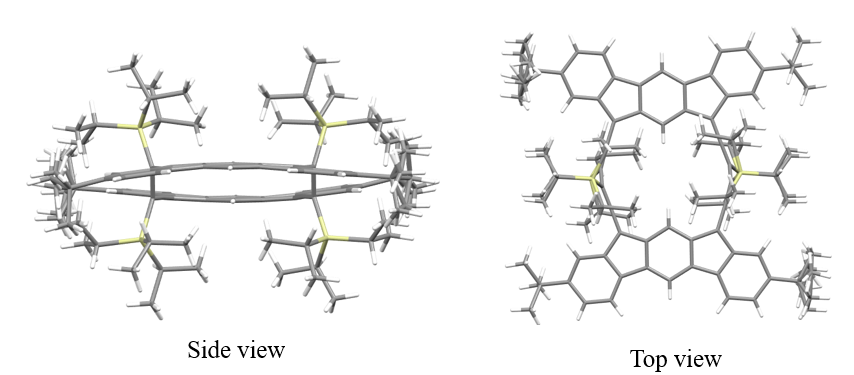
**

**Figure S8b.** Crystal structures of *T,T,T,T***-13c** (Molecule B) in which TIPS groups are disordered.

**X-ray Crystallographic Structure of *H,T,T,T*-15a**

Crystals suitable for X-ray analysis was obtained by the solvent diffusion method in chloroform and acetonitrile under dark.

**Table S4.** Crystal Data for *H,T,T,T***-15a**

| Compound | [(tricyclohexylsilyl)ethynyl]indeno[2,1-*b*]fluorene *H*,*T*,*T*,*T* dimer |
| --- | --- |
| Empirical formula | C_120_ H_152_ Si_4_ |
| Formula weight | 1706.77 |
| Temperature / *K* | 150(2) |
| Crystal system | triclinic |
| Space group | *P*-1 |
| *a* / Å | 12.2355(4) |
| *b* / Å | 17.0783(5) |
| *c* / Å | 24.9873(7) |
| *α* / ° | 100.5430(17) |
| *β* / ° | 90.5890(17) |
| *γ* / ° | 100.5531(18) |
| *V* / Å^3^ | 5041.2(3) |
| *Z* | 2 |
| *ρ_calc_* (*g*/cm^3^) | 1.124 |
| *μ* / mm^‑1^ | 0.903 |
| *F* (000) | 1856 |
| Crystal size/mm^3^ | 0.135 ×0.090 × 0.047 |
| 2*θ* range for data collection / ° | 1.800 to 68.460 |
| Index ranges | -14 ≤ h ≤ 14, -20 ≤ k ≤ 19, -30 ≤ l ≤ 29 |
| Reflections collected | 34569 |
| Independent reflections | 18390 [R(int) = 0.0474] |
| Data/restraints/parameters | 18390 / 79 / 1153 |
| Goodness-of-fit on *F*^2 a^ | 1.024 |
| Final *R* indexes [*I* ≥ 2σ (*I*)]^b^ | *R*_1_ = 0.0591, *wR*_2_ = 0.1449 |
| Final *R* indexes [all data]^b^ | *R*_1_ = 0.0952, *wR*_2_ = 0.1687 |
| Largest diff. peak/hole / e Å^-3^ | 0.486/ -0.410 |
| CCDC No. | 2451658 |

^a^ Goodness-of-fit = [Σ[*w*(*F*_o_^2^-*F*_c_^2^)^2^]/(*N*_obs_-*N*_params_)]^½^, based on all data.

^b^*R*1 = Σ||*F*_o_|-|*F*_c_||/Σ|*F*_o_|. *wR*2 = [Σ[*w*(*F*_o_^2^-*F*_c_^2^)^2^]/Σ[*w*(*F*_o_^2^)^2^]].**_
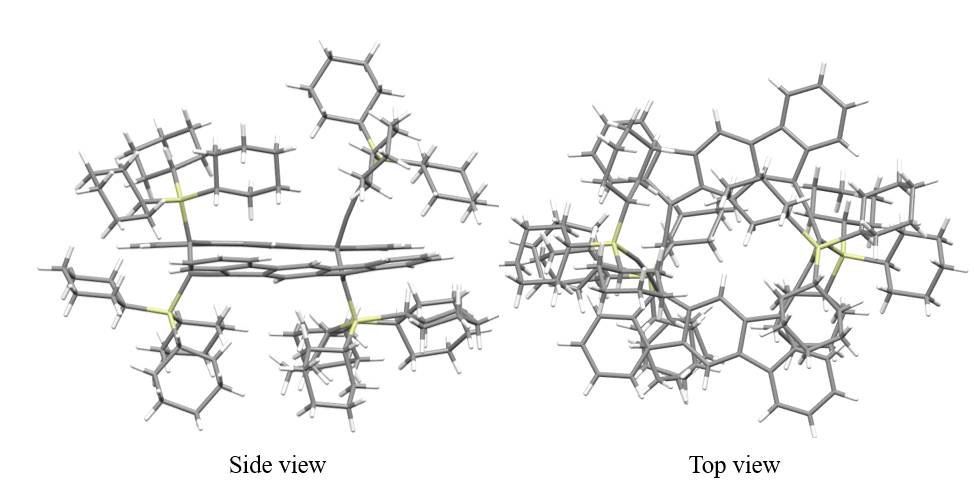
_**

**Figure S9.** Crystal structures of *H,T,T,T***-15a**

**X-ray Crystallographic Structure of *H,T,T,T*-17a**

Crystals suitable for X-ray analysis was obtained by the solvent diffusion method in dichloromethane and acetonitrile of under dark.

**Table S5.** Crystal Data for *H,T,T,T***-17a**

| Compound | [(triisopropylsilyl)ethynyl]fluoereno[2,3-*b*]fluorene *H,T,T,T* dimer |
| --- | --- |
| Empirical formula | C_92.75_ H_109.50_ Cl_1.50_ Si_4_ |
| Formula weight | 1389.83 |
| Temperature / *K* | 100(2) |
| Crystal system | triclinic |
| Space group | *P*-1 |
| *a* / Å | 18.0335(10) |
| *b* / Å | 20.5118(12) |
| *c* / Å | 23.8122(13) |
| *α* / ° | 82.228(3) |
| *β* / ° | 70.244(3) |
| *γ* / ° | 77.764(3) |
| *V* / Å^3^ | 8082.0(8) |
| *Z* | 4 |
| *ρ*_calc_ (g/cm^3^) | 1.142 |
| *μ* / mm^‑1^ | 0.168 |
| *F* (000) | 2990 |
| Crystal size / mm^3^ | 0.18 × 0.05 × 0.05 |
| 2*θ* range for data collection / ° | 0.911 to 25.000 |
| Index ranges | -21 ≤ h ≤ 21, -24 ≤ k ≤ 24, -28 ≤ l ≤ 28 |
| Reflections collected | 124116 |
| Independent reflections | 28332 [*R*(int) = 0.0873] |
| Data / restraints / parameters | 28332 / 635 / 1930 |
| Goodness-of-fit on *F*^2 a^ | 1.047 |
| Final *R* indexes [*I* >2*σ* (*I*)] ^b^ | *R*_1_ = 0.1375, *wR*_2_ = 0.3456 |
| Final *R* indexes [all data] ^b^ | *R*_1_ = 0.2105, *wR*_2_ = 0.3970 |
| Largest diff. peak/hole / e Å^-3^ | 1.338/ -0.949 |
| CCDC No. | 2451665 |

^a^ Goodness-of-fit = [Σ[*w*(*F*_o_^2^-*F*_c_^2^)^2^]/(*N*_obs_-*N*_params_)]^½^, based on all data.

^b^*R*1 = Σ||*F*_o_|-|*F*_c_||/Σ|*F*_o_|. *wR*2 = [Σ[*w*(*F*_o_^2^-*F*_c_^2^)^2^]/Σ[*w*(*F*_o_^2^)^2^]].


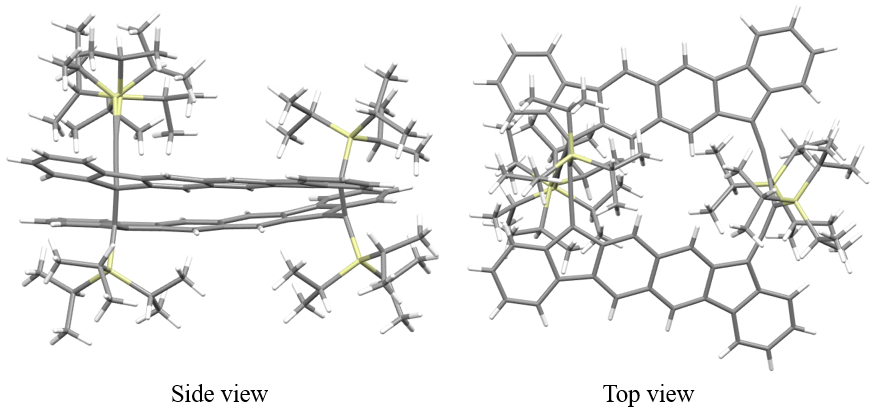


**Figure S10.** Crystal structure of *H,T,T,T***-17a**

**X-ray Crystallographic Structure of *H,T,H,T*-17b**

Crystals suitable for X-ray analysis was obtained by the solvent diffusion method in THF and acetonitrile under dark.

**Table S6.** Crystal Data for *H,T,H,T***-17b**

| Compound | [(triisopropylsilyl)ethynyl]fluoereno[2,3-*b*]fluorene *H,T,H,T* dimer |
| --- | --- |
| Empirical formula | C_100_H_136_Si_4_ |
| Formula weight | 1450.44 |
| Temperature / *K* | 115(20) |
| Crystal system | triclinic |
| Space group | *P*-1 |
| *a* / Å | 12.5027(2) |
| *b* / Å | 16.2353(3) |
| *c* / Å | 22.1077(3) |
| *α* / ° | 87.2710(10) |
| *β* / ° | 83.6070(10) |
| *γ* / ° | 78.6930(10) |
| *V* / Å^3^ | 4371.54(12) |
| *Z* | 2 |
| *ρ*_calc_ (g/cm^3^) | 1.102 |
| *μ* / mm^‑1^ | 0.959 |
| *F* (000) | 1584.0 |
| Crystal size / mm^3^ | 0.06 × 0.04 × 0.03 |
| Radiation | Cu Kα (λ = 1.54184) |
| 2*θ* range for data collection / ° | 6.772 to 134.16 |
| Index ranges | -14 ≤ h ≤ 10, -19 ≤ k ≤ 19, -26 ≤ l ≤ 26 |
| Reflections collected | 56512 |
| Independent reflections | 15439 [*R*_int_ = 0.0859, *R*_sigma_ = 0.0729] |
| Data / restraints / parameters | 15439 / 39 / 1004 |
| Goodness-of-fit on *F*^2 a^ | 1.074 |
| Final *R* indexes [*I* >2*σ* (*I*)] ^b^ | *R*_1_ = 0.0696, *wR*_2_ = 0.2003 |
| Final *R* indexes [all data] ^b^ | *R*_1_ = 0.1166, *wR*_2_ = 0.2854 |
| Largest diff. peak/hole / e Å^-3^ | 1.24/ -0.76 |
| CCDC No. | 2449648 |

^a^ Goodness-of-fit = [Σ[*w*(*F*_o_^2^-*F*_c_^2^)^2^]/(*N*_obs_-*N*_params_)]^½^, based on all data.

^b^*R*1 = Σ||*F*_o_|-|*F*_c_||/Σ|*F*_o_|. *wR*2 = [Σ[*w*(*F*_o_^2^-*F*_c_^2^)^2^]/Σ[*w*(*F*_o_^2^)^2^]].


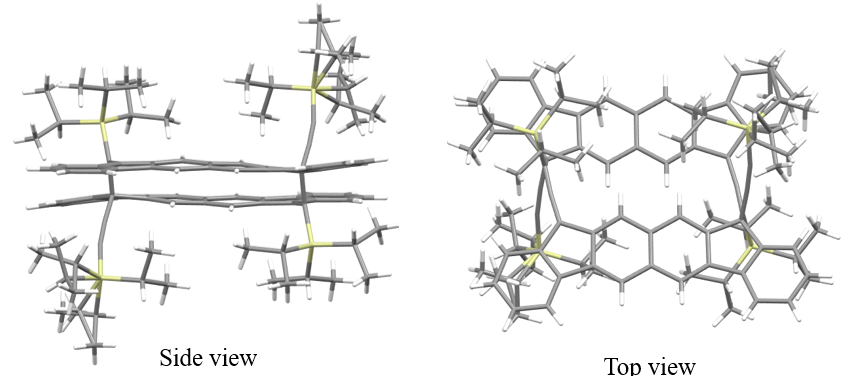


**Figure S11.** Crystal structures of *H,T,H,T***-17b**.

**X-ray Crystallographic Structure of *H,T,T,T*-18a**

Crystals suitable for X-ray analysis was obtained by the solvent diffusion method in chloroform and acetonitrile under dark.

**Table S7.** Crystal Data for *H,T,T,T***-18a**

| Compound | [(tricyclohexylsilyl)ethynyl]fluoereno[2,3-*b*]fluorene *H,T,T,T* dimer |
| --- | --- |
| Empirical formula | C_129.50_ H_157.50_ Cl_4.50_ Si_4_ |
| Formula weight | 1985.93 |
| Temperature / *K* | 150(2) |
| Crystal system | triclinic |
| Space group | *P*-1 |
| *a* / Å | 16.6769(6) |
| *b* / Å | 19.5977(7) |
| *c* / Å | 19.7408(7) |
| *α* / ° | 69.2259(13) |
| *β* / ° | 80.0362(15) |
| *γ* / ° | 69.5463(14) |
| *V* / Å^3^ | 5643.2(4) |
| *Z* | 2 |
| *ρ*_calc_ (g/cm^3^) | 1.169 |
| *μ* / mm^‑1^ | 0.208 |
| *F* (000) | 2134.0 |
| Crystal size / mm^3^ | 0.421 × 0.277 × 0.124 |
| 2*θ* range for data collection / ° | 1.848 to 25.250 |
| Index ranges | -19 ≤ h ≤ 20, 23 ≤ k ≤ 23, -23 ≤ l ≤ 23 |
| Reflections collected | 36023 |
| Independent reflections | 20409 [R(int) = 0.0457] |
| Data / restraints / parameters | 20409 / 130 / 1330 |
| Goodness-of-fit on *F*^2 a^ | 1.023 |
| Final *R* indexes [*I* >2*σ* (*I*)] ^b^ | *R*_1_ = 0.0787, *wR*_2_ = 0.1965 |
| Final *R* indexes [all data] ^b^ | *R*_1_ = 0.1104, *wR*_2_ = 0.2250 |
| Largest diff. peak/hole / e Å^-3^ | 1.411/ -1.189 |
| CCDC No. | 2451663 |

^a^ Goodness-of-fit = [Σ[*w*(*F*_o_^2^-*F*_c_^2^)^2^]/(*N*_obs_-*N*_params_)]^½^, based on all data.

^b^*R*1 = Σ||*F*_o_|-|*F*_c_||/Σ|*F*_o_|. *wR*2 = [Σ[*w*(*F*_o_^2^-*F*_c_^2^)^2^]/Σ[*w*(*F*_o_^2^)^2^]].


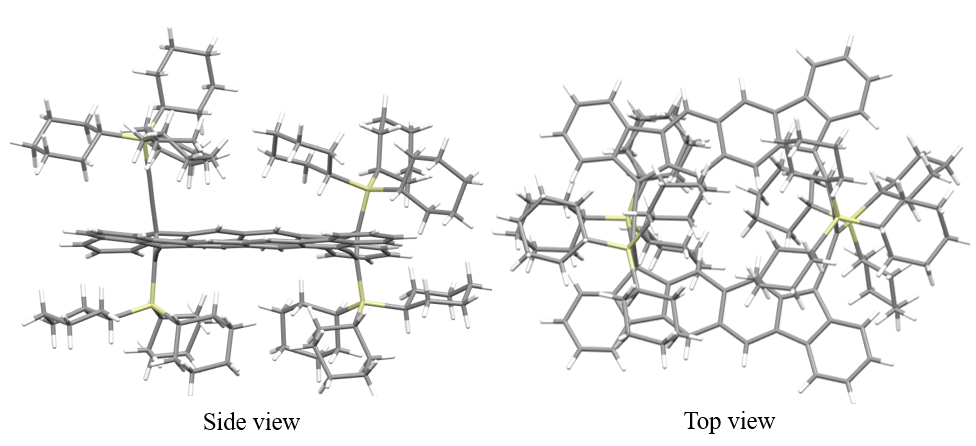


**Figure S12.** Crystal structures of *H,T,T,T***-18a**

**
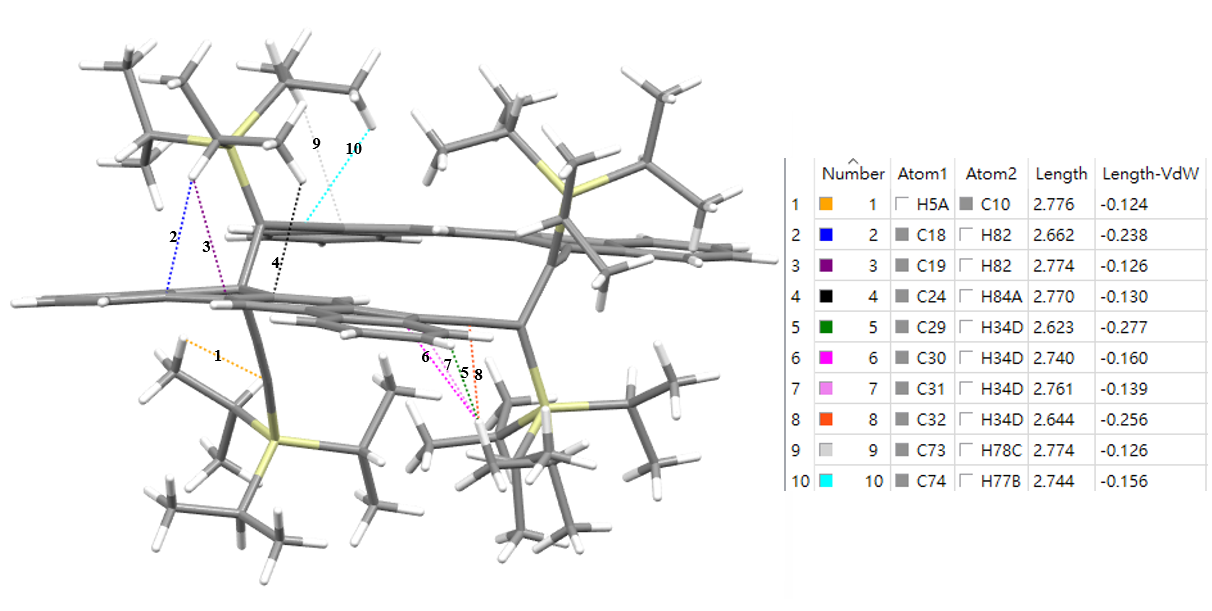
7. Short Intramolecular Contacts in Crystal Structures of Cyclodimers**

**Figure S13-1. S**hort intramolecular contacts in crystal structure of *H,T,T,T***-12a**.


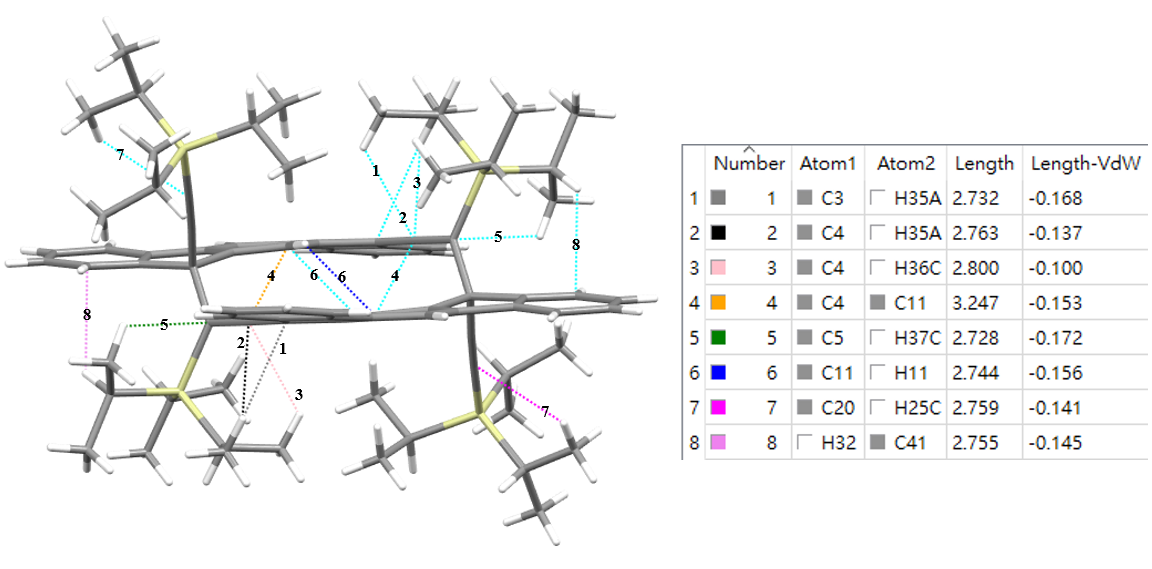


**Figure S13-2.** Short intramolecular contacts in crystal structure of *H,T,H,T***-12b**.

**Figure S13-3a.** Short intramolecular contacts in crystal structure of *T,T,T,T***-13c**
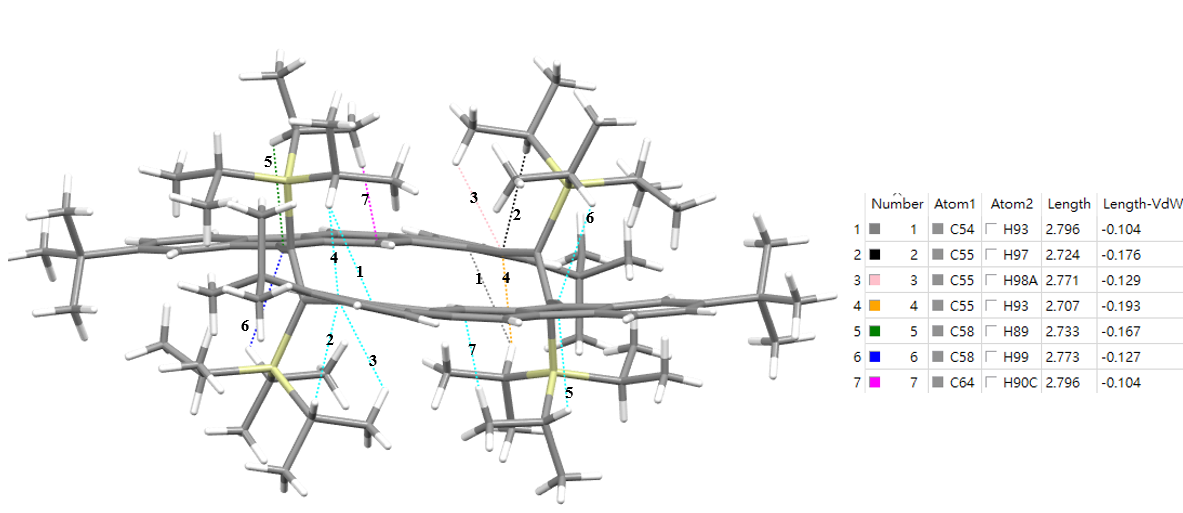
 (Molecule A).


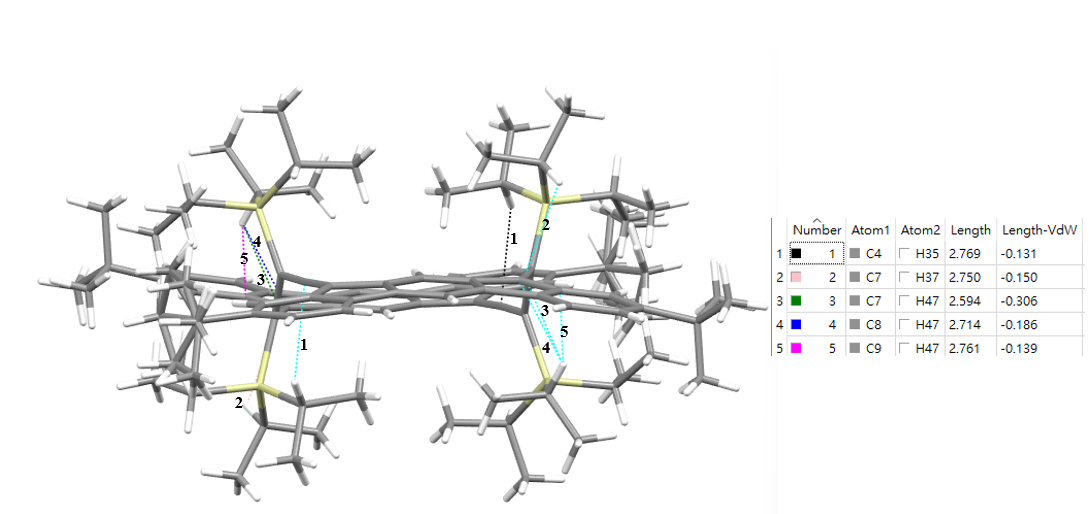


**Figure S13-3b.** Short intramolecular contacts in crystal structure of *T,T,T,T***-13c** (Molecule B).


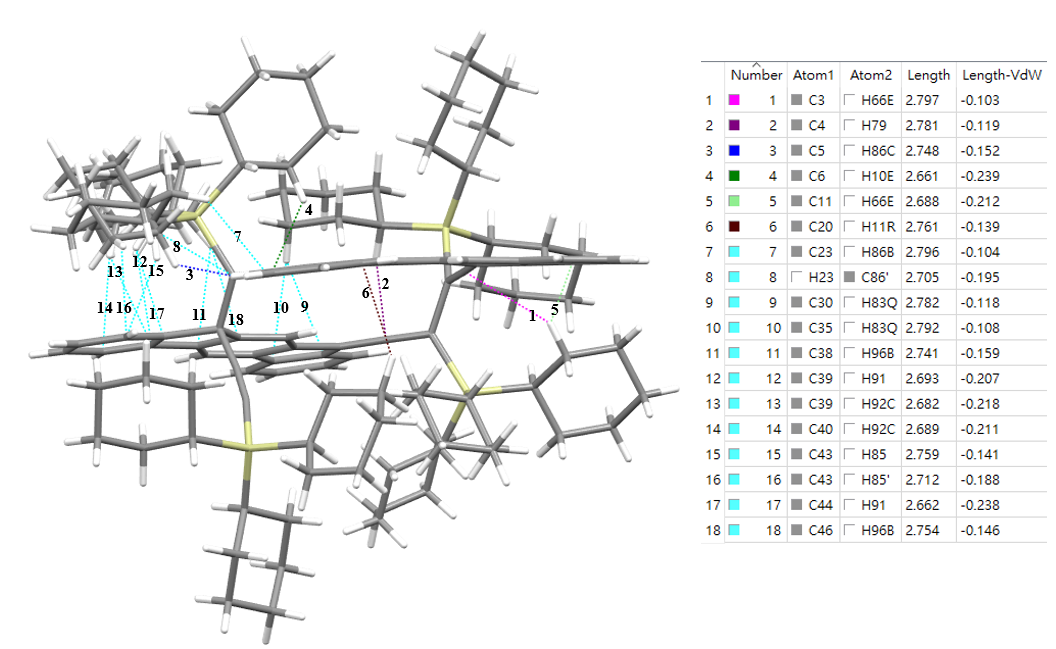


**Figure S13-4a. S**hort intramolecular contacts in crystal structure of *H,T,T,T***-15a**.


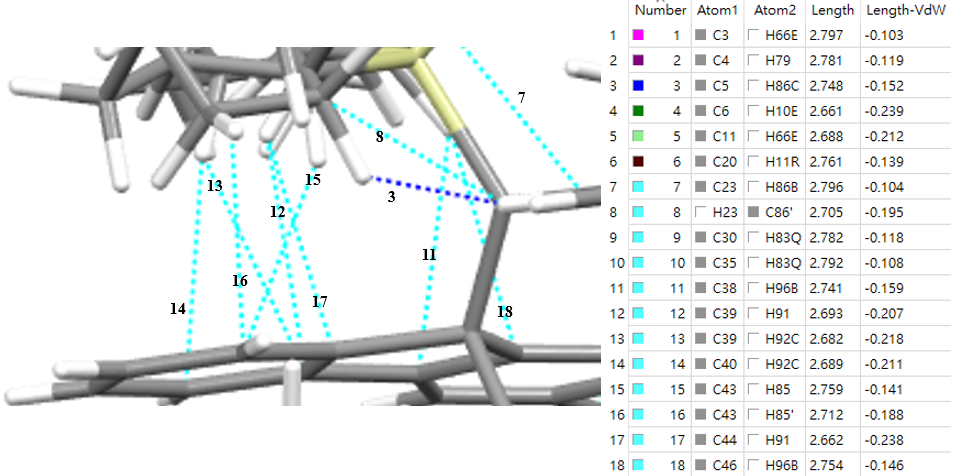


**Figure S13-4b. S**hort intramolecular contacts of disorder region in crystal structure of *H,T,T,T***-15a**.


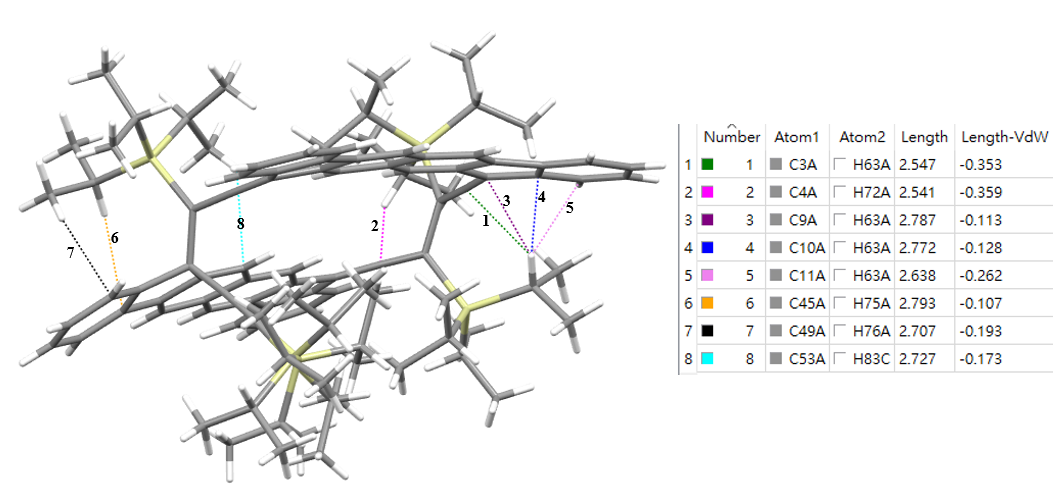


**Figure S13-5. S**hort intramolecular contacts in crystal structure of *H,T,T,T***-17a**.


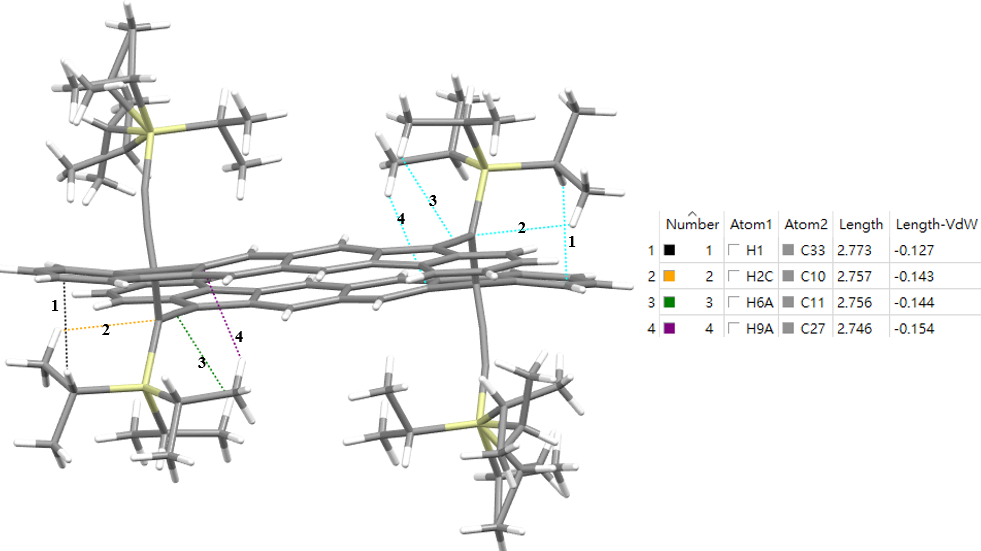


**Figure S13-6. S**hort intramolecular contacts in crystal structure of *H,T,H,T***-17b** .


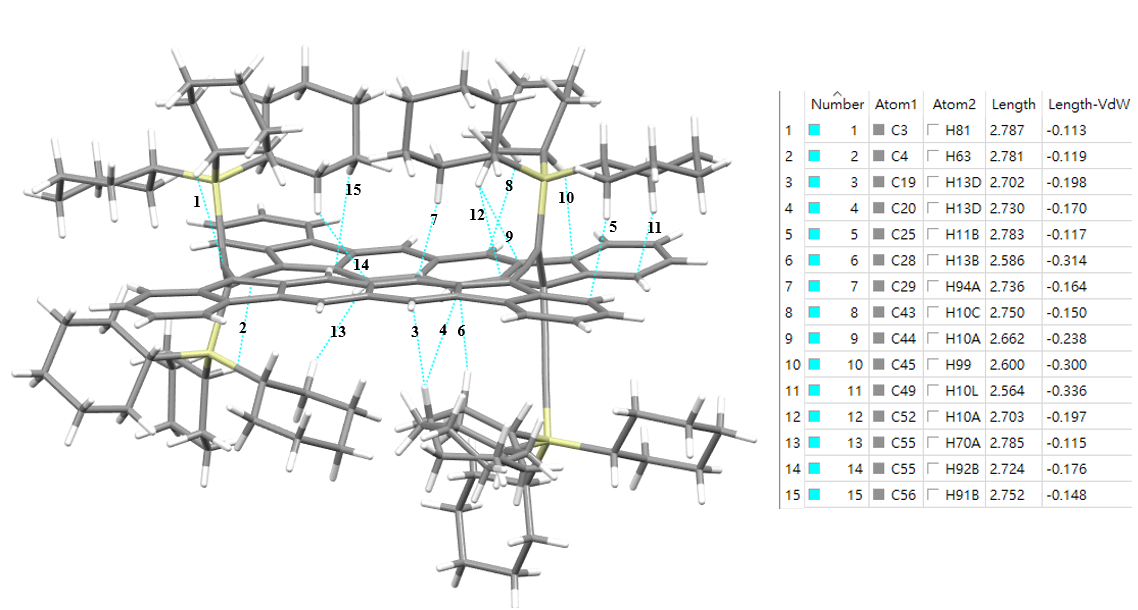


**Figure S13-7. S**hort intramolecular contacts in crystal structure of *H,T,T,T***-18a**.

**8. Theoretical Geometries of Cyclodimers**

**Table S8.** Theoretical nonbonded Si∙∙∙Si Distances in *H,T,T,T* and *H,T,H,T* Type Cyclodimers.^a^

| cyclodimer | C(sp^3^)Si∙∙∙SiC(sp^2^), Å | C(sp^2^)Si∙∙∙SiC(sp^2^), Å |
| --- | --- | --- |
| *H,T,T,T***-14a** | 6.65^a^ | 6.47^a^ |
| *H,T,H,T***-14b** | 6.37^a^ | – |
| *H,T,T,T***-12a** | 7.55^a^ (7.555)^b^ | 7.94^a^ (7.971)^b^ |
| *H,T,H,T***-12b** | 8.04^a^ (8.324)^b^ | – |
| *H,T,T,T***-15a** | 7.41^a^ (8.443)^b^ | 7.88^a^ (8.126)^b^ |
| *H,T,H,T***-15b** | 8.11^a^ | – |
| *H,T,T,T***-16a** | 8.45^a^ | 8.72^a^ |
| *H,T,H,T***-16b** | 8.79^a^ | – |
| *H,T,T,T***-17a** | 8.97^a^ (9.227)^b^ | 9.45^a^ (9.641)^b^ |
| *H,T,H,T***-17b** | 9.02 (9.037)^b^ | – |
| *H,T,T,T***-18a** | 8.99^a^ (9.761)^b^ | 9.02^a^  (9.029)^b^ |
| *H,T,H,T***-18b** | 10.25^a^ | – |

^a^ Calculated by the B3LYP-D3(BJ)/6-311G(d,p) level. ^b^ The Si∙∙∙Si distance in the structure of X-ray analysis is included in parentheses.

As the measure of steric compression, the intramolecular distances between the silicon atoms belonging to the same π conjugated framework are compared (Table S8). In the cyclodimers **12a**,**b** and **15a**,**b**, the Si∙∙∙Si distances are elongated compared to those of **14a**,**b** to avoid steric repulsion between the bulky alkyl groups. The same trend is observed in the theoretical structures of the indenofluorene monomers (Table 1) discussed above. However, whereas the Si∙∙∙Si distances of the fluorenofluorene monomers with bulky substituents (**9d** and **9e**) are shorter than that of **9a**, in the cyclodimers, the Si∙∙∙Si distances of the former dimers (**17a**,**b** and **18a**,**b**) are increased compared to that of **16a**.**b**. We attribute this to the small space defined by the dimer skeleton is not enough to fit the bulky (trialkyl)silyl groups in due to confinement by forming the new C-C bonds.

**9. NCI Plots for Cyclodimers**

The noncovalent interaction (NCI) analysis^11^ was done based on the results of the DFT calculations at the #B3LYP EmpiricalDispersion=GD3BJ/6-311g(d,p) opt level of theory using the Multiwfn program (ver. 3.8).^12^ The three-dimensional plots are shown in **Figure** **S14**. The regions showing van der Waals interactions are represented by green-colored isosurface, and the regions showing attractive and repulsive forces are displayed as blue- and red-colored isosurfaces (s = 0.5 au), respectively. The visualization was performed using a VMD program (ver. 1.9.3).^13^ The plots of the reduced density gradient versus the electron density multiplied by the sign of the second Hessian eigenvalue sign(λ_2_)ρ are prepared using Gnuplot 5.4.^14^ and are shown in **Figure S15**.


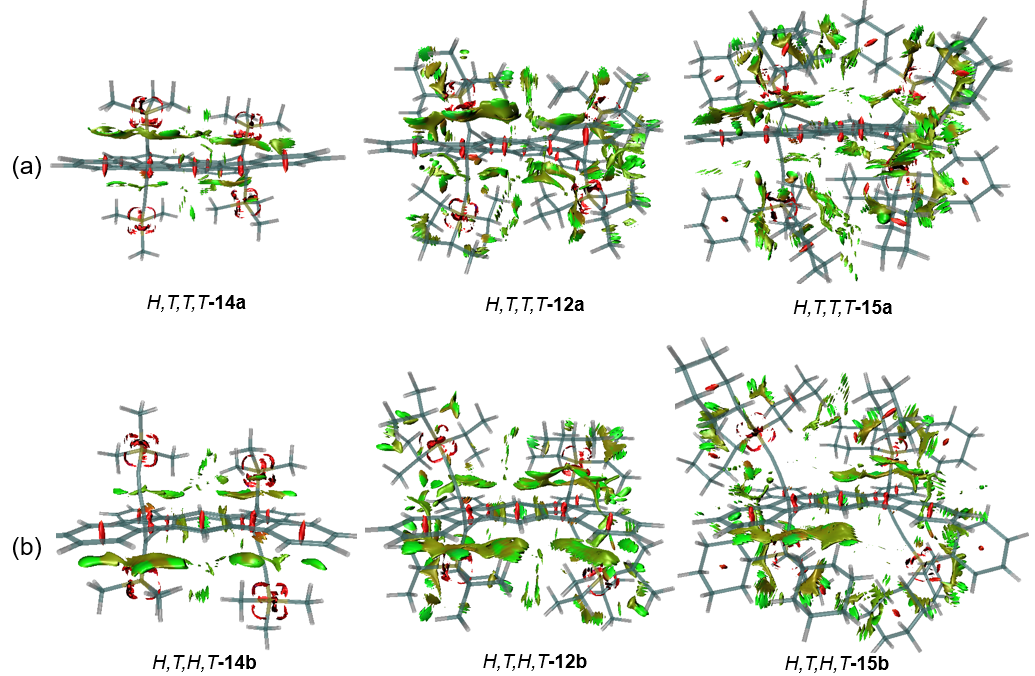


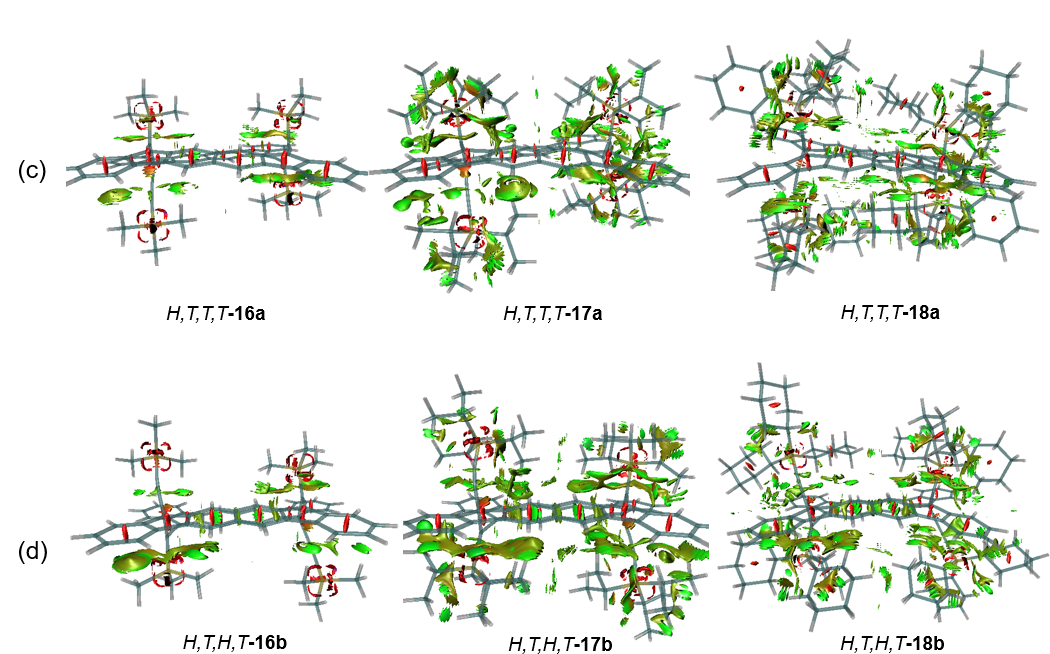


**Figure S14.** The three-dimensional plots of dimers. (a) *H,T,T,T* type indenofluorene dimers, left: TMS, middle: TIPS, right: TCHS. (b) *H,T,H,T* type indenofluorene dimers, left: TMS, middle: TIPS, right: TCHS. (c) *H,T,T,T* type fluorenofluorene dimers, left: TMS, middle: TIPS, right: TCHS. (d) *H,T,H,T* type fluorenofluorene dimers, left: TMS, middle: TIPS, right: TCHS.


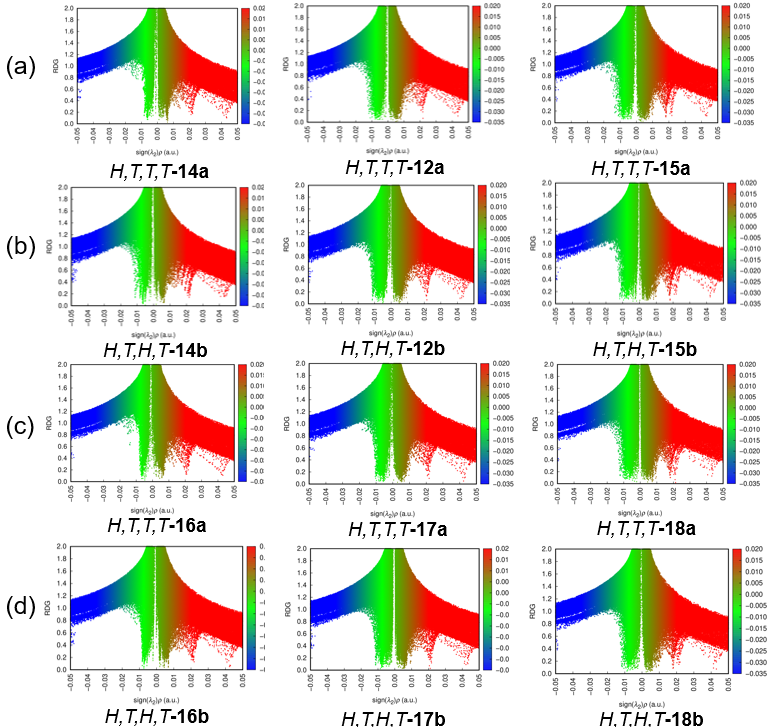


**Figure S15.** The plots of the reduced density gradient versus the electron density multiplied by the sign of the second Hessian eigenvalue sign(λ_2_)ρ of dimers. (a) *H,T,T,T* type indenofluorene dimers, left: TMS, middle: TIPS, right: TCHS. (b) *H,T,H,T* type indenofluorene dimers, left: TMS, middle: TIPS, right: TCHS. (c) *H,T,T,T* type fluorenofluorene dimers, left: TMS, middle: TIPS, right: TCHS. (d) *H,T,H,T* type fluorenofluorene dimers, left: TMS, middle: TIPS, right: TCHS.


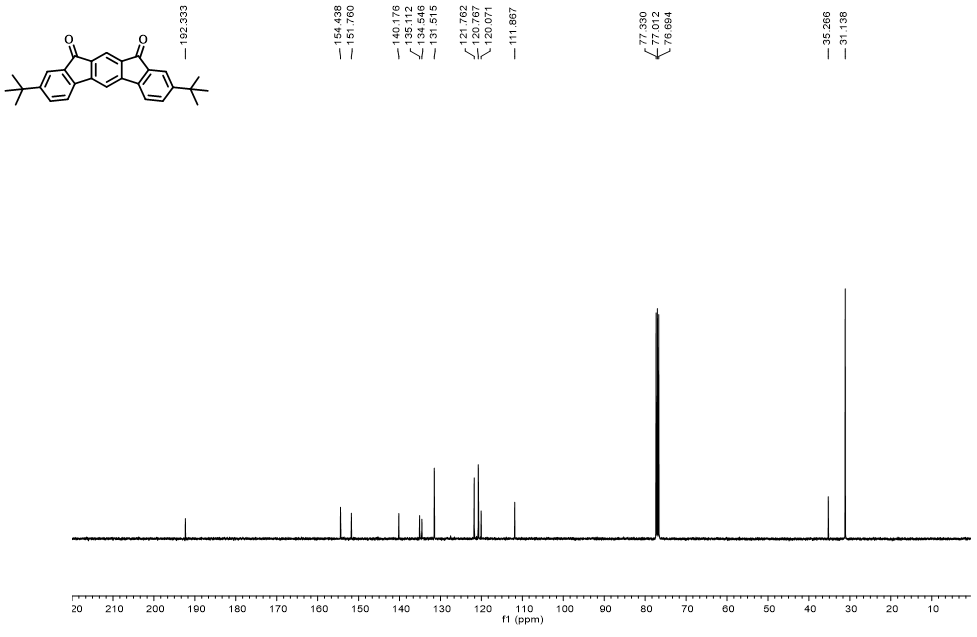

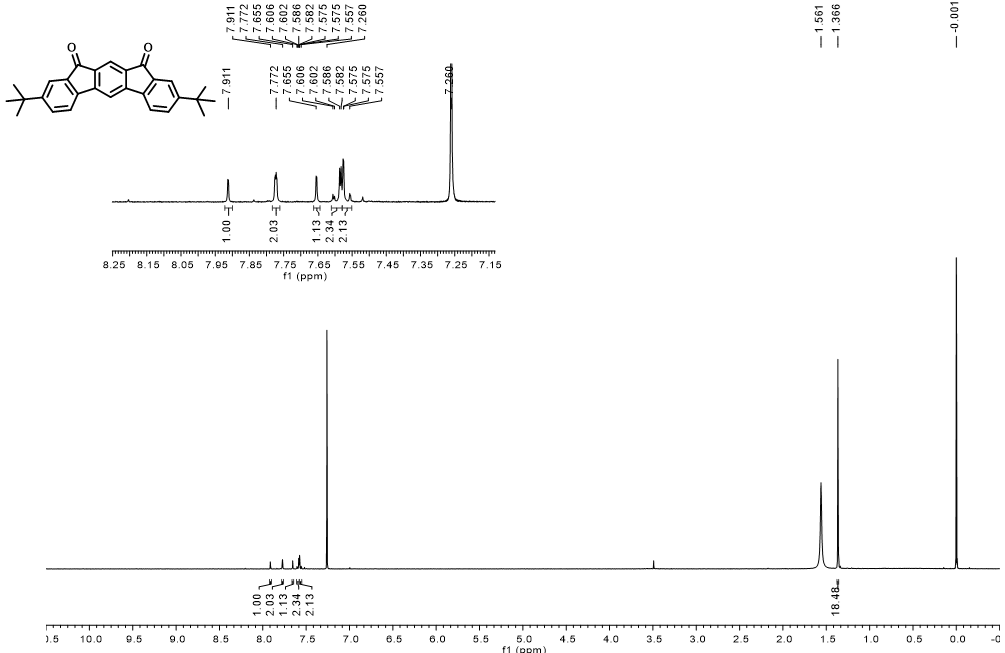
**10. NMR Spectra of New Compounds**

**Figure S16-1.** ^1^H NMR spectrum (400 MHz, CDCl_3_, rt) and ^13^C NMR spectrum (100 MHz, CDCl_3_, rt) of di-*t*-Bu-indenofluorenedione.

**
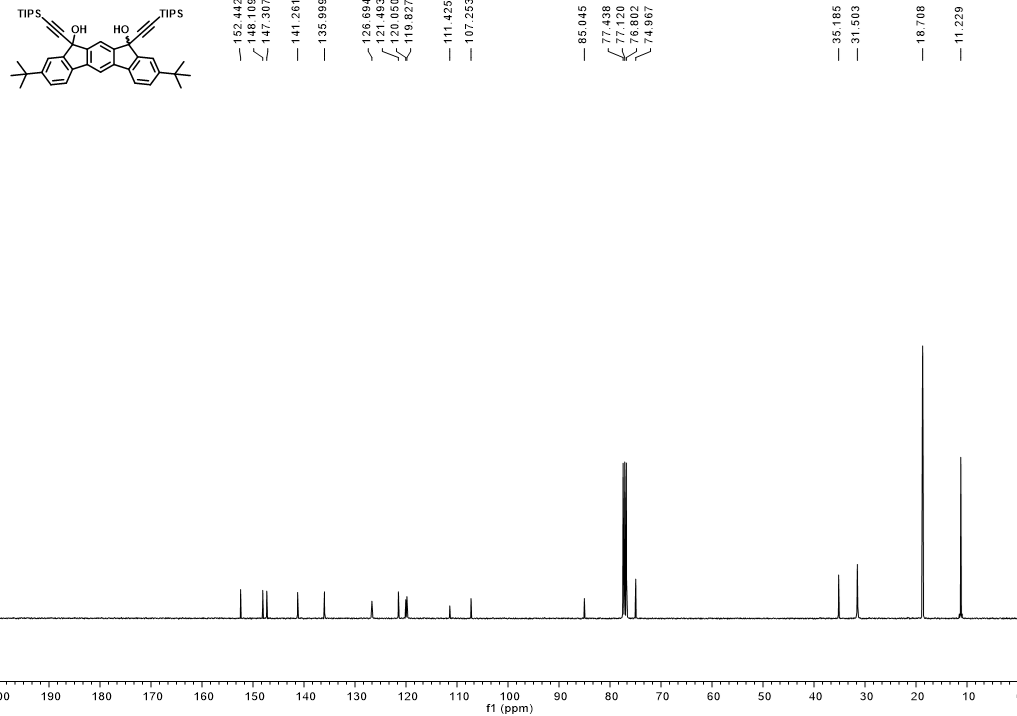

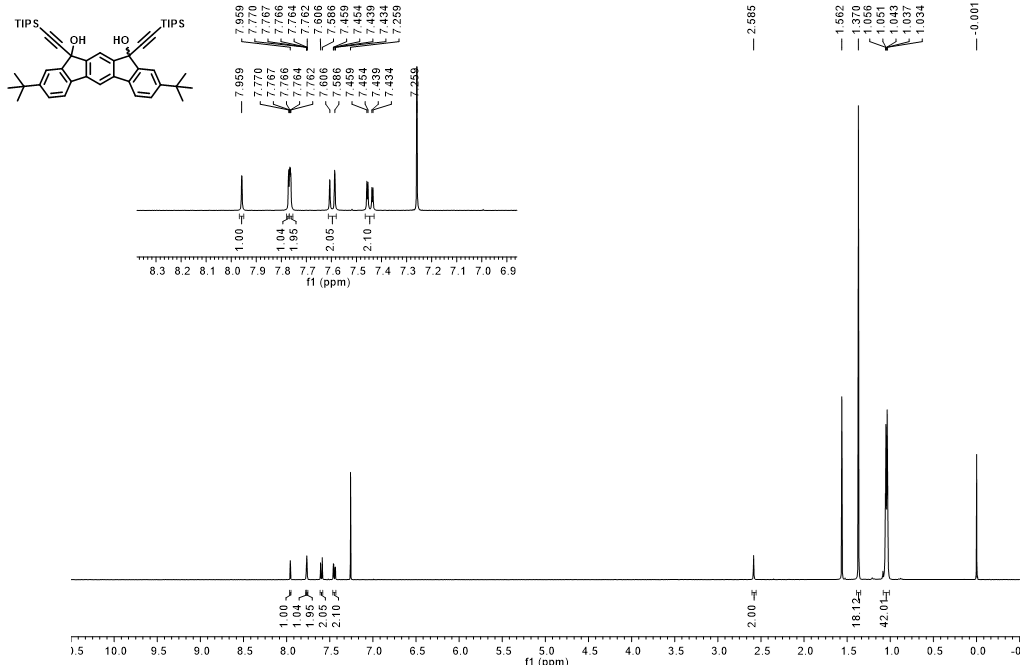
****Figure S16-2.** ^1^H NMR spectrum (400 MHz, CDCl_3_, rt) and ^13^C NMR spectrum (100 MHz, CDCl_3_, rt) of compound **10b’**.

**Figure S16-3.** ^1^H NMR spectrum (400 MHz, CDCl_3_, rt) and ^13^C NMR spectrum (100 MHz, CDCl_3_, rt) of compound
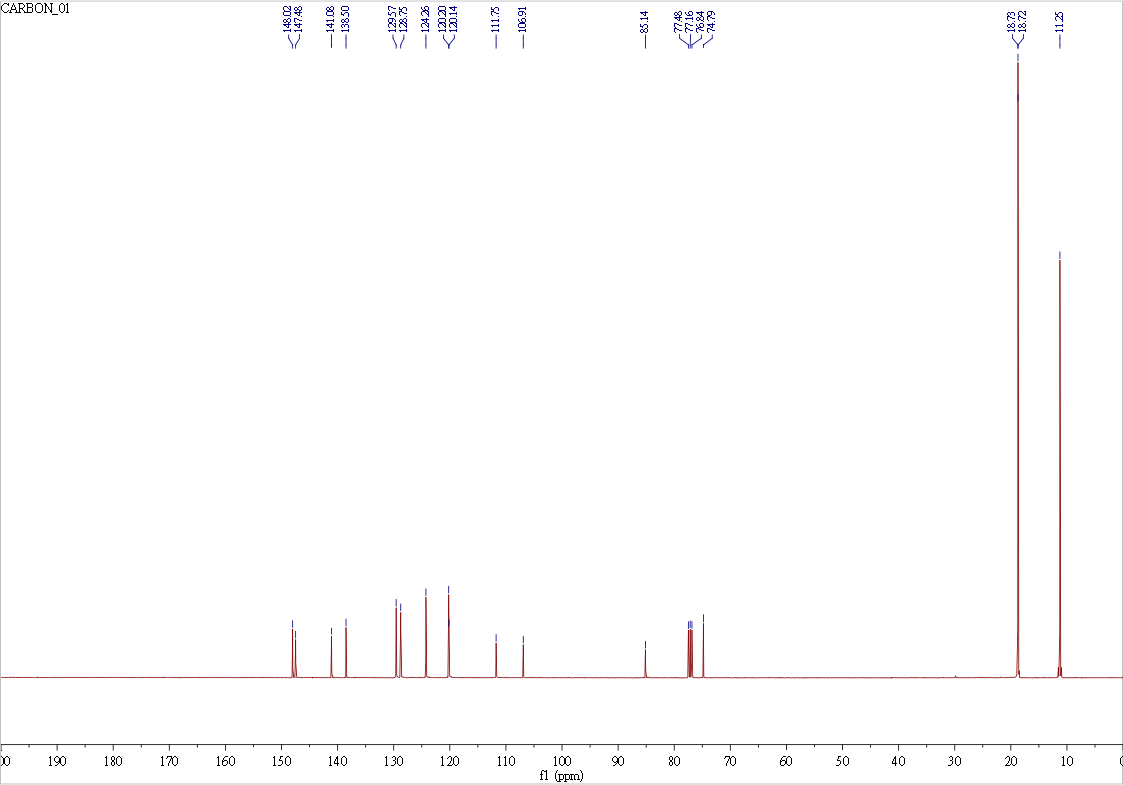

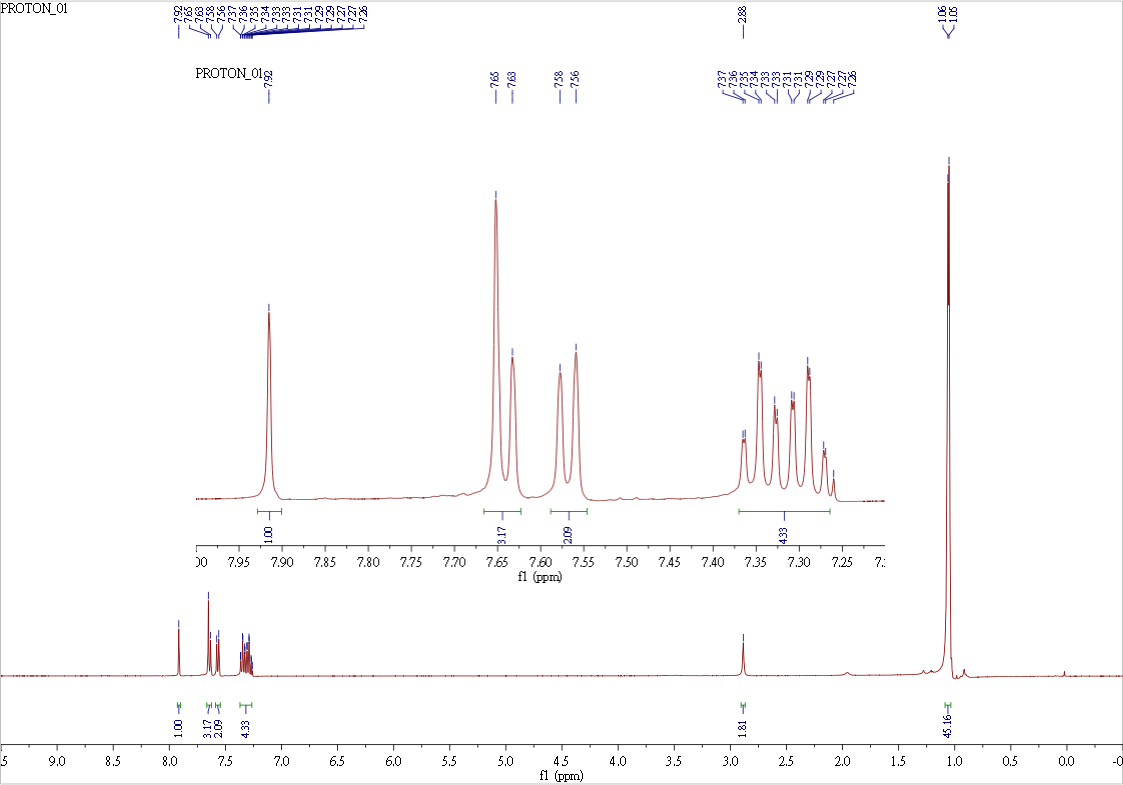
**10b**.

**
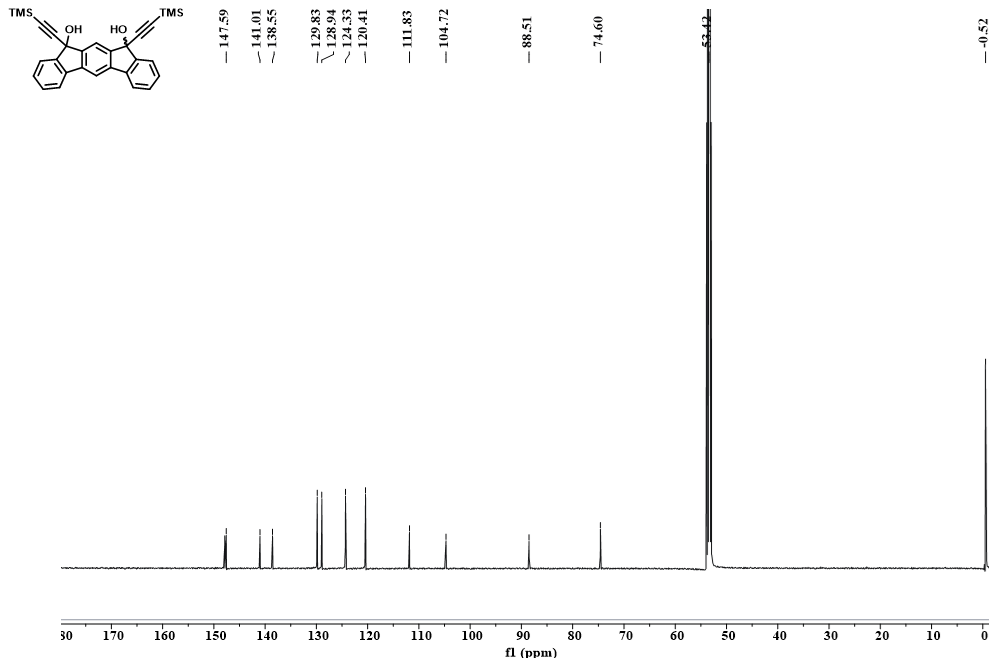

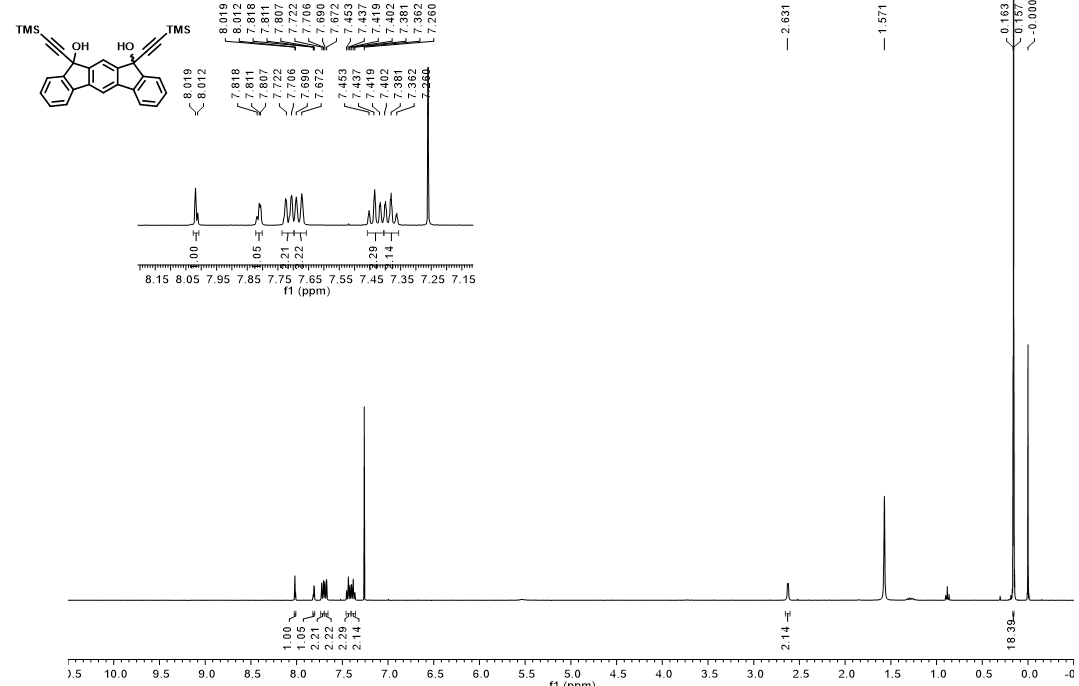
Figure S16-4.** ^1^H NMR spectrum (400 MHz, CDCl_3_, rt) and ^13^C NMR spectrum (100 MHz, CD_2_Cl_2_, rt) of compound **10a**.

**
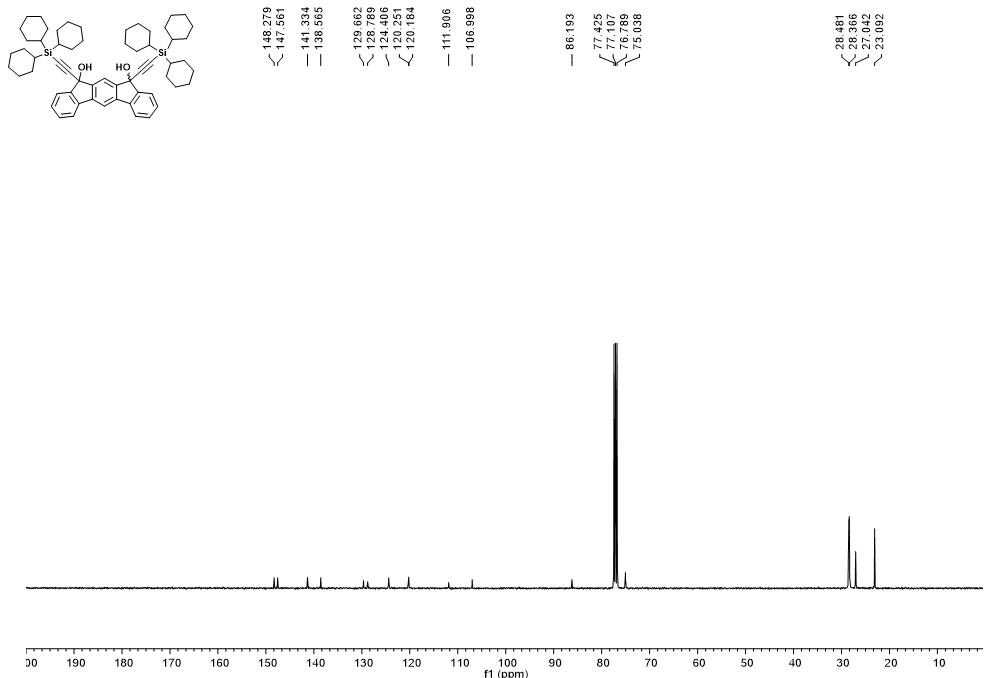
**
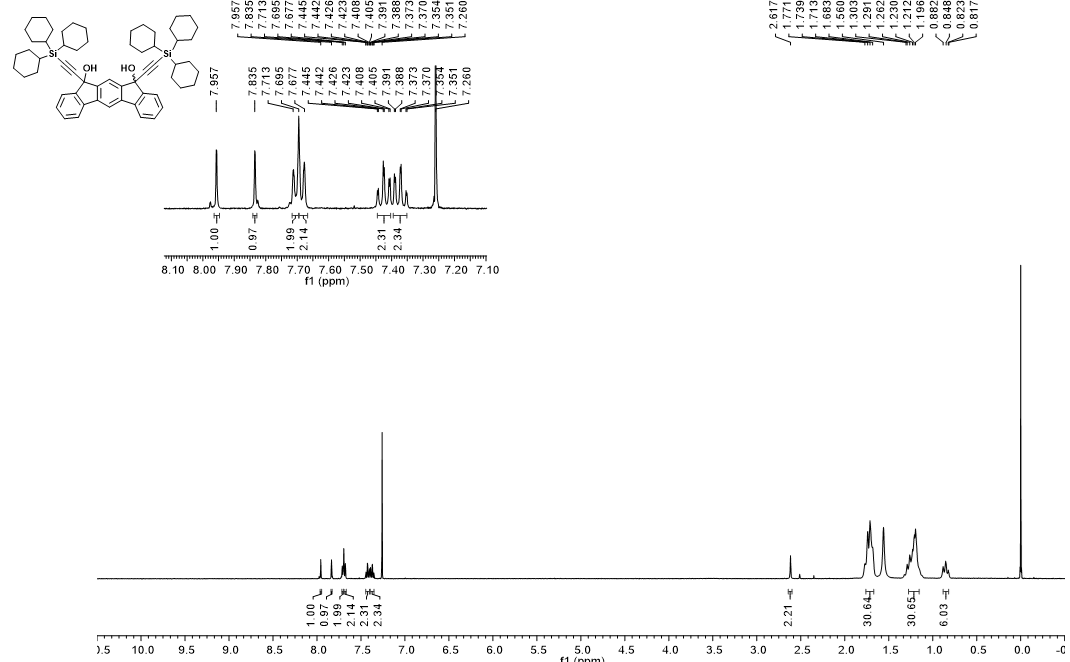
**Figure S16-5.** ^1^H NMR spectrum (400 MHz, CDCl_3_, rt) and ^13^C NMR spectrum (100 MHz, CDCl_3_, rt) of compound **10c**.

**Figure S16-6.** ^1^H NMR spectrum (400 MHz, CDCl_3_, rt) and ^13^C NMR spectrum (100 MHz, CDCl_3_, rt) of compound **
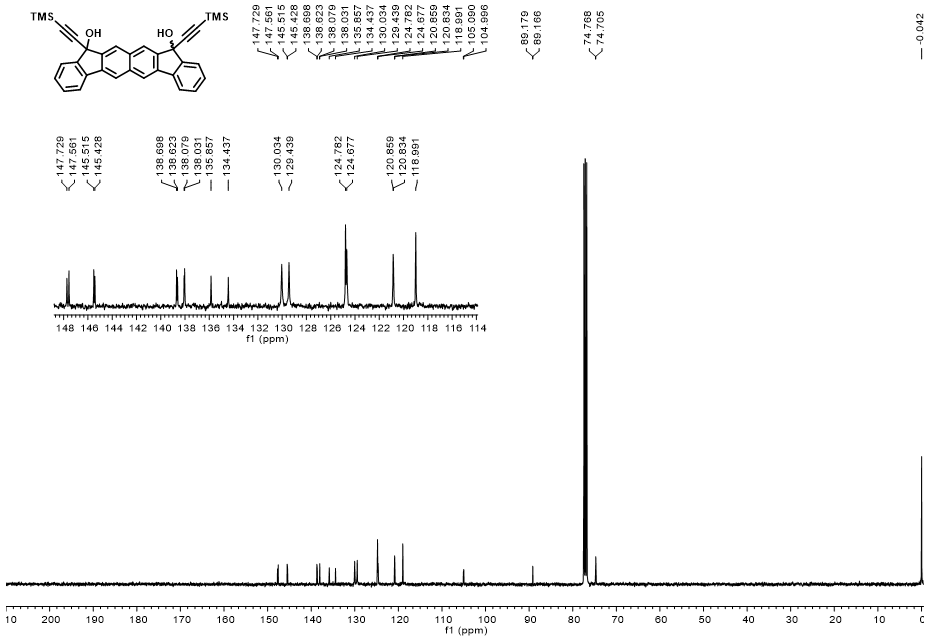

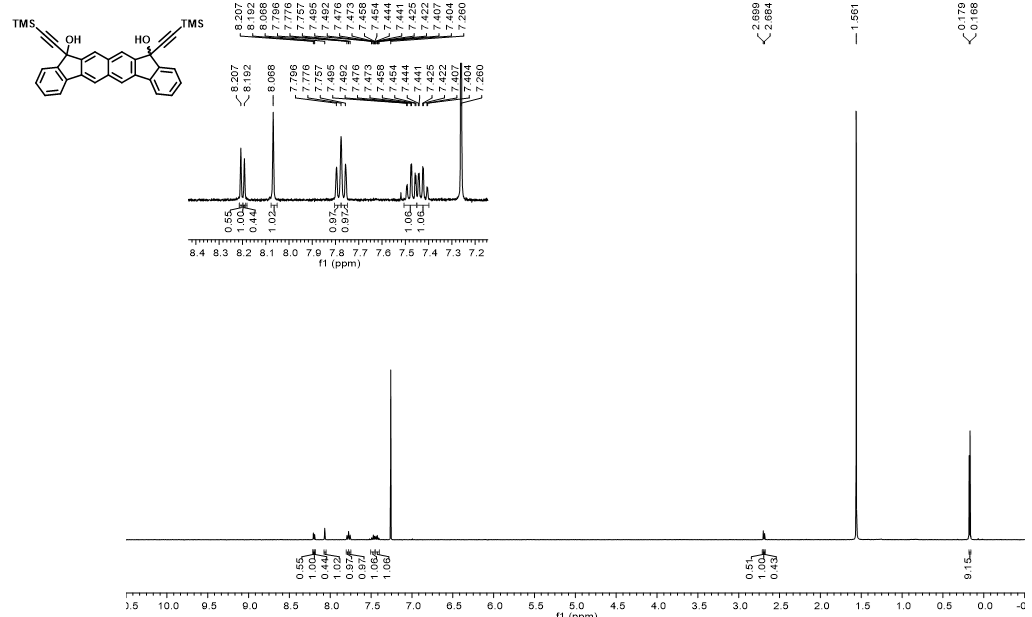
11a**.

**Figure S16-7.** ^1^H NMR spectrum (400 MHz, CDCl_3_, rt) and ^13^C NMR spectrum (100 MHz, CDCl_3_, rt) of compound
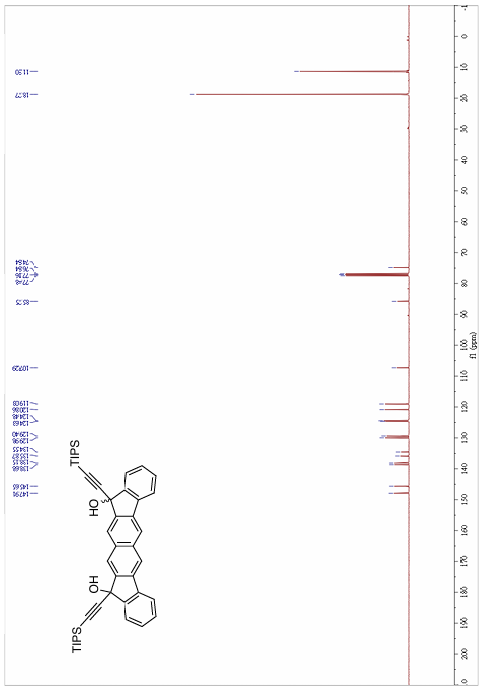
**
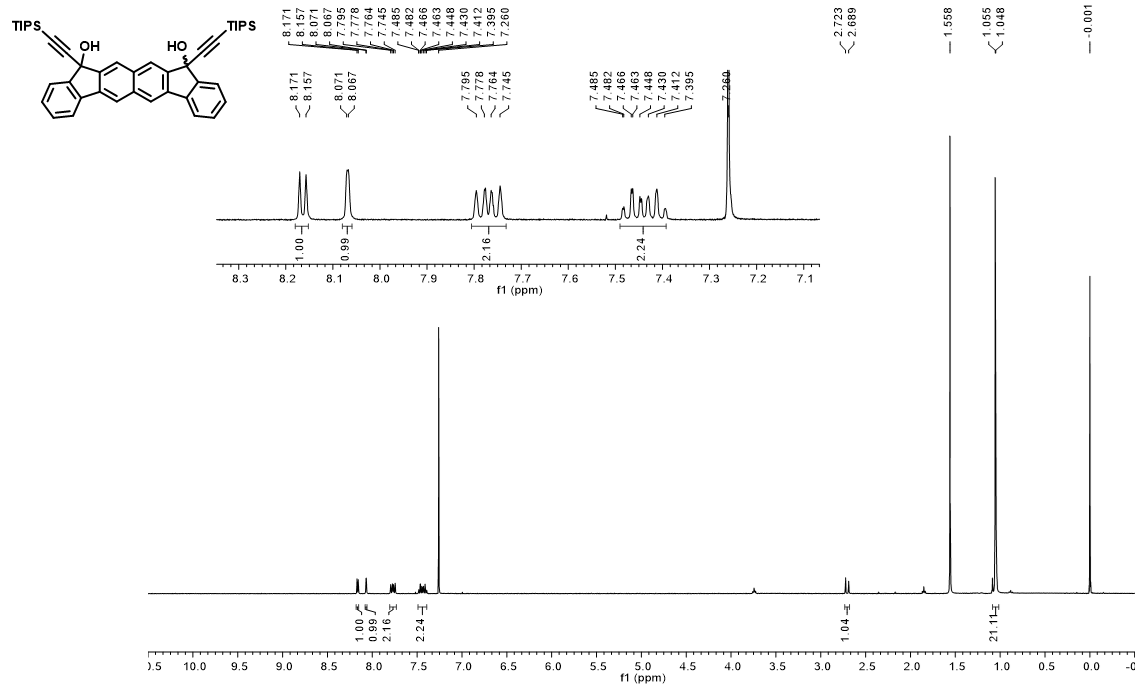
11b**.

**
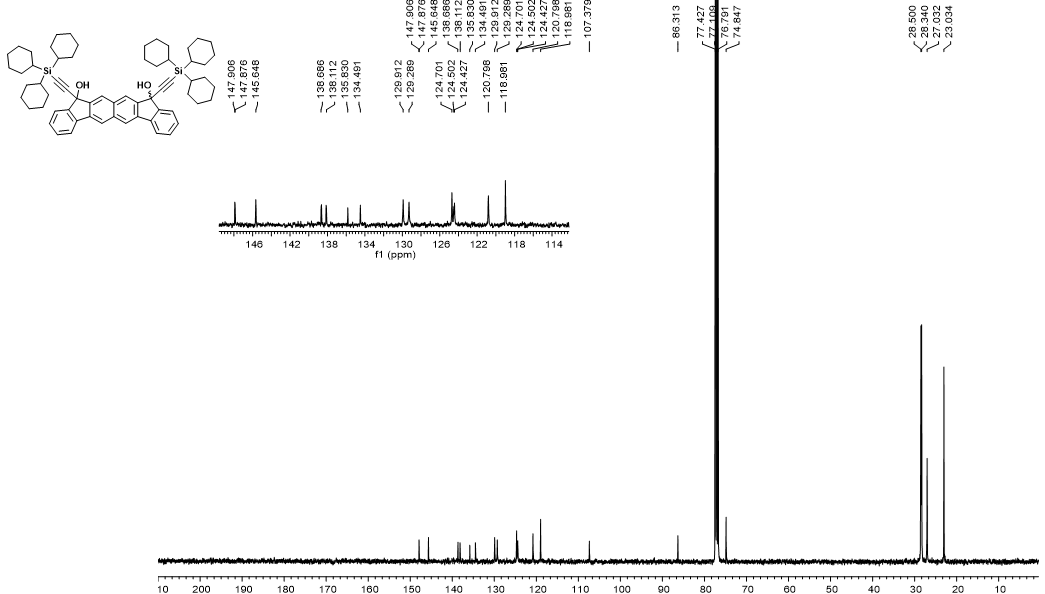

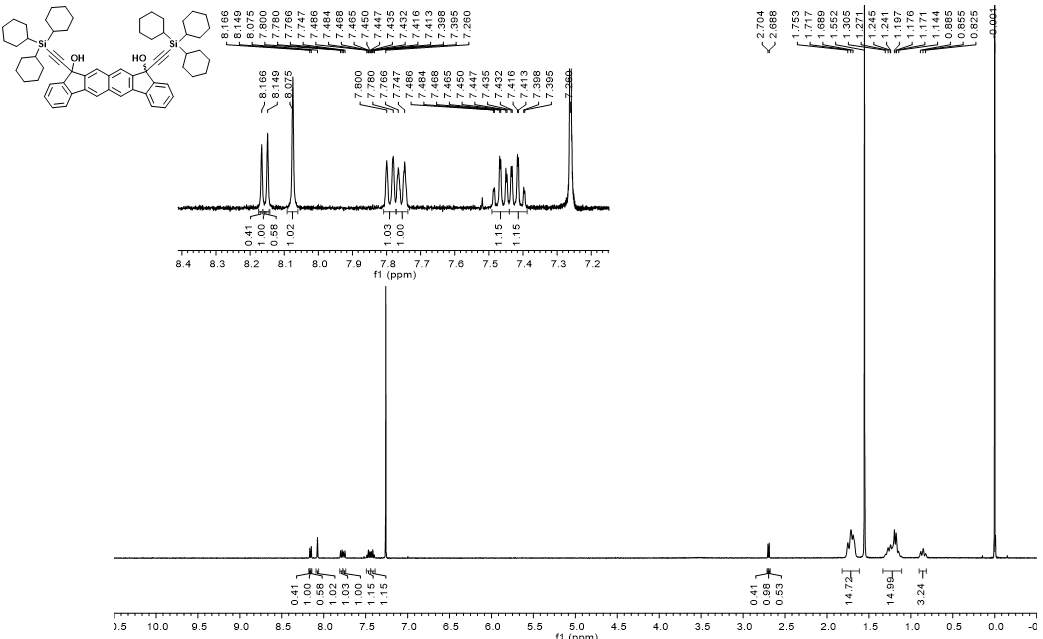
Figure S16-8.**  ^1^H NMR spectrum (400 MHz, CDCl_3_, rt) and ^13^C NMR spectrum (100 MHz, CDCl_3_, rt) of compound **11c**.

**
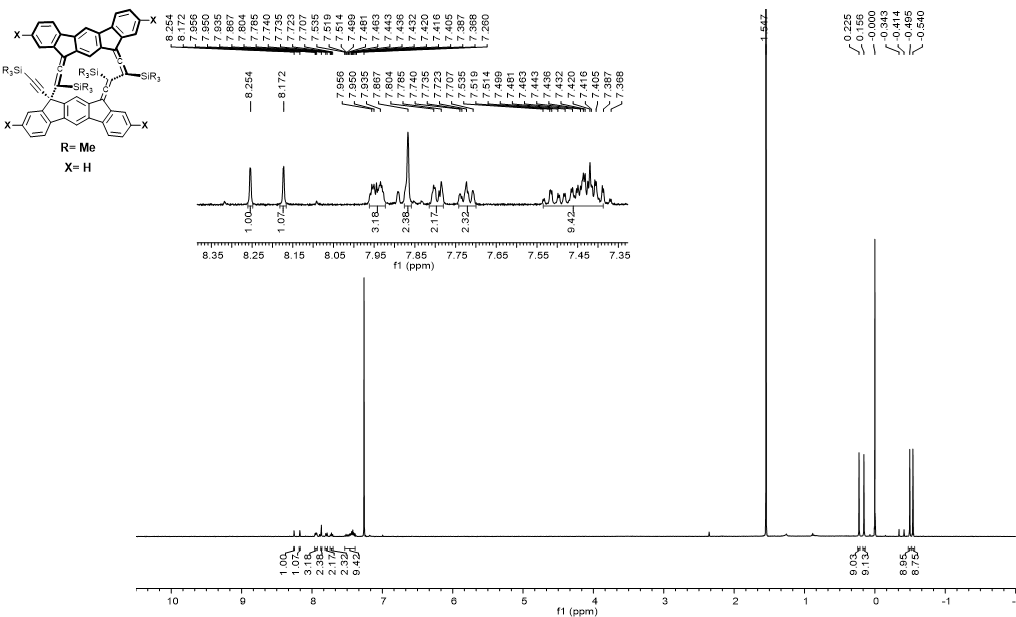
**

**
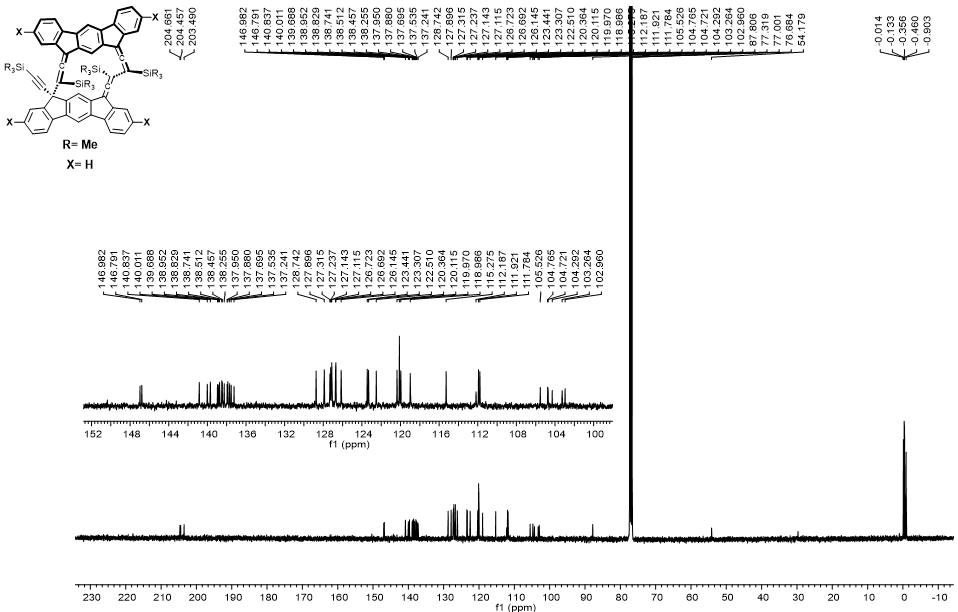
**

**Figure S16-9.** ^1^H NMR spectrum (400 MHz, CDCl_3_, rt) and ^13^C NMR spectrum (100 MHz, CDCl_3_, rt) of *H,T,T,T***-14a**.

**Figure S16-10.** ^
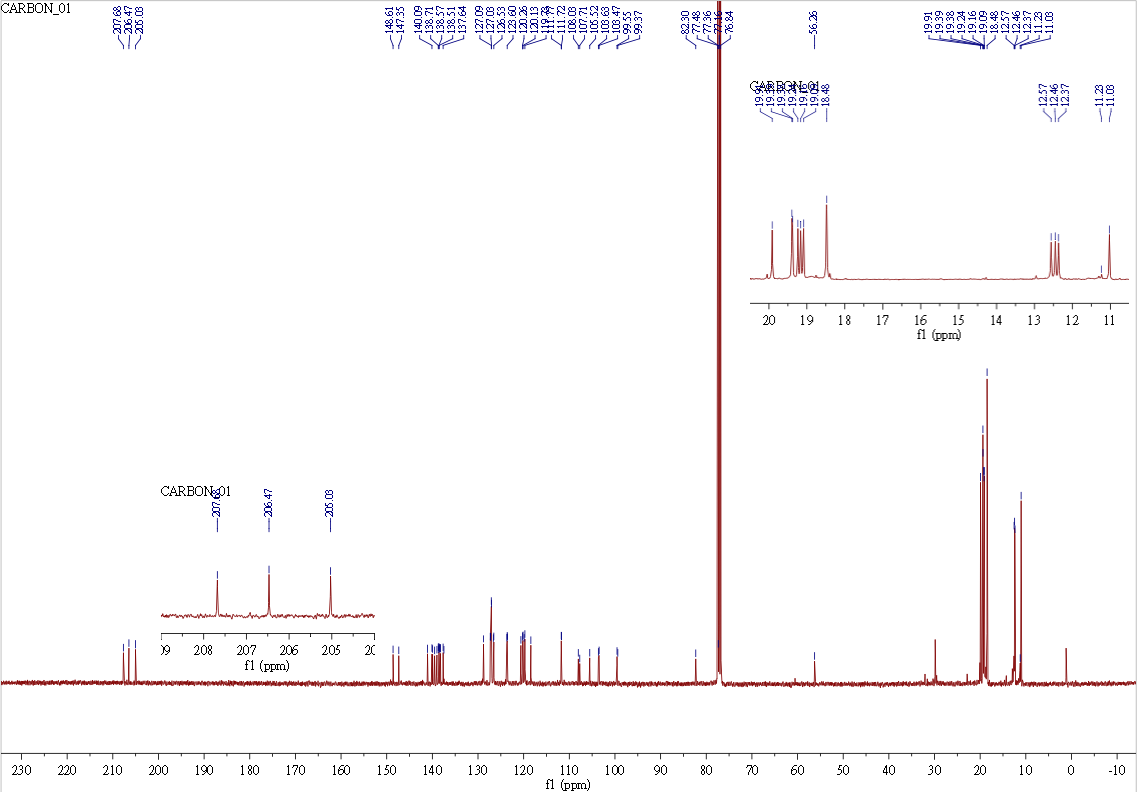

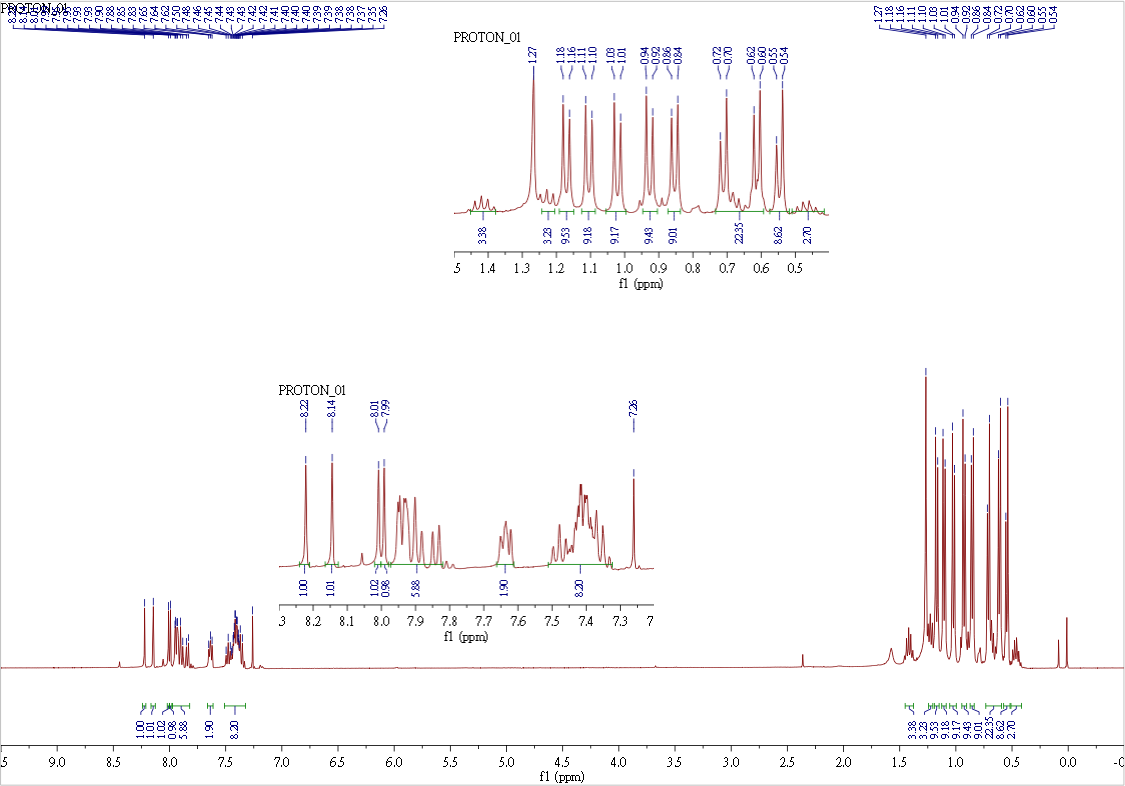
1^H NMR spectrum (400 MHz, CDCl_3_, rt) and ^13^C NMR spectrum (100 MHz, CDCl_3_, rt) of *H,T,T,T***-12a**.


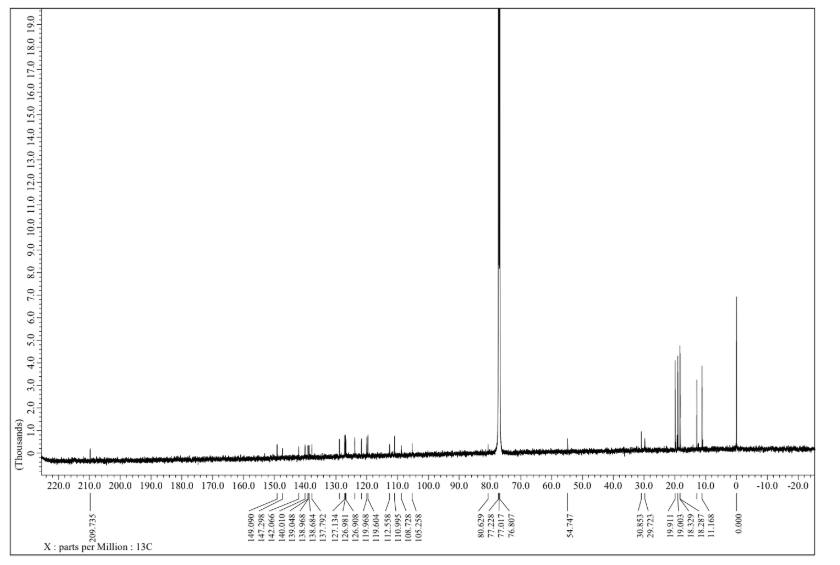

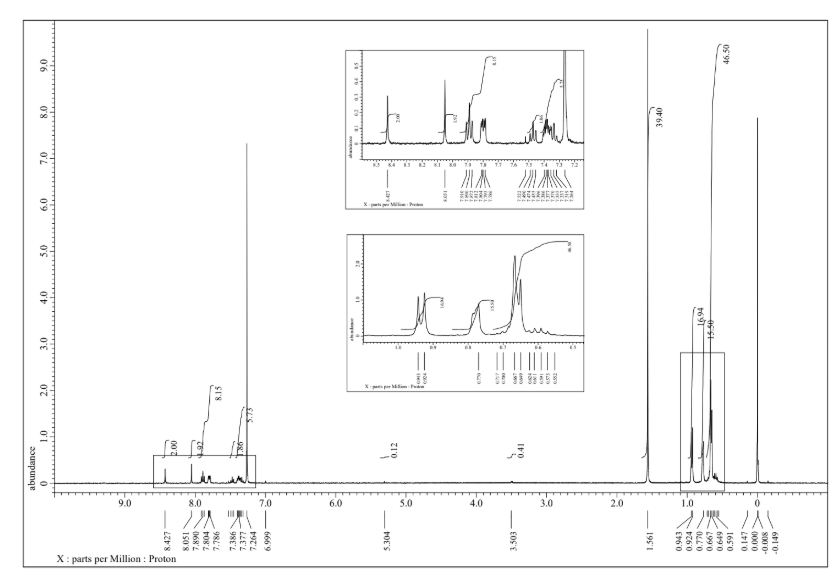
**Figure S16-11.** ^1^H NMR spectrum (400 MHz, CDCl_3_, rt) and ^13^C NMR spectrum (100 MHz, CDCl_3_, rt) of *H,T,H,T***-12b_._**

**
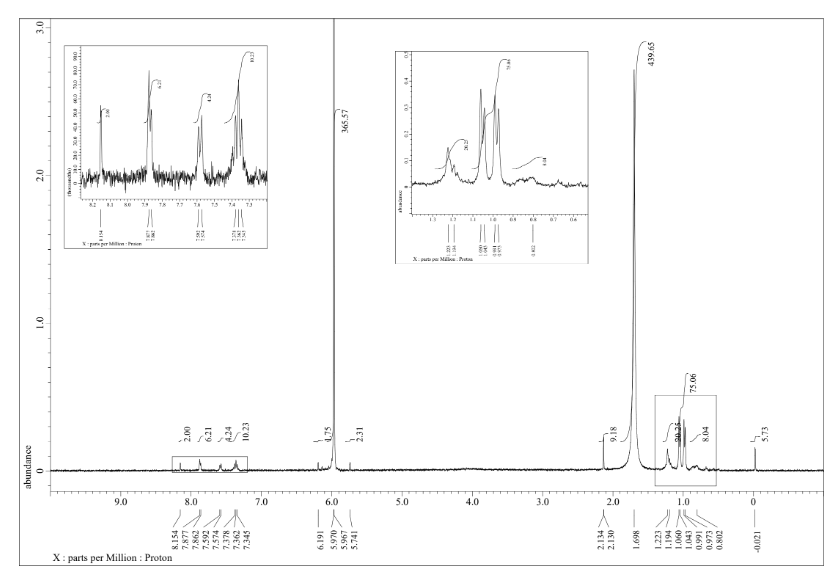
Figure S16-12.** ^1^H NMR spectrum (400 MHz, C_2_D_2_Cl_4_, 100 °C) of *T,T,T,T***-12c.**

**
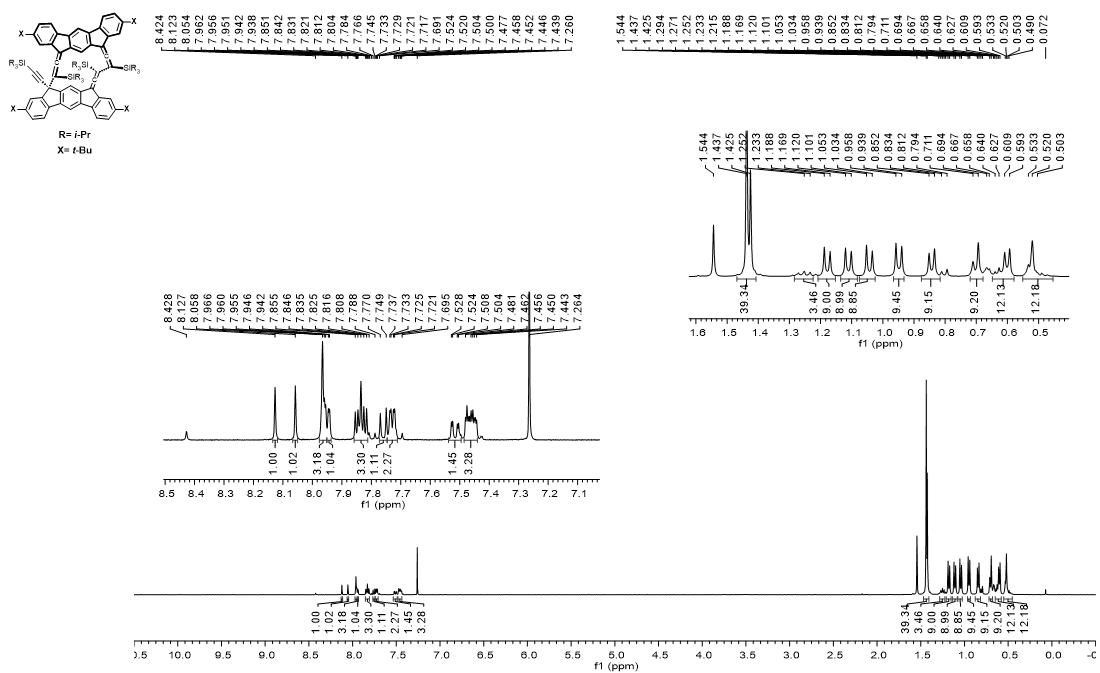
**

**
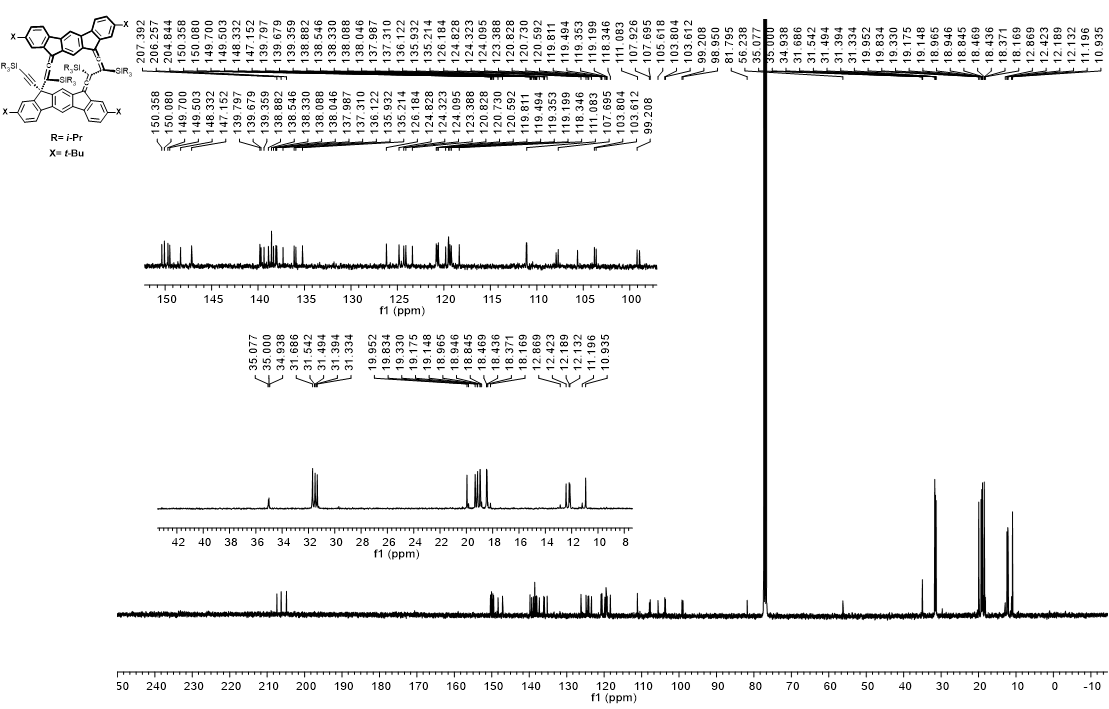
**

**Figure S16-13.** ^1^H NMR spectrum (400 MHz, CDCl_3_, rt) and ^13^C NMR spectrum (100 MHz, CDCl_3_, rt) of *H,T,T,T***-13a.**


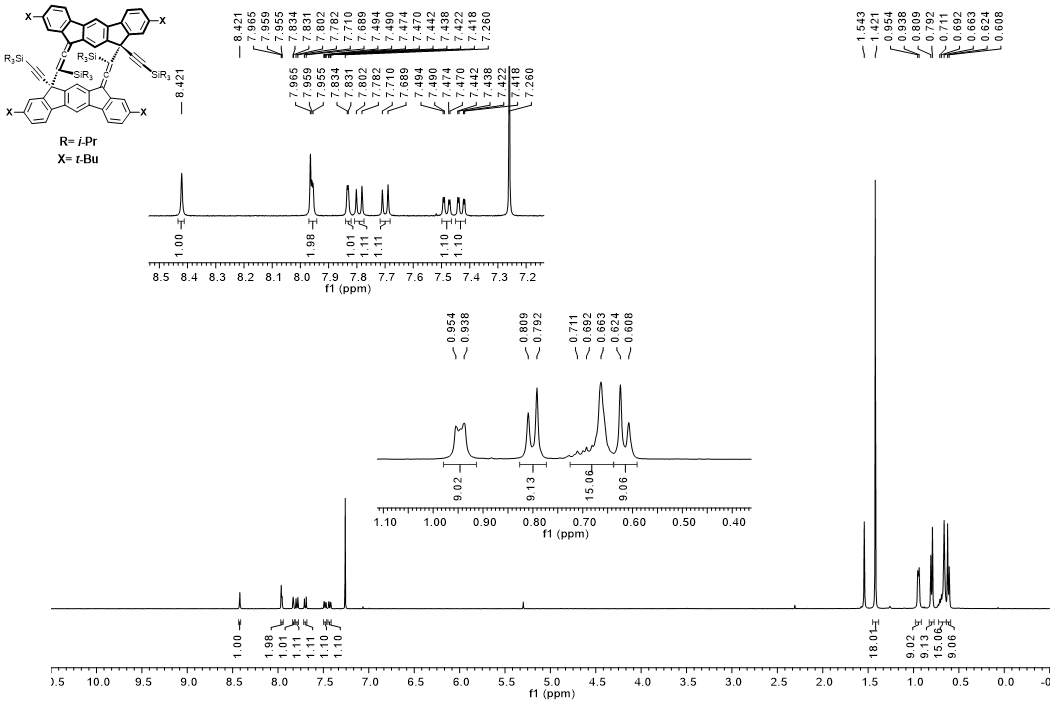


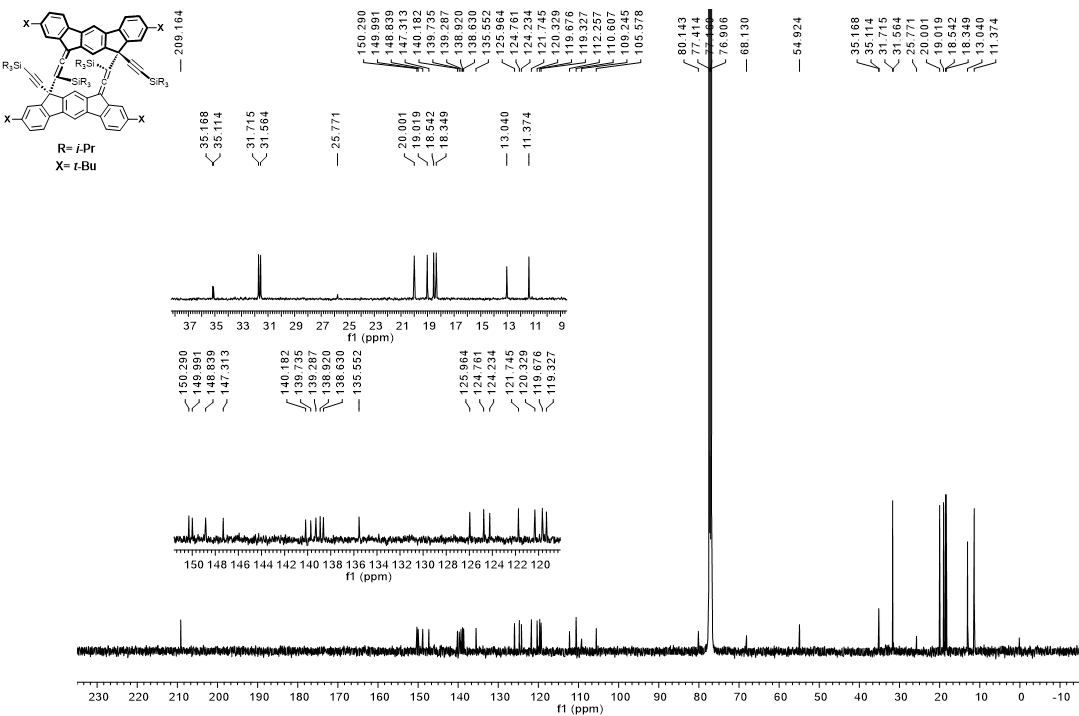


**Figure S16-14.** ^1^H NMR spectrum (400 MHz, CDCl_3_, rt) and ^13^C NMR spectrum (100 MHz, CDCl_3_, rt) of *H,T,H,T***-13b_._**


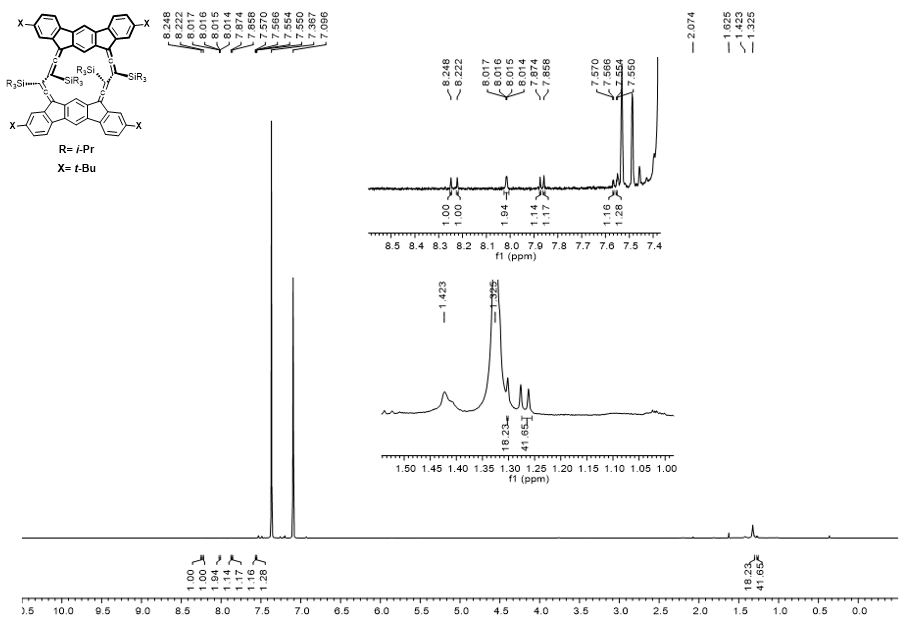


**Figure S16-15.** ^1^H NMR spectrum (500 MHz, C_6_D_4_Cl_2_, 100°C) of *T,T,T,T***-13c_._**

**
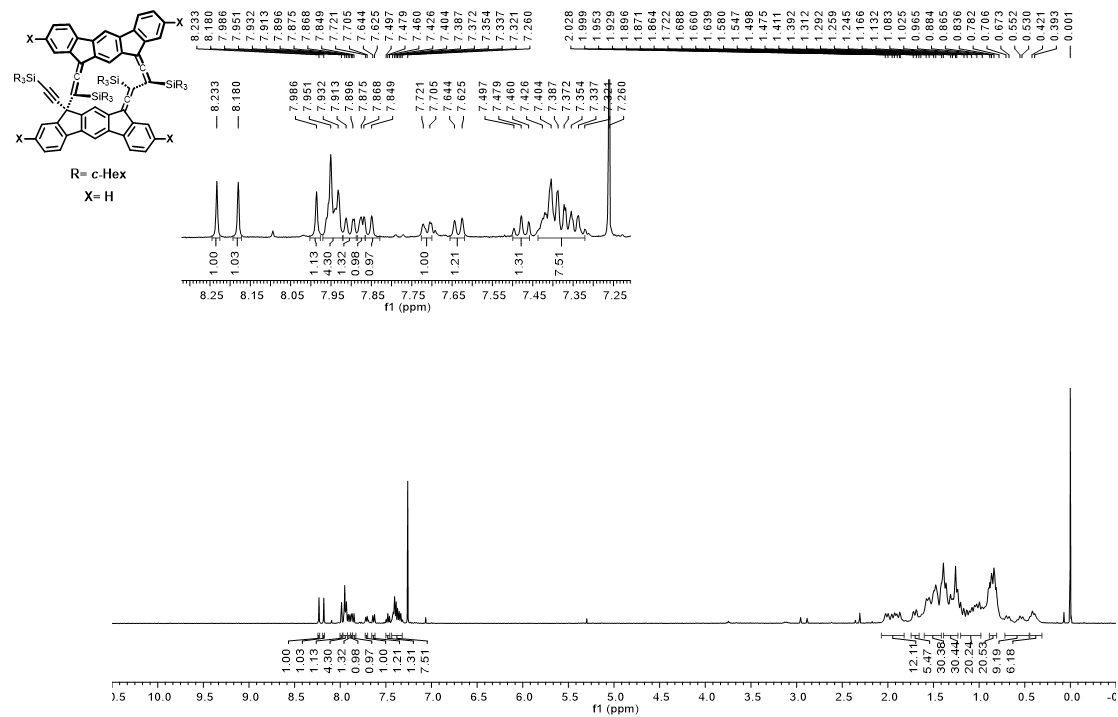
**

**
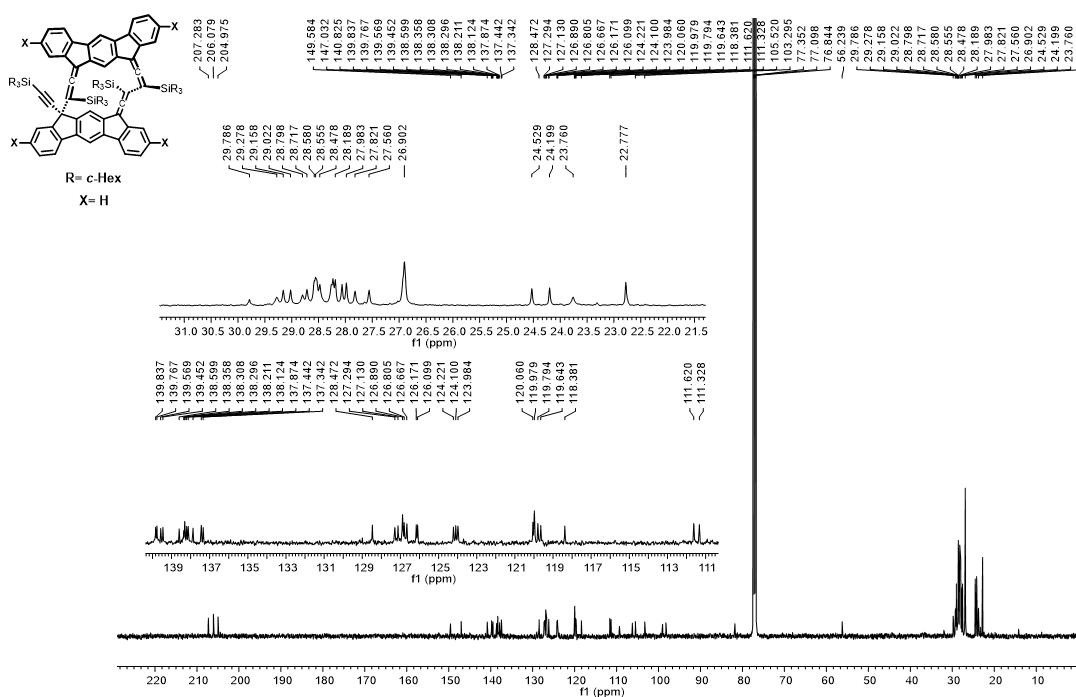
**

**Figure S16-16.**  ^1^H NMR spectrum (400 MHz, CDCl_3_, rt) and ^13^C NMR spectrum (100 MHz, CDCl_3_, rt) of *H,T,T,T***-15a_._**

**
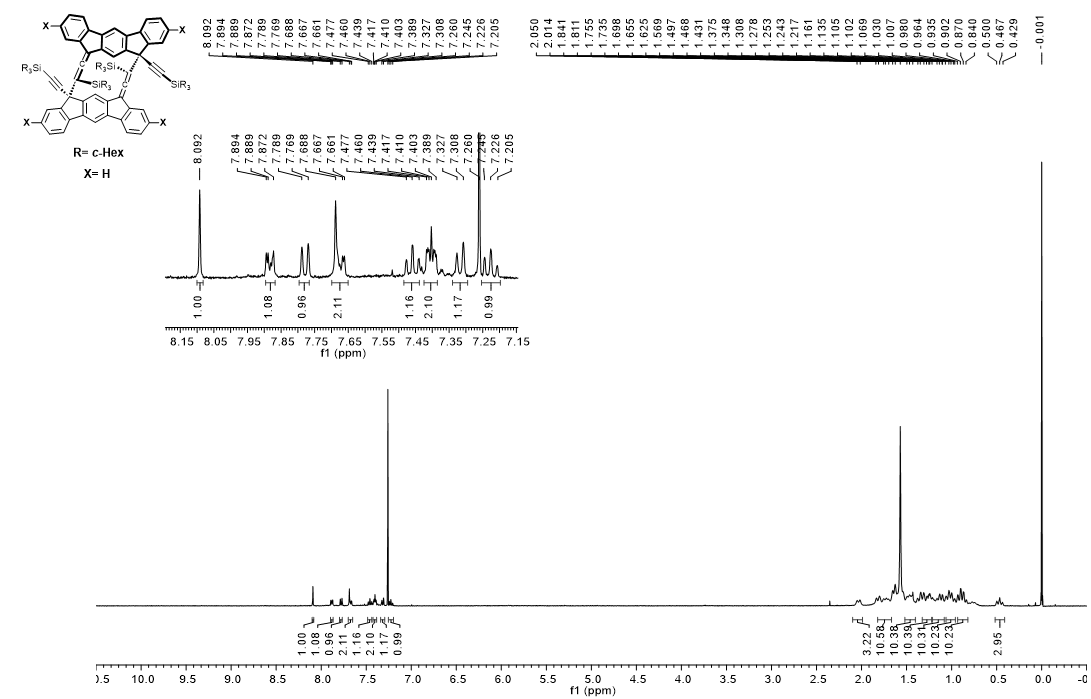
**

**
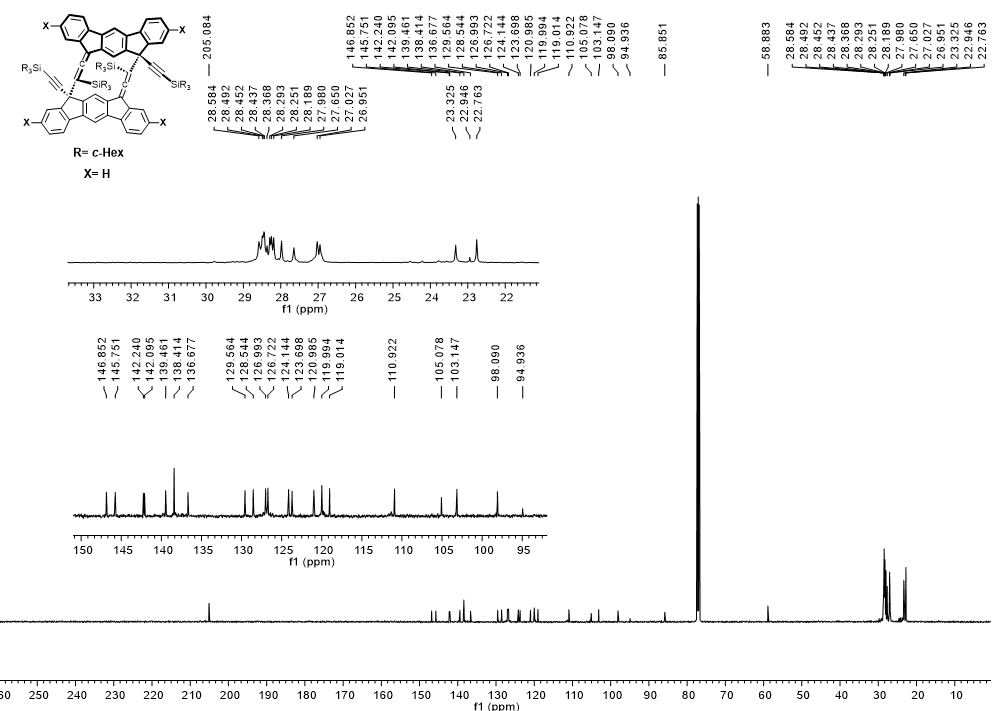
**

**Figure S16-17.**  ^1^H NMR spectrum (400 MHz, CDCl_3_, rt) and ^13^C NMR spectrum (100 MHz, CDCl_3_, rt) of *H,T,H,T***-15b_._**

**
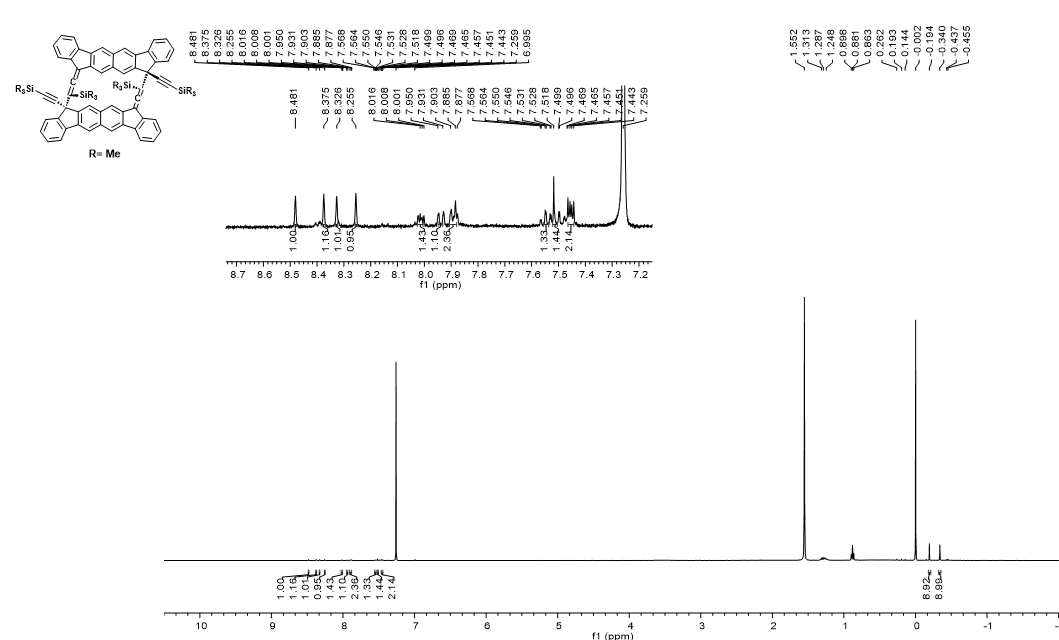
**

**Figure S16-18.** ^1^H NMR spectrum of *H,T,H,T***-16b** (400 MHz, CDCl_3_, rt).

**Figure S16-19.**
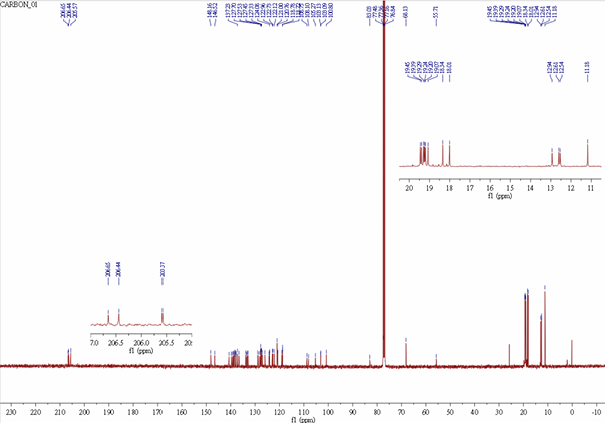

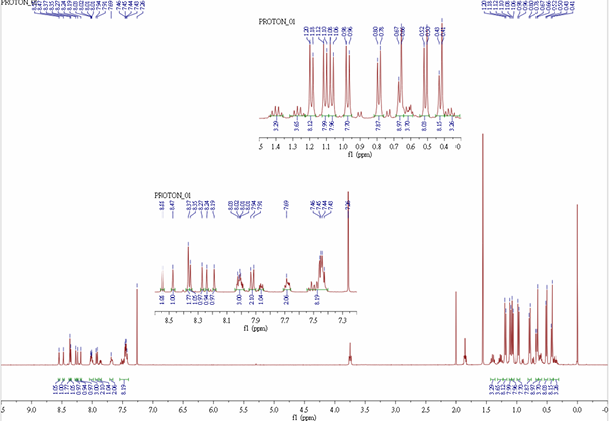
 ^1^H NMR spectrum (400 MHz, CDCl_3_, rt) and ^13^C NMR spectrum (100 MHz, CDCl_3_, rt) of *H,T,T,T***-17a.**

**
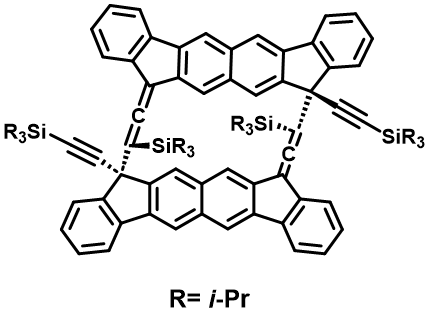
Figure S16-20.**
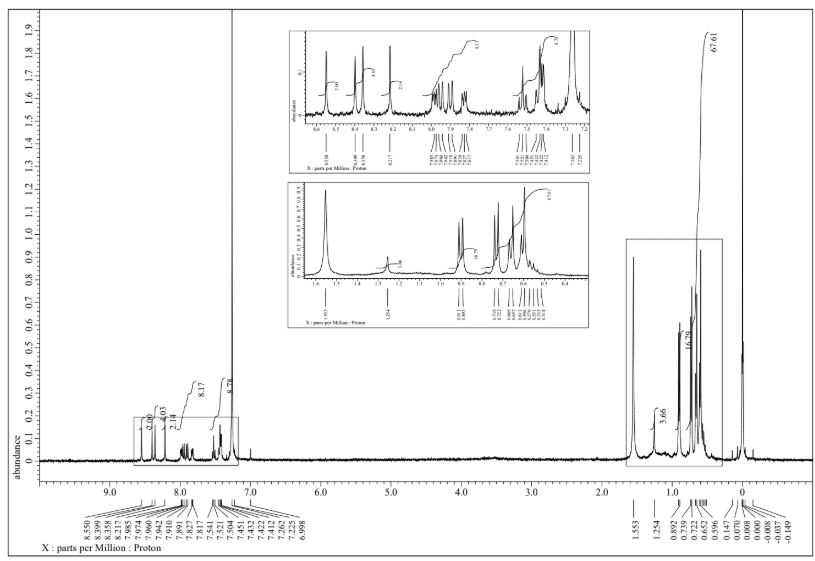
^1^H NMR spectrum (400 MHz, CDCl_3_, rt)of *H,T,H,T***-17b.**

**
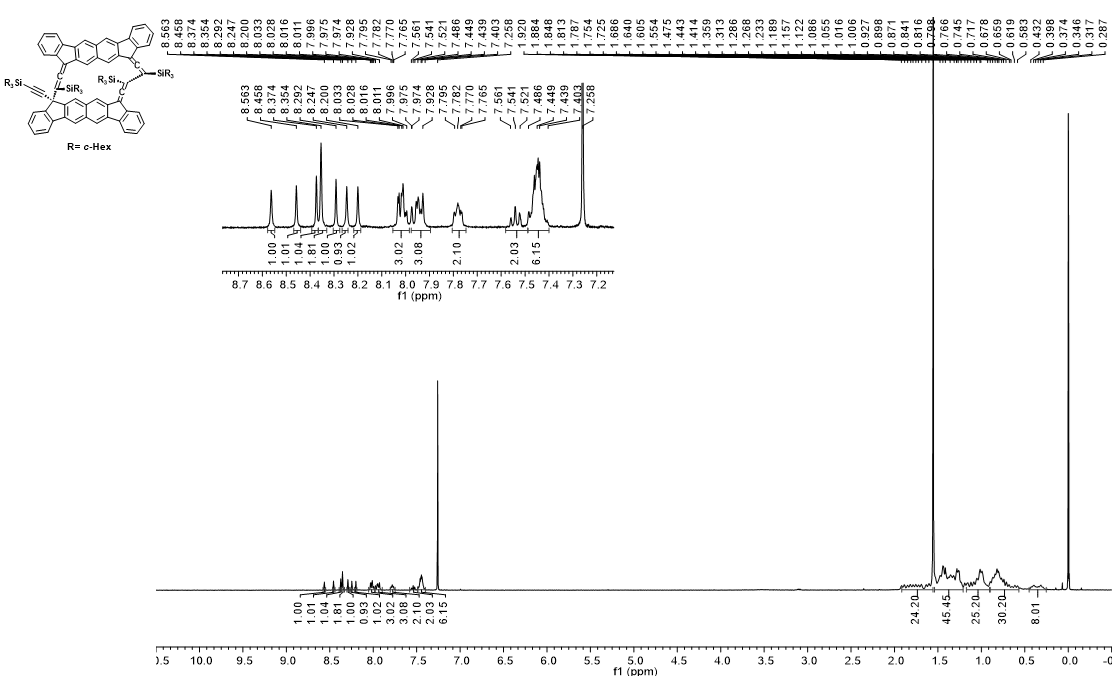
**

**
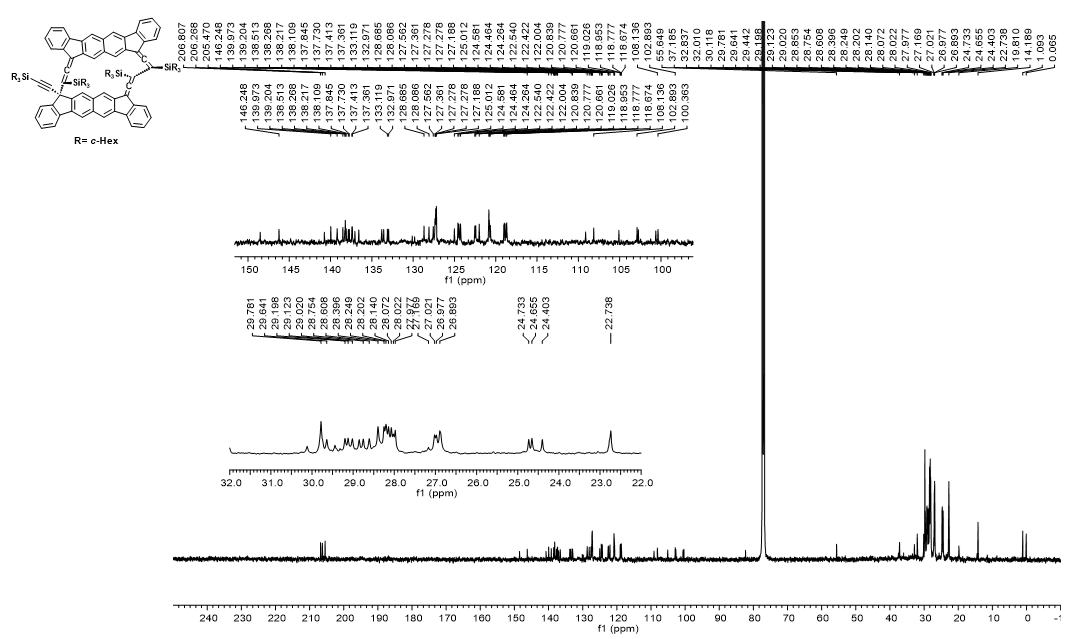
**

**Figure S16-21.** ^1^H NMR spectrum (400 MHz, CDCl_3_, rt) and ^13^C NMR spectrum (100 MHz, CDCl_3_, rt) of *H,T,T,T***-18a_._**

**
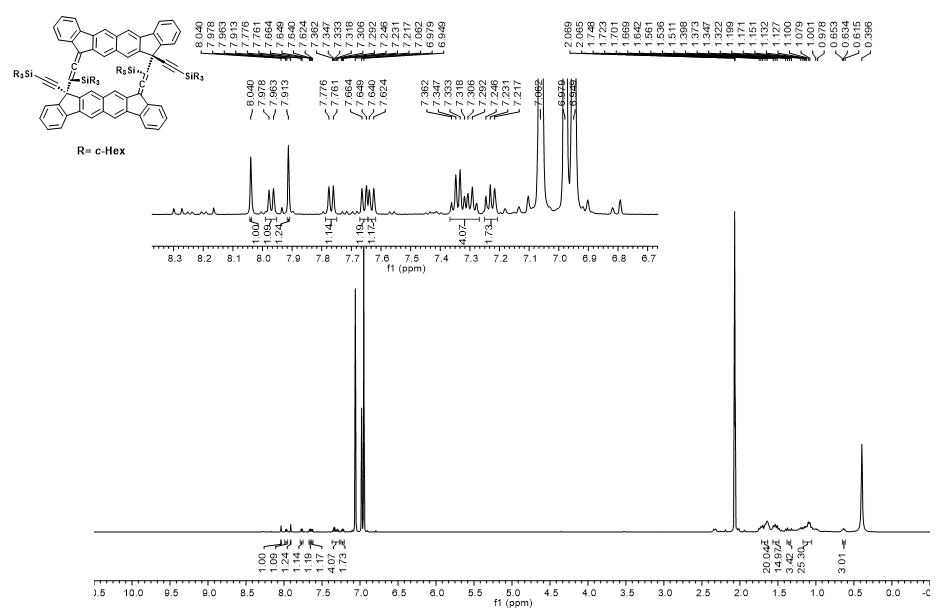
**

**Figure S16-22.** ^1^H NMR spectrum (500 MHz, C_6_D_5_CD_3_, 55 °C) of *H,T,H,T***-18b_._**

**11. References**

1. Jun-Young Jung, a. B. T. K. *J. Org. Chem* **2005**, *70* 8522–8526.
2. Tan, P. W.; Haughey, M.; Dixon, D. J. *Chem. Commun.* **2015**, *51* 4406-4409.
3. Dumartin, M.; Lipke, M. C.; Stoddart, J. F. *J. Am. Chem. Soc.* **2019**, *141* 18308–18317.
4. Miyoshi, H.; Miki, M.; Hirano, S.; Shimizu, A.; Kishi, R.; Fukuda, K.; Shiomi, D.; Sato, K.; Takui, T.; Hisaki, I.; et al. *J. Org. Chem.* **2017**, *82* 1380–1388.
5. Kubota Toru, Y. A., Endo Mikio, Hirahara Tomiaki,. Japan Patent, **1995**, JPH07291977A
6. Anthony, J. E. *Org. Lett.* **2010**, *12* 2060–2063.
7. Chardonnens, L.; Chardonnens, H. *Helv. Chim. Acta* **1858**. *41*. 2109–2111
8. Bruker (**2019**). Apex3 v2019.1-0, SAINT V8.40A, Bruker AXS Inc.: Madison (WI), USA.
9. Bruker (**2008**). *SADABS* (Version 2008/1). Bruker AXS Inc., Madison, Wisconsin, USA.
10. Sheldrick, G. M. (**2015**). Crystal structure refinement with SHELXL. *Acta Crystallographica Section C: Structural Chemistry*, *71*(1), 3–8.
11. Johnson, E. R.; Keinan, S.; Mori-Sánchez, P.; Contreras-García, J.; Cohen, A. J.; Yang, W. Revealing Noncovalent Interactions. J. Am. Chem. Soc. **2010**, *132*, 6498–6506.
12. Lu, T.; Chen, F. Multiwfn: A Multifunctional Wavefunction Analyzer. J. Comput. Chem. **2012**, *33*, 580–592.
13. Humphrey, W.; Dalke, A.; Schulten, K. VMD: Visual Molecular Dynamics. J. Mol. Graphics **1996**, *14*, 33−38.
14. Williams, T.; Kelley, C. Gnuplot 4.5: an interactive plotting program. URL http://gnuplot.sourceforge.net/ (Last accessed: 2022 January 12)
15. S. Qiu, Y. Zhang, X. Huang, L. Bao, Y. Hong, Z. Zeng, J. Wu, *Org. Lett.* 2016, *18*, 6018−6021.
