# Supplementary material for: Taming m‐Quionodimethanes by Dispersion Force: Cyclodimerization of (Trialkylsilyl)Ethynyl‐Substituted Indeno[2,1‐b]Fluorenes and Fluoreno[2,3‐b]Fluorenes
Source: Chemistry. 2025 Aug 19;31(54):e02053. doi: 10.1002/chem.202502053 (PMC12462229; doi:10.1002/chem.202502053)
Supplement: Supplementary file 2 — Supporting Information [file CHEM-31-e02053-s002.docx]

**Supporting Information for**

**Taming *m*-Quionodimethanes by Dispersion Force: Cyclodimerization of (Trialkylsilyl)Ethynyl-Substituted** **Indeno[2.1-*b*]Fluorenes and** **Fluoreno[2.3-*b*]Fluorenes**

**12. Atomic Coordinates of Theoretically Optimized Structures**

**Indeno[2.1-*b*]fluorenes**

**Compound 4c closed-shell**

C 1.19864200 -2.72173900 -0.00142400

C -0.00013700 -3.43292600 -0.00003500

C -1.19886900 -2.72166000 0.00134000

C -1.19161100 -1.28427900 0.00124400

C -0.00004400 -0.56141900 -0.00005600

C 1.19147700 -1.28435700 -0.00134000

C 2.54810100 -0.80866000 -0.00240700

C 3.40090200 -1.95390200 -0.00322100

C 2.57887100 -3.13560300 -0.00271800

C 4.80309700 -2.05294600 -0.00426300

C 5.37922400 -3.30777400 -0.00491600

C 4.57462700 -4.46914900 -0.00450800

C 3.19002800 -4.39406500 -0.00339800

C -2.57912400 -3.13543500 0.00270200

C -3.40107900 -1.95368000 0.00326500

C -2.54820500 -0.80849500 0.00242600

C 2.93373200 0.53146100 -0.00192600

C 3.23600000 1.71379500 -0.00075300

C -3.19036300 -4.39385800 0.00337800

C -4.57496600 -4.46885200 0.00455900

C -5.37948900 -3.30742500 0.00503600

C -4.80328100 -2.05263400 0.00437500

C -2.93373800 0.53165500 0.00194500

C -3.23591900 1.71401100 0.00078300

Si 3.63328700 3.50856300 0.00446300

C 4.94295600 3.82898600 1.31478600

C 4.27601600 3.98387800 -1.69778800

C 2.05320200 4.44844800 0.39986000

Si -3.63300200 3.50882700 -0.00447000

C -4.27564700 3.98441100 1.69773100

C -2.05272800 4.44837800 -0.39990900

C -4.94269300 3.82928200 -1.31476800

H -0.00017200 -4.51811200 -0.00002900

H -0.00000900 0.52334600 -0.00003700

H 5.40996500 -1.15496500 -0.00444100

H 6.45775800 -3.41005600 -0.00570800

H 5.05525300 -5.44090800 -0.00505000

H 2.59320000 -5.29893200 -0.00305600

H -2.59359300 -5.29876300 0.00298300

H -5.05565500 -5.44058000 0.00510600

H -6.45802900 -3.40963700 0.00588700

H -5.41009000 -1.15461300 0.00460200

H 5.85511500 3.26443200 1.10502400

H 4.58738900 3.53717100 2.30618600

H 5.20278600 4.89152600 1.34694100

H 5.17956700 3.42215600 -1.94806200

H 3.52975900 3.78174800 -2.47023800

H 4.51953500 5.05028700 -1.73197800

H 2.23781600 5.52681400 0.41849900

H 1.65826000 4.15652400 1.37647600

H 1.28094700 4.25178200 -0.34799100

H -3.52948200 3.78206400 2.47021500

H -5.17940900 3.42303100 1.94800700

H -4.51877600 5.05091100 1.73184900

H -1.28075100 4.25191300 0.34828200

H -1.65753300 4.15593100 -1.37626300

H -2.23715100 5.52676500 -0.41908200

H -4.58717200 3.53751500 -2.30619500

H -5.20257700 4.89181300 -1.34686500

H -5.85482100 3.26468800 -1.10497100

**Compound 4c open-shell singlet**

C 1.19528400 2.70192500 0.00147700

C -0.00000100 3.41717800 0.00000100

C -1.19528400 2.70192500 -0.00147600

C -1.19009100 1.27043600 -0.00132900

C 0.00000000 0.54676600 -0.00000300

C 1.19009100 1.27043700 0.00132500

C 2.55843700 0.80052600 0.00252200

C 3.41065000 1.96186100 0.00356400

C 2.58552400 3.13067900 0.00301400

C 4.80497400 2.07172500 0.00480600

C 5.37364300 3.33870300 0.00563000

C 4.56564900 4.48693700 0.00517900

C 3.17483100 4.39166600 0.00384400

C -2.58552400 3.13067800 -0.00301300

C -3.41065100 1.96186000 -0.00356600

C -2.55843700 0.80052600 -0.00252500

C 2.94951800 -0.53154100 0.00173600

C 3.25443000 -1.71569300 0.00011800

C -3.17483100 4.39166500 -0.00384000

C -4.56565000 4.48693600 -0.00517600

C -5.37364400 3.33870200 -0.00563100

C -4.80497400 2.07172300 -0.00480900

C -2.94951700 -0.53154200 -0.00174200

C -3.25442700 -1.71569300 -0.00012600

Si 3.64743600 -3.51123600 -0.00485200

C 4.95082600 -3.83709500 -1.32015100

C 4.29566200 -3.98721800 1.69509800

C 2.06310800 -4.44693900 -0.39339200

Si -3.64743500 -3.51123600 0.00485300

C -4.29569600 -3.98722000 -1.69508400

C -2.06310000 -4.44693900 0.39336200

C -4.95079900 -3.83709300 1.32017900

H -0.00000100 4.50180600 0.00000300

H 0.00000000 -0.53736000 -0.00000400

H 5.42197800 1.18070700 0.00502200

H 6.45180900 3.44619200 0.00659600

H 5.03347500 5.46464600 0.00584700

H 2.56634800 5.28887000 0.00345600

H -2.56634900 5.28886900 -0.00345000

H -5.03347600 5.46464400 -0.00584200

H -6.45180900 3.44619000 -0.00659700

H -5.42197800 1.18070600 -0.00502800

H 5.86570800 -3.27535500 -1.11468100

H 4.59214700 -3.54500500 -2.31037000

H 5.20710600 -4.90048600 -1.35253600

H 5.20206300 -3.42826600 1.94131400

H 3.55318600 -3.78202600 2.47040700

H 4.53587300 -5.05436300 1.72938000

H 2.24419700 -5.52592900 -0.41092400

H 1.66592800 -4.15515400 -1.36913700

H 1.29381700 -4.24638500 0.35650100

H -3.55323800 -3.78202200 -2.47040900

H -5.20210500 -3.42827300 -1.94127900

H -4.53590100 -5.05436600 -1.72936200

H -1.29382500 -4.24638800 -0.35654800

H -1.66589800 -4.15515100 1.36909800

H -2.24418900 -5.52592900 0.41090100

H -4.59209800 -3.54501000 2.31039100

H -5.20708500 -4.90048300 1.35256400

H -5.86568200 -3.27534800 1.11473000

**Compound 4c open-shell triplet**

C 1.19287200 2.68711300 0.00151400

C -0.00000900 3.40449400 0.00000000

C -1.19288700 2.68710800 -0.00151400

C -1.19190200 1.25071000 -0.00134200

C -0.00000300 0.52641600 0.00000000

C 1.19189400 1.25071500 0.00134100

C 2.55651000 0.79317600 0.00254900

C 3.41374600 1.96888400 0.00368200

C 2.58824600 3.12894400 0.00312900

C 4.80000400 2.08521000 0.00498300

C 5.36443700 3.36273400 0.00586800

C 4.55674800 4.50159100 0.00541400

C 3.16093500 4.39158900 0.00400300

C -2.58826300 3.12893300 -0.00312900

C -3.41375800 1.96886900 -0.00368200

C -2.55651600 0.79316500 -0.00255000

C 2.95825200 -0.53372300 0.00166800

C 3.27227900 -1.71631300 -0.00007700

C -3.16095600 4.39157600 -0.00400200

C -4.55677100 4.50157100 -0.00541300

C -5.36445400 3.36271100 -0.00586800

C -4.80001500 2.08518900 -0.00498300

C -2.95825300 -0.53373400 -0.00166900

C -3.27227400 -1.71632700 0.00007600

Si 3.66682800 -3.51082600 -0.00493600

C 4.96651200 -3.83865800 -1.32370000

C 4.32076100 -3.98727400 1.69288700

C 2.08177400 -4.44777700 -0.38832100

Si -3.66680900 -3.51084200 0.00493600

C -4.32076300 -3.98729200 -1.69287800

C -2.08174300 -4.44778200 0.38829600

C -4.96647100 -3.83868700 1.32371900

H -0.00001100 4.48875400 0.00000000

H -0.00000100 -0.55708900 0.00000000

H 5.42457500 1.19953500 0.00520300

H 6.44253800 3.47230300 0.00688800

H 5.01581400 5.48314000 0.00612100

H 2.54398700 5.28312200 0.00359800

H -2.54401300 5.28311100 -0.00359800

H -5.01584100 5.48311900 -0.00612000

H -6.44255600 3.47227600 -0.00688800

H -5.42458300 1.19951100 -0.00520400

H 5.88207800 -3.27673600 -1.12168900

H 4.60490100 -3.54775000 -2.31321200

H 5.22267400 -4.90210300 -1.35555900

H 5.22788400 -3.42823400 1.93633400

H 3.58072200 -3.78221200 2.47057000

H 4.56123900 -5.05439500 1.72634400

H 2.26281700 -5.52682000 -0.40383900

H 1.68279600 -4.15794500 -1.36390900

H 1.31373200 -4.24533700 0.36239600

H -3.58073900 -3.78221700 -2.47057300

H -5.22789800 -3.42826200 -1.93630800

H -4.56122800 -5.05441600 -1.72633600

H -1.31371400 -4.24533700 -0.36243300

H -1.68275100 -4.15794700 1.36387800

H -2.26277800 -5.52682600 0.40381800

H -4.60484600 -3.54778400 2.31322600

H -5.22263000 -4.90213300 1.35557400

H -5.88204200 -3.27676600 1.12172500

**Compound 4d closed-shell**

C -0.78238200 3.87711900 0.00634800

C 0.44780700 4.52849200 -0.03658300

C 1.60906700 3.75996500 -0.08872900

C 1.53213500 2.32412000 -0.09709600

C 0.30698500 1.65997100 -0.05486700

C -0.84889100 2.43986900 -0.00323400

C -2.22785700 2.03827100 0.04615800

C -3.01844100 3.22855000 0.08570100

C -2.13731400 4.36423500 0.06206800

C -4.41257100 3.39850000 0.13861000

C -4.92276800 4.68153900 0.16890900

C -4.05941700 5.79942200 0.14666700

C -2.68132800 5.65273200 0.09362000

C 3.00506900 4.10946100 -0.14072600

C 3.76989500 2.89114500 -0.17979100

C 2.86586100 1.78686200 -0.15217400

C -2.72928200 0.73753200 0.05281400

C -3.21890700 -0.38046600 0.06036300

C 3.67430000 5.33837300 -0.15685100

C 5.05953800 5.34848600 -0.21040300

C 5.80835200 4.15047200 -0.24914800

C 5.17445600 2.92427500 -0.23426900

C 3.20258100 0.43485000 -0.16783300

C 3.48463000 -0.75227200 -0.17990300

Si -4.09866000 -1.99593900 0.03741200

C -5.35627800 -1.94574800 1.46576100

C -4.83334400 -2.16985600 -1.71120400

C -2.82425000 -3.37273500 0.33538200

Si 3.83729100 -2.55525000 -0.13777300

C 5.72776300 -2.77680100 -0.21400600

C 3.05544900 -3.22009100 1.46451400

C 3.05277800 -3.35833300 -1.68552700

H 0.50085100 5.61233900 -0.02987500

H 0.25531800 0.57629900 -0.06338000

H -5.06466000 2.53284700 0.15353400

H -5.99381900 4.83914700 0.20996100

H -4.48831800 6.79477800 0.17141000

H -2.03881100 6.52558400 0.07670300

H 3.12115500 6.27014300 -0.12825700

H 5.58511900 6.29659800 -0.22314900

H 6.88966900 4.20233600 -0.29089600

H 5.73804100 1.99904000 -0.26573700

H -3.97595700 -1.97101400 -2.36732100

H -3.37977700 -4.31886900 0.29241500

H 5.97059900 -2.59218000 -1.26950400

H 3.18457000 -4.30989700 1.43504200

H 3.84381700 -3.31572000 -2.44609600

C -2.16926200 -3.26833400 1.72191700

H -1.43897300 -4.07066200 1.86764700

H -1.63838300 -2.31850600 1.83569800

H -2.90209800 -3.33941300 2.52925300

C -1.75864400 -3.39351300 -0.77207400

H -1.20513200 -2.45047000 -0.79602000

H -1.03422100 -4.19717500 -0.60300500

H -2.19208000 -3.54562800 -1.76356700

C -5.33908100 -3.58840500 -2.02335800

H -6.20010600 -3.85366900 -1.40462000

H -5.65658100 -3.66066000 -3.06917600

H -4.56947000 -4.34717900 -1.86115200

C -5.90544600 -1.11598200 -2.02871100

H -5.55914300 -0.10355600 -1.80649800

H -6.17949700 -1.15038200 -3.08857100

H -6.81784000 -1.29319900 -1.45274500

C -6.25527700 -3.19307400 1.50462400

H -5.67945400 -4.12247500 1.47604700

H -6.85805200 -3.20730100 2.41886500

H -6.94955000 -3.20700600 0.65989400

C 1.54832400 -2.91882900 1.51485100

H 1.10166200 -3.33093000 2.42565700

H 1.36565800 -1.84034600 1.51818900

H 1.00872600 -3.34133000 0.66585800

C 3.74803200 -2.69004700 2.73012400

H 3.72542200 -1.59675700 2.76376200

H 3.23963600 -3.05738900 3.62829300

H 4.79168400 -3.00445800 2.79171100

C 6.51904300 -1.75626000 0.61980000

H 6.35706800 -1.89648100 1.69105500

H 7.59379200 -1.86208300 0.43574200

H 6.23331100 -0.73063700 0.37701500

C 6.15705700 -4.21685600 0.11685500

H 7.24043800 -4.33343200 0.00674900

H 5.90547000 -4.48174300 1.14824600

H 5.68187800 -4.95099500 -0.53875600

C 2.69856400 -4.84004500 -1.46615900

H 2.34898600 -5.29355500 -2.39972000

H 3.55046000 -5.42773100 -1.11493300

H 1.89680400 -4.95293700 -0.73123800

C 1.84343100 -2.58409000 -2.23508500

H 2.09988700 -1.55016400 -2.47308400

H 1.46325900 -3.05986900 -3.14579300

H 1.02276900 -2.55941700 -1.51470200

C -6.19058300 -0.65628500 1.52904900

H -5.55578700 0.23239000 1.53579400

H -6.87135400 -0.57446100 0.67823700

H -6.80155900 -0.63948700 2.43808900

H -4.72501500 -1.96302100 2.36355200

**Compound 4d open-shell singlet**

C -0.76946700 3.86026200 0.00789800

C 0.45907000 4.51344300 -0.03656900

C 1.61473500 3.73894400 -0.09156200

C 1.53713300 2.30910500 -0.10129600

C 0.31170200 1.64643800 -0.05775500

C -0.84072200 2.42914000 -0.00320000

C -2.23209300 2.03699300 0.04862300

C -3.01815300 3.24494600 0.09145400

C -2.13188100 4.36578800 0.06749600

C -4.40347400 3.42870000 0.14716100

C -4.90219900 4.72453200 0.18006300

C -4.03344200 5.82729300 0.15740500

C -2.65091500 5.65703700 0.10106000

C 3.02247300 4.10021100 -0.14603400

C 3.78814000 2.89318800 -0.18759000

C 2.88104100 1.77453800 -0.15937600

C -2.74168200 0.74602900 0.05407100

C -3.23685200 -0.37215000 0.06052100

C 3.67237400 5.33114300 -0.16126600

C 5.06496400 5.35826000 -0.21740700

C 5.81495900 4.17184800 -0.25846800

C 5.18511100 2.93433200 -0.24395600

C 3.22039600 0.42948900 -0.17503800

C 3.50159800 -0.76028900 -0.18645800

Si -4.11242900 -1.98962500 0.03418200

C -5.36930400 -1.94692300 1.46332100

C -4.84723400 -2.16328800 -1.71426300

C -2.83286600 -3.36251000 0.32898900

Si 3.83880400 -2.56604000 -0.13568500

C 5.72743900 -2.80546700 -0.20642700

C 3.04800400 -3.21554500 1.46849000

C 3.04988300 -3.36886900 -1.68108100

H 0.51421000 5.59661600 -0.02879000

H 0.25794200 0.56352800 -0.06772900

H -5.06730200 2.57204400 0.16271700

H -5.97211700 4.88997100 0.22352600

H -4.44696100 6.82882000 0.18394700

H -1.99511800 6.52008700 0.08360700

H 3.10925500 6.25697800 -0.13031600

H 5.58000400 6.31185800 -0.22965200

H 6.89621100 4.22649500 -0.30191200

H 5.75709000 2.01434600 -0.27720700

H -3.99067900 -1.96053400 -2.37032100

H -3.38501200 -4.31055300 0.28419500

H 5.97428100 -2.63158800 -1.26282200

H 3.16974900 -4.30638600 1.44645900

H 3.84113400 -3.33350600 -2.44183100

C -2.17790600 -3.25855500 1.71557000

H -1.44497900 -4.05878900 1.85960900

H -1.65003300 -2.30723100 1.83085800

H -2.91030000 -3.33353100 2.52297400

C -1.76719700 -3.37726900 -0.77857700

H -1.21629000 -2.43264500 -0.80001000

H -1.04059800 -4.17941100 -0.61161500

H -2.20022900 -3.52807500 -1.77046100

C -5.34839800 -3.58290900 -2.02881300

H -6.20817500 -3.85217300 -1.41003100

H -5.66626900 -3.65444800 -3.07457200

H -4.57614600 -4.33936000 -1.86832500

C -5.92290300 -1.11248000 -2.03000200

H -5.58026300 -0.09928600 -1.80555500

H -6.19656500 -1.14562800 -3.09001600

H -6.83484300 -1.29402600 -1.45465400

C -6.26193300 -3.19877200 1.50273600

H -5.68134600 -4.12519000 1.47375400

H -6.86414200 -3.21618100 2.41730900

H -6.95660800 -3.21627600 0.65838800

C 1.54281400 -2.90367400 1.51330100

H 1.09176600 -3.30487400 2.42681700

H 1.36709500 -1.82406700 1.50674000

H 1.00204000 -3.33016300 0.66705600

C 3.74116000 -2.68205600 2.73239700

H 3.72550300 -1.58845200 2.75918400

H 3.22833500 -3.04040200 3.63168100

H 4.78263900 -3.00277100 2.79841500

C 6.52592600 -1.78523900 0.62092300

H 6.35939200 -1.91461900 1.69286600

H 7.60018500 -1.90283100 0.44111600

H 6.25063700 -0.75911300 0.36835100

C 6.14344100 -4.24644600 0.13711500

H 7.22594900 -4.37349000 0.02992500

H 5.88773100 -4.50049100 1.17020200

H 5.66289700 -4.98180500 -0.51317600

C 2.68662200 -4.84772600 -1.45710900

H 2.33352500 -5.30186200 -2.38903600

H 3.53507600 -5.43968000 -1.10471800

H 1.88471500 -4.95337300 -0.72127500

C 1.84516200 -2.58911000 -2.23317200

H 2.10756900 -1.55745300 -2.47442300

H 1.46239100 -3.06549300 -3.14248500

H 1.02448300 -2.55711500 -1.51311400

C -6.21012600 -0.66173900 1.52779000

H -5.57993000 0.23026300 1.53259400

H -6.89305100 -0.58392100 0.67833200

H -6.81931400 -0.64754600 2.43809000

H -4.73718000 -1.96124300 2.36059000

**Compound 4d open-shell triplet**

C -0.76783900 3.84747800 0.00760400

C 0.45880600 4.50237400 -0.03737000

C 1.61155900 3.72548600 -0.09402100

C 1.53722200 2.29054300 -0.10522500

C 0.30954900 1.62776100 -0.06122800

C -0.84419300 2.41161800 -0.00477000

C -2.23116200 2.03217000 0.04791500

C -3.02085500 3.25485600 0.09333400

C -2.13415600 4.36676700 0.06960200

C -4.39776600 3.44512600 0.15054400

C -4.89101200 4.75130600 0.18538600

C -4.02256100 5.84452100 0.16290600

C -2.63609500 5.65898700 0.10453300

C 3.02526500 4.09885800 -0.14913300

C 3.79097800 2.90010700 -0.19198600

C 2.87729900 1.76791800 -0.16407200

C -2.75105700 0.74730900 0.05283400

C -3.25400200 -0.36820900 0.05890300

C 3.65941000 5.33185100 -0.16303500

C 5.05770900 5.37256800 -0.22008200

C 5.80709700 4.19511800 -0.26233600

C 5.18019300 2.94722900 -0.24867900

C 3.22631100 0.42721400 -0.17994600

C 3.51476000 -0.76161800 -0.19126600

Si -4.12263100 -1.98860300 0.03275500

C -5.37788800 -1.95388600 1.46366700

C -4.85996000 -2.16704100 -1.71424700

C -2.83526500 -3.35502700 0.32506200

Si 3.84863400 -2.56720000 -0.13493700

C 5.73707900 -2.81176200 -0.20045400

C 3.05383500 -3.21121700 1.46969200

C 3.06101300 -3.37350300 -1.67922800

H 0.51439500 5.58515300 -0.02856500

H 0.25535400 0.54550400 -0.07247700

H -5.06906700 2.59436600 0.16631100

H -5.96062000 4.91929700 0.23026400

H -4.42681300 6.84949700 0.19073900

H -1.97188400 6.51571000 0.08696900

H 3.08816300 6.25277100 -0.13063700

H 5.56479900 6.33011700 -0.23147100

H 6.88839800 4.25121200 -0.30628300

H 5.75939600 2.03186300 -0.28297500

H -4.00573500 -1.95948900 -2.37184700

H -3.38248500 -4.30597900 0.28089000

H 5.98667600 -2.64580400 -1.25747700

H 3.17649400 -4.30205900 1.45249000

H 3.85196300 -3.33723900 -2.44028600

C -2.17844900 -3.24797500 1.71053000

H -1.44140800 -4.04467000 1.85324100

H -1.65500700 -2.29408200 1.82475300

H -2.90905600 -3.32649800 2.51924300

C -1.77099600 -3.36398100 -0.78399600

H -1.22407600 -2.41703300 -0.80509100

H -1.04086800 -4.16328600 -0.61878700

H -2.20470200 -3.51563600 -1.77547800

C -5.35379500 -3.58935900 -2.02805400

H -6.21085000 -3.86355100 -1.40764400

H -5.67326400 -3.66269400 -3.07322800

H -4.57697300 -4.34142900 -1.86905800

C -5.94199600 -1.12224400 -2.02813800

H -5.60450900 -0.10721600 -1.80406800

H -6.21734600 -1.15673500 -3.08770200

H -6.85194200 -1.30884500 -1.45121500

C -6.26397900 -3.21027100 1.50429200

H -5.67859000 -4.13362400 1.47404200

H -6.86463600 -3.23114500 2.41984400

H -6.95992500 -3.23120100 0.66105900

C 1.54831800 -2.90022700 1.50977000

H 1.09592700 -3.29592500 2.42505600

H 1.37154700 -1.82087800 1.49576100

H 1.00951200 -3.33276200 0.66531800

C 3.74363000 -2.67188400 2.73297300

H 3.72639800 -1.57820600 2.75522700

H 3.22941100 -3.02730900 3.63264300

H 4.78544400 -2.99088300 2.80244900

C 6.53563600 -1.78720300 0.62151000

H 6.36563500 -1.90818700 1.69392100

H 7.61018100 -1.90854100 0.44574200

H 6.26323300 -0.76241400 0.36052000

C 6.15003200 -4.25087200 0.15430400

H 7.23257300 -4.38048500 0.05028400

H 5.89175600 -4.49701600 1.18865300

H 5.66959900 -4.99016500 -0.49156500

C 2.70087700 -4.85287300 -1.45361500

H 2.34761300 -5.30858500 -2.38473200

H 3.55081800 -5.44290800 -1.10160300

H 1.89995700 -4.95937300 -0.71680600

C 1.85438500 -2.59702800 -2.23185900

H 2.11394200 -1.56458000 -2.47268500

H 1.47352500 -3.07442200 -3.14147500

H 1.03313700 -2.56731800 -1.51234900

C -6.22519600 -0.67305800 1.52981100

H -5.59941900 0.22209800 1.53287100

H -6.91033400 -0.59895400 0.68178500

H -6.83251100 -0.66166300 2.44143900

H -4.74428700 -1.96510800 2.35994600

**Compound 4e closed-shell siinglet**

C 1.18189000 4.83329800 -0.21170800

C -0.01980400 5.52953800 -0.32631100

C -1.20969000 4.80556100 -0.36910200

C -1.19080500 3.36957200 -0.29348200

C 0.00461800 2.66090200 -0.18335300

C 1.18809600 3.39661500 -0.14370700

C 2.54656700 2.94003600 -0.03511700

C 3.38574400 4.09606200 -0.02971500

C 2.55458700 5.26519800 -0.13980400

C 4.78375200 4.21163000 0.05901100

C 5.34727700 5.47205500 0.03792600

C 4.53363500 6.62202000 -0.07121900

C 3.15284400 6.52991400 -0.15941900

C -2.59034500 5.20436300 -0.47556000

C -3.40132800 4.01591800 -0.46051000

C -2.54158000 2.88141800 -0.34720300

C -3.21132100 6.45380600 -0.58019900

C -4.59419500 6.51217300 -0.66584100

C -5.38782800 5.34328300 -0.64911900

C -4.80197000 4.09704900 -0.54676000

C 2.96992400 1.61403900 0.03338800

C -2.93471100 1.54653900 -0.27952500

C 3.35204800 0.45589000 0.07710100

C -3.27889800 0.37815400 -0.20787100

Si 4.02798700 -1.25436600 0.09216300

Si -3.79783500 -1.37123900 0.01751800

C 5.82130700 -1.08995200 0.68912700

C 3.01796900 -2.28322100 1.32269900

C 3.93173900 -1.94243600 -1.67861300

C -2.38902400 -2.44139000 -0.66911500

C -4.00644900 -1.63133500 1.88563600

C -5.41795800 -1.65102300 -0.93881800

C 6.67204700 -0.18933400 -0.22748500

C 8.09168600 -0.00115500 0.32242500

C 8.78255800 -1.34912700 0.56004400

C 7.94376600 -2.25575200 1.46834500

C 6.52401000 -2.44284500 0.91683400

C 1.53380200 -2.42965700 0.93082900

C 0.76499500 -3.28584800 1.94383100

C 0.88447400 -2.71484200 3.35937900

C 2.35320500 -2.57279400 3.77168800

C 3.14300200 -1.73334700 2.75909000

C 2.72434100 -1.40893800 -2.47682100

C 2.68167600 -1.96872700 -3.90351800

C 2.72775600 -3.50060700 -3.91580100

C 3.94654300 -4.02455100 -3.14928400

C 3.97396200 -3.48569800 -1.71328100

C -5.07381800 -0.70145000 2.49788000

C -5.21964100 -0.91062900 4.01070700

C -3.87791200 -0.74575900 4.73202200

C -2.81562800 -1.67909800 4.14237200

C -2.66576700 -1.47218000 2.63037100

C -2.12828300 -2.16710700 -2.16386800

C -0.95789200 -2.99971500 -2.69926600

C -1.17207000 -4.49611300 -2.45115500

C -1.41783700 -4.78117500 -0.96555400

C -2.58755400 -3.94993700 -0.42039700

C -6.30802300 -0.39519900 -1.04688000

C -7.57360000 -0.66513300 -1.87012200

C -8.36668500 -1.85220900 -1.31244400

C -7.49226200 -3.10697300 -1.21534800

C -6.22789900 -2.84438200 -0.38700600

H -0.02875600 6.61336800 -0.38003300

H 0.01335700 1.57732000 -0.12987000

H 5.39699800 3.32204600 0.14539400

H 6.42237500 5.58750200 0.10553200

H 5.00413000 7.59862900 -0.08571900

H 2.54916600 7.42645500 -0.24193700

H -2.62368800 7.36459300 -0.59406600

H -5.08218900 7.47684000 -0.74731900

H -6.46533000 5.43298200 -0.71751400

H -5.39920800 3.19258900 -0.53095200

H 5.75068100 -0.58550600 1.66355100

H 3.46298300 -3.28929000 1.30913800

H 4.83864600 -1.57911100 -2.18309700

H -1.49040300 -2.12704700 -0.12006200

H -4.34594100 -2.66905400 2.01958000

H -5.10998500 -1.91210400 -1.96159800

H 6.73713000 -0.64201200 -1.22517000

H 6.18530900 0.78155600 -0.35925900

H 8.68410200 0.61235100 -0.36414400

H 8.03716100 0.54915400 1.27022400

H 8.93234800 -1.84968900 -0.40536300

H 9.77723000 -1.19666900 0.99029400

H 8.43260300 -3.22781600 1.59008000

H 7.88046400 -1.80709100 2.46771800

H 6.58431800 -2.98652500 -0.03427700

H 5.94039100 -3.07113200 1.59722600

H 1.07383600 -1.43486900 0.87557600

H 1.44087300 -2.86763000 -0.06607400

H -0.28527400 -3.36365800 1.65097100

H 1.16822000 -4.30652700 1.92869700

H 0.40485500 -1.72877600 3.38822900

H 0.34526200 -3.34546000 4.07291800

H 2.42724900 -2.12453000 4.76770700

H 2.80573800 -3.57031200 3.84063100

H 2.77101700 -0.70135200 2.77870500

H 4.19393400 -1.68877000 3.06009800

H 1.79693900 -1.67862200 -1.96044000

H 2.74800800 -0.31677200 -2.50165700

H 1.78273600 -1.61212300 -4.41631600

H 3.54009400 -1.57927300 -4.46552300

H 1.81598100 -3.89235600 -3.44903900

H 2.73642100 -3.87260300 -4.94506000

H 3.94818200 -5.11943800 -3.13868200

H 4.86224200 -3.71241600 -3.66720900

H 3.10990700 -3.88820600 -1.17155300

H 4.86173800 -3.85895000 -1.19421800

H -4.79202900 0.34062000 2.30378900

H -6.04093900 -0.85604900 2.01177600

H -5.95760000 -0.21045000 4.41544400

H -5.60769400 -1.92029700 4.19732100

H -3.53988800 0.29307600 4.62809200

H -3.99614400 -0.93164200 5.80409500

H -1.85268900 -1.52292000 4.63823500

H -3.10274700 -2.72098600 4.33447500

H -2.26977800 -0.46623900 2.44276400

H -1.92739500 -2.17327600 2.23605800

H -3.02788300 -2.41070000 -2.74326900

H -1.93595300 -1.10169300 -2.32162300

H -0.81199400 -2.80636600 -3.76675700

H -0.03891000 -2.68121000 -2.19604500

H -2.03798300 -4.83757200 -3.03285300

H -0.31051000 -5.06855700 -2.80937800

H -1.61090800 -5.84744900 -0.80917900

H -0.51179700 -4.53931400 -0.39855300

H -3.51029600 -4.28113900 -0.91282400

H -2.71903000 -4.14882200 0.64827200

H -6.59893900 -0.06240400 -0.04332700

H -5.73904600 0.42719200 -1.48802000

H -8.20147800 0.23140100 -1.89857000

H -7.28570800 -0.87991700 -2.90717200

H -8.73978000 -1.59707400 -0.31207300

H -9.24621600 -2.04883000 -1.93336300

H -8.06124700 -3.93467900 -0.77956300

H -7.19984800 -3.42234400 -2.22494900

H -6.52158200 -2.63774900 0.64895600

H -5.61221600 -3.74818600 -0.35583400

**Compound 4e open-shell singlet**

C 1.16299500 4.81495400 -0.20677300

C -0.03313200 5.51722400 -0.33091500

C -1.22032800 4.79123200 -0.38423200

C -1.20646100 3.36109900 -0.31045500

C -0.01447600 2.64960500 -0.19083900

C 1.16849400 3.38409700 -0.14028700

C 2.53704000 2.93160600 -0.02116200

C 3.37696400 4.10259200 -0.00781700

C 2.54535100 5.26011200 -0.12281300

C 4.76646400 4.22686300 0.09151200

C 5.32418600 5.49860000 0.07579700

C 4.50968900 6.63669600 -0.03798500

C 3.12355900 6.52638000 -0.13744000

C -2.60927600 5.20736300 -0.50365500

C -3.42562000 4.03304700 -0.49711300

C -2.56935000 2.88109300 -0.37634400

C -3.20522100 6.46084200 -0.61086400

C -4.59348600 6.54194100 -0.70935800

C -5.39283200 5.38749400 -0.70111600

C -4.81764100 4.12792300 -0.59481600

C 2.96557100 1.61360900 0.04579600

C -2.96907900 1.55455600 -0.30829900

C 3.35333700 0.45475500 0.08862800

C -3.31210900 0.38347200 -0.23402900

Si 4.03043300 -1.25505800 0.09059700

Si -3.79957600 -1.37103200 0.01980400

C 5.82710600 -1.09457500 0.67858300

C 3.02720800 -2.29175200 1.31995700

C 3.92539000 -1.93326700 -1.68343100

C -2.38356300 -2.42534900 -0.67706500

C -3.97338000 -1.60991100 1.89419900

C -5.42855500 -1.69481800 -0.90677200

C 6.67224000 -0.18586900 -0.23522600

C 8.09527000 -0.00247400 0.30752600

C 8.78762100 -1.35242500 0.52909200

C 7.95447300 -2.26691200 1.43469300

C 6.53138100 -2.44925200 0.89026800

C 1.53949700 -2.42898800 0.93797600

C 0.77478300 -3.29146600 1.94882000

C 0.90790600 -2.73419900 3.36865100

C 2.38031500 -2.60216100 3.77089700

C 3.16527200 -1.75576900 2.76035400

C 2.71495300 -1.39476800 -2.47357000

C 2.66715000 -1.94534200 -3.90368900

C 2.71299300 -3.47712300 -3.92608400

C 3.93361100 -4.00656200 -3.16627700

C 3.96571500 -3.47632100 -1.72717200

C -5.05360000 -0.69740600 2.51006900

C -5.17296100 -0.89066400 4.02728600

C -3.82542400 -0.68494400 4.72698900

C -2.74902400 -1.59922500 4.13300900

C -2.62617100 -1.40746900 2.61653400

C -2.14795300 -2.15823300 -2.17738400

C -0.97483900 -2.97920000 -2.72465600

C -1.16537000 -4.47645200 -2.46274100

C -1.38458600 -4.75383700 -0.97159200

C -2.55735900 -3.93470100 -0.41494400

C -6.34463700 -0.45835800 -1.01938200

C -7.61489400 -0.76457500 -1.82246200

C -8.37720000 -1.95932100 -1.23856400

C -7.47663200 -3.19497700 -1.13411300

C -6.20736700 -2.89514900 -0.32622600

H -0.03987600 6.60056400 -0.38352600

H -0.00803700 1.56657800 -0.13863500

H 5.38783000 3.34335100 0.18179400

H 6.39843800 5.61776700 0.15206000

H 4.96881700 7.61847500 -0.04834800

H 2.51021300 7.41605300 -0.22432200

H -2.60408800 7.36298800 -0.61764300

H -5.06634200 7.51361200 -0.79346300

H -6.46922900 5.48449800 -0.77854700

H -5.42692700 3.23148600 -0.58576500

H 5.76217100 -0.59863200 1.65776600

H 3.46843200 -3.29923500 1.29374700

H 4.83035100 -1.56808100 -2.19013300

H -1.48202900 -2.09431200 -0.14273900

H -4.28546400 -2.65349800 2.04789600

H -5.13091400 -1.96480800 -1.93033700

H 6.73120900 -0.62973200 -1.23724800

H 6.18468100 0.78611300 -0.35541800

H 8.68346200 0.61704600 -0.37729000

H 8.04665000 0.53949200 1.26045300

H 8.93130500 -1.84457500 -0.44155400

H 9.78498900 -1.20368500 0.95439400

H 8.44405700 -3.23996900 1.54499500

H 7.89733300 -1.82690700 2.43828200

H 6.58592100 -2.98452300 -0.06594000

H 5.95192900 -3.08358800 1.56860400

H 1.08315500 -1.43195500 0.89521200

H 1.43769100 -2.85732300 -0.06226900

H -0.27819300 -3.36207800 1.66366500

H 1.17328400 -4.31373300 1.92100500

H 0.43323300 -1.74628100 3.41025400

H 0.37125800 -3.36881500 4.08058100

H 2.46385400 -2.16408600 4.77070400

H 2.82949000 -3.60203900 3.82635600

H 2.79682600 -0.72280600 2.79269200

H 4.21863400 -1.71762100 3.05364300

H 1.78966600 -1.66812100 -1.95518600

H 2.73784800 -0.30247800 -2.49156800

H 1.76643300 -1.58542900 -4.41101400

H 3.52364000 -1.55225800 -4.46612100

H 1.80244300 -3.87165300 -3.45938800

H 2.71867200 -3.84242100 -4.95776900

H 3.93436900 -5.10150200 -3.16233800

H 4.84809100 -3.69204400 -3.68493200

H 3.10257800 -3.88075900 -1.18535200

H 4.85442000 -3.85384600 -1.21281700

H -4.79903000 0.34838900 2.29905500

H -6.02362600 -0.88050600 2.03996400

H -5.92158400 -0.20310700 4.43411400

H -5.53413700 -1.90680700 4.23185300

H -3.51424900 0.36041600 4.60603900

H -3.92380700 -0.86071400 5.80278000

H -1.78342200 -1.41314400 4.61306000

H -3.00715900 -2.64542900 4.34199900

H -2.25903000 -0.39418500 2.41095500

H -1.87572300 -2.09418400 2.21956100

H -3.05265400 -2.41737800 -2.74196800

H -1.97169100 -1.09159300 -2.34499400

H -0.84799100 -2.79180500 -3.79566000

H -0.05258400 -2.64477500 -2.23805700

H -2.03551000 -4.83344200 -3.02860600

H -0.30195900 -5.04021700 -2.83012300

H -1.56052700 -5.82146500 -0.80452500

H -0.47355500 -4.49536300 -0.42028800

H -3.48242700 -4.28214000 -0.89151000

H -2.67051200 -4.12763000 0.65693200

H -6.62920300 -0.11820600 -0.01649300

H -5.79798300 0.36935400 -1.47856900

H -8.26097000 0.11880200 -1.85507600

H -7.33580000 -0.98806000 -2.86008300

H -8.74367800 -1.69731700 -0.23752100

H -9.25979600 -2.18237900 -1.84606200

H -8.02359600 -4.02711000 -0.67898700

H -7.19060400 -3.51957900 -2.14261600

H -6.49175000 -2.67825200 0.71021300

H -5.57375100 -3.78618900 -0.28930000

**Compound 4e open-shell triplet**

C 1.15956900 4.80230700 -0.20847800

C -0.03436000 5.50668800 -0.33123700

C -1.21916100 4.77854100 -0.38361200

C -1.20931100 3.34352500 -0.31077200

C -0.01530300 2.63141600 -0.19227100

C 1.16947800 3.36665000 -0.14196500

C 2.53443100 2.92660300 -0.02552200

C 3.37913300 4.11196400 -0.01296400

C 2.54694000 5.26078100 -0.12604700

C 4.76069000 4.24271800 0.08433300

C 5.31383700 5.52497600 0.06808100

C 4.49941700 6.65372400 -0.04369200

C 3.10837300 6.52860300 -0.14170200

C -2.61343100 5.20776300 -0.50260500

C -3.43014500 4.04208500 -0.49620500

C -2.56861100 2.87592700 -0.37574600

C -3.19288400 6.46309500 -0.60764300

C -4.58629900 6.55884500 -0.70602700

C -5.38536900 5.41370500 -0.69812500

C -4.81430100 4.14355700 -0.59253500

C 2.97359700 1.61410700 0.04169100

C -2.97845300 1.55440100 -0.30823000

C 3.36998000 0.45741900 0.08617800

C -3.32887200 0.38458200 -0.23544500

Si 4.04178300 -1.25373000 0.09213700

Si -3.80999300 -1.37096100 0.01863100

C 5.83843800 -1.10038400 0.68238300

C 3.03230100 -2.28384000 1.32225200

C 3.93691700 -1.93683600 -1.68022400

C -2.38819300 -2.41920700 -0.67608400

C -3.98515800 -1.61211600 1.89275200

C -5.43619900 -1.70377200 -0.90975500

C 6.68824400 -0.19548400 -0.23084200

C 8.11156300 -0.01775200 0.31307300

C 8.79810200 -1.37046500 0.53590000

C 7.96034500 -2.28098000 1.44126200

C 6.53703100 -2.45771300 0.89557700

C 1.54486300 -2.41556700 0.93721500

C 0.77383900 -3.27163900 1.94866900

C 0.90568400 -2.71060700 3.36711300

C 2.37770400 -2.58402000 3.77264000

C 3.16921100 -1.74443500 2.76147000

C 2.73038800 -1.39477000 -2.47394500

C 2.68270800 -1.94861200 -3.90277900

C 2.72093000 -3.48064000 -3.92105500

C 3.93759900 -4.01410600 -3.15771900

C 3.96997600 -3.48016400 -1.71997800

C -5.06886600 -0.70327400 2.50786400

C -5.18887900 -0.89680900 4.02500900

C -3.84258500 -0.68663300 4.72581100

C -2.76284300 -1.59765200 4.13288100

C -2.63930400 -1.40574800 2.61649300

C -2.14919600 -2.14937600 -2.17534800

C -0.97088500 -2.96475100 -2.71990300

C -1.15617200 -4.46310700 -2.46043200

C -1.37890300 -4.74331700 -0.97033300

C -2.55660500 -3.92952900 -0.41620400

C -6.35823100 -0.47198500 -1.02436900

C -7.62530800 -0.78470800 -1.82996100

C -8.38285900 -1.98308300 -1.24722900

C -7.47631200 -3.21417000 -1.14010600

C -6.21016700 -2.90748800 -0.32991500

H -0.04120800 6.58968500 -0.38332800

H -0.00872800 1.54897000 -0.14087900

H 5.38974500 3.36460100 0.17340600

H 6.38806600 5.64637700 0.14318000

H 4.94969000 7.63928800 -0.05466700

H 2.48645200 7.41250700 -0.22782400

H -2.58328400 7.35967300 -0.61356600

H -5.05055700 7.53444800 -0.78895500

H -6.46177700 5.51283000 -0.77473000

H -5.43117300 3.25237700 -0.58417400

H 5.77432000 -0.60370500 1.66125500

H 3.46933400 -3.29323900 1.29970400

H 4.84447100 -1.57730800 -2.18642300

H -1.48945800 -2.08507900 -0.13898600

H -4.29411300 -2.65674200 2.04567200

H -5.13567300 -1.97278700 -1.93276400

H 6.74626300 -0.63985500 -1.23270700

H 6.20466800 0.77842400 -0.35168900

H 8.70301700 0.59900000 -0.37147100

H 8.06435500 0.52479700 1.26575300

H 8.94048200 -1.86371100 -0.43438700

H 9.79575900 -1.22569400 0.96192800

H 8.44585300 -3.25597300 1.55259300

H 7.90411400 -1.84013300 2.44454100

H 6.59031600 -2.99378800 -0.06025200

H 5.95426300 -3.08923900 1.57369800

H 1.09315100 -1.41664100 0.89042500

H 1.44351400 -2.84640700 -0.06199200

H -0.27866600 -3.33841900 1.66097300

H 1.16790000 -4.29575100 1.92506000

H 0.43520000 -1.72048200 3.40443000

H 0.36444800 -3.34070600 4.07960100

H 2.46076500 -2.14320200 4.77130300

H 2.82203700 -3.58582700 3.83238600

H 2.80558700 -0.70967100 2.78977500

H 4.22203000 -1.71046100 3.05731200

H 1.80285300 -1.66269200 -1.95675200

H 2.75807600 -0.30266100 -2.49434400

H 1.78474100 -1.58556800 -4.41276900

H 3.54226400 -1.56126900 -4.46456400

H 1.80761100 -3.86920100 -3.45476700

H 2.72647400 -3.84884200 -4.95172600

H 3.93289600 -5.10903900 -3.15087800

H 4.85449700 -3.70553600 -3.67570900

H 3.10407900 -3.87905800 -1.17848000

H 4.85602800 -3.86063600 -1.20321900

H -4.81754000 0.34334500 2.29700700

H -6.03792000 -0.88957500 2.03693900

H -5.94011800 -0.21170700 4.43122600

H -5.54685000 -1.91413300 4.22937100

H -3.53461400 0.35967600 4.60479100

H -3.94132100 -0.86242300 5.80158700

H -1.79816500 -1.40851300 4.61363600

H -3.01800100 -2.64459500 4.34188700

H -2.27458900 -0.39159600 2.41096700

H -1.88660600 -2.09052000 2.22043600

H -3.05107200 -2.41174200 -2.74302600

H -1.97690800 -1.08182900 -2.34100700

H -0.84141600 -2.77557500 -3.79029700

H -0.05154800 -2.62702200 -2.22996600

H -2.02315900 -4.82280300 -3.02944400

H -0.28943000 -5.02303200 -2.82584400

H -1.55112700 -5.81187600 -0.80523500

H -0.47059800 -4.48198800 -0.41592400

H -3.47887000 -4.28015300 -0.89589200

H -2.67210600 -4.12418000 0.65511900

H -6.64654500 -0.13328400 -0.02203700

H -5.81472100 0.35845500 -1.48239800

H -8.27581500 0.09537800 -1.86423200

H -7.34294000 -1.00716700 -2.86692200

H -8.75287700 -1.72244700 -0.24712300

H -9.26298300 -2.21088200 -1.85657800

H -8.02012500 -4.04871300 -0.68558600

H -7.18662700 -3.53798600 -2.14782300

H -6.49765800 -2.69119700 0.70580400

H -5.57223900 -3.79535100 -0.29102600

**Fluoereno[2.3-*b*]fluorenes**

**Compound 9c closed-shell singlet**

C -0.00148300 -1.06442000 0.00009600

C -0.00180900 -2.51329100 -0.00030700

C 1.23377600 -3.20073100 -0.00011700

C 2.42281300 -2.50599100 0.00023600

C 2.41731000 -1.05874200 0.00028100

C 1.22292900 -0.36214300 0.00038600

C 3.80432800 -2.92229200 0.00071700

C 4.62111300 -1.74149600 0.00097700

C 3.77180600 -0.58880300 0.00039300

C -1.22558700 -0.36157500 0.00012500

C -2.42026100 -1.05766600 -0.00045700

C -2.42642400 -2.50490200 -0.00092900

C -1.23768100 -3.20018000 -0.00080500

C -3.77455600 -0.58710300 -0.00071400

C -4.62437800 -1.73938800 -0.00124800

C -3.80811000 -2.92056700 -0.00141100

C 4.40847400 -4.18277800 0.00113300

C 5.79284800 -4.26408600 0.00185700

C 6.59511700 -3.10221300 0.00224500

C 6.02294900 -1.84484400 0.00181400

C -6.02626000 -1.84217800 -0.00159700

C -6.59897200 -3.09928500 -0.00212400

C -5.79720700 -4.26151600 -0.00230400

C -4.41281100 -4.18079700 -0.00194500

C 4.17808400 0.74496100 -0.00028000

C -4.17980900 0.74697400 -0.00043000

C -4.52633900 1.91752700 -0.00001700

Si -5.04013400 3.68054700 0.00181600

C 4.52668500 1.91490200 -0.00093700

Si 5.04589100 3.67638400 -0.00113900

H 1.22607300 -4.28617800 -0.00025400

H 1.21916000 0.72295800 0.00094400

H -1.22133500 0.72353100 0.00067900

H -1.23046700 -4.28563000 -0.00109800

H 3.80671800 -5.08450200 0.00094000

H 6.27214300 -5.23618800 0.00222200

H 7.67389900 -3.20469100 0.00299400

H 6.63297300 -0.94920100 0.00239000

H -6.63591900 -0.94629200 -0.00140700

H -7.67779900 -3.20129800 -0.00239100

H -6.27692900 -5.23340600 -0.00271900

H -3.81143800 -5.08277600 -0.00207400

C 6.55519100 3.85454900 1.10641900

H 7.38219800 3.23780500 0.74507600

H 6.89484400 4.89454400 1.13063900

H 6.32972100 3.54786200 2.13091700

C 5.46600900 4.18045500 -1.76424900

H 4.60190400 4.06334200 -2.42316000

H 5.78050500 5.22813200 -1.80001700

H 6.27873100 3.56909500 -2.16467200

C 3.61941900 4.71291200 0.65325700

H 2.73215600 4.60131000 0.02448100

H 3.35135400 4.41545500 1.67026900

H 3.88900300 5.77330300 0.67073000

C -3.49362300 4.74445200 0.11791000

H -2.93714400 4.52992200 1.03397400

H -2.82872300 4.56447600 -0.73090200

H -3.75499700 5.80698800 0.12250800

C -6.15809800 3.97840400 1.48480500

H -6.48592000 5.02206700 1.51633300

H -7.04832700 3.34582900 1.43866500

H -5.63770400 3.75813200 2.42029100

C -5.96660200 4.03897800 -1.59550400

H -6.85445500 3.40770700 -1.68468300

H -6.28983200 5.08411100 -1.62649500

H -5.33403200 3.85378400 -2.46730800

**Compound 9c open-shell singlet**

C -0.00038400 -1.03982600 0.00029000

C -0.00048100 -2.48294800 0.00037200

C -1.23731800 -3.17830000 0.00056500

C -2.42128300 -2.48478200 0.00076200

C -2.41838900 -1.04263800 0.00076200

C -1.23197100 -0.34099400 0.00042900

C -3.81332300 -2.91563200 0.00076300

C -4.63517700 -1.74790400 0.00078100

C -3.78773100 -0.57993000 0.00096300

C 1.23129400 -0.34114500 0.00008900

C 2.41761900 -1.04295300 0.00005300

C 2.42032000 -2.48509800 0.00000300

C 1.23626500 -3.17846200 0.00016700

C 3.78702300 -0.58043300 -0.00001100

C 4.63431200 -1.74852400 -0.00030900

C 3.81229900 -2.91613900 -0.00027400

C -4.39834200 -4.17758300 0.00057300

C -5.78890900 -4.27646600 0.00035700

C -6.59603700 -3.12801800 0.00028100

C -6.02979300 -1.86006800 0.00048800

C 6.02890900 -1.86089300 -0.00065000

C 6.59498100 -3.12891700 -0.00094300

C 5.78769500 -4.27725200 -0.00089100

C 4.39714300 -4.17817200 -0.00056100

C -4.20341000 0.74470600 0.00110100

C 4.20292300 0.74413400 0.00026400

C 4.56110500 1.91342800 0.00046300

Si 5.09037400 3.67273600 0.00032500

C -4.56139800 1.91406000 0.00085400

Si -5.08906300 3.67385600 -0.00095100

H -1.22592600 -4.26333200 0.00052500

H -1.23234900 0.74365100 0.00007000

H 1.23181600 0.74350300 -0.00013100

H 1.22473400 -4.26349200 0.00014900

H -3.78682200 -5.07275100 0.00054900

H -6.25585700 -5.25443900 0.00017900

H -7.67430900 -3.23593900 -0.00001300

H -6.64810900 -0.97010100 0.00023500

H 6.64735900 -0.97102000 -0.00076000

H 7.67323800 -3.23698600 -0.00124700

H 6.25450700 -5.25529000 -0.00113600

H 3.78549700 -5.07325300 -0.00055200

C -6.42278100 3.89588500 -1.30799500

H -7.28858400 3.26277100 -1.09733600

H -6.76340000 4.93543500 -1.33777100

H -6.04827700 3.63366200 -2.30071300

C -5.76443800 4.09946000 1.70213100

H -5.00413700 3.95563800 2.47399800

H -6.08945500 5.14392400 1.73657600

H -6.62175500 3.46989400 1.95377300

C -3.59006100 4.73734100 -0.39847100

H -2.80239200 4.59961500 0.34688100

H -3.17597400 4.48244700 -1.37738800

H -3.86091500 5.79745400 -0.41139300

C 3.55459900 4.74950100 -0.13277400

H 3.00482500 4.53749400 -1.05342700

H 2.88012300 4.57715200 0.70999900

H 3.82535000 5.80969700 -0.13732400

C 6.22503300 3.95570200 -1.47260000

H 6.56242200 4.99628500 -1.50408100

H 7.10913900 3.31543000 -1.41618300

H 5.71159800 3.73746600 -2.41241300

C 6.00411100 4.02351000 1.60642700

H 6.88559300 3.38471600 1.70470900

H 6.33596600 5.06582300 1.64025500

H 5.36131300 3.84436500 2.47200100

**Compound 9c open-shell triplet**

C -0.00048200 -1.01571500 0.00020800

C -0.00059500 -2.45995200 0.00041000

C -1.23658800 -3.16078900 0.00054800

C -2.41970100 -2.47014500 0.00058000

C -2.42129500 -1.02853700 0.00048300

C -1.23498900 -0.32180800 0.00019700

C -3.81613200 -2.91262500 0.00048400

C -4.64037500 -1.75215700 0.00036000

C -3.79072800 -0.57477100 0.00057000

C 1.23413100 -0.32198700 0.00002100

C 2.42032800 -1.02890700 0.00011500

C 2.41850600 -2.47051500 0.00021200

C 1.23529000 -3.16097800 0.00036300

C 3.78983200 -0.57536200 0.00008300

C 4.63929200 -1.75288800 -0.00005700

C 3.81486400 -2.91322100 0.00005800

C -4.38963900 -4.17522300 0.00030600

C -5.78405200 -4.28425300 -0.00004100

C -6.59295600 -3.14362800 -0.00026200

C -6.02981800 -1.86878100 -0.00006500

C 6.02871300 -1.86974600 -0.00033200

C 6.59164900 -3.14468100 -0.00047100

C 5.78256100 -4.28517300 -0.00033100

C 4.38816700 -4.17591300 -0.00007200

C -4.21620300 0.74508200 0.00071500

C 4.21558500 0.74440100 0.00022100

C 4.58586500 1.91053600 0.00026300

Si 5.13088400 3.66521900 -0.00006500

C -4.58619700 1.91130700 0.00049300

Si -5.12922300 3.66661400 -0.00056400

H -1.22146800 -4.24561400 0.00059100

H -1.23971200 0.76252900 -0.00026700

H 1.23901900 0.76235000 -0.00030600

H 1.22000600 -4.24580100 0.00044100

H -3.77263400 -5.06668000 0.00038800

H -6.24399100 -5.26543400 -0.00021300

H -7.67099000 -3.25414800 -0.00065700

H -6.65309000 -0.98226400 -0.00043800

H 6.65213700 -0.98333800 -0.00051700

H 7.66966600 -3.25537400 -0.00072000

H 6.24234000 -5.26642900 -0.00045500

H 3.77101600 -5.06726800 0.00000200

C -6.47359800 3.87425900 -1.29901300

H -7.33237100 3.23396600 -1.08140100

H -6.82352900 4.91072000 -1.32837800

H -6.10342700 3.61356400 -2.29375700

C -5.79707100 4.08798300 1.70652600

H -5.03039300 3.95180300 2.47344700

H -6.13103000 5.12958700 1.74193800

H -6.64712200 3.45118900 1.96453200

C -3.64248500 4.74263000 -0.40953000

H -2.84868100 4.61272800 0.33067300

H -3.23265500 4.48995700 -1.39079800

H -3.92280500 5.80028800 -0.42203700

C 3.60588100 4.75562900 -0.14330700

H 3.05902900 4.54678500 -1.06640200

H 2.92540200 4.59093000 0.69614200

H 3.88638500 5.81328800 -0.14839400

C 6.27610500 3.93448500 -1.46736300

H 6.62331700 4.97182200 -1.49905800

H 7.15390000 3.28614300 -1.40490900

H 5.76569600 3.71920700 -2.40950300

C 6.03916000 4.00975300 1.61047100

H 6.91430900 3.36319600 1.71436700

H 6.38017700 5.04909200 1.64463600

H 5.39008900 3.83768600 2.47279000

**Compound 9d closed-shell singlet**

C -0.00304300 2.32382500 -0.00045100

C -0.00315000 3.77265700 -0.02116000

C 1.23569000 4.45706300 -0.04426200

C 2.42389300 3.75828700 -0.04721300

C 2.41059600 2.31255800 -0.02499600

C 1.21708000 1.61860700 -0.00092700

C 3.81084500 4.16087900 -0.06876600

C 4.61885500 2.97170000 -0.06043800

C 3.75888900 1.82875200 -0.03332400

C -1.22336100 1.61871600 0.01761900

C -2.41625200 2.31403400 0.01648300

C -2.43020300 3.75958800 0.00095300

C -1.24168200 4.45789300 -0.01844800

C -3.76456700 1.82970900 0.02750800

C -4.62518700 2.97181200 0.02212000

C -3.81759300 4.16155600 0.00605200

C 4.42660700 5.41516100 -0.09386600

C 5.81182400 5.48431500 -0.11037800

C 6.60413500 4.31597800 -0.10234500

C 6.02131600 3.06358600 -0.07762500

C -6.02788700 3.06303300 0.02855800

C -6.61135600 4.31528600 0.01983000

C -5.81963900 5.48404400 0.00444000

C -4.43431900 5.41561700 -0.00258500

C 4.10203400 0.47766000 -0.01471800

C -4.09718400 0.47627200 0.03085300

C -4.26765700 -0.73253100 0.02261400

Si -4.27661800 -2.56877300 -0.00471700

C 4.30255700 -0.72645500 0.00411000

Si 4.42699400 -2.55893100 0.05962300

H 1.23080400 5.54246300 -0.05963400

H 1.21062500 0.53401300 0.01992800

H -1.21841300 0.53376400 0.03244400

H -1.23631700 5.54330700 -0.03269900

H 3.83295100 6.32221800 -0.10039100

H 6.29926700 6.45215000 -0.12984000

H 7.68367000 4.40904300 -0.11587300

H 6.62459400 2.16332000 -0.07180900

H -6.63054900 2.16238900 0.04058400

H -7.69098900 4.40802100 0.02482400

H -6.30775800 6.45171600 -0.00209200

H -3.84132500 6.32305400 -0.01457100

C 4.83577900 -3.15261200 -1.70151600

H 5.84085700 -2.74822300 -1.87960200

C 5.84010100 -3.01432400 1.24680500

H 5.87765900 -4.11125700 1.27796700

C 2.76530100 -3.15712500 0.77708300

H 2.65672500 -2.55357200 1.68776700

C -2.47447600 -3.06123600 -0.37843900

H -1.89045700 -2.60022900 0.42867600

C -5.46451300 -3.17411400 -1.36617000

H -4.86846700 -3.15586600 -2.28781300

C -4.78440200 -3.17315600 1.72448700

H -4.81895700 -4.26914800 1.67044000

C 3.91662600 -2.57081800 -2.78743300

H 3.85453700 -1.48219000 -2.72252300

H 4.28909600 -2.82852100 -3.78480500

H 2.90144100 -2.96723800 -2.70822500

C 4.92333800 -4.68418000 -1.80658900

H 5.29981100 -4.98440800 -2.79027600

H 5.58893100 -5.11567800 -1.05374600

H 3.93966600 -5.14589400 -1.68310300

C 7.19964200 -2.50550100 0.74105700

H 7.19493600 -1.41624100 0.63548800

H 7.99758700 -2.76475400 1.44515400

H 7.46508500 -2.93301000 -0.22901400

C 5.56315900 -2.50406700 2.67085800

H 5.47573000 -1.41355300 2.68369300

H 4.63884500 -2.91485900 3.08485400

H 6.37899000 -2.77907100 3.34788400

C 2.77434000 -4.63629200 1.19779000

H 1.83675400 -4.89986400 1.69924700

H 2.87641900 -5.29825400 0.33384300

H 3.59091500 -4.86494100 1.88702300

C 1.55650600 -2.85020000 -0.12058400

H 1.54708500 -3.49315800 -1.00468000

H 0.61840400 -3.02552400 0.41667400

H 1.55506700 -1.81390900 -0.46787600

C -2.24158400 -4.57942800 -0.32343900

H -1.17861400 -4.81373100 -0.44397000

H -2.57037500 -5.01555200 0.62389900

H -2.77728500 -5.09089400 -1.12869100

C -1.97427000 -2.47333500 -1.70712700

H -2.10763300 -1.38945400 -1.74639000

H -0.91061700 -2.68814500 -1.85218200

H -2.51094900 -2.90335900 -2.55858100

C -5.92108000 -4.62541100 -1.13312100

H -6.52549900 -4.97946500 -1.97501500

H -5.08075700 -5.31456800 -1.01886100

H -6.53801500 -4.70778300 -0.23373900

C -6.67084900 -2.24561200 -1.58342100

H -6.35690800 -1.22376900 -1.80537400

H -7.28569900 -2.60221500 -2.41708900

H -7.31212100 -2.20649300 -0.69840300

C -3.74147400 -2.77833200 2.78326000

H -3.62886100 -1.69113500 2.83544700

H -4.04641400 -3.12629100 3.77605100

H -2.75771800 -3.20512600 2.57230200

C -6.18083400 -2.66890400 2.12403500

H -6.95641100 -3.01338900 1.43616100

H -6.44836500 -3.02114400 3.12616700

H -6.21154200 -1.57545200 2.13863500

**Compound 9d open-shell singlet**

C -0.00646400 2.30336200 0.00113000

C -0.00732700 3.74653200 -0.02021800

C 1.23228900 4.43957200 -0.04383600

C 2.41599600 3.74269800 -0.04649700

C 2.40623700 2.30214000 -0.02349700

C 1.22119700 1.60242000 0.00109500

C 3.81341100 4.15998100 -0.06882200

C 4.62710200 2.98386100 -0.06009200

C 3.76963700 1.82584000 -0.03206100

C -1.23349900 1.60127900 0.01969500

C -2.41870800 2.30119100 0.01824300

C -2.43066800 3.74156800 0.00166100

C -1.24747900 4.43917500 -0.01798600

C -3.78151800 1.82290000 0.02900500

C -4.64103000 2.97908000 0.02291500

C -3.82912900 4.15667300 0.00639000

C 4.40981200 5.41609900 -0.09458400

C 5.80126200 5.50311700 -0.11164500

C 6.59903100 4.34828900 -0.10324400

C 6.02258500 3.08520700 -0.07758900

C -6.03684300 3.07813900 0.02914900

C -6.61546000 4.34044700 0.01970200

C -5.81963800 5.49656400 0.00387000

C -4.42791100 5.41185100 -0.00301900

C 4.12026700 0.48267500 -0.01335200

C -4.11946800 0.47671100 0.03156100

C -4.28983100 -0.73417600 0.02261500

Si -4.28859000 -2.57150000 -0.00445300

C 4.32425700 -0.72297400 0.00562400

Si 4.44537400 -2.55671300 0.05960800

H 1.22307100 5.52455800 -0.05975700

H 1.21959600 0.51832100 0.02303600

H -1.23231500 0.51679700 0.03500300

H -1.23887900 5.52418400 -0.03286000

H 3.80608700 6.31651900 -0.10140600

H 6.27614400 6.47704600 -0.13172400

H 7.67805900 4.44722400 -0.11702200

H 6.63450400 2.19077300 -0.07141500

H -6.64711600 2.18262000 0.04149000

H -7.69470500 4.43778600 0.02458600

H -6.29629900 6.46980800 -0.00324300

H -3.82589300 6.31335300 -0.01553500

C 4.85165700 -3.14779600 -1.70264400

H 5.85988300 -2.75068300 -1.87923700

C 5.85814500 -3.01411200 1.24615200

H 5.89284800 -4.11108900 1.27861500

C 2.78284900 -3.15199300 0.77693900

H 2.67256500 -2.54521800 1.68526200

C -2.48179300 -3.05384000 -0.36779700

H -1.90456600 -2.58980200 0.44240100

C -5.46524000 -3.18234800 -1.37292800

H -4.86381800 -3.16153100 -2.29100000

C -4.80378500 -3.17559000 1.72234300

H -4.83187400 -4.27182800 1.66978000

C 3.93744500 -2.55678700 -2.78785500

H 3.88403600 -1.46779400 -2.72164400

H 4.30813000 -2.81609900 -3.78546700

H 2.91901000 -2.94480500 -2.70943500

C 4.92766900 -4.67980900 -1.81105500

H 5.30261600 -4.98068900 -2.79510000

H 5.58934500 -5.11808200 -1.05864000

H 3.94035800 -5.13419100 -1.68936700

C 7.21887400 -2.50950300 0.73938200

H 7.21708200 -1.42039100 0.63212900

H 8.01625000 -2.76974500 1.44374700

H 7.48305700 -2.93919800 -0.23005700

C 5.58300900 -2.50150100 2.66977200

H 5.49954400 -1.41065100 2.68175400

H 4.65724700 -2.90850500 3.08422100

H 6.39790100 -2.77870000 3.34701300

C 2.79179100 -4.62972000 1.20286200

H 1.85294800 -4.89189100 1.70266300

H 2.89652600 -5.29472600 0.34157200

H 3.60652900 -4.85554500 1.89518900

C 1.57542800 -2.84852400 -0.12389300

H 1.56818700 -3.49420700 -1.00609000

H 0.63670700 -3.02322300 0.41250400

H 1.57317100 -1.81316600 -0.47360300

C -2.24196800 -4.57097300 -0.31138100

H -1.17715400 -4.79988300 -0.42574000

H -2.57405700 -5.00881600 0.63403000

H -2.77034100 -5.08506600 -1.11980900

C -1.97702600 -2.46353800 -1.69374400

H -2.11257200 -1.38001300 -1.73274400

H -0.91202700 -2.67513600 -1.83335000

H -2.50804600 -2.89474700 -2.54817400

C -5.91592700 -4.63586900 -1.14208200

H -6.51345300 -4.99320500 -1.98748100

H -5.07284500 -5.32072200 -1.02252400

H -6.53784600 -4.72103500 -0.24640700

C -6.67499700 -2.26008600 -1.59775900

H -6.36521200 -1.23682200 -1.81906500

H -7.28331800 -2.62056000 -2.43453300

H -7.32142500 -2.22336800 -0.71641000

C -3.76943400 -2.77351800 2.78686900

H -3.66294800 -1.68569400 2.83861900

H -4.07842000 -3.12203700 3.77819600

H -2.78207600 -3.19497700 2.58229500

C -6.20553400 -2.67882200 2.11268900

H -6.97499900 -3.02886400 1.42076900

H -6.47695000 -3.03110300 3.11374600

H -6.24286400 -1.58555000 2.12556100

**Compound 9d open-shell triplet**

C -0.01059000 2.28310500 0.00302800

C -0.01249400 3.72724100 -0.01920700

C 1.22591700 4.42656400 -0.04293400

C 2.40916900 3.73319600 -0.04496000

C 2.40466100 2.29334800 -0.02113200

C 1.22047400 1.58783900 0.00388800

C 3.81109800 4.16253200 -0.06769700

C 4.62778000 2.99395700 -0.05842100

C 3.76877700 1.82638500 -0.02967200

C -1.24000500 1.58495000 0.02156100

C -2.42537900 2.28899900 0.01914800

C -2.43419200 3.72865000 0.00157800

C -1.25247300 4.42443000 -0.01796000

C -3.78820100 1.81802500 0.02886700

C -4.65103300 2.98244900 0.02150800

C -3.83788500 4.15371400 0.00502200

C 4.39541700 5.41959900 -0.09405200

C 5.79080300 5.51739600 -0.11134300

C 6.59093800 4.37076700 -0.10242600

C 6.01811900 3.10051600 -0.07600400

C -6.04185400 3.08466400 0.02669800

C -6.61877100 4.35330800 0.01615800

C -5.82229400 5.50239900 0.00035100

C -4.42644300 5.40897500 -0.00550700

C 4.12771200 0.48712700 -0.01119500

C -4.13184400 0.47496800 0.03132100

C -4.30537100 -0.73608600 0.02240000

Si -4.29993800 -2.57367600 -0.00336900

C 4.33867500 -0.71795400 0.00736600

Si 4.46359200 -2.55172700 0.05893200

H 1.21232400 5.51133400 -0.05949600

H 1.22386200 0.50408600 0.02687900

H -1.24230700 0.50079500 0.03751700

H -1.24102800 5.50924100 -0.03360600

H 3.78576000 6.31606900 -0.10130300

H 6.25822600 6.49480800 -0.13193800

H 7.66970100 4.47278500 -0.11627600

H 6.63546700 2.20981600 -0.06942700

H -6.65625000 2.19196200 0.03900500

H -7.69791000 4.45210700 0.02025000

H -6.29294700 6.47844700 -0.00763300

H -3.81981800 6.30744100 -0.01812400

C 4.87647800 -3.13851300 -1.70324000

H 5.88434800 -2.73895000 -1.87627700

C 5.87406700 -3.00768200 1.24877000

H 5.91094700 -4.10462700 1.27979000

C 2.80031200 -3.15175000 0.77036700

H 2.68574000 -2.54626100 1.67901000

C -2.49056500 -3.05212600 -0.35884200

H -1.91754000 -2.58548900 0.45282300

C -5.46964100 -3.18791800 -1.37626400

H -4.86446700 -3.16636000 -2.29183600

C -4.82131400 -3.17679800 1.72190400

H -4.84647600 -4.27315800 1.67038600

C 3.96397100 -2.54778800 -2.79003600

H 3.90797200 -1.45901700 -2.72241700

H 4.33795000 -2.80486000 -3.78700200

H 2.94615100 -2.93799100 -2.71491600

C 4.95605400 -4.67019300 -1.81371900

H 5.33411500 -4.96879300 -2.79726700

H 5.61677000 -5.10816400 -1.06028000

H 3.96940500 -5.12680300 -1.69516400

C 7.23516900 -2.49956800 0.74655600

H 7.23146500 -1.41030900 0.64081100

H 8.03108400 -2.75915400 1.45281600

H 7.50296700 -2.92736500 -0.22273000

C 5.59384900 -2.49768300 2.67234900

H 5.50841100 -1.40699600 2.68576800

H 4.66760200 -2.90691300 3.08347900

H 6.40725800 -2.77443000 3.35155700

C 2.81186500 -4.62994600 1.19468500

H 1.87217100 -4.89499800 1.69135000

H 2.92083100 -5.29374000 0.33297200

H 3.62506100 -4.85453000 1.88923900

C 1.59470500 -2.85025600 -0.13366300

H 1.59165200 -3.49500000 -1.01660500

H 0.65500800 -3.02805600 0.40005600

H 1.59050700 -1.81445600 -0.48183600

C -2.24763300 -4.56868400 -0.29914300

H -1.18183300 -4.79524400 -0.40902800

H -2.58238200 -5.00593500 0.64561300

H -2.77157500 -5.08523000 -1.10890300

C -1.98181700 -2.46279900 -1.68373300

H -2.11821100 -1.37950200 -1.72444700

H -0.91602000 -2.67321300 -1.81897400

H -2.50906000 -2.89587000 -2.53958500

C -5.91791200 -4.64232200 -1.14627400

H -6.51107300 -5.00162400 -1.99391200

H -5.07373000 -5.32509900 -1.02274100

H -6.54335400 -4.72833400 -0.25313700

C -6.68059200 -2.26862500 -1.60671500

H -6.37232700 -1.24481200 -1.82764100

H -7.28469300 -2.63117200 -2.44565000

H -7.33066400 -2.23267500 -0.72802300

C -3.79251200 -2.77106400 2.79041400

H -3.68891300 -1.68294800 2.84166000

H -4.10481200 -3.11953100 3.78072100

H -2.80322700 -3.19011400 2.59035700

C -6.22598500 -2.68313200 2.10560500

H -6.99161600 -3.03604600 1.41089200

H -6.50073300 -3.03483400 3.10595700

H -6.26622200 -1.58994400 2.11695300

**Compound 9e closed-shell singlet**

C 0.10216000 3.42998800 0.09389500

C 0.12705800 4.87869500 0.07946300

C 1.37392000 5.54137100 0.19330400

C 2.54605300 4.82097800 0.29525900

C 2.50444100 3.37673400 0.28108800

C 1.30307300 2.70348600 0.19885800

C 3.93998500 5.19088900 0.39246200

C 4.72217100 3.98280200 0.41842600

C 3.83918300 2.86108300 0.34326800

C -1.12072100 2.74183500 -0.01934100

C -2.29363800 3.45383200 -0.16008500

C -2.28704300 4.89859600 -0.18709100

C -1.09479200 5.58123100 -0.06227900

C -3.63776900 2.98078100 -0.30014000

C -4.47947200 4.12993500 -0.42149200

C -3.66191300 5.31261200 -0.35262200

C 4.58199300 6.43065700 0.45028300

C 5.96632700 6.46925300 0.53074600

C 6.73245600 5.28400500 0.55349500

C 6.12420200 4.04467100 0.49707400

C -5.87196600 4.23603900 -0.58088700

C -6.43650300 5.49387800 -0.67098700

C -5.63571400 6.65434400 -0.60434800

C -4.26001400 6.57203600 -0.44642400

C 4.10705100 1.49512800 0.26147200

C -3.95293900 1.62302900 -0.27123900

C -4.04484200 0.40782800 -0.19425900

Si -3.89569300 -1.38448800 0.19964200

C 4.14174700 0.28192400 0.12682400

Si 3.82242800 -1.49971800 -0.21177400

H 1.38904300 6.62681600 0.18622500

H 1.27627600 1.61972600 0.20619500

H -1.13268000 1.65740500 -0.00510000

H -1.07296200 6.66649800 -0.07563800

H 4.00888300 7.35070500 0.43236000

H 6.47330700 7.42612000 0.57639600

H 7.81205300 5.35323600 0.61624200

H 6.70796500 3.13171900 0.51429800

H -6.48232200 3.34194000 -0.63167500

H -7.50808900 5.59717100 -0.79471200

H -6.10873900 7.62670900 -0.67825600

H -3.65987400 7.47349600 -0.39678700

C 5.13269800 -2.11868500 -1.43999800

C 6.51570300 -1.45976500 -1.25668800

C 5.27661400 -3.65453100 -1.47688800

H 4.75374300 -1.80173100 -2.42397000

C 7.50505900 -1.91195500 -2.33794000

H 6.91749500 -1.72392500 -0.27090800

H 6.41691900 -0.37146700 -1.26644800

C 6.26061600 -4.10437900 -2.56445800

H 5.64414200 -4.00379000 -0.50492900

H 4.30641800 -4.13499300 -1.63119500

C 7.62992100 -3.43903700 -2.38679700

H 8.48554500 -1.45666400 -2.16452600

H 7.15740700 -1.54745400 -3.31298800

H 6.36312100 -5.19441100 -2.55127000

H 5.85299700 -3.83786100 -3.54787800

H 8.30694900 -3.74082700 -3.19203000

H 8.08001700 -3.79239900 -1.45001400

C 3.83041900 -2.33450400 1.49653400

C 3.33171600 -3.79208800 1.55778500

C 5.19938600 -2.20425700 2.19642300

H 3.11572300 -1.72503800 2.07160100

C 3.28850200 -4.31280000 3.00071500

H 3.99270800 -4.43580500 0.96758600

H 2.33780500 -3.87629000 1.11552500

C 5.15330800 -2.72710100 3.63758200

H 5.94818300 -2.77784800 1.63603400

H 5.53108500 -1.16197700 2.18480300

C 4.64883500 -4.17336900 3.69227700

H 2.96131400 -5.35770200 3.01163400

H 2.53815000 -3.74285700 3.56226700

H 6.14422400 -2.65235300 4.09705700

H 4.48392200 -2.08771500 4.22674100

H 4.58509300 -4.51607000 4.72974400

H 5.37512800 -4.82597800 3.19059800

C 2.08068800 -1.52265400 -0.99020800

C 1.87375200 -0.41550700 -2.04520100

C 1.65609300 -2.88012700 -1.58958800

H 1.40251200 -1.30140300 -0.15258600

C 0.42091100 -0.36844400 -2.53470600

H 2.53331000 -0.60571700 -2.90204200

H 2.16782500 0.55692700 -1.64683700

C 0.20467400 -2.84925300 -2.08994300

H 2.31780100 -3.12027500 -2.43094200

H 1.77428700 -3.68834400 -0.86441100

C -0.02533200 -1.72081000 -3.10003500

H 0.30016500 0.41757400 -3.28646200

H -0.23125100 -0.09676900 -1.69436800

H -0.06238900 -3.81514600 -2.53141200

H -0.45986900 -2.70426200 -1.23149000

H -1.07912300 -1.68247500 -3.39301400

H 0.54581700 -1.93312300 -4.01263600

C -2.20946500 -1.49774200 1.07679300

C -2.12290700 -0.61262800 2.33487100

C -1.70942400 -2.92372900 1.38364000

H -1.51775800 -1.06976000 0.33366900

C -0.70295700 -0.59447800 2.91478200

H -2.81492700 -0.99505400 3.09644500

H -2.44769000 0.40556800 2.10391700

C -0.29643400 -2.91179300 1.98145000

H -2.38990600 -3.41016400 2.09061700

H -1.71593800 -3.53669200 0.47823200

C -0.20449700 -2.01178900 3.21776800

H -0.67055700 0.02165200 3.81871800

H -0.02766000 -0.12046000 2.19028400

H 0.01601800 -3.93110500 2.22962600

H 0.40362500 -2.54615300 1.22355300

H 0.82553500 -1.98238100 3.58638400

H -0.81365900 -2.44130400 4.02333500

C -5.38076100 -1.77564400 1.31520700

C -6.71436400 -1.62559100 0.55298800

C -5.32785400 -3.12198500 2.06433600

H -5.35235900 -0.98056100 2.07527000

C -7.92446300 -1.81361500 1.47591700

H -6.76210300 -2.37498400 -0.24657700

H -6.76068700 -0.64705700 0.06575600

C -6.54585800 -3.30812000 2.97982900

H -5.29278300 -3.94806500 1.34539200

H -4.41327900 -3.18916800 2.65884700

C -7.86158000 -3.15833500 2.20852400

H -8.85246900 -1.73702000 0.90000100

H -7.94208800 -1.00062200 2.21254300

H -6.50077300 -4.28670000 3.46881600

H -6.50945800 -2.55536800 3.77750400

H -8.71376600 -3.26349200 2.88711300

H -7.94278700 -3.97094200 1.47487000

C -3.81154600 -2.35676100 -1.43143100

C -4.13572400 -3.85879500 -1.29545200

C -4.63722100 -1.73665400 -2.57735300

H -2.75392800 -2.28015800 -1.72405800

C -3.90100600 -4.60462500 -2.61537200

H -5.18605100 -3.97650200 -1.00406300

H -3.54269000 -4.31816200 -0.49976900

C -4.41416700 -2.48304800 -3.89859100

H -5.70289500 -1.77133600 -2.32063400

H -4.38415300 -0.67966900 -2.69305200

C -4.70860400 -3.98100500 -3.75915800

H -4.15852000 -5.66269700 -2.50134700

H -2.83230200 -4.56426400 -2.86137700

H -5.03533200 -2.04620200 -4.68716200

H -3.37067000 -2.34926500 -4.21099300

H -4.49896100 -4.49984100 -4.69976700

H -5.77870500 -4.11879900 -3.55704000

**Compound 9e open-shell singlet**

C 0.09764500 3.41042600 0.09132500

C 0.12177900 4.85351800 0.07559300

C 1.36973600 5.52483800 0.18744200

C 2.53748600 4.80635200 0.28807400

C 2.49919500 3.36729600 0.27836400

C 1.30606200 2.68815500 0.19791100

C 3.94230500 5.19057100 0.38515000

C 4.73023500 3.99522700 0.41325800

C 3.84941500 2.85864900 0.33945000

C -1.13213600 2.72543200 -0.02040300

C -2.29745100 3.44184000 -0.15969500

C -2.28895700 4.88127100 -0.18624800

C -1.10171800 5.56339800 -0.06426400

C -3.65640700 2.97493900 -0.29920200

C -4.49673200 4.13798600 -0.41954400

C -3.67485700 5.30852400 -0.35105000

C 4.56521100 6.43264700 0.43997900

C 5.95608100 6.48886400 0.52161100

C 6.72778700 5.31682300 0.54672800

C 6.12558800 4.06670800 0.49193600

C -5.88259000 4.25206900 -0.57656200

C -6.44209000 5.51991300 -0.66536600

C -5.63716100 6.66775900 -0.59886100

C -4.25517100 6.56901700 -0.44176700

C 4.12394500 1.50022700 0.25390000

C -3.97633900 1.62436900 -0.26956800

C -4.06640600 0.40690200 -0.19239700

Si -3.90410200 -1.38574900 0.20028900

C 4.15937300 0.28514000 0.11723500

Si 3.83229800 -1.49774200 -0.21376600

H 1.38059300 6.60994900 0.17944600

H 1.28436100 1.60473300 0.20514600

H -1.14805500 1.64153300 -0.00541200

H -1.07668600 6.64821100 -0.07815500

H 3.98205500 7.34633600 0.41933900

H 6.45081900 7.45207700 0.56552000

H 7.80699800 5.39190800 0.60971200

H 6.71799200 3.15936600 0.51076800

H -6.50059500 3.36324300 -0.62755200

H -7.51338100 5.62796100 -0.78771300

H -6.09881700 7.64559900 -0.67081100

H -3.64599400 7.46439000 -0.39149000

C 5.14078500 -2.12874900 -1.43747200

C 6.52588600 -1.47299100 -1.25852600

C 5.27978400 -3.66532000 -1.46316500

H 4.76298700 -1.81779600 -2.42378400

C 7.51418100 -1.93624500 -2.33605500

H 6.92644700 -1.73115100 -0.27066000

H 6.43086200 -0.38444100 -1.27629800

C 6.26275000 -4.12623400 -2.54700500

H 5.64575800 -4.00869600 -0.48852500

H 4.30808500 -4.14372600 -1.61435400

C 7.63413100 -3.46402700 -2.37364700

H 8.49605500 -1.48284400 -2.16557700

H 7.16806400 -1.57779000 -3.31388200

H 6.36175100 -5.21645800 -2.52581400

H 5.85636500 -3.86560700 -3.53250800

H 8.31049700 -3.77387800 -3.17636500

H 8.08272200 -3.81194900 -1.43411100

C 3.83762900 -2.31977900 1.50045100

C 3.33214000 -3.77458600 1.57216400

C 5.20743200 -2.19114000 2.19908000

H 3.12585900 -1.70309300 2.07145200

C 3.28663300 -4.28465700 3.01878400

H 3.99008300 -4.42559200 0.98652100

H 2.33783200 -3.85710800 1.13055400

C 5.15912800 -2.70335800 3.64397100

H 5.95327600 -2.77236500 1.64264000

H 5.54442300 -1.15065900 2.18009200

C 4.64771000 -4.14675200 3.70919300

H 2.95447900 -5.32787800 3.03726800

H 2.53904200 -3.70712800 3.57624600

H 6.15049200 -2.63007700 4.10271800

H 4.49294700 -2.05651800 4.22859000

H 4.58246200 -4.48156500 4.74913500

H 5.37080300 -4.80647300 3.21220900

C 2.09104700 -1.51762700 -0.99239500

C 1.88947100 -0.41697900 -2.05533700

C 1.66165000 -2.87792400 -1.58206800

H 1.41344200 -1.28747800 -0.15674300

C 0.43758700 -0.36883000 -2.54783700

H 2.55027200 -0.61499900 -2.90955100

H 2.18366600 0.55743900 -1.66247100

C 0.21084900 -2.84493400 -2.08401800

H 2.32299800 -3.12698500 -2.42113800

H 1.77596100 -3.68123100 -0.85081000

C -0.01301400 -1.72373600 -3.10342500

H 0.32149000 0.41185100 -3.30580500

H -0.21442700 -0.08716700 -1.71086400

H -0.05987000 -3.81336200 -2.51769400

H -0.45386200 -2.68987200 -1.22743200

H -1.06622000 -1.68367900 -3.39842200

H 0.55869200 -1.94578900 -4.01339600

C -2.21826000 -1.48719600 1.07895500

C -2.13923200 -0.60335900 2.33850200

C -1.70885600 -2.91025800 1.38422200

H -1.52886300 -1.05320800 0.33722600

C -0.72001900 -0.57665000 2.91994700

H -2.82979600 -0.99149900 3.09857000

H -2.46915400 0.41324600 2.10848900

C -0.29638000 -2.88934100 1.98292800

H -2.38632400 -3.40218400 2.09031100

H -1.71071300 -3.52213300 0.47807100

C -0.21196500 -1.99096000 3.22094300

H -0.69317000 0.03796200 3.82508200

H -0.04776300 -0.09600200 2.19724200

H 0.02302000 -3.90691600 2.22941900

H 0.40152700 -2.51722200 1.22613200

H 0.81751900 -1.95499400 3.59057200

H -0.81875500 -2.42645700 4.02512500

C -5.38797600 -1.78887600 1.31277800

C -6.72157200 -1.64756800 0.54882500

C -5.32618900 -3.13596500 2.05995700

H -5.36654500 -0.99484200 2.07421700

C -7.93163000 -1.84616300 1.46956800

H -6.76247200 -2.39598900 -0.25201800

H -6.77456700 -0.66864100 0.06304600

C -6.54421500 -3.33277900 2.97314100

H -5.28371500 -3.96059600 1.33971900

H -4.41210200 -3.19717200 2.65583100

C -7.85981000 -3.19161000 2.20000800

H -8.85928600 -1.77552100 0.89233600

H -7.95647100 -1.03458500 2.20755000

H -6.49253100 -4.31180700 3.46055600

H -6.51474900 -2.58113900 3.77214900

H -8.71224700 -3.30429300 2.87706600

H -7.93377200 -4.00355900 1.46486400

C -3.81094400 -2.35224300 -1.43349200

C -4.12338200 -3.85715700 -1.30146900

C -4.64051200 -1.73579900 -2.57862700

H -2.75369100 -2.26655900 -1.72500800

C -3.88183800 -4.59786300 -2.62301300

H -5.17294000 -3.98383600 -1.01106000

H -3.52722800 -4.31375200 -0.50653400

C -4.41054600 -2.47726200 -3.90147300

H -5.70609300 -1.77926600 -2.32290600

H -4.39541800 -0.67668200 -2.69206600

C -4.69342400 -3.97778600 -3.76589500

H -4.13114600 -5.65818600 -2.51175500

H -2.81331100 -4.54851300 -2.86806400

H -5.03443800 -2.04333900 -4.68949800

H -3.36787200 -2.33454400 -4.21259500

H -4.47897400 -4.49263200 -4.70760600

H -5.76257900 -4.12445100 -3.56499600

**Compound 9e open-shell triplet**

C 0.09541400 3.39130000 0.09289400

C 0.11944400 4.83523000 0.07555000

C 1.36683600 5.51219800 0.18562400

C 2.53377800 4.79654100 0.28600200

C 2.49974200 3.35823700 0.27880800

C 1.30697800 2.67397600 0.20023700

C 3.94384800 5.19187400 0.38086700

C 4.73425300 4.00350500 0.40847900

C 3.85107300 2.85804900 0.33638800

C -1.13733300 2.71092700 -0.01722300

C -2.30220900 3.43205100 -0.15691100

C -2.28972000 4.87054300 -0.18414000

C -1.10347300 5.55029000 -0.06371100

C -3.66183300 2.97318500 -0.29533100

C -4.50444300 4.14486400 -0.41634000

C -3.68057000 5.30859200 -0.34885900

C 4.55549200 6.43485400 0.43350200

C 5.95065800 6.50099900 0.51378200

C 6.72427700 5.33662600 0.53863100

C 6.12479100 4.07960600 0.48515500

C -5.88537100 4.26296500 -0.57225900

C -6.44222000 5.53742600 -0.66142400

C -5.63587800 6.67777200 -0.59578900

C -4.24990200 6.56942200 -0.43880500

C 4.13237300 1.50285000 0.24914400

C -3.98792700 1.62584000 -0.26593300

C -4.08070000 0.40788400 -0.18939000

Si -3.91133600 -1.38506700 0.20044000

C 4.17145400 0.28731500 0.11166500

Si 3.83964700 -1.49590800 -0.21449600

H 1.37403600 6.59716500 0.17600200

H 1.28974500 1.59078200 0.20814200

H -1.15747200 1.62742900 -0.00131500

H -1.07497400 6.63484600 -0.07827700

H 3.96682300 7.34505000 0.41255500

H 6.43864600 7.46759900 0.55633200

H 7.80341400 5.41422900 0.60020100

H 6.72222300 3.17556300 0.50366700

H -6.50807700 3.37740400 -0.62285600

H -7.51337200 5.64764100 -0.78326500

H -6.09091000 7.65862600 -0.66743000

H -3.63553400 7.46131100 -0.38876100

C 5.14704900 -2.13510700 -1.43503700

C 6.53358800 -1.48179400 -1.25831500

C 5.28243000 -3.67210500 -1.45392100

H 4.77033700 -1.82762400 -2.42284000

C 7.52139200 -1.95212600 -2.33322400

H 6.93302800 -1.73642300 -0.26908000

H 6.44116200 -0.39309800 -1.28099300

C 6.26493900 -4.14008800 -2.53514100

H 5.64696800 -4.01211300 -0.47756600

H 4.30967000 -4.14882500 -1.60361000

C 7.63777600 -3.48033200 -2.36392400

H 8.50423600 -1.50026700 -2.16421700

H 7.17664600 -1.59723700 -3.31283500

H 6.36138300 -5.23043600 -2.50908700

H 5.85972600 -3.88286500 -3.52201800

H 8.31388300 -3.79534900 -3.16485000

H 8.08500000 -3.82509900 -1.42257400

C 3.84256600 -2.31086900 1.50314400

C 3.33149500 -3.76344600 1.58053900

C 5.21295700 -2.18474400 2.20104400

H 3.13318400 -1.68929100 2.07178100

C 3.28430200 -4.26771200 3.02913100

H 3.98677700 -4.41926000 0.99725500

H 2.33678100 -3.84367100 1.13947600

C 5.16295700 -2.69122800 3.64790200

H 5.95652500 -2.77089700 1.64673400

H 5.55391800 -1.14563300 2.17807500

C 4.64603100 -4.13239800 3.71875600

H 2.94811100 -5.30956000 3.05172100

H 2.53903400 -3.68514100 3.58445300

H 6.15468300 -2.61997800 4.10619800

H 4.49934600 -2.03961400 4.23012800

H 4.57971100 -4.46295200 4.75999300

H 5.36649300 -4.79679100 3.22417300

C 2.09863300 -1.51367100 -0.99344600

C 1.90053700 -0.41681200 -2.06103300

C 1.66592900 -2.87538500 -1.57750500

H 1.42143000 -1.27815600 -0.15896700

C 0.44923300 -0.36746700 -2.55527000

H 2.56199300 -0.61968100 -2.91366900

H 2.19529800 0.55860600 -1.67140500

C 0.21546700 -2.84062100 -2.08032200

H 2.32692200 -3.12980800 -2.41526700

H 1.77773000 -3.67589900 -0.84279700

C -0.00446400 -1.72364300 -3.10514800

H 0.33615500 0.41008000 -3.31689000

H -0.20256500 -0.07967400 -1.72033100

H -0.05780400 -3.81040000 -2.50939700

H -0.44922500 -2.67937900 -1.22484600

H -1.05733000 -1.68221100 -3.40125800

H 0.56742700 -1.95159700 -4.01357100

C -2.22610800 -1.48055100 1.08074400

C -2.15251100 -0.59958800 2.34266500

C -1.71044500 -2.90201900 1.38294400

H -1.53802000 -1.04142000 0.34085100

C -0.73396300 -0.56779400 2.92550500

H -2.84216500 -0.99289200 3.10094900

H -2.48621200 0.41617300 2.11478000

C -0.29843600 -2.87583400 1.98253200

H -2.38598800 -3.39870200 2.08757200

H -1.70895100 -3.51180500 0.47540100

C -0.21931600 -1.98044500 3.22305500

H -0.71110100 0.04432200 3.83244200

H -0.06366800 -0.08148800 2.20491500

H 0.02573200 -3.89253600 2.22642000

H 0.39807900 -2.49814600 1.22714900

H 0.80972600 -1.94052200 3.59354700

H -0.82454400 -2.42123400 4.02555300

C -5.39493100 -1.79724200 1.30984300

C -6.72813300 -1.66063100 0.54437000

C -5.32768600 -3.14567000 2.05417200

H -5.37827400 -1.00482200 2.07309000

C -7.93851400 -1.86716100 1.46294000

H -6.76430000 -2.40739900 -0.25823500

H -6.78519400 -0.68087200 0.06071200

C -6.54604300 -3.35042400 2.96514600

H -5.28019200 -3.96848800 1.33216600

H -4.41415100 -3.20374300 2.65120000

C -7.86120300 -3.21389600 2.19044000

H -8.86569000 -1.79969700 0.88455300

H -7.96832600 -1.05740100 2.20273900

H -6.49031300 -4.33028600 3.45043500

H -6.52136900 -2.60046000 3.76589100

H -8.71405500 -3.33224500 2.86601000

H -7.93017500 -4.02452600 1.45335800

C -3.81169600 -2.34685300 -1.43579000

C -4.11665100 -3.85363100 -1.30763300

C -4.64306400 -1.73191700 -2.58042100

H -2.75452600 -2.25505400 -1.72584000

C -3.86979200 -4.58998700 -2.63062700

H -5.16589000 -3.98629000 -1.01874100

H -3.51907900 -4.30906700 -0.51310800

C -4.40777400 -2.46910300 -3.90473100

H -5.70870800 -1.78134900 -2.32605600

H -4.40312200 -0.67133900 -2.69121500

C -4.68317700 -3.97135900 -3.77301500

H -4.11383300 -5.65182300 -2.52213800

H -2.80124900 -4.53461000 -2.87426700

H -5.03293300 -2.03650100 -4.69248300

H -3.36547400 -2.32032200 -4.21422900

H -4.46496700 -4.48287200 -4.71568000

H -5.75181200 -4.12395700 -3.57376100

**Indeno[2.1-*b*]fluorenes dimer**

**Compound 14a**

C 0.16249900 -2.75646300 2.18059200

C -0.75561700 -2.61433700 1.40417600

C -1.73674300 -2.43972300 0.33887300

C -2.85153900 -3.48576500 0.37637500

C -2.73368700 -4.86304100 0.32927000

H -1.75572100 -5.32871500 0.29327800

C -3.89843700 -5.63272700 0.32311000

H -3.83036500 -6.71337300 0.28341800

C -5.15295800 -5.01922500 0.36234600

H -6.04731800 -5.63133500 0.35140900

C -5.26933100 -3.63111900 0.42336600

H -6.24687900 -3.16456500 0.46474700

C -4.10828300 -2.85972000 0.43831800

C -3.91531200 -1.40638500 0.50793500

C -4.84249700 -0.36429100 0.56005200

H -5.90799200 -0.56430400 0.57003900

C -4.35420700 0.94087800 0.58391300

C -2.95845500 1.18668900 0.57384800

C -2.03371900 0.14957400 0.51799700

H -0.96756800 0.34353800 0.49688500

C -2.53182900 -1.13976900 0.47968400

C -5.04127600 2.23862300 0.60879900

C -6.39477900 2.56141900 0.62164400

H -7.15033100 1.78397100 0.61897300

C -6.76824300 3.90500100 0.63788800

H -7.81931000 4.16898700 0.64750400

C -5.80390300 4.91679400 0.64320200

H -6.11652200 5.95429200 0.65734400

C -4.44452400 4.60469900 0.63247100

H -3.69536200 5.38805700 0.64193200

C -4.06730400 3.26782800 0.61300000

C -2.73181300 2.64665800 0.59691900

C -1.52815700 3.17944100 0.57709300

C -0.25449400 3.44998100 0.61760000

C 0.56608500 3.50803300 -0.62877800

C 1.81696400 3.13991800 -0.60786900

C 2.97191300 2.51044600 -0.62800300

C 4.35097300 3.00118100 -0.46010000

C 4.84124700 4.29210200 -0.31130000

H 4.16776400 5.14141900 -0.32078700

C 6.21569000 4.47313700 -0.15429000

H 6.61590400 5.47330800 -0.03689600

C 7.08232500 3.37686300 -0.14903800

H 8.14699000 3.53851500 -0.02647400

C 6.59530000 2.07866100 -0.30085300

H 7.27644300 1.23529600 -0.29642900

C 5.22602100 1.88604400 -0.45767900

C 4.43284800 0.66303700 -0.63160800

C 3.07083900 1.03946600 -0.74738500

C 2.06086100 0.10100000 -0.90686800

H 1.02165800 0.39998100 -0.98174900

C 2.43325300 -1.23524900 -0.95190800

C 3.79228400 -1.63110500 -0.83387400

C 4.80090000 -0.68112000 -0.67419800

H 5.83814200 -0.98042200 -0.57360200

C 3.84145900 -3.09684300 -0.87136800

C 4.90735300 -3.98625600 -0.76439400

H 5.92167100 -3.62367700 -0.64182100

C 4.65137400 -5.35601200 -0.81421700

H 5.47267600 -6.05818100 -0.73081100

C 3.34732200 -5.83640500 -0.96836800

H 3.17100900 -6.90510300 -1.00178400

C 2.27204000 -4.95428200 -1.06995700

H 1.25808900 -5.32254400 -1.17678900

C 2.52201900 -3.58937500 -1.01881700

C 1.59535600 -2.44267000 -1.05847200

C 0.28041700 -2.46776200 -1.07936200

C -1.01652800 -2.44979600 -1.04767100

Si 1.66723600 -3.01845100 3.20589000

Si 0.54858200 3.47797200 2.35157900

Si -0.23302600 3.81341400 -2.33248700

Si -1.92294600 -2.41698100 -2.72766700

C 1.88995300 4.79420900 2.38908000

H 1.50023200 5.76577200 2.07351700

H 2.28045200 4.90249600 3.40564300

H 2.72610000 4.53273900 1.73799000

C -0.80231300 3.86077500 3.59873100

H -1.24208900 4.84566500 3.42087400

H -1.60576000 3.12194900 3.54661000

H -0.39905000 3.84934700 4.61562600

C 1.26722400 1.77090500 2.66885000

H 0.48364000 1.00859400 2.65224200

H 2.01349600 1.50250400 1.91916500

H 1.74835800 1.73772000 3.65145600

C 2.90369700 -1.65857900 2.82357300

H 3.14443500 -1.63124500 1.75957800

H 2.51414300 -0.67802700 3.10265900

H 3.83334800 -1.82571800 3.37671500

C 2.39008200 -4.69491200 2.75920900

H 3.27505700 -4.90454100 3.36812700

H 1.66596900 -5.49652800 2.92733700

H 2.68790700 -4.72238300 1.70898400

C 1.17721100 -2.96954000 5.02207900

H 0.73278600 -2.00484900 5.28124700

H 0.44635600 -3.74862500 5.25432700

H 2.05081200 -3.12250900 5.66317200

C -0.60663600 -2.05684700 -4.02183500

H -0.13112600 -1.08888000 -3.84416600

H 0.17855900 -2.81645500 -4.02179500

H -1.05657400 -2.03360800 -5.01891500

C -3.24358900 -1.08105200 -2.78494700

H -4.11070500 -1.33745200 -2.17598500

H -2.86372800 -0.11827400 -2.44082200

H -3.58041400 -0.96126700 -3.81984000

C -2.70939600 -4.09320300 -3.06298000

H -1.97913000 -4.90064300 -2.96560500

H -3.53134200 -4.29846400 -2.37496200

H -3.10468800 -4.11735100 -4.08365800

C 1.13725500 4.21916900 -3.54974600

H 1.65615800 5.14028000 -3.27171900

H 1.87860400 3.41699400 -3.58585100

H 0.73182200 4.35038600 -4.55726400

C -1.46484700 5.22668300 -2.19581500

H -2.30076600 4.96359600 -1.54440200

H -0.99061100 6.12725500 -1.79652800

H -1.87338600 5.46986200 -3.18139900

C -1.10294900 2.21897600 -2.82472300

H -0.38785200 1.39562700 -2.90130000

H -1.86032000 1.94411400 -2.08855800

H -1.59787900 2.32572400 -3.79468200

**Compound 14b**

Si -3.29900700 -2.76305700 -2.73491000

Si 2.98144800 -1.70502300 -2.52445100

C 1.62842900 4.11973700 -1.64116300

C 2.61768300 3.10533000 -1.60738600

C 1.95448300 1.80992600 -1.37315400

C 2.48294200 0.61964400 -1.17639100

C 2.87627300 -0.59972200 -0.96955600

C -3.09324200 1.08939400 -0.50132900

C -4.11343800 2.22206200 -0.60139000

C -3.47129900 3.43097900 -0.93104400

C -2.03476700 3.18035600 -1.10497400

C -1.80625200 1.80493200 -0.92301800

C -0.55154700 1.24189100 -1.05059300

H -0.40638000 0.17489900 -0.95264700

C 0.51161500 2.10204600 -1.29021100

C 0.31220900 3.49905600 -1.44159800

C 3.96265600 3.41744000 -1.75664200

H 4.71395500 2.63704600 -1.71948200

C 4.32413300 4.75163400 -1.94158000

H 5.36921100 5.01371100 -2.05760700

C 3.35274900 5.75683300 -1.97048000

H 3.65531600 6.78798900 -2.11065000

C 2.00115200 5.44890700 -1.81997600

H 1.25549700 6.23560900 -1.84096400

C -0.96940300 4.04520900 -1.36933900

H -1.13162000 5.10967200 -1.49529200

C -3.36381600 -0.05949400 -1.35699100

C -3.45309600 -1.10108400 -1.96767400

C -5.47915200 2.17013400 -0.38974700

H -5.96198600 1.23060200 -0.14725900

C -6.21957400 3.35071800 -0.49470800

H -7.29078000 3.33094000 -0.33299900

C -5.58835400 4.55576800 -0.80846300

H -6.17670600 5.46274700 -0.88551600

C -4.21250700 4.60608100 -1.03189100

H -3.73200300 5.54475800 -1.28263900

Si -2.98121300 1.70452100 2.52480200

C -1.62809300 -4.11992700 1.64079700

C -2.61742500 -3.10559700 1.60692100

C -1.95429100 -1.81012800 1.37287200

C -2.48276400 -0.61983000 1.17622600

C -2.87604700 0.59958800 0.96962700

C 3.09345300 -1.08922200 0.50151600

C 4.11376200 -2.22176900 0.60181900

C 3.47168400 -3.43074000 0.93139800

C 2.03510100 -3.18024700 1.10514600

C 1.80648900 -1.80485300 0.92313000

C 0.55172400 -1.24190500 1.05054100

H 0.40649800 -0.17492400 0.95256800

C -0.51139600 -2.10214300 1.29003000

C -0.31189500 -3.49913900 1.44142900

C -3.96239900 -3.41782700 1.75591200

H -4.71375700 -2.63749400 1.71867900

C -4.32380000 -4.75206600 1.94069000

H -5.36887700 -5.01423400 2.05652500

C -3.35233900 -5.75718800 1.96968700

H -3.65484700 -6.78838000 2.10971800

C -2.00074100 -5.44914100 1.81944500

H -1.25502800 -6.23578700 1.84048100

C 0.96977200 -4.04518700 1.36937000

H 1.13205400 -5.10964000 1.49532100

C 3.36378400 0.05984000 1.35700200

C 3.45278300 1.10152000 1.96757500

C 5.47951500 -2.16969000 0.39048300

H 5.96229900 -1.23011600 0.14805400

C 6.22004900 -3.35018400 0.49566700

H 7.29129100 -3.33028200 0.33421300

C 5.58889400 -4.55529700 0.80930700

H 6.17732800 -5.46220800 0.88652800

C 4.21300100 -4.60575900 1.03242900

H 3.73254700 -5.54447900 1.28311400

Si 3.29790700 2.76352800 2.73458300

C -4.56756400 -3.92311100 -1.97830700

H -4.50162900 -4.91397000 -2.43840100

H -4.39687700 -4.03755300 -0.90570700

H -5.58446100 -3.54953800 -2.12543800

C -1.55502600 -3.35438300 -2.35155000

H -0.81399800 -2.67894500 -2.78600200

H -1.38783900 -3.38989500 -1.27281900

H -1.37771900 -4.35554000 -2.75564700

C -3.55734500 -2.61754800 -4.59202900

H -3.43825300 -3.59115100 -5.07726500

H -4.55936000 -2.24597600 -4.82241000

H -2.83404400 -1.92777000 -5.03479900

C -4.77359900 1.82037700 3.08588000

H -4.83412900 2.34758400 4.04327200

H -5.38333500 2.36331500 2.36137900

H -5.20634900 0.82562000 3.22199600

C -1.95356200 0.84672900 3.84668300

H -2.33723600 -0.15000200 4.07567000

H -0.91344600 0.73467600 3.52915200

H -1.96376600 1.43408200 4.76980000

C -2.29082800 3.42601500 2.22272900

H -1.32120200 3.40087900 1.72417700

H -2.96851500 4.03640300 1.62518400

H -2.15680400 3.91869200 3.19127300

C 1.55364900 3.35397100 2.35114200

H 1.38630800 3.38894800 1.27241700

H 0.81292400 2.67843800 2.78596500

H 1.37600800 4.35522700 2.75484400

C 3.55631200 2.61837000 4.59172600

H 4.55855400 2.24739700 4.82209500

H 3.43668800 3.59195100 5.07687600

H 2.83342600 1.92822400 5.03459600

C 4.56586700 3.92417200 1.97789500

H 4.49941600 4.91502600 2.43792500

H 5.58295900 3.55113600 2.12504900

H 4.39514700 4.03845300 0.90528600

C 4.77396000 -1.82228500 -3.08481800

H 5.38301400 -2.36569500 -2.36010400

H 4.83437600 -2.34954500 -4.04219000

H 5.20755900 -0.82788000 -3.22080900

C 1.95489800 -0.84689100 -3.84696300

H 0.91467800 -0.73426900 -3.52997600

H 2.33918900 0.14960600 -4.07593300

H 1.96527600 -1.43440800 -4.76997600

C 2.28972800 -3.42596700 -2.22224900

H 2.96677400 -4.03672900 -1.62435600

H 1.31996400 -3.40004000 -1.72400700

H 2.15567300 -3.91875500 -3.19073400

**Compound 12a**

C 4.19481900 1.78018100 4.51900400

H 4.59429400 2.67525700 5.01364000

C 2.89650400 1.37439300 5.23708000

H 2.16172300 2.18342500 5.25003700

H 3.09836100 1.09449600 6.27681300

H 2.43360800 0.51205100 4.74782400

C 5.24479300 0.66253100 4.62925800

H 6.21536800 0.97145000 4.23464800

H 4.92959400 -0.22819200 4.07781800

H 5.39208700 0.37046500 5.67496500

C 5.40413100 2.84232000 1.78007300

H 5.02109800 3.38377900 0.90646700

C 6.26884400 1.68900600 1.24790400

H 5.67939000 1.00032100 0.64244500

H 6.71953400 1.11335400 2.06073400

H 7.08340700 2.07630200 0.62596000

C 6.25567200 3.81916700 2.60959700

H 5.68454900 4.68510100 2.95147900

H 7.09826400 4.19401500 2.01819500

H 6.67255600 3.32982500 3.49510700

C 2.46834600 3.57370100 2.64325800

H 1.60746000 3.08156000 3.11302400

C 2.77487300 4.84504100 3.45002700

H 3.05012000 4.62661800 4.48568100

H 1.90217200 5.50677900 3.47242300

H 3.59482500 5.41199800 3.00010100

C 2.08384500 3.90995500 1.19643000

H 1.81013600 3.01579700 0.63621800

H 2.90848500 4.39204800 0.66468600

H 1.22877800 4.59330400 1.16141600

C 3.23693200 0.75769800 1.86225100

C 3.00133800 -0.26875700 1.26523700

C 2.82108400 -1.48411700 0.47825900

C 3.90580900 -2.50586000 0.84687200

C 5.28016500 -2.34402700 0.85613700

H 5.72656900 -1.39327400 0.59942600

C 6.08289500 -3.42701300 1.22046300

H 7.16085200 -3.31797300 1.22477500

C 5.50687300 -4.64490300 1.58712300

H 6.14377700 -5.47635800 1.86575500

C 4.12156900 -4.79406300 1.62376500

H 3.67869600 -5.73133700 1.94025100

C 3.31997800 -3.71287600 1.26278800

C 1.86287700 -3.56029900 1.26272700

C 0.86284100 -4.47143900 1.59712600

H 1.10678700 -5.48652600 1.88906800

C -0.45682500 -4.03339500 1.55156400

C -0.76553100 -2.69516100 1.19564500

C 0.23393600 -1.79838600 0.82068400

H -0.00073100 -0.78367600 0.52167100

C 1.54384600 -2.25009800 0.85385000

C -1.71172700 -4.73707600 1.83202000

C -1.96038200 -6.05562500 2.20124600

H -1.14521500 -6.75909800 2.32763900

C -3.27824500 -6.46142500 2.40717500

H -3.48619900 -7.48581100 2.69323900

C -4.33500900 -5.55997400 2.25255200

H -5.35240000 -5.89245500 2.42211500

C -4.09515500 -4.23582800 1.88618700

H -4.91662800 -3.53971800 1.77898000

C -2.78400200 -3.82827500 1.66900500

C -2.23015500 -2.51498800 1.28239900

C -2.87442100 -1.38740700 1.06062200

C -4.21820600 -0.19082800 3.82080800

H -3.42098200 -0.93659200 3.93785900

C -4.33030000 0.58522900 5.14461400

H -3.40505900 1.10699600 5.40139700

H -4.56716000 -0.09252300 5.97178200

H -5.12941500 1.33096300 5.09884600

C -5.51309100 -0.96276400 3.53221600

H -5.44978000 -1.52061300 2.59847800

H -6.37531400 -0.29567800 3.46279200

H -5.71837000 -1.68060800 4.33369100

C -4.68866200 2.38020700 1.92765700

H -4.29435000 2.70147800 0.95876600

C -6.16268100 2.00286700 1.72525800

H -6.28453600 1.13359600 1.07638200

H -6.71655700 2.83264900 1.27205300

H -6.64670700 1.77434300 2.67825600

C -4.56279100 3.58291300 2.87812100

H -3.53368800 3.93866400 2.95637200

H -4.91124400 3.34275600 3.88663700

H -5.16948000 4.41814800 2.51193500

C -1.86905100 1.57615400 2.90868400

H -2.08286800 2.30164300 3.70522000

C -0.93454600 0.50676000 3.49533800

H -1.34561700 0.04757700 4.39745800

H 0.03341000 0.94487600 3.75804000

H -0.73603500 -0.29297100 2.77984400

C -1.18034200 2.32882800 1.76285100

H -1.81436400 3.09960400 1.31919700

H -0.89441800 1.64570100 0.96152100

H -0.26602500 2.81163400 2.11215800

C -3.40698800 -0.21890200 0.85776800

C -3.70374000 0.21993700 -0.55497700

C -5.71095800 0.33831700 -2.85206700

H -4.99711900 1.07968700 -3.23512900

C -6.37708400 -0.33833900 -4.06208000

H -5.65497600 -0.84843800 -4.70416300

H -6.90153600 0.40023700 -4.67786500

H -7.11787200 -1.07776700 -3.74403400

C -6.74950800 1.10456900 -2.01996600

H -6.30403300 1.56605700 -1.13838800

H -7.55353100 0.44871900 -1.67816100

H -7.20921700 1.89993700 -2.61648600

C -5.49043200 -2.27150100 -0.96075200

H -4.71040100 -2.68879700 -0.31533600

C -6.64458400 -1.81574000 -0.05810400

H -6.35820100 -0.99198300 0.59750500

H -6.98923900 -2.63865900 0.57845600

H -7.50243600 -1.48654200 -0.64929100

C -5.94826900 -3.40165000 -1.89806800

H -5.12458800 -3.80728300 -2.48904500

H -6.72148800 -3.06184600 -2.59279700

H -6.37169500 -4.22815800 -1.31708300

C -3.32384500 -1.60336600 -3.05021800

H -3.90932400 -2.24543800 -3.72192100

C -2.30909700 -2.49400300 -2.32023900

H -2.78391000 -3.25197800 -1.69298900

H -1.66038800 -1.90012600 -1.67180200

H -1.66768900 -3.01376300 -3.03591200

C -2.61014700 -0.55300100 -3.91498400

H -3.31122000 0.04488300 -4.50180100

H -1.91995100 -1.03389600 -4.61438100

H -2.02206600 0.13589700 -3.30471200

C -3.23884500 1.36166100 -0.96972000

C -2.65869800 2.46363800 -1.39534900

C -3.22922700 3.82130400 -1.49079200

C -4.52669600 4.27687600 -1.29031800

H -5.31982800 3.58898400 -1.02618800

C -4.79014700 5.63833200 -1.44260800

H -5.79618100 6.01030200 -1.28796600

C -3.77105900 6.52664100 -1.79709100

H -3.99678400 7.58033700 -1.91282700

C -2.46908900 6.07299400 -2.00665900

H -1.68531500 6.76887400 -2.28363900

C -2.19529300 4.71688800 -1.85385200

C -0.94541500 3.96119300 -1.97761200

C -1.22366600 2.59264200 -1.72752300

C -0.20812600 1.64234600 -1.67074900

H -0.42515100 0.60606200 -1.43917300

C 1.09827700 2.08012100 -1.86213100

C 1.37956600 3.44725500 -2.13207700

C 0.36013500 4.39223600 -2.19560700

H 0.58227300 5.44025200 -2.36187000

C 2.83117900 3.61718000 -2.19365400

C 3.61268600 4.75054800 -2.39934800

H 3.15251200 5.71353400 -2.58960700

C 5.00087100 4.63106000 -2.34904300

H 5.62149500 5.50591200 -2.50427300

C 5.60086900 3.39352300 -2.09994000

H 6.68123000 3.31821000 -2.06160900

C 4.82417400 2.25378800 -1.89443700

H 5.29106000 1.30074900 -1.69440900

C 3.44142000 2.36817500 -1.93658500

C 2.38109700 1.35858400 -1.72050900

C 2.56698000 0.09486700 -1.39530400

C 2.79741700 -1.13792600 -1.05460600

C 2.47598500 -1.68142800 -4.11628800

H 2.54731100 -2.54420900 -4.79521300

C 0.99666900 -1.26578300 -4.05412000

H 0.35416200 -2.04340300 -3.64684000

H 0.62957200 -1.02232500 -5.05699500

H 0.86425500 -0.37363600 -3.44113100

C 3.29943200 -0.53896800 -4.74016400

H 4.33675300 -0.81834900 -4.92427500

H 3.30045600 0.34712500 -4.10356500

H 2.86392600 -0.24652200 -5.70198100

C 5.06929300 -2.59239200 -2.58680800

H 5.30990100 -3.27927700 -1.76631500

C 5.85846000 -1.29603900 -2.34960900

H 5.56426200 -0.80222000 -1.42514900

H 5.70700000 -0.58227900 -3.16240000

H 6.93256900 -1.50431100 -2.29124100

C 5.55035900 -3.24452300 -3.89853500

H 5.05508100 -4.19022600 -4.11434800

H 6.62676300 -3.44210200 -3.84574100

H 5.38769900 -2.58749100 -4.75575400

C 2.39839000 -4.08084800 -2.06158900

H 2.74997500 -4.28436000 -1.04808100

C 2.91316000 -5.23457800 -2.93988000

H 3.98159800 -5.40928000 -2.80609100

H 2.72694600 -5.05760200 -4.00408300

H 2.39570100 -6.16292900 -2.67355000

C 0.86365400 -4.10112100 -2.03233200

H 0.43917700 -3.26142600 -1.48406400

H 0.50363900 -5.01748600 -1.55411400

H 0.45551500 -4.08395100 -3.04590300

Si 3.84472700 2.26788900 2.71515100

Si -3.57818700 0.91192500 2.40717800

Si -4.60324000 -0.85469300 -1.86497900

Si 3.16673600 -2.38115500 -2.47946600

**Compound 12b**

Si 5.33088000 2.26839800 -1.17432700

Si -2.61925500 3.41531600 -1.52313000

C -2.21282900 -2.26384900 -3.37500900

C -2.94353300 -1.19119300 -2.81187200

C -2.02733100 -0.37337900 -1.99422400

C -2.33308900 0.69346000 -1.28452300

C -2.62003400 1.74458100 -0.58129300

C 2.92248900 -1.52653300 -0.95170900

C 3.64565200 -2.69205500 -1.63584200

C 2.77704300 -3.35084300 -2.52571600

C 1.48059600 -2.66730200 -2.52356100

C 1.56903000 -1.54955900 -1.67463000

C 0.50122400 -0.69138200 -1.48225700

H 0.59796800 0.18356600 -0.85446600

C -0.70332900 -1.01541500 -2.09875800

C -0.81891700 -2.16304300 -2.92880400

C -4.30178800 -1.04589500 -3.05781300

H -4.85488400 -0.22470400 -2.62000200

C -4.93905600 -1.98844400 -3.86457800

H -6.00059700 -1.89459600 -4.06169800

C -4.22480700 -3.05683000 -4.41506100

H -4.73989300 -3.78070700 -5.03568500

C -2.85802900 -3.20065700 -4.17652000

H -2.31232200 -4.03222100 -4.60779100

C 0.28159000 -2.98208900 -3.16567700

H 0.20213000 -3.85523500 -3.80318500

C 3.65868900 -0.27902400 -1.11819000

C 4.30267200 0.74481900 -1.17218300

C 6.45361400 2.17019500 0.35798500

H 5.75262600 2.32050800 1.18606400

C 7.11400600 0.80090800 0.57466800

H 6.37985900 -0.00637300 0.54345000

H 7.61536100 0.76313100 1.54877800

H 7.86859200 0.59043100 -0.18761500

C 6.22752200 2.31223100 -2.85134100

H 5.41567000 2.18151600 -3.57890400

C 7.20115800 1.13773200 -3.03701600

H 8.06536900 1.23170800 -2.37309900

H 7.58272400 1.10689000 -4.06351600

H 6.72140500 0.17769200 -2.82991500

C 6.91196100 3.65581500 -3.15201500

H 6.22183900 4.49910000 -3.06593900

H 7.31632600 3.66395300 -4.17017100

H 7.74629100 3.84245700 -2.47103300

C 4.16225100 3.75623700 -1.01014700

H 4.80057700 4.64885200 -0.96215900

C 3.33169000 3.68984900 0.28212300

H 2.69049000 2.80477100 0.28893900

H 2.68179200 4.56552000 0.37192300

H 3.95370200 3.64882000 1.17833000

C 3.24885000 3.88368600 -2.23955000

H 3.81424800 4.01338500 -3.16580200

H 2.57793900 4.74367100 -2.14008500

H 2.62654600 2.99186300 -2.35410700

C 7.48935800 3.30397200 0.42201400

H 8.26183300 3.18228200 -0.34257000

H 7.99435500 3.31172700 1.39433900

H 7.03505800 4.28878400 0.28138300

C -4.45150900 3.92677300 -1.74387500

H -4.72535300 4.37083900 -0.78022200

C -4.67119700 5.00291700 -2.82401300

H -4.05797200 5.89074400 -2.66510400

H -5.71859300 5.32441400 -2.83013800

H -4.44532800 4.62465000 -3.82351100

C -1.78919500 3.21508500 -3.23061000

H -1.75785300 4.25434900 -3.59076600

C -2.59548700 2.40516100 -4.26287800

H -2.67519700 1.35594500 -3.97707300

H -2.09445200 2.43781300 -5.23655500

H -3.60610100 2.78886000 -4.40431300

C -0.33874900 2.70427300 -3.19182800

H 0.29792800 3.29934300 -2.54099400

H 0.09870000 2.73281200 -4.19579600

H -0.28906200 1.66976500 -2.85059900

C -5.39217300 2.73474700 -1.99027400

H -5.20275300 2.27093200 -2.96148100

H -6.43866200 3.05938200 -1.98286600

H -5.27752200 1.95396300 -1.23744200

C -1.71907100 4.71192300 -0.45381100

H -2.27250000 4.70001200 0.48891700

C -0.25332100 4.36833800 -0.13809900

H -0.10002700 3.31726200 0.10891300

H 0.10061300 4.95591800 0.71450600

H 0.39449700 4.60816000 -0.98304700

C -1.81438100 6.14385300 -1.00825800

H -1.38753400 6.22212200 -2.01330900

H -1.25568900 6.83397000 -0.36630000

H -2.84450300 6.50203400 -1.04925400

C 4.96149200 -3.09628000 -1.49961300

H 5.64065800 -2.55703600 -0.85246500

C 5.40232100 -4.21006500 -2.21969400

H 6.42834100 -4.54298800 -2.11768100

C 4.53385500 -4.89065000 -3.07404000

H 4.89124400 -5.75256300 -3.62540400

C 3.21813300 -4.46052200 -3.24238300

H 2.55520700 -4.97708300 -3.92681300

Si -5.33087100 -2.26843000 1.17423300

Si 2.61915800 -3.41533300 1.52329100

C 2.21291200 2.26397300 3.37501100

C 2.94362700 1.19132500 2.81187400

C 2.02741100 0.37344500 1.99431300

C 2.33315900 -0.69343800 1.28467700

C 2.62000500 -1.74459600 0.58146700

C -2.92246300 1.52648600 0.95188900

C -3.64564200 2.69198200 1.63605600

C -2.77702000 3.35079500 2.52589900

C -1.48055700 2.66728900 2.52371200

C -1.56899000 1.54953700 1.67478800

C -0.50117200 0.69137400 1.48240900

H -0.59791200 -0.18358900 0.85463800

C 0.70339100 1.01544400 2.09887600

C 0.81898100 2.16309900 2.92888500

C 4.30191200 1.04611900 3.05770000

H 4.85502300 0.22494900 2.61987000

C 4.93919800 1.98874600 3.86435900

H 6.00076200 1.89496900 4.06138500

C 4.22493300 3.05711300 4.41486100

H 4.74003000 3.78105000 5.03540600

C 2.85812600 3.20085100 4.17642900

H 2.31241000 4.03240800 4.60770200

C -0.28153900 2.98211800 3.16578200

H -0.20207700 3.85528100 3.80326700

C -3.65861600 0.27895000 1.11838800

C -4.30256500 -0.74491500 1.17235200

C -6.45322700 -2.16988000 -0.35831300

H -5.75182600 -2.31934800 -1.18619600

C -7.11419700 -0.80079200 -0.57445100

H -6.38046800 0.00684000 -0.54239800

H -7.61511600 -0.76265900 -1.54877300

H -7.86925100 -0.59117300 0.18760400

C -6.22769800 -2.31244500 2.85113000

H -5.41588900 -2.18219000 3.57882300

C -7.20104100 -1.13774900 3.03708800

H -8.06526600 -1.23134200 2.37313500

H -7.58261500 -1.10708200 4.06359000

H -6.72105200 -0.17777400 2.83023700

C -6.91252800 -3.65594800 3.15127300

H -6.22259500 -4.49937800 3.06507800

H -7.31710700 -3.66429900 4.16934100

H -7.74676400 -3.84217000 2.47005600

C -4.16238200 -3.75636500 1.00998900

H -4.80079200 -4.64891900 0.96200700

C -3.33186400 -3.69003300 -0.28231100

H -2.69065000 -2.80496300 -0.28919700

H -2.68198200 -4.56571700 -0.37210300

H -3.95391700 -3.64903200 -1.17849200

C -3.24897400 -3.88393600 2.23937600

H -3.81438500 -4.01362300 3.16562200

H -2.57814500 -4.74398100 2.13987700

H -2.62658500 -2.99217500 2.35396700

C -7.48837000 -3.30413500 -0.42346300

H -8.26122200 -3.18331900 0.34088000

H -7.99296800 -3.31153400 -1.39600000

H -7.03363300 -4.28880600 -0.28327000

C 4.45140100 -3.92681900 1.74404500

H 4.72523700 -4.37095000 0.78041800

C 4.67110000 -5.00289200 2.82425100

H 4.05789000 -5.89073600 2.66538200

H 5.71850100 -5.32437100 2.83040000

H 4.44521800 -4.62457700 3.82372800

C 1.78909000 -3.21508000 3.23076500

H 1.75778200 -4.25433100 3.59095900

C 2.59537400 -2.40508900 4.26298800

H 2.67507900 -1.35588800 3.97712600

H 2.09433600 -2.43768600 5.23666600

H 3.60598900 -2.78877700 4.40444900

C 0.33863100 -2.70430600 3.19198000

H -0.29804600 -3.29942500 2.54119000

H -0.09879800 -2.73280300 4.19595800

H 0.28891100 -1.66981700 2.85069600

C 5.39208500 -2.73478200 1.99033600

H 5.20266400 -2.27085700 2.96149000

H 6.43856600 -3.05944200 1.98297100

H 5.27745600 -1.95408600 1.23741100

C 1.71894400 -4.71188900 0.45393400

H 2.27239500 -4.69999100 -0.48878200

C 0.25321700 -4.36824600 0.13818700

H 0.09997400 -3.31716000 -0.10881100

H -0.10071200 -4.95579700 -0.71443900

H -0.39463600 -4.60805800 0.98311000

C 1.81418900 -6.14383000 1.00836800

H 1.38735800 -6.22208100 2.01342700

H 1.25544700 -6.83391300 0.36641700

H 2.84429200 -6.50206600 1.04934200

C -4.96149600 3.09617500 1.49987000

H -5.64067300 2.55690900 0.85275500

C -5.40232400 4.20996200 2.21994900

H -6.42835500 4.54286200 2.11796900

C -4.53384300 4.89058000 3.07425300

H -4.89123300 5.75249500 3.62561300

C -3.21810900 4.46047900 3.24256200

H -2.55517400 4.97706000 3.92696800

**Compound 15a**

C 3.63032700 -1.58743300 -0.32185200

C 3.29951000 -0.47102800 -0.65115900

C 2.99930800 0.87058700 -1.14018200

C 4.10996800 1.26108400 -2.12945600

C 5.47914600 1.28159600 -1.92589800

H 5.89818400 1.01982000 -0.96444600

C 6.31508000 1.62198200 -2.99043200

H 7.38779100 1.64879500 -2.84166600

C 5.77969300 1.91490900 -4.24677600

H 6.44157100 2.18172400 -5.06241200

C 4.40692500 1.83144700 -4.46995500

H 3.99948600 2.01208400 -5.45794800

C 3.57307100 1.48136200 -3.40895100

C 2.13468800 1.20637500 -3.37757500

C 1.18868100 1.23417600 -4.40068200

H 1.46433100 1.51801900 -5.40987100

C -0.12203800 0.89134600 -4.08284300

C -0.47868900 0.52358000 -2.75865200

C 0.46905200 0.50598300 -1.73814500

H 0.20079600 0.23934500 -0.72380600

C 1.77120400 0.84302100 -2.06565300

C -1.32799600 0.84968900 -4.91268000

C -1.52210100 1.11517700 -6.26554400

H -0.69292800 1.41601300 -6.89588400

C -2.80222100 0.98404200 -6.80188300

H -2.96718900 1.18680500 -7.85360000

C -3.87557000 0.58892700 -5.99786000

H -4.86326100 0.48626700 -6.43204000

C -3.68825700 0.32164400 -4.64278100

H -4.51632300 0.00888600 -4.02393000

C -2.41750300 0.45743300 -4.10098700

C -1.92638100 0.22482400 -2.72770800

C -2.60756500 -0.21337700 -1.68648400

C -3.18852200 -0.73572000 -0.64759300

C -3.71879400 0.15066000 0.45148200

C -3.33609600 -0.13992100 1.66035400

C -2.78726300 -0.43833500 2.81736300

C -3.34129500 -1.27168300 3.89841800

C -4.60052500 -1.83980100 4.04390500

H -5.36750300 -1.67803300 3.29657100

C -4.85494000 -2.62160500 5.17065900

H -5.83076000 -3.07461100 5.30057800

C -3.86268400 -2.82571600 6.13439400

H -4.07984000 -3.43645700 7.00294600

C -2.59799000 -2.25477500 5.99252900

H -1.83508900 -2.42290200 6.74414700

C -2.33341000 -1.47359900 4.87076600

C -1.11296300 -0.79188900 4.42602500

C -1.38386100 -0.15245000 3.18771900

C -0.37614400 0.46326700 2.44969800

H -0.58268500 0.90148700 1.48009500

C 0.91240300 0.44336800 2.97352400

C 1.17871700 -0.14595700 4.24110600

C 0.16972400 -0.77229200 4.96824300

H 0.38664600 -1.26573200 5.90892500

C 2.61270700 -0.02758000 4.51465900

C 3.37237600 -0.38639000 5.62462600

H 2.90706900 -0.83850900 6.49311300

C 4.74710700 -0.15577500 5.60290500

H 5.35033000 -0.42898100 6.46090500

C 5.35956600 0.41466300 4.48284600

H 6.43124100 0.57566800 4.48229000

C 4.60577500 0.78056200 3.36835100

H 5.07764000 1.22155600 2.49892600

C 3.23367500 0.56801100 3.39370100

C 2.19638600 0.87802200 2.38692300

C 2.43684800 1.37852100 1.19271300

C 2.77146000 1.87043900 0.03840700

Si 4.26585500 -3.31293200 -0.31346200

Si -3.10879500 -2.66360100 -0.46303600

Si -4.72047300 1.78464100 0.25547400

Si 2.89347800 3.78615900 -0.10855100

C 2.19386100 4.51002200 1.51019700

C 3.12425900 4.32393100 2.72828100

C 1.78211500 5.99624000 1.40104000

H 1.28161500 3.93240200 1.71242500

C 2.46930400 4.80978400 4.02460300

H 4.04144700 4.90199300 2.57072500

H 3.42483500 3.28261600 2.83685400

C 1.16771600 6.52286800 2.70645000

H 2.66007100 6.60306800 1.15310100

H 1.07015700 6.14075200 0.58512000

C 2.07959400 6.28684900 3.91433100

H 3.14952800 4.65420100 4.86802400

H 1.57601000 4.20521000 4.22437400

H 0.93821400 7.58890400 2.60420400

H 0.21641700 6.01199500 2.88112500

H 1.58647800 6.62599500 4.83084600

H 2.99098200 6.88939700 3.80653200

C 1.83513900 4.22879200 -1.62368900

C 0.33494200 4.01523300 -1.34061700

C 2.07263400 5.60471200 -2.27650000

H 2.12709300 3.48789200 -2.37448600

C -0.51605300 4.18625700 -2.60251300

H 0.00667800 4.74160500 -0.59158200

H 0.16313200 3.02662100 -0.90879700

C 1.20853800 5.77895500 -3.53420300

H 1.84161100 6.40830400 -1.56768500

H 3.12622000 5.71653700 -2.54598300

C -0.27997800 5.55807600 -3.24130500

H -1.57608000 4.04846500 -2.36706400

H -0.25444900 3.40269500 -3.31765100

H 1.36889700 6.77339800 -3.96374400

H 1.53537000 5.05197600 -4.28852600

H -0.86584600 5.65579700 -4.16063700

H -0.63302300 6.34273400 -2.55935300

C 4.72508700 4.24027300 -0.36811500

C 5.02243100 5.75247800 -0.26175000

C 5.70151300 3.47526900 0.55043400

H 4.94483700 3.93761200 -1.40099200

C 6.47929700 6.05891800 -0.63540300

H 4.84412100 6.08559300 0.76679800

H 4.34982200 6.33447100 -0.89463800

C 7.16122200 3.76050100 0.18007900

H 5.53718200 3.77693900 1.58875400

H 5.50236100 2.40364400 0.51600100

C 7.46153400 5.26318200 0.23172100

H 6.67455500 7.13212400 -0.53999100

H 6.63645800 5.80153200 -1.69046400

H 7.83520600 3.21675800 0.85078400

H 7.35220000 3.38930600 -0.83350200

H 8.49151700 5.45760600 -0.08359900

H 7.38117300 5.60831900 1.27053000

C -3.42667700 3.18257700 0.34934900

C -3.92709500 4.55368700 -0.15484000

C -2.85395900 3.29764200 1.77843400

H -2.60651700 2.86913400 -0.31300300

C -2.87663100 5.66110200 -0.00069100

H -4.82399700 4.84779500 0.40163400

H -4.22388500 4.48025800 -1.20498300

C -1.79551500 4.39556400 1.89924600

H -3.66325700 3.52197000 2.48147600

H -2.43166000 2.34365400 2.09736400

C -2.34213500 5.74770500 1.43172400

H -3.30692300 6.62117300 -0.30438800

H -2.04537300 5.46326000 -0.68203100

H -1.44866000 4.45840700 2.93518700

H -0.92479200 4.12444300 1.29474300

H -1.56943300 6.51873400 1.49919500

H -3.15276500 6.05772000 2.10405700

C -5.82187600 1.80781200 1.81299700

C -6.48671600 3.15329600 2.17114300

C -6.86751400 0.67612000 1.85168500

H -5.10202000 1.59211000 2.61651200

C -7.19354600 3.08247900 3.53173500

H -7.22168000 3.42288400 1.40620800

H -5.74902300 3.95835700 2.18606000

C -7.58434100 0.60620900 3.20542100

H -7.61149800 0.83370800 1.06143600

H -6.39205400 -0.28267100 1.64126200

C -8.22466000 1.94930800 3.57083200

H -7.67413700 4.04061500 3.75496300

H -6.44345000 2.91530100 4.31494500

H -8.34108400 -0.18532100 3.18839100

H -6.85623300 0.33088400 3.97825000

H -8.69148200 1.89272200 4.55908000

H -9.02810700 2.17097200 2.85628700

C -5.68478000 1.85615900 -1.39068300

C -6.30935400 0.52684600 -1.85915900

C -6.78586900 2.94296600 -1.33832200

H -4.94802400 2.16058400 -2.14918600

C -7.07342800 0.67049300 -3.18325600

H -7.00625300 0.16320500 -1.09687000

H -5.53899800 -0.23840400 -1.95693400

C -7.51931700 3.09301000 -2.67603600

H -7.52033900 2.66279100 -0.57542000

H -6.37584100 3.90641000 -1.03226700

C -8.14220200 1.76388500 -3.11105900

H -7.52261000 -0.29027300 -3.45623400

H -6.37068700 0.93011900 -3.98231100

H -8.28866100 3.86759300 -2.59329400

H -6.81026800 3.43112700 -3.44210000

H -8.64327000 1.87123200 -4.07810500

H -8.91392400 1.47380800 -2.38646300

C -4.75739800 -3.27598500 0.27003300

C -5.99657800 -2.46211600 -0.13404100

C -5.03249200 -4.77646100 0.02646300

H -4.62330900 -3.14353000 1.35417500

C -7.24872200 -2.91615300 0.62392200

H -6.17149000 -2.56676800 -1.21074000

H -5.82154700 -1.40392600 0.04450300

C -6.27571900 -5.24593100 0.79392700

H -5.19416200 -4.94226400 -1.04465400

H -4.17387500 -5.38981500 0.30571500

C -7.50972300 -4.41340000 0.42764000

H -8.11716400 -2.33114600 0.30319700

H -7.11228500 -2.71123500 1.69362600

H -6.46220300 -6.30633100 0.59497700

H -6.08299800 -5.15596700 1.87051200

H -8.37404700 -4.73176500 1.01888800

H -7.76461500 -4.59646800 -0.62413600

C -2.74641200 -3.29227300 -2.21571900

C -2.26355800 -4.75374300 -2.31595100

C -3.93867100 -3.05386500 -3.16678100

H -1.92033800 -2.66030500 -2.57146600

C -1.94816500 -5.14331300 -3.76740800

H -3.03097100 -5.43052500 -1.92451000

H -1.37454400 -4.90266600 -1.69700000

C -3.59918100 -3.42655500 -4.61267500

H -4.79204900 -3.65869900 -2.83797100

H -4.26103100 -2.01243100 -3.11631200

C -3.13315100 -4.88357800 -4.70361600

H -1.64853500 -6.19528200 -3.81415800

H -1.09033800 -4.55677900 -4.11348900

H -4.46752000 -3.26108500 -5.25887200

H -2.80734900 -2.76070400 -4.97589400

H -2.86172300 -5.13505700 -5.73368900

H -3.96540800 -5.54446100 -4.42788800

C -1.74645900 -3.05537100 0.80241100

C -1.77563200 -4.49236400 1.36504200

C -0.32861600 -2.67386400 0.34639600

H -1.99030900 -2.40397500 1.64592100

C -0.74587100 -4.66916300 2.48874000

H -1.56797400 -5.21719300 0.56890700

H -2.76913700 -4.72594000 1.75452900

C 0.69173500 -2.86251800 1.47470700

H -0.03715800 -3.28414600 -0.51729900

H -0.30889000 -1.63728000 0.00447200

C 0.66289800 -4.29020100 2.02415300

H -0.76486100 -5.70155600 2.85368700

H -1.03145600 -4.02826400 3.33193400

H 1.68893600 -2.59212700 1.12436500

H 0.45434700 -2.16811800 2.28461700

H 1.37313800 -4.39059300 2.85039900

H 0.98659600 -4.99202500 1.24398700

C 5.92922200 -3.13135900 -1.21049300

C 6.92078300 -2.24593300 -0.42935000

C 6.60905600 -4.43448900 -1.67083900

H 5.65454800 -2.57308700 -2.11804800

C 8.18040000 -1.95207700 -1.25266300

H 7.21107200 -2.75231600 0.49968000

H 6.43538400 -1.31102400 -0.13524100

C 7.87236300 -4.14153800 -2.49265400

H 6.88225400 -5.04052400 -0.80023200

H 5.91568600 -5.03813600 -2.26367500

C 8.85236900 -3.24804200 -1.72247300

H 8.88491100 -1.35074300 -0.66791600

H 7.89901700 -1.35058400 -2.12643700

H 8.36123900 -5.07787700 -2.78139800

H 7.58121500 -3.63744500 -3.42291000

H 9.72606200 -3.02181600 -2.34198500

H 9.22327200 -3.79576800 -0.84635200

C 2.97837200 -4.26361900 -1.32851700

C 2.92932000 -3.79256800 -2.79449200

C 3.03497600 -5.79948500 -1.24491400

H 2.03007300 -3.95795800 -0.86592700

C 1.75347000 -4.42913800 -3.54222100

H 3.86615700 -4.06298100 -3.29832700

H 2.85295800 -2.70235700 -2.83666100

C 1.85756100 -6.43583600 -1.99738800

H 3.97533200 -6.16431900 -1.67610500

H 3.02458100 -6.12590500 -0.20046100

C 1.79410900 -5.95876500 -3.45336900

H 1.74771100 -4.10903100 -4.58937700

H 0.82019800 -4.06444500 -3.09784800

H 1.92777800 -7.52790200 -1.95861200

H 0.92428300 -6.16232900 -1.48862800

H 0.92437000 -6.39198000 -3.95590100

H 2.68042600 -6.32406900 -3.98832800

C 4.34819700 -3.98784000 1.45400300

C 5.35682600 -5.14172100 1.62866700

C 4.57292200 -2.90544500 2.52863300

H 3.34982400 -4.40904600 1.63010400

C 5.28973100 -5.73743200 3.04039300

H 6.37175700 -4.76682200 1.45152900

H 5.18346000 -5.92614500 0.88554200

C 4.51732400 -3.49902100 3.94078600

H 5.54925100 -2.42913500 2.37661400

H 3.83189000 -2.10986400 2.42944200

C 5.50424800 -4.65941300 4.10827300

H 6.03160900 -6.53568100 3.14911700

H 4.30493900 -6.19956900 3.18562700

H 4.71543500 -2.71858900 4.67775400

H 3.49844200 -3.85973200 4.13261800

H 5.41642100 -5.09357300 5.10956900

H 6.52844200 -4.27254000 4.02398500

**Compound 15b**

Si 5.94171500 1.61340900 -0.28903800

Si -1.81561600 3.94445300 -0.22289800

C -2.06311800 -0.49441800 -4.15904100

C -2.76226200 0.32148700 -3.23756100

C -1.86594900 0.63164600 -2.10746700

C -2.13391200 1.34646900 -1.03356000

C -2.32389100 2.10127800 0.00344500

C 2.81491000 -1.43197700 -1.33260800

C 3.45847700 -2.39003700 -2.34240300

C 2.65135500 -2.50649100 -3.48849000

C 1.46369200 -1.66181800 -3.32463100

C 1.56585600 -0.98102900 -2.09800900

C 0.57154700 -0.13376700 -1.64336200

H 0.67704600 0.39703100 -0.70715500

C -0.58978700 -0.04519700 -2.40283400

C -0.71400000 -0.73409400 -3.63859100

C -4.07913700 0.69374600 -3.47423100

H -4.60941300 1.31624600 -2.76488600

C -4.70151600 0.24431300 -4.63870700

H -5.72817900 0.52507500 -4.84096000

C -4.01711200 -0.56655000 -5.54908500

H -4.52156100 -0.90682400 -6.44587300

C -2.69462100 -0.94141900 -5.31598000

H -2.17051200 -1.57082700 -6.02622600

C 0.32638800 -1.52331900 -4.12144300

H 0.23508000 -2.05481600 -5.06179800

C 3.73501000 -0.34323500 -1.03065300

C 4.55625400 0.49817500 -0.74518300

C 4.67053900 -3.05162600 -2.25541000

H 5.31112700 -2.91962100 -1.39336700

C 5.05762500 -3.88739000 -3.30601300

H 5.99993800 -4.41918300 -3.24915000

C 4.24280700 -4.03673700 -4.42955100

H 4.55684400 -4.68955300 -5.23565900

C 3.03871800 -3.34127200 -4.53442700

H 2.42092700 -3.44430100 -5.41902600

Si 1.81523300 -3.94419500 0.22266200

C 2.06329900 0.49476300 4.15855800

C 2.76244100 -0.32119400 3.23711800

C 1.86615300 -0.63140400 2.10704100

C 2.13404300 -1.34625700 1.03313600

C 2.32390300 -2.10114200 -0.00383500

C -2.81480200 1.43197100 1.33216600

C -3.45850900 2.38989900 2.34199400

C -2.65140500 2.50641800 3.48809500

C -1.46362200 1.66191100 3.32421100

C -1.56571500 0.98114700 2.09756800

C -0.57134700 0.13397000 1.64288900

H -0.67683600 -0.39682100 0.70667900

C 0.58999200 0.04548100 2.40235100

C 0.71417700 0.73440500 3.63810000

C 4.07932600 -0.69340700 3.47377300

H 4.60957900 -1.31594100 2.76443900

C 4.70173700 -0.24388300 4.63819700

H 5.72842000 -0.52459000 4.84042900

C 4.01734200 0.56703600 5.54853300

H 4.52181100 0.90739200 6.44527900

C 2.69483700 0.94186700 5.31543800

H 2.17074800 1.57134000 6.02564300

C -0.32628100 1.52351500 4.12099200

H -0.23500200 2.05499500 5.06136000

C -3.73469500 0.34306500 1.03022000

C -4.55566900 -0.49862400 0.74480200

C -4.67063300 3.05136900 2.25499300

H -5.31116000 2.91935400 1.39290500

C -5.05784700 3.88703700 3.30562700

H -6.00022000 4.41872400 3.24877100

C -4.24307400 4.03641800 4.42919200

H -4.55721900 4.68914300 5.23533300

C -3.03889700 3.34110200 4.53406000

H -2.42113400 3.44417900 5.41867300

Si -5.94125700 -1.61391800 0.28921500

C -6.95581000 -1.80527700 1.88499800

C -7.52905500 -0.46590900 2.39141100

C -8.05322800 -2.88789700 1.86047600

H -6.20381100 -2.12571800 2.62167900

C -8.19878700 -0.61897400 3.76235000

H -8.27388000 -0.09576000 1.67614000

H -6.73815100 0.28806500 2.44110100

C -8.72544500 -3.03790500 3.23222900

H -8.81554500 -2.62730500 1.11823500

H -7.63719200 -3.84997700 1.54965900

C -9.28363700 -1.70177400 3.73551300

H -8.62430100 0.33755800 4.08341300

H -7.43559700 -0.88836100 4.50314300

H -9.52240700 -3.78712200 3.18114000

H -7.98600000 -3.41278600 3.95127000

H -9.72513200 -1.82487800 4.72952000

H -10.09516600 -1.37932600 3.07003500

C -5.15367800 -3.25535600 -0.24815500

C -4.57002700 -4.01327300 0.96343700

C -6.05742600 -4.17347900 -1.09412300

H -4.30882000 -2.95503500 -0.88518400

C -3.86703200 -5.31128400 0.55228400

H -5.37674500 -4.25893500 1.66435800

H -3.87304000 -3.36752800 1.50713500

C -5.33437200 -5.46321100 -1.50327800

H -6.95858900 -4.43681900 -0.52726000

H -6.39703600 -3.64448800 -1.98941800

C -4.78346000 -6.21030700 -0.28374100

H -3.51504600 -5.84478600 1.44056000

H -2.97908300 -5.06114600 -0.03524800

H -6.01190000 -6.11007400 -2.07030800

H -4.50611400 -5.20901100 -2.17452700

H -4.24563400 -7.10945900 -0.60124100

H -5.62127400 -6.55055600 0.33875500

C -6.85960000 -0.75622800 -1.14262200

C -8.28122400 -1.30464200 -1.38705900

C -6.90655600 0.78111500 -1.00162000

H -6.25767600 -0.98079700 -2.03609600

C -8.94326100 -0.64522700 -2.60349800

H -8.89997000 -1.10664800 -0.50404700

H -8.26530800 -2.39002800 -1.51535600

C -7.59080600 1.45121500 -2.19917600

H -7.45212700 1.04796500 -0.08934100

H -5.89565000 1.17388200 -0.87459000

C -8.98982700 0.87851200 -2.44942300

H -9.95312400 -1.04395500 -2.74517300

H -8.37239400 -0.90222600 -3.50489000

H -7.64588300 2.53282400 -2.04061100

H -6.97907900 1.29747100 -3.09506700

H -9.43715200 1.33967100 -3.33565800

H -9.63973200 1.13222800 -1.60200100

C 0.81064700 -3.99720800 1.84025300

C -0.13087800 -5.21067800 1.99863300

C 1.64284000 -3.84805500 3.13296500

H 0.16568000 -3.10910800 1.77183500

C -1.05384600 -5.03051800 3.21213400

H 0.46345900 -6.12331000 2.12615900

H -0.73849100 -5.35993300 1.10563300

C 0.73361400 -3.63291400 4.34786000

H 2.23172700 -4.75977200 3.28680900

H 2.35495400 -3.02824000 3.06074100

C -0.26242800 -4.78816100 4.50300900

H -1.70209300 -5.90578500 3.32541600

H -1.71341800 -4.17404800 3.02458600

H 1.33577200 -3.52592400 5.25579800

H 0.19018500 -2.68898700 4.21823500

H -0.94661100 -4.59475700 5.33526600

H 0.29078800 -5.70146600 4.75811500

C 0.75006000 -4.42797000 -1.27853500

C 0.61385600 -5.95001400 -1.49715300

C -0.62609800 -3.72776300 -1.27862300

H 1.30127100 -4.05418900 -2.14598900

C -0.20910100 -6.26605800 -2.75233000

H 0.13510400 -6.42255400 -0.63218500

H 1.60551000 -6.40055000 -1.59131400

C -1.47024100 -4.09594000 -2.50593300

H -1.18112200 -3.99754200 -0.37524800

H -0.49524900 -2.64459500 -1.24119800

C -1.59184900 -5.61139100 -2.68865200

H -0.30417700 -7.35004800 -2.87492100

H 0.32913600 -5.89280700 -3.63303700

H -2.45918700 -3.63341400 -2.42680800

H -1.00170500 -3.66893200 -3.39740500

H -2.16497400 -5.83805000 -3.59347900

H -2.15061500 -6.04324400 -1.85044500

C 3.41724500 -4.96760200 0.30192900

C 4.56422500 -4.25677100 1.05148600

C 3.22742700 -6.38629500 0.88175100

H 3.73308100 -5.08237400 -0.74400600

C 5.86797300 -5.05844300 0.97069900

H 4.28803800 -4.12885000 2.10405300

H 4.71672200 -3.24923500 0.65942000

C 4.52632300 -7.19992700 0.80293900

H 2.91911900 -6.31151300 1.93022700

H 2.42740400 -6.91931800 0.36385800

C 5.68263300 -6.48211800 1.50789000

H 6.66146000 -4.54596400 1.52360600

H 6.19277700 -5.10638200 -0.07680100

H 4.37568800 -8.19308900 1.23867900

H 4.78541200 -7.35444200 -0.25224200

H 6.60926000 -7.05453800 1.39946900

H 5.46926900 -6.43154100 2.58331300

C 6.85964400 0.75550100 1.14293000

C 6.90686100 -0.78179800 1.00154200

C 8.28106600 1.30408600 1.38812900

H 6.25730800 0.97977400 2.03619600

C 7.59081000 -1.45218000 2.19911800

H 7.45274800 -1.04834600 0.08936900

H 5.89604100 -1.17466200 0.87409300

C 8.94268200 0.64441300 2.60465800

H 8.90024100 1.10648200 0.50533100

H 8.26487800 2.38942900 1.51675400

C 8.98961100 -0.87926600 2.45011200

H 7.64619400 -2.53371400 2.04015000

H 6.97871300 -1.29895500 3.09484700

H 9.95240600 1.04328500 2.74692100

H 8.37135600 0.90102300 3.50587100

H 9.43667300 -1.34062800 3.33637300

H 9.63990800 -1.13256900 1.60286800

C 6.95684400 1.80524500 -1.88438500

C 8.05402700 2.88809300 -1.85912600

C 7.53060400 0.46609300 -2.39078000

H 6.20511800 2.12567300 -2.62134900

C 8.72692900 3.03846200 -3.23050100

H 8.81600100 2.62756200 -1.11651100

H 7.63760700 3.85003200 -1.54840100

C 8.20097700 0.61954400 -3.76136000

H 8.27515500 0.09594500 -1.67522400

H 6.73986200 -0.28802400 -2.44098400

C 9.28562200 1.70253200 -3.73375500

H 9.52372600 3.78781800 -3.17886100

H 7.98778900 3.41334400 -3.94985600

H 8.62682500 -0.33684700 -4.08239800

H 7.43811100 0.88894700 -4.50248000

H 9.72760700 1.82590600 -4.72751000

H 10.09686400 1.38009500 -3.06792200

C 5.15376700 3.25466300 0.24842600

C 6.05716800 4.17282400 1.09472100

C 4.57024400 4.01260600 -0.96322000

H 4.30882600 2.95410100 0.88523000

C 5.33390000 5.46247500 1.50375500

H 6.95848700 4.43629700 0.52817500

H 6.39653700 3.64377900 1.99007700

C 3.86703100 5.31051100 -0.55209600

H 5.37705400 4.25842800 -1.66397900

H 3.87344200 3.36680300 -1.50708200

C 4.78326000 6.20958000 0.28409900

H 6.01123500 6.10934100 2.07100900

H 4.50546600 5.20819800 2.17475800

H 3.51507800 5.84400900 -1.44038900

H 2.97905700 5.06023500 0.03533600

H 4.24536900 7.10873500 0.60147500

H 5.62120600 6.54982600 -0.33822400

C -3.41788400 4.96746000 -0.30223300

C -3.22835900 6.38624800 -0.88191700

C -4.56469900 4.25644100 -1.05185700

H -3.73376800 5.08207000 0.74370900

C -4.52742700 7.19959400 -0.80307500

H -2.91998400 6.31164000 -1.93038400

H -2.42847000 6.91939000 -0.36393500

C -5.86861400 5.05783800 -0.97102400

H -4.28847100 4.12865000 -2.10443000

H -4.71698700 3.24885000 -0.65985000

C -5.68356800 6.48159400 -1.50810600

H -4.37699200 8.19282100 -1.23873400

H -4.78657500 7.35397300 0.25211200

H -6.66199200 4.54523600 -1.52397500

H -6.19344100 5.10563100 0.07647500

H -6.61032000 7.05380700 -1.39966100

H -5.47017200 6.43114100 -2.58352800

C -0.75070200 4.42842800 1.27840800

C 0.62542600 3.72814300 1.27865000

C -0.61444400 5.95050200 1.49677200

H -1.30197500 4.05479700 2.14588900

C 1.46954400 4.09647500 2.50592000

H 1.18048900 3.99775200 0.37525100

H 0.49450600 2.64498800 1.24138000

C 0.20858400 6.26674000 2.75185400

H -0.13573700 6.42291400 0.63171300

H -1.60609500 6.40105500 1.59091200

C 1.59128900 5.61195900 2.68827300

H 2.45843700 3.63381300 2.42694500

H 1.00090600 3.66972000 3.39746600

H 0.30373300 7.35075000 2.87421600

H -0.32963700 5.89371000 3.63266500

H 2.16447800 5.83881300 3.59300900

H 2.15004100 6.04354100 1.84991600

C -0.81087500 3.99775700 -1.84040300

C -1.64306700 3.84866500 -3.13313500

C 0.13058800 5.21126900 -1.99869900

H -0.16588500 3.10967000 -1.77204600

C -0.73384200 3.63364800 -4.34805100

H -2.23198600 4.76037100 -3.28691000

H -2.35515000 3.02882000 -3.06097200

C 1.05355500 5.03123700 -3.21222400

H -0.46379000 6.12388800 -2.12614800

H 0.73821000 5.36047800 -1.10569800

C 0.26215000 4.78894800 -4.50311900

H -1.33600500 3.52670300 -5.25599200

H -0.19037400 2.68973300 -4.21850400

H 1.70176700 5.90653900 -3.32543400

H 1.71315900 4.17477700 -3.02473900

H 0.94634200 4.59563700 -5.33538900

H -0.29110400 5.70225000 -4.75815400

**Fluoreno[2.3-*b*]fluorenes dimer**

**Compound 16a**

Si -4.77422900 0.39537700 -2.38616600

Si 3.83405100 1.74741300 -2.71892500

Si -4.51658600 -0.28525600 2.32362500

C -3.83032200 -2.87232800 -0.56760800

C -4.48630900 -4.17835400 -0.37553600

C -5.83225100 -4.50106200 -0.25085200

H -6.59033700 -3.72826300 -0.30477500

C -6.18639800 -5.83602000 -0.05857800

H -7.23136300 -6.10549800 0.04039700

C -5.20713500 -6.83157900 0.00621900

H -5.50335000 -7.86331900 0.15558400

C -3.85570600 -6.51334300 -0.12018100

H -3.10320900 -7.29202600 -0.06946500

C -3.49050200 -5.18395100 -0.31210300

C -2.17342800 -4.55643400 -0.47193300

C -0.89890400 -5.06970000 -0.46423700

H -0.72356800 -6.13126900 -0.32534600

C 0.21776600 -4.20728700 -0.63109400

C 1.54863300 -4.70190600 -0.60856600

H 1.71006100 -5.76447200 -0.46083200

C 2.60758200 -3.83901400 -0.75867000

C 4.05831000 -4.05359900 -0.73717700

C 4.81265800 -5.21041500 -0.55960700

H 4.32918000 -6.17112900 -0.42363900

C 6.20303900 -5.11488200 -0.55227000

H 6.80155400 -6.00739000 -0.41223900

C 6.83637400 -3.87908800 -0.71901000

H 7.91875000 -3.82704300 -0.70576500

C 6.09069300 -2.71473300 -0.89427000

H 6.57832000 -1.75388900 -1.01152200

C 4.70433200 -2.80464400 -0.90280900

C 3.68016900 -1.75103300 -1.01797000

C 2.37561700 -2.43704300 -0.94327200

C 1.10547000 -1.92878100 -0.97881100

H 0.93635400 -0.86708300 -1.12036400

C -0.00374400 -2.79467700 -0.81722600

C -1.32812600 -2.29154800 -0.81754600

H -1.49597900 -1.22721100 -0.94501900

C -2.37917800 -3.15040000 -0.64566200

C -4.32575600 -1.65429600 -0.59005400

C -4.57629000 -0.37561000 -0.65270100

C -4.49543400 0.47581400 0.57060400

C -4.22285200 1.74862200 0.49608000

C -5.67336000 4.64624200 0.45832200

H -6.44646400 3.88693400 0.48195200

C -6.00357200 6.00047000 0.42719300

H -7.04531800 6.29904700 0.42355200

C -5.00492900 6.97864200 0.40205000

H -5.28321300 8.02576800 0.37873700

C -3.65710100 6.62286100 0.40763000

H -2.88921900 7.38785100 0.38915200

C -3.31574000 5.27378200 0.43702000

C -2.00761900 4.60771600 0.44784700

C -0.72314900 5.09532400 0.43680800

H -0.52903300 6.16251600 0.42090500

C 0.37880800 4.19764900 0.44541900

C 1.71967600 4.66518300 0.44391700

H 1.90287500 5.73437200 0.44820600

C 2.76104100 3.76606800 0.43952500

C 4.21598000 3.95578400 0.44182500

C 4.98625500 5.11777200 0.45157900

H 4.51638600 6.09455700 0.45817300

C 6.37518300 5.00343200 0.45651700

H 6.98645800 5.89833800 0.46374400

C 6.99041500 3.74897400 0.45822200

H 8.07186900 3.68210900 0.46833800

C 6.22285800 2.58347800 0.44307100

H 6.69160100 1.60627400 0.43959200

C 4.84407200 2.69825300 0.42821500

C 3.80705800 1.57794600 0.34760900

C 2.49766200 2.36812900 0.42581100

C 1.22678400 1.87855500 0.44829600

H 1.04405900 0.80991900 0.46062400

C 0.13030100 2.77812800 0.46031900

C -1.20272400 2.30189800 0.47830800

H -1.38922900 1.23336200 0.49357900

C -2.23956200 3.19517100 0.47562900

C -3.69866000 2.95540200 0.48718700

C -4.33096900 4.28606100 0.46120700

C 3.89900000 -0.31419000 2.21828800

C 3.90035300 0.60087700 1.42577800

C 3.87085500 0.85069800 -1.03526100

C 3.83613400 -0.44626100 -1.05830400

C -3.11950200 1.16186100 -2.85106800

H -3.18107200 1.64850700 -3.82939800

C -5.21292300 -0.98471400 -3.58185100

H -4.45085100 -1.76795600 -3.57412400

C -6.12875500 1.69865600 -2.33899000

H -5.84553900 2.54905800 -1.71586500

C 5.29523700 2.91685800 -2.90513200

H 6.24177000 2.39847100 -2.73479600

C 2.21715100 2.69141400 -2.90642100

H 1.35786800 2.03840600 -2.73587700

C 3.92809700 0.40346200 -4.03108100

H 3.90834200 0.85184100 -5.02902300

C -4.80341100 1.11117000 3.54673000

H -4.02865000 1.87675900 3.45926400

C -5.89321800 -1.56075200 2.43503300

H -5.69067300 -2.42983100 1.80677600

C -2.83424600 -1.07274800 2.62125200

H -2.80304800 -1.54824000 3.60656500

H -2.80273900 1.91051000 -2.12224300

H -2.34395000 0.39294600 -2.90862400

H -6.16859700 -1.44740500 -3.32173100

H -5.29097300 -0.59913200 -4.60262800

H -6.32510700 2.07468000 -3.34778000

H -7.06160200 1.28356700 -1.94785000

H -5.99554100 -1.91154300 3.46663100

H -6.85214600 -1.13332100 2.12956800

H -5.76979400 1.59441300 3.38047200

H -4.78787700 0.73077700 4.57235600

H -2.60180200 -1.83326700 1.87365900

H -2.04616800 -0.31513100 2.59137400

H 5.30936300 3.31967000 -3.92312200

H 5.24432600 3.75551100 -2.20911200

H 4.84654600 -0.18205300 -3.94185500

H 3.08599300 -0.28864300 -3.95446100

H 2.14210100 3.52738500 -2.20972000

H 2.14257400 3.08925900 -3.92364900

Si 3.74226600 -1.81568700 3.26786500

C 3.80161500 -1.30361500 5.07744700

H 3.69545700 -2.17547100 5.73026800

H 4.75003200 -0.81550900 5.31717700

H 2.99535400 -0.60411700 5.31345800

C 2.08796300 -2.61237500 2.86252400

H 1.26075200 -1.92951200 3.07345600

H 2.03308300 -2.88912100 1.80808900

H 1.93902200 -3.51814400 3.45850800

C 5.15687000 -2.98689700 2.87178000

H 5.13752200 -3.28910600 1.82334200

H 6.12411400 -2.51712400 3.06885800

H 5.08829900 -3.88987500 3.48644700

**Compound 16b**

C 0.31784900 -2.07316600 1.12664400

C -0.90895400 -1.37517000 1.22693300

H -0.90554000 -0.29208600 1.21504600

C -2.08212300 -2.07530300 1.30602900

C -3.47729000 -1.59254300 1.34342700

C -4.32138500 -2.79522400 1.44865400

C -5.70398200 -2.92183200 1.50229300

H -6.33511700 -2.04074900 1.49706800

C -6.25831700 -4.19999700 1.55018500

H -7.33491600 -4.31717600 1.59125800

C -5.44050900 -5.33460600 1.53885000

H -5.89231300 -6.31906200 1.57219000

C -4.05333400 -5.21326600 1.48227800

H -3.42667100 -6.09772100 1.46774200

C -3.48850500 -3.94137300 1.43873700

C -2.08908000 -3.50726500 1.34776900

C -0.90777500 -4.20786500 1.28410400

H -0.89853400 -5.29229600 1.31194500

C 0.32620200 -3.51354000 1.15705600

C 1.56589200 -4.19904900 1.04397300

H 1.57279900 -5.28278100 1.09244700

C 2.72947200 -3.48853800 0.85297800

C 4.12474900 -3.91348500 0.68314900

C 4.70331200 -5.18147300 0.70733600

H 4.09450500 -6.06558800 0.85763200

C 6.08352200 -5.29464200 0.54774700

H 6.54641800 -6.27439900 0.56749300

C 6.87969100 -4.16012100 0.37354100

H 7.95185900 -4.26914200 0.26134700

C 6.30336200 -2.88937100 0.33749100

H 6.91304000 -2.00453900 0.19635300

C 4.93178700 -2.77874000 0.48092100

C 4.07515300 -1.51523200 0.41540200

C 2.69234600 -2.06934100 0.77302800

C 1.53572200 -1.37181600 0.94062100

H 1.53063600 -0.28907300 0.90595700

C -3.81801900 -0.32902500 1.20498500

C -3.98417300 0.94814100 1.04218500

C 4.49217700 -0.45341700 1.32241400

C 4.76078700 0.52718800 1.97955100

C -0.31784000 2.07321000 -1.12671400

C 0.90896300 1.37521700 -1.22700900

H 0.90554800 0.29213300 -1.21516500

C 2.08213400 2.07535200 -1.30606100

C 3.47729900 1.59258800 -1.34346300

C 4.32140200 2.79527500 -1.44856500

C 5.70400200 2.92188400 -1.50210300

H 6.33513500 2.04080000 -1.49688400

C 6.25834200 4.20005000 -1.54989400

H 7.33494400 4.31723100 -1.59088800

C 5.44053500 5.33466000 -1.53856500

H 5.89234200 6.31911700 -1.57182700

C 4.05335600 5.21331900 -1.48209400

H 3.42669300 6.09777400 -1.46755400

C 3.48852200 3.94142500 -1.43864600

C 2.08909400 3.50731500 -1.34774800

C 0.90778800 4.20791400 -1.28408700

H 0.89854700 5.29234600 -1.31189000

C -0.32619300 3.51358500 -1.15708600

C -1.56588700 4.19909000 -1.04400900

H -1.57279500 5.28282300 -1.09246400

C -2.72946900 3.48857200 -0.85305600

C -4.12475200 3.91351100 -0.68325200

C -4.70332000 5.18149600 -0.70742700

H -4.09451500 6.06561700 -0.85769400

C -6.08353500 5.29465500 -0.54786600

H -6.54643500 6.27441100 -0.56760300

C -6.87970200 4.16012700 -0.37369800

H -7.95187300 4.26914200 -0.26152300

C -6.30336800 2.88938000 -0.33765800

H -6.91304400 2.00454200 -0.19654500

C -4.93178900 2.77875800 -0.48106300

C -4.07515100 1.51525700 -0.41553100

C -2.69234000 2.06937400 -0.77313000

C -1.53571400 1.37185400 -0.94072400

H -1.53062600 0.28911100 -0.90608600

C 3.81802600 0.32906200 -1.20509400

C 3.98414400 -0.94811100 -1.04231000

C -4.49214000 0.45343600 -1.32255000

C -4.76070700 -0.52720100 -1.97965700

Si -5.00130300 -2.13249800 -2.84071200

Si 3.78460400 -2.01131100 -2.61577400

Si -3.78477000 2.01134100 2.61566900

Si 5.00144300 2.13233900 2.84085900

C -5.18755400 3.24096900 2.84873100

H -5.09023700 3.70913700 3.83381400

H -5.17490200 4.03001000 2.09573800

H -6.16275800 2.75037700 2.80535900

C -3.75373000 0.81034100 4.06272600

H -4.67441700 0.22419300 4.11980400

H -2.91897400 0.10998800 3.98424000

H -3.64576200 1.36200200 5.00155400

C -2.13686600 2.91623600 2.52501900

H -1.31967300 2.22123900 2.31626500

H -2.12626400 3.68182700 1.74862400

H -1.93088900 3.40147300 3.48443400

C 3.57075800 3.24775200 2.34812900

H 3.57290500 3.42440100 1.27091600

H 2.61014900 2.79941300 2.61428300

H 3.64400700 4.21760200 2.84984400

C 5.00790000 1.83520300 4.69816000

H 5.82386900 1.16746300 4.98704200

H 5.13294800 2.77710100 5.24104000

H 4.06983300 1.37849000 5.02443000

C 6.63374600 2.88241600 2.28740500

H 7.47166500 2.21522900 2.50617900

H 6.62056100 3.07817100 1.21279500

H 6.81367000 3.83214500 2.80061200

C 5.18730600 -3.24102000 -2.84890000

H 5.17464600 -4.03005600 -2.09590300

H 5.08990400 -3.70918700 -3.83397500

H 6.16254400 -2.75049000 -2.80558600

C 2.13665700 -2.91611900 -2.52502500

H 2.12605800 -3.68169400 -1.74861300

H 1.31950900 -2.22107600 -2.31624300

H 1.93060500 -3.40136800 -3.48441800

C 3.75356200 -0.81031300 -4.06283100

H 2.91885600 -0.10990500 -3.98429600

H 4.67428400 -0.22422400 -4.11995600

H 3.64550700 -1.36196000 -5.00165800

C -3.57047900 -3.24769400 -2.34789400

H -2.60993800 -2.79928200 -2.61417500

H -3.57253900 -3.42416600 -1.27065200

H -3.64364000 -4.21763400 -2.84944700

C -5.00792300 -1.83565500 -4.69805900

H -5.13293000 -2.77765300 -5.24077400

H -5.82398100 -1.16804000 -4.98698100

H -4.06992700 -1.37890800 -5.02448500

C -6.63352300 -2.88261400 -2.28706400

H -6.62030200 -3.07819300 -1.21242400

H -7.47149200 -2.21552000 -2.50593000

H -6.81339400 -3.83243700 -2.80011600

**Compound 17a**

Si -5.09811900 0.73522100 -2.26191500

Si 4.21467100 2.43706100 -2.36610600

Si -4.71227300 -0.70245100 2.26763100

C -3.50406900 -2.62855400 -1.24659400

C -4.13071000 -3.96538500 -1.29809700

C -5.46178200 -4.34522300 -1.16802300

H -6.23481500 -3.60397300 -1.01421400

C -5.78495900 -5.69930600 -1.24689000

H -6.81828000 -6.01015900 -1.14658400

C -4.79123300 -6.65939100 -1.45817100

H -5.06321300 -7.70676400 -1.51755500

C -3.45607300 -6.28383900 -1.59702200

H -2.69113900 -7.03361600 -1.76400300

C -3.12251200 -4.93466200 -1.51804900

C -1.82765400 -4.25386600 -1.61578400

C -0.55134300 -4.73469600 -1.78040800

H -0.36395300 -5.79811200 -1.88497800

C 0.55060800 -3.83986500 -1.79155500

C 1.88632000 -4.30449900 -1.92011500

H 2.06156500 -5.36915900 -2.03332900

C 2.93279900 -3.41570900 -1.87218500

C 4.38620200 -3.60709600 -1.92095700

C 5.15602100 -4.75790100 -2.06407600

H 4.68584100 -5.72778800 -2.17979500

C 6.54559600 -4.64541300 -2.05550500

H 7.15648400 -5.53356700 -2.16722400

C 7.16145500 -3.39917000 -1.90553700

H 8.24321100 -3.33414400 -1.90151800

C 6.40013700 -2.24165500 -1.75237100

H 6.87610000 -1.27667900 -1.62620000

C 5.01530900 -2.34856300 -1.75731600

C 3.97617300 -1.31720300 -1.58281800

C 2.68198200 -2.01640000 -1.69652200

C 1.40507300 -1.53415500 -1.59730700

H 1.21931300 -0.47572200 -1.46422500

C 0.30876100 -2.42842200 -1.63517600

C -1.01784500 -1.95871300 -1.47381200

H -1.18925700 -0.89675000 -1.33878900

C -2.05859300 -2.84789200 -1.46589900

C -4.05666000 -1.46479300 -0.97236400

C -4.49553000 -0.26187200 -0.73192100

C -4.40890200 0.31030000 0.65659200

C -4.02142700 1.53892500 0.84490000

C -5.47412800 4.36780000 1.38832100

H -6.24298100 3.62818700 1.20685400

C -5.81539100 5.69075000 1.66675500

H -6.85942100 5.97997200 1.69506600

C -4.82633000 6.64742900 1.91257600

H -5.11299400 7.66997300 2.12856400

C -3.47663000 6.29996800 1.88416000

H -2.71508600 7.04709400 2.07671200

C -3.12444500 4.98304400 1.60346900

C -1.81122700 4.33757600 1.49524800

C -0.53337400 4.82223500 1.63699900

H -0.35379100 5.85958400 1.89867300

C 0.57973300 3.96267900 1.43328500

C 1.91539400 4.41621500 1.60198800

H 2.08642400 5.43561900 1.93102400

C 2.96602400 3.56053800 1.36250100

C 4.41803100 3.72256200 1.51112000

C 5.17431800 4.79149300 1.98773200

H 4.69632900 5.71327600 2.29867700

C 6.55842000 4.65253600 2.07136600

H 7.15782400 5.47494200 2.44391400

C 7.18333000 3.46384300 1.68690200

H 8.26001600 3.37256400 1.76694400

C 6.43014100 2.39315300 1.20104400

H 6.90625900 1.46491900 0.90838500

C 5.05622700 2.53530300 1.11149200

C 4.03225300 1.50392300 0.63554500

C 2.71646800 2.23493900 0.90956900

C 1.45145300 1.74615600 0.78850000

H 1.28006200 0.71532600 0.50030700

C 0.34639500 2.59254600 1.05463800

C -0.97945200 2.11313600 0.93856200

H -1.14697000 1.07744900 0.66546900

C -2.02932100 2.96056600 1.16855100

C -3.48306400 2.71440300 1.09705900

C -4.12936000 4.01625400 1.35354600

C 4.09780900 -0.78077300 2.01372100

C 4.09879800 0.27157300 1.41542900

C 4.14189400 1.19659100 -0.89411200

C 4.11382100 -0.04867000 -1.26123700

C -3.59114200 1.39386500 -3.21980300

H -4.01541600 2.04051900 -3.99952900

C -2.67404900 2.25856100 -2.34260400

H -2.20534700 1.66284600 -1.55632100

H -3.20828900 3.07452600 -1.85103100

H -1.86824600 2.69916100 -2.93771000

C -2.79040400 0.28540400 -3.92081500

H -1.92931900 0.70842600 -4.44947600

H -3.39080800 -0.25955700 -4.65261300

H -2.40326700 -0.44568800 -3.20861600

C -6.01697300 -0.50800900 -3.37267100

H -5.25879600 -1.27996100 -3.55905800

C -6.41737900 0.09013600 -4.73225500

H -6.81051200 -0.68781300 -5.39553700

H -5.57678300 0.56463200 -5.24418900

H -7.20223500 0.84317500 -4.61783000

C -7.21775100 -1.20615800 -2.71635900

H -8.06304700 -0.52358300 -2.60168300

H -6.97268900 -1.59736400 -1.72843500

H -7.55800800 -2.04608300 -3.33133600

C -6.12423000 2.20909600 -1.63543600

H -5.46515900 2.69712800 -0.91018400

C -6.43046100 3.24795200 -2.72738800

H -7.08348600 2.83945100 -3.50348200

H -5.52348700 3.61376800 -3.21374100

H -6.94097500 4.11462600 -2.29367600

C -7.40594000 1.80223700 -0.89518700

H -7.85796000 2.66742300 -0.39709400

H -7.22060200 1.04262100 -0.13336600

H -8.15292300 1.40329000 -1.58530700

C 5.70182600 1.84180200 -3.39883000

H 5.41046800 0.82300200 -3.68632600

C 7.02318200 1.72418100 -2.62285300

H 7.78016400 1.21479000 -3.22947400

H 6.89746700 1.15448300 -1.69968900

H 7.42382700 2.70320600 -2.35376600

C 5.88718300 2.65542600 -4.69038100

H 6.18793300 3.68449900 -4.47427000

H 4.97172000 2.69716100 -5.28643200

H 6.66896000 2.21535900 -5.31909300

C 4.31861000 4.27456900 -1.84191900

H 3.71757300 4.36155200 -0.93334300

C 3.69031000 5.21864800 -2.88593700

H 3.78392800 6.25692000 -2.54930600

H 2.62952200 5.02152200 -3.04636500

H 4.19382300 5.14746500 -3.85451600

C 5.74128600 4.75618200 -1.51580000

H 6.35540900 4.79599800 -2.41952400

H 6.24889300 4.11915100 -0.79476700

H 5.71287100 5.76753600 -1.09791000

C 2.63686700 2.23211400 -3.41991600

H 2.71096200 3.04462600 -4.15372700

C 2.54993800 0.91855600 -4.21171500

H 2.49908500 0.04894700 -3.55616200

H 3.40612400 0.78065600 -4.87593400

H 1.64694500 0.90849300 -4.83237500

C 1.35326400 2.46131300 -2.60730600

H 0.47714300 2.48700900 -3.26295800

H 1.37003800 3.39281400 -2.03596400

H 1.19764200 1.65609000 -1.88830700

C -5.54283700 0.49171300 3.49699200

H -4.80777600 1.30118100 3.59454300

C -6.85194700 1.13143800 3.01158400

H -7.66753600 0.40515300 2.98449900

H -6.75198400 1.55405000 2.01183500

H -7.15686800 1.93993100 3.68456600

C -5.73282000 -0.13654000 4.88848400

H -6.07291600 0.61547500 5.60856000

H -4.81087100 -0.57328700 5.27991400

H -6.48903100 -0.92633700 4.86537100

C -5.72204200 -2.24464600 1.80176100

H -5.15791000 -2.68600600 0.97509700

C -7.13107500 -1.92770800 1.28188400

H -7.59790600 -2.82390400 0.85814000

H -7.12323000 -1.16043600 0.50485300

H -7.78170600 -1.57694400 2.08640800

C -5.77565000 -3.30533400 2.91394000

H -6.31924100 -2.94692800 3.79237600

H -4.77863400 -3.60944700 3.23986700

H -6.28935200 -4.20342600 2.55435800

C -3.04466600 -1.24804700 3.00620800

H -3.31411700 -1.93103600 3.82299200

C -2.18580100 -2.03359800 2.00530300

H -1.88320600 -1.40433400 1.16533700

H -2.70790600 -2.89684600 1.58675900

H -1.27060500 -2.39601300 2.48348400

C -2.24115500 -0.08967000 3.61786800

H -1.29612400 -0.45707800 4.03247400

H -2.78216700 0.41056300 4.42409300

H -1.99196900 0.66699700 2.87200300

Si 4.07280600 -2.41051600 2.87363100

C 5.09019700 -3.56855200 1.76227000

H 4.69240400 -3.38883800 0.75840600

C 4.93912400 -5.06977000 2.05079900

H 3.90172700 -5.39952000 1.96267900

H 5.52973900 -5.65192000 1.33501100

H 5.28646500 -5.33380200 3.05349700

C 6.56939800 -3.14968500 1.74503400

H 6.68267200 -2.09708800 1.47359300

H 7.04015700 -3.29781000 2.72200300

H 7.12680200 -3.74037100 1.01246100

C 2.23912300 -2.90850200 2.99698500

H 1.73720300 -2.00835500 3.37591000

C 1.66614800 -3.20734800 1.60338200

H 0.59082100 -3.40419000 1.65070500

H 1.81861200 -2.37301400 0.91880500

H 2.13518700 -4.08598400 1.15356500

C 1.94278800 -4.05407700 3.97831800

H 2.23438100 -3.80563800 5.00098500

H 0.87033000 -4.27889500 3.98773000

H 2.46286700 -4.97456400 3.70013000

C 4.87842400 -2.09864700 4.56920900

H 5.80517400 -1.56229200 4.32729900

C 5.26687100 -3.36620900 5.34718000

H 5.75255700 -3.10219100 6.29333000

H 4.39551200 -3.97913400 5.59112000

H 5.96316700 -3.99267700 4.78537200

C 4.01244200 -1.15809100 5.42409700

H 3.77270000 -0.23554400 4.88923500

H 3.06851300 -1.63321400 5.70862200

H 4.53066400 -0.88409900 6.34966800

**Compound 17b**

Si 4.22855100 -3.16836500 1.36739300

Si -4.78649700 -3.17103600 1.63398900

C -0.38092200 1.20897300 2.00123900

C 0.82311700 0.51040800 1.74713200

H 0.78801400 -0.41580000 1.18746500

C 2.01192300 1.03292100 2.17930400

C 3.39379300 0.56823400 1.95738400

C 4.26739700 1.54255700 2.63550800

C 5.65160100 1.57966100 2.74823800

H 6.25919200 0.79963900 2.30522800

C 6.23790400 2.64822500 3.42411000

H 7.31607600 2.69554000 3.51970500

C 5.45087700 3.66731600 3.96948900

H 5.92787800 4.49431000 4.48222100

C 4.06106000 3.63058800 3.86355500

H 3.45867100 4.42418400 4.29077100

C 3.46367800 2.56069800 3.20271500

C 2.05512200 2.25573800 2.92679200

C 0.89441800 2.92555400 3.22866000

H 0.91322600 3.84891400 3.79779600

C -0.35475900 2.42642600 2.77035300

C -1.57612400 3.09960900 3.03785800

H -1.56026300 3.99262400 3.65349100

C -2.75228100 2.62477400 2.50412000

C -4.14066800 3.08316400 2.62395200

C -4.69168800 4.11794300 3.37661000

H -4.05955500 4.76203200 3.97689500

C -6.07314000 4.30145600 3.35988900

H -6.51550500 5.10112500 3.94248300

C -6.89571300 3.45026900 2.61999900

H -7.97000900 3.59007700 2.63796900

C -6.34554800 2.41520300 1.86055800

H -6.98526000 1.74729400 1.29912200

C -4.97092800 2.25507900 1.84383700

C -4.13573200 1.22269200 1.08023700

C -2.74337200 1.47418000 1.67152200

C -1.60810500 0.75353400 1.45870800

H -1.62554000 -0.14960300 0.86215500

C 3.74709000 -0.46731000 1.22652700

C 4.03613500 -1.48264900 0.47339100

C 3.21786000 -4.47399500 0.40831500

H 3.61633800 -4.41723900 -0.60808900

C 3.44906000 -5.91467600 0.89625600

H 2.81608600 -6.60945900 0.33313700

H 3.19542300 -6.03359000 1.95447000

H 4.48331300 -6.23442000 0.75855500

C 1.70792700 -4.18372900 0.32985900

H 1.20680600 -4.46701000 1.25799000

H 1.25049200 -4.76765500 -0.47426700

H 1.48402800 -3.13414200 0.13509500

C 3.64713300 -3.04199800 3.18302900

H 3.73372500 -4.08606300 3.51962800

C 4.55665600 -2.19883000 4.09638700

H 4.55764600 -1.14782700 3.80308700

H 5.58967500 -2.54729100 4.09683300

H 4.19584100 -2.24600500 5.12967400

C 2.18170700 -2.61970600 3.38894700

H 2.01965700 -1.58327200 3.09336000

H 1.91537800 -2.69608000 4.44883300

H 1.48046200 -3.23774500 2.83061300

C 6.09115600 -3.60727800 1.31822400

H 6.25461900 -3.98546500 0.30264900

C 6.49727900 -4.72221500 2.30035300

H 6.37279000 -4.40836100 3.33964300

H 7.55381300 -4.97934900 2.16588400

H 5.91888300 -5.63653000 2.16394900

C 7.00914200 -2.38606800 1.51100300

H 6.97278100 -2.02018300 2.53966100

H 6.73350900 -1.55069700 0.86642800

H 8.05039300 -2.65074700 1.29628200

C -4.54535500 -0.15579600 1.32141900

C -4.75987700 -1.34017800 1.45356500

C -5.61390900 -3.87966200 0.07751000

H -5.09627000 -3.36728000 -0.74074200

C -7.10240600 -3.51988000 -0.03206300

H -7.69506400 -4.03813600 0.72720600

H -7.49608000 -3.81063400 -1.01129000

H -7.26936300 -2.44612600 0.08750300

C -5.39352000 -5.38871000 -0.11499400

H -4.33442700 -5.65661800 -0.08600400

H -5.78541800 -5.70771500 -1.08676000

H -5.90440500 -5.97520100 0.65340800

C -5.64752500 -3.56476100 3.28380600

H -4.93514500 -3.20855700 4.03945300

C -5.84713300 -5.07306300 3.50477100

H -6.58633700 -5.47949100 2.80865300

H -6.21167600 -5.27377200 4.51807400

H -4.92032100 -5.63817300 3.37039800

C -6.95804600 -2.79098700 3.50027400

H -6.81512600 -1.71611600 3.36804800

H -7.34241000 -2.95867800 4.51262900

H -7.73394200 -3.11172500 2.80048800

C -2.96267000 -3.71533000 1.69701100

H -2.96889400 -4.80301500 1.85014900

C -2.25066000 -3.41949100 0.36804400

H -1.20490000 -3.74057900 0.39945200

H -2.25635100 -2.34884200 0.15575900

H -2.72321500 -3.91677500 -0.48125400

C -2.20371600 -3.07062800 2.86868500

H -2.21950400 -1.97958600 2.79339300

H -1.15426800 -3.38474000 2.87131500

H -2.63027800 -3.34178400 3.83781200

Si -4.22854900 3.16853800 -1.36718200

Si 4.78659600 3.17084100 -1.63422100

C 0.38088400 -1.20891100 -2.00133200

C -0.82312900 -0.51027600 -1.74728300

H -0.78799300 0.41593900 -1.18762900

C -2.01195100 -1.03275300 -2.17945300

C -3.39381000 -0.56803200 -1.95753200

C -4.26743900 -1.54234700 -2.63563600

C -5.65164500 -1.57941700 -2.74836100

H -6.25921300 -0.79937500 -2.30535400

C -6.23797900 -2.64798000 -3.42420700

H -7.31615300 -2.69527000 -3.51979500

C -5.45097900 -3.66710100 -3.96957100

H -5.92800300 -4.49409000 -4.48229000

C -4.06116100 -3.63040200 -3.86364800

H -3.45879300 -4.42402400 -4.29084700

C -3.46375000 -2.56051600 -3.20282800

C -2.05518700 -2.25558800 -2.92691000

C -0.89450800 -2.92545900 -3.22874400

H -0.91335000 -3.84883600 -3.79785000

C 0.35468000 -2.42638100 -2.77041900

C 1.57601400 -3.09964200 -3.03786600

H 1.56011800 -3.99267500 -3.65347300

C 2.75217900 -2.62486900 -2.50409400

C 4.14053900 -3.08336800 -2.62383800

C 4.69151900 -4.11822400 -3.37642000

H 4.05936800 -4.76228200 -3.97672100

C 6.07295100 -4.30187500 -3.35958100

H 6.51528300 -5.10160900 -3.94211000

C 6.89554800 -3.45075400 -2.61964400

H 7.96983100 -3.59066800 -2.63752400

C 6.34542400 -2.41560600 -1.86028300

H 6.98515400 -1.74773300 -1.29882400

C 4.97081800 -2.25533900 -1.84368600

C 4.13567800 -1.22280400 -1.08022900

C 2.74330700 -1.47425400 -1.67152300

C 1.60807500 -0.75353400 -1.45876500

H 1.62554900 0.14962500 -0.86224500

C -3.74709300 0.46747100 -1.22661200

C -4.03621800 1.48271200 -0.47337300

C -3.21764400 4.47392500 -0.40801400

H -3.61610900 4.41712300 0.60839300

C -3.44865300 5.91468200 -0.89582400

H -2.81563400 6.60934500 -0.33260700

H -3.19495100 6.03367100 -1.95401400

H -4.48288200 6.23452300 -0.75815000

C -1.70774900 4.18342200 -0.32964500

H -1.20661700 4.46675700 -1.25775300

H -1.25020100 4.76716000 0.47455000

H -1.48399000 3.13377700 -0.13504400

C -3.64731900 3.04237000 -3.18288300

H -3.73391700 4.08648100 -3.51933800

C -4.55699100 2.19938400 -4.09625900

H -4.55798600 1.14833400 -3.80313300

H -5.58999600 2.54789000 -4.09651300

H -4.19630600 2.24672200 -5.12958400

C -2.18192300 2.62007200 -3.38898700

H -2.01976900 1.58369400 -3.09324800

H -1.91578300 2.69627400 -4.44893200

H -1.48060700 3.23824800 -2.83088700

C -6.09108700 3.60766100 -1.31784800

H -6.25442600 3.98581200 -0.30224100

C -6.49711500 4.72269900 -2.29989700

H -6.37272200 4.40885600 -3.33920500

H -7.55360600 4.97997500 -2.16535700

H -5.91857700 5.63692600 -2.16348400

C -7.00924400 2.38658100 -1.51063300

H -6.97309800 2.02083400 -2.53934600

H -6.73360100 1.55108400 -0.86622300

H -8.05043000 2.65134500 -1.29570500

C 4.54541600 0.15562700 -1.32154600

C 4.75998000 1.33999500 -1.45373800

C 5.61391600 3.87952600 -0.07772200

H 5.09631300 3.36710600 0.74052600

C 7.10243900 3.51984700 0.03185400

H 7.69506400 4.03810600 -0.72744000

H 7.49609700 3.81066300 1.01107000

H 7.26946100 2.44609700 -0.08766400

C 5.39342300 5.38856100 0.11478200

H 4.33431000 5.65638800 0.08581000

H 5.78531300 5.70759200 1.08654300

H 5.90425200 5.97508900 -0.65362800

C 5.64777000 3.56455000 -3.28397200

H 4.93535500 3.20858100 -4.03969600

C 5.84769900 5.07283500 -3.50476200

H 6.58695300 5.47903900 -2.80856600

H 6.21232900 5.27358000 -4.51802700

H 4.92099500 5.63812000 -3.37036900

C 6.95814700 2.79053500 -3.50045000

H 6.81498700 1.71567400 -3.36841100

H 7.34263700 2.95831600 -4.51274300

H 7.73404700 3.11099100 -2.80053800

C 2.96277100 3.71509600 -1.69738700

H 2.96899000 4.80277400 -1.85058300

C 2.25070000 3.41929900 -0.36844300

H 1.20493600 3.74035700 -0.39992400

H 2.25640800 2.34865600 -0.15610400

H 2.72319600 3.91662900 0.48086000

C 2.20388700 3.07031100 -2.86906100

H 2.21969100 1.97927400 -2.79369900

H 1.15443300 3.38440300 -2.87176100

H 2.63048900 3.34141200 -3.83818700

**Compound 18a**

C -3.95717800 0.46852300 -2.86585800

C -4.73222000 0.12294000 -4.07015900

C -6.10373700 0.00016400 -4.25414400

H -6.78671500 0.19686000 -3.43780400

C -6.58182800 -0.37465400 -5.50903700

H -7.64903100 -0.47544700 -5.66811600

C -5.69829700 -0.61868500 -6.56490300

H -6.08957900 -0.90900200 -7.53291000

C -4.32147200 -0.48715100 -6.38852500

H -3.64390700 -0.67396400 -7.21384000

C -3.83372300 -0.11201100 -5.13978800

C -2.46303500 0.08727800 -4.65525300

C -1.23907400 -0.12139400 -5.24426000

H -1.16344200 -0.46216200 -6.27142600

C -0.04710700 0.05249200 -4.49163600

C 1.22751100 -0.24562100 -5.04041200

H 1.29066600 -0.57346900 -6.07253400

C 2.35372600 -0.14146700 -4.26137900

C 3.76396100 -0.41846900 -4.54665800

C 4.39255200 -0.88405400 -5.69829000

H 3.82282800 -1.07997400 -6.59944600

C 5.76643900 -1.11344300 -5.67017700

H 6.26758500 -1.48225400 -6.55746000

C 6.50545200 -0.88737100 -4.50401300

H 7.57060800 -1.08710100 -4.49846400

C 5.88680100 -0.41291800 -3.34834100

H 6.45332200 -0.25029300 -2.44035700

C 4.51924400 -0.16758800 -3.37760300

C 3.61433900 0.33034600 -2.32234900

C 2.25649800 0.29643500 -2.89876100

C 1.04671700 0.62739300 -2.35102500

H 0.97209100 0.97346000 -1.32620900

C -0.13502600 0.48973300 -3.12165600

C -1.40838700 0.69846300 -2.54010700

H -1.47423900 0.96774700 -1.49258300

C -2.53882000 0.49072400 -3.28337300

C -4.33259000 0.54663600 -1.60686200

C -4.48220800 0.60960100 -0.31512800

C -4.18357600 -0.60609300 0.51513200

C -3.67205900 -0.45339300 1.70251200

C -4.95488700 -0.31732100 4.62308100

H -5.71977600 -0.63128100 3.92594900

C -5.26024700 -0.10014400 5.96547800

H -6.27694700 -0.24095900 6.31327600

C -4.26768500 0.29428000 6.86812900

H -4.52496200 0.45882800 7.90786800

C -2.95057600 0.46890700 6.44662500

H -2.18454100 0.76590700 7.15384000

C -2.63678700 0.25266600 5.10741100

C -1.36360100 0.33936900 4.38643400

C -0.10011900 0.67146100 4.80677800

H 0.09310600 0.94100400 5.83954400

C 0.98210500 0.65672300 3.88793200

C 2.30107400 0.97999600 4.29636800

H 2.47640400 1.25897700 5.32965600

C 3.33016800 0.94319200 3.38619900

C 4.75476900 1.26071700 3.51826500

C 5.48155700 1.74291600 4.60447900

H 5.00059300 1.91469900 5.56043900

C 6.83899900 2.01384100 4.43899600

H 7.41508100 2.39708000 5.27305200

C 7.46678800 1.79253800 3.21138300

H 8.52435500 2.00186900 3.10289500

C 6.74264600 1.30185200 2.12233600

H 7.22934800 1.12105000 1.17220900

C 5.38992900 1.05696100 2.28036500

C 4.38964300 0.55129700 1.23739500

C 3.07814700 0.56589100 2.03903700

C 1.82414700 0.24912800 1.60545800

H 1.65023300 -0.05081400 0.57850800

C 0.73464100 0.30081500 2.51431700

C -0.58665800 -0.00304200 2.09894600

H -0.77083700 -0.24555400 1.05907900

C -1.60729000 0.00193100 3.01366300

C -3.05417000 -0.26267100 2.84981500

C -3.64699900 -0.12975900 4.19458900

C 4.80600200 -2.02105400 0.66832700

C 4.67642200 -0.84097700 0.90226000

C 4.27530700 1.41828400 -0.04256500

C 3.95024100 0.82998800 -1.15279200

Si 4.54112000 -3.83226600 0.50429900

Si -4.30387100 -2.37964600 -0.21902200

Si -4.84123500 2.31334100 0.48841100

Si 4.51875500 3.32851100 -0.16308200

C 2.77765600 -4.03745900 1.18795800

C 2.14073600 -5.43194800 1.02780600

C 2.61078100 -3.52495100 2.63278600

H 2.20576500 -3.34731300 0.55171900

C 0.67121400 -5.43787800 1.47305700

H 2.69742200 -6.16342700 1.62307300

H 2.20568900 -5.76510000 -0.01203000

C 1.13467400 -3.50880200 3.04914600

H 3.16927700 -4.17387400 3.31974900

H 3.03580700 -2.52307900 2.72873700

C 0.49837600 -4.89484000 2.89617100

H 0.26110500 -6.45092500 1.40156500

H 0.09289100 -4.81677100 0.78274800

H 1.03545600 -3.15474500 4.08031900

H 0.59619000 -2.78767500 2.42297000

H -0.56412800 -4.85714400 3.15723200

H 0.97090200 -5.58658700 3.60580300

C 5.91348400 -4.72482000 1.46883100

C 6.40382200 -3.93644000 2.70182100

C 5.55102800 -6.16803800 1.87560100

H 6.75801500 -4.78336300 0.76403600

C 7.57317700 -4.64303000 3.39852000

H 5.57642100 -3.82464200 3.41322300

H 6.69270900 -2.92340500 2.41180300

C 6.72559900 -6.87664900 2.56202600

H 4.70405100 -6.13995700 2.57092700

H 5.21982900 -6.74815900 1.00952300

C 7.21831500 -6.08511800 3.77874600

H 7.87657100 -4.08017400 4.28746800

H 8.43762200 -4.65192500 2.72204900

H 6.43272000 -7.88870300 2.86051600

H 7.54768000 -6.98539800 1.84305300

H 8.08026700 -6.58328800 4.23390700

H 6.42684200 -6.07067100 4.53927900

C 4.63712300 -4.20739500 -1.35203400

C 4.76541900 -5.69694300 -1.72566200

C 3.47074600 -3.54963500 -2.11650300

H 5.56241200 -3.70861500 -1.67878100

C 4.87869600 -5.89223900 -3.24401300

H 3.88894700 -6.24766700 -1.36333000

H 5.63545700 -6.13998700 -1.23125900

C 3.59755900 -3.74766400 -3.62950100

H 2.52653800 -3.99257000 -1.78147200

H 3.41705500 -2.48343400 -1.88092800

C 3.70992300 -5.23310700 -3.98589400

H 4.93002000 -6.95972100 -3.48321900

H 5.81916500 -5.44484400 -3.59010300

H 2.74264200 -3.29323600 -4.13975200

H 4.48772700 -3.21933700 -3.98572900

H 3.82952100 -5.35806500 -5.06697900

H 2.77645300 -5.74230700 -3.71212600

C -4.25109500 -3.52604100 1.29054400

C -5.46809700 -3.37640900 2.22557600

C -3.99294700 -5.01915400 1.00337600

H -3.37719800 -3.15878900 1.84940900

C -5.26154600 -4.14544100 3.53544000

H -6.36342400 -3.75967200 1.72080800

H -5.65999100 -2.32225700 2.43879000

C -3.77745100 -5.79832000 2.30793900

H -4.84743900 -5.44748400 0.46898500

H -3.12622800 -5.14689600 0.35041200

C -4.95986000 -5.62545300 3.26939600

H -6.14416000 -4.04818400 4.17630700

H -4.42569500 -3.69179600 4.08182000

H -3.61756700 -6.85986500 2.09253300

H -2.86182500 -5.43414900 2.79003900

H -4.76281600 -6.14834000 4.21050500

H -5.84842400 -6.09680900 2.82947100

C -2.75034800 -2.55966400 -1.29641000

C -1.48072800 -2.81498100 -0.46513300

C -2.84667800 -3.56725400 -2.45842900

H -2.63125800 -1.57518900 -1.75809400

C -0.21978600 -2.77574300 -1.33382100

H -1.55039300 -3.79526400 0.01657000

H -1.40049400 -2.08853900 0.34766500

C -1.58237800 -3.52890200 -3.32799800

H -2.98257900 -4.58239700 -2.06417700

H -3.72107800 -3.34938100 -3.07812400

C -0.31530900 -3.76558500 -2.49909300

H 0.66235200 -2.99023100 -0.72613100

H -0.08470200 -1.76769700 -1.73322100

H -1.65553000 -4.27203300 -4.12913100

H -1.51704100 -2.54826700 -3.81148300

H 0.57044500 -3.68480400 -3.13468400

H -0.32667900 -4.78959000 -2.10219700

C -5.87976400 -2.51393200 -1.27772900

C -6.27106600 -3.98386700 -1.54726200

C -7.09346900 -1.73856100 -0.72911100

H -5.61496600 -2.06464200 -2.24476000

C -7.47960500 -4.08618500 -2.48553500

H -6.52543600 -4.46827900 -0.59760200

H -5.42888300 -4.54175700 -1.96342800

C -8.32386300 -1.86403400 -1.63756700

H -7.34995300 -2.11366100 0.26866800

H -6.83911200 -0.68590800 -0.60690300

C -8.68269200 -3.32658900 -1.91732100

H -7.73710600 -5.13723600 -2.65243200

H -7.21184800 -3.66539000 -3.46271100

H -9.17372700 -1.34065000 -1.18692700

H -8.11601400 -1.36541200 -2.59113000

H -9.53260400 -3.38517600 -2.60460200

H -9.00069100 -3.80674100 -0.98275400

C -6.04886900 2.07971900 1.94306800

C -6.70977800 3.41085800 2.36470900

C -7.12943600 1.00763300 1.69380700

H -5.43535900 1.73860700 2.78730500

C -7.63222400 3.23242900 3.57616400

H -7.30752400 3.79617700 1.53131500

H -5.95356500 4.16933600 2.58051400

C -8.10006200 0.85707200 2.87380400

H -7.70495200 1.26637400 0.79772400

H -6.65757900 0.04660600 1.48708100

C -8.72100000 2.19554200 3.28365300

H -8.08281800 4.19247000 3.84835000

H -7.03763700 2.90389200 4.43762500

H -8.88154900 0.13375300 2.61837200

H -7.56145100 0.44692400 3.73476200

H -9.36964900 2.06218800 4.15516200

H -9.35886200 2.56555600 2.47046700

C -3.13973400 2.92587500 1.08390900

C -2.28772000 3.51476000 -0.05851900

C -3.17562000 3.87676000 2.29654400

H -2.62811900 2.01786900 1.42272000

C -0.87819000 3.88352000 0.41977100

H -2.76553600 4.41829000 -0.45225900

H -2.23373600 2.81485200 -0.89572000

C -1.76108700 4.25723900 2.75324800

H -3.72233300 4.79242800 2.03798200

H -3.71698800 3.40978400 3.12424700

C -0.95237100 4.85808800 1.59956800

H -0.30171500 4.31878300 -0.40249900

H -0.34720100 2.97645300 0.73450600

H -1.81333400 4.96051200 3.59095600

H -1.25145600 3.35913400 3.12393500

H 0.04772200 5.13251200 1.93743800

H -1.43511000 5.78602200 1.26564500

C -5.51633500 3.37803900 -0.93054400

C -6.85135800 2.86708100 -1.51167700

C -5.61220000 4.89440000 -0.66440100

H -4.76008800 3.24436600 -1.71858900

C -7.22242000 3.61600500 -2.79682400

H -7.64800600 3.00906900 -0.77130800

H -6.79852600 1.79346900 -1.70834600

C -5.97379500 5.65502600 -1.94741400

H -6.37886100 5.09067000 0.09198900

H -4.67423300 5.28028300 -0.25734200

C -7.27720800 5.13059900 -2.56285500

H -8.18356900 3.25768700 -3.18037400

H -6.47350800 3.38973500 -3.56553200

H -6.05905900 6.72681800 -1.74022700

H -5.15826900 5.53729500 -2.67197500

H -7.49103000 5.65273500 -3.50067300

H -8.10846100 5.35641000 -1.88222600

C 4.09646700 4.07961400 1.53322800

C 4.62432500 5.51269800 1.75886200

C 2.60625600 3.98742400 1.91736700

H 4.64890700 3.44926600 2.23748500

C 4.36479600 5.98620500 3.19607300

H 4.14600100 6.20826700 1.06015200

H 5.69778300 5.55516900 1.55890400

C 2.37213000 4.44753200 3.36099300

H 2.01733800 4.61868800 1.24530200

H 2.23139600 2.97239500 1.78600600

C 2.88402500 5.87477000 3.57462300

H 4.71419700 7.01675800 3.31868400

H 4.95662900 5.36697100 3.88191100

H 1.31080700 4.37353500 3.61546700

H 2.90147200 3.76827200 4.03777800

H 2.73360300 6.18475600 4.61372300

H 2.29863300 6.56559200 2.95371600

C 3.33953300 3.90592300 -1.55037300

C 2.89218000 5.38083200 -1.45169500

C 3.82017500 3.63237300 -2.99458700

H 2.44064500 3.29240500 -1.39083700

C 1.78708400 5.69586800 -2.47018400

H 3.75162800 6.03697400 -1.63567100

H 2.53299000 5.61606500 -0.44859900

C 2.70817000 3.91287800 -4.01240700

H 4.67201200 4.28392500 -3.21812800

H 4.17457200 2.61088000 -3.11166400

C 2.21276500 5.35874900 -3.90338500

H 1.49836200 6.74969500 -2.39705700

H 0.89712400 5.10774800 -2.21363200

H 3.06874800 3.70962900 -5.02588800

H 1.87767300 3.22076900 -3.83077900

H 1.38280200 5.53335500 -4.59531900

H 3.02138900 6.03712300 -4.20532900

C 6.36248700 3.64071200 -0.53747400

C 7.01168900 2.55976600 -1.42406800

C 6.65091700 5.04015700 -1.12496300

H 6.85413000 3.59397700 0.44543800

C 8.51448500 2.79771800 -1.61029000

H 6.52418800 2.54702200 -2.40553100

H 6.84302100 1.56916700 -0.99651800

C 8.15665300 5.28136100 -1.29427500

H 6.17062200 5.12812900 -2.10449400

H 6.21877600 5.82500800 -0.50062000

C 8.79983700 4.19719200 -2.16569400

H 8.93624200 2.03340200 -2.27145800

H 9.01596500 2.68719400 -0.63970000

H 8.33198700 6.27093800 -1.72887500

H 8.63298800 5.28233100 -0.30543200

H 9.87867800 4.36267900 -2.24752500

H 8.39302200 4.26779900 -3.18253500

**Compound 18b**

C 0.32599400 0.63820900 -2.22312400

C -0.80929700 -0.02165000 -1.68859800

H -0.68226400 -0.70021600 -0.85361500

C -2.05021000 0.22720200 -2.21478800

C -3.38336200 -0.25287500 -1.80344400

C -4.34275900 0.33064400 -2.76116300

C -5.72264900 0.19269500 -2.84646500

H -6.25916400 -0.41579300 -2.12943700

C -6.40012500 0.86662000 -3.86152700

H -7.47651400 0.77193600 -3.94141500

C -5.70797400 1.66930900 -4.77358100

H -6.25479200 2.18772100 -5.55230800

C -4.32347800 1.80894500 -4.69176700

H -3.79383100 2.43224600 -5.40302000

C -3.63656000 1.13694900 -3.68441900

C -2.21110200 1.09822700 -3.34440100

C -1.12445600 1.73362600 -3.89264300

H -1.23612500 2.40399600 -4.73807900

C 0.16856700 1.53675800 -3.33830200

C 1.30426600 2.22122300 -3.84095200

H 1.18242600 2.88986200 -4.68592500

C 2.52671500 2.06284300 -3.23093700

C 3.82736400 2.68481900 -3.49058000

C 4.22103000 3.58942400 -4.47419900

H 3.51427500 3.94276900 -5.21596100

C 5.54215600 4.03362400 -4.49016100

H 5.86154000 4.74078500 -5.24670500

C 6.46351800 3.56375900 -3.55212700

H 7.49164300 3.90368900 -3.59075600

C 6.07198500 2.65420800 -2.56699000

H 6.78914000 2.27653600 -1.84980100

C 4.75124400 2.24307400 -2.52623000

C 4.07775700 1.30316300 -1.52315500

C 2.66261000 1.19865600 -2.11064500

C 1.60823600 0.47560400 -1.63686800

H 1.72513100 -0.19170800 -0.79174700

C -3.66422000 -1.02371300 -0.77432500

C -3.93448400 -1.80931200 0.22094800

C 4.75657400 0.01557500 -1.44450600

C 5.31203300 -1.04409200 -1.26280200

C -0.41985100 -0.44896100 2.38868500

C 0.73354800 0.15949900 1.83133400

H 0.62096900 0.84081400 0.99664100

C 1.97281700 -0.16508800 2.31802600

C 3.31981300 0.22314900 1.85697300

C 4.27225200 -0.50021300 2.72158300

C 5.66103900 -0.51491500 2.70713700

H 6.20511300 0.08223500 1.98673600

C 6.33115400 -1.32416000 3.62278600

H 7.41385900 -1.35506400 3.61948000

C 5.62040100 -2.10592000 4.53931400

H 6.16061100 -2.73489700 5.23703400

C 4.22668700 -2.08353300 4.56543600

H 3.68277900 -2.69178100 5.27911400

C 3.54880800 -1.27331900 3.65807300

C 2.11614400 -1.06918900 3.42393700

C 1.00987400 -1.61069500 4.02910000

H 1.10772200 -2.29254600 4.86699900

C -0.28554800 -1.33052500 3.52014900

C -1.44174000 -1.95354100 4.05510100

H -1.33768600 -2.59419600 4.92360900

C -2.65477700 -1.80003100 3.42576700

C -3.96468000 -2.40696100 3.67825700

C -4.39193200 -3.25327600 4.69904800

H -3.71211100 -3.55768500 5.48634300

C -5.71203600 -3.70092600 4.69363100

H -6.05795100 -4.36143300 5.48012400

C -6.59864600 -3.29439800 3.69461200

H -7.62644800 -3.63683800 3.71530000

C -6.17313400 -2.44479600 2.67070800

H -6.86417300 -2.11704100 1.90494400

C -4.85408800 -2.02631000 2.65647400

C -4.14614800 -1.14029900 1.62885700

C -2.75838900 -0.98817100 2.26451400

C -1.69378500 -0.28292400 1.78701400

H -1.79310000 0.35039600 0.91367200

C 3.62607200 1.04131600 0.87255300

C 3.94748400 1.87691600 -0.06520300

C -4.81996100 0.13622700 1.43559500

C -5.35242700 1.18409500 1.14916600

Si -6.03391600 2.75464700 0.48122800

Si 4.17398900 3.69667200 0.51036200

Si -3.98294400 -3.68211900 -0.21156100

Si 6.07627800 -2.64437800 -0.77879100

C 7.69961700 -2.15715900 0.07916200

C 8.33572900 -3.23729900 0.97346400

C 8.72682600 -1.57520800 -0.91147200

H 7.39708800 -1.33649500 0.74238200

C 9.59568600 -2.71622800 1.67840500

H 8.60285200 -4.11414100 0.37181300

H 7.61249700 -3.57921800 1.71933400

C 9.97612600 -1.05342500 -0.19123900

H 9.02914600 -2.34975400 -1.62712300

H 8.26731900 -0.77309400 -1.49768700

C 10.60921900 -2.14335600 0.68119000

H 10.05407300 -3.51425400 2.27164500

H 9.30766600 -1.92632100 2.38393000

H 10.70400900 -0.67764000 -0.91779800

H 9.69424500 -0.20166100 0.44130600

H 11.48205100 -1.74937900 1.21131600

H 10.97388200 -2.95219100 0.03485300

C 6.37549600 -3.58780300 -2.39834800

C 7.27709600 -4.83382300 -2.29254000

C 5.06052400 -3.92588200 -3.13035400

H 6.90937600 -2.85617300 -3.02374800

C 7.53056500 -5.46433900 -3.66887900

H 6.80695700 -5.57931300 -1.64216900

H 8.23191900 -4.57575400 -1.82598100

C 5.31956600 -4.55770300 -4.50337500

H 4.47668100 -4.62876000 -2.52387500

H 4.44890900 -3.02535200 -3.23880500

C 6.21735300 -5.79478000 -4.38740300

H 8.14286300 -6.36601300 -3.56291000

H 8.10854900 -4.76094100 -4.28181700

H 4.37077000 -4.82024500 -4.98267200

H 5.80573000 -3.81733300 -5.15111000

H 6.42136200 -6.21274000 -5.37827900

H 5.68562900 -6.57192700 -3.82295900

C 4.87139000 -3.47607100 0.43556100

C 3.38895800 -3.19020500 0.10996700

C 5.10771100 -4.99293600 0.58920600

H 5.08346700 -3.00858400 1.40714700

C 2.43871200 -3.83940000 1.12213800

H 3.15236000 -3.56603100 -0.89256400

H 3.22066600 -2.11209700 0.08154900

C 4.16096900 -5.61325200 1.62373400

H 4.93981900 -5.48704000 -0.37478200

H 6.14650700 -5.19765200 0.86425500

C 2.69710300 -5.34268500 1.26158300

H 1.40115000 -3.65202000 0.82893400

H 2.57661900 -3.36195400 2.09658600

H 4.34268800 -6.69040100 1.70200900

H 4.37502100 -5.18084000 2.60931200

H 2.03309700 -5.77822200 2.01415300

H 2.46309800 -5.84271500 0.31246300

C -5.82073000 -4.18211100 -0.26959500

C -6.74498300 -3.07192500 -0.81257300

C -6.09769600 -5.50092000 -1.02380400

H -6.09384600 -4.34846300 0.78159700

C -8.22317500 -3.46430300 -0.71454800

H -6.49684900 -2.87577100 -1.86170400

H -6.57096800 -2.13219200 -0.28343500

C -7.57539100 -5.90338500 -0.92210200

H -5.83709000 -5.37937300 -2.08040300

H -5.46864200 -6.30923300 -0.64437300

C -8.49755600 -4.79037100 -1.43284100

H -8.85223600 -2.66910300 -1.12781100

H -8.49668200 -3.56404200 0.34407200

H -7.75285000 -6.82797200 -1.48111400

H -7.81457600 -6.11852600 0.12718900

H -9.54634100 -5.07957900 -1.31306500

H -8.33080900 -4.65378300 -2.50903100

C -3.09894400 -3.81453700 -1.89577000

C -2.47787600 -5.19077100 -2.21486600

C -3.93705000 -3.35103300 -3.10789200

H -2.26087400 -3.10874900 -1.80053900

C -1.61831100 -5.12690600 -3.48591900

H -3.27456300 -5.93259600 -2.35044700

H -1.86343700 -5.54385600 -1.38476300

C -3.07412600 -3.24998400 -4.37009700

H -4.74025200 -4.07437900 -3.28726700

H -4.41908000 -2.39396500 -2.91853200

C -2.40598300 -4.59251000 -4.68780000

H -1.20790800 -6.11662600 -3.71225400

H -0.76219200 -4.46750900 -3.29507600

H -3.68318000 -2.91818500 -5.21709600

H -2.30733400 -2.48125300 -4.21387200

H -1.74910600 -4.49785100 -5.55820000

H -3.18217800 -5.32013100 -4.95859900

C -3.07970100 -4.62703600 1.16609600

C -3.29413800 -6.15641700 1.15895400

C -1.57938400 -4.27802100 1.26510900

H -3.55008200 -4.26533600 2.08636600

C -2.60727900 -6.83137500 2.35472800

H -2.90469400 -6.59231300 0.23284900

H -4.36307500 -6.38369800 1.18637400

C -0.93274100 -4.95382700 2.47886900

H -1.05723700 -4.60657500 0.35876200

H -1.44143200 -3.19643200 1.32425300

C -1.11959800 -6.47226000 2.43249200

H -2.73773200 -7.91698000 2.29532800

H -3.10195000 -6.50289100 3.27759800

H 0.12538000 -4.69073800 2.53786100

H -1.40313200 -4.56493000 3.38807700

H -0.66128800 -6.94153200 3.30911300

H -0.59987200 -6.87554300 1.55382500

C 6.04574800 4.04821400 0.55007200

C 6.44113600 5.27432100 1.40124000

C 6.89657300 2.82869600 0.96210700

H 6.30077300 4.28790400 -0.49119300

C 7.94219500 5.57332100 1.28687800

H 6.19841700 5.08528600 2.45196700

H 5.86534100 6.15532200 1.10750800

C 8.39736600 3.12058000 0.85290800

H 6.66025900 2.55549100 1.99674400

H 6.63900100 1.95638000 0.35581200

C 8.78832100 4.35479600 1.67359700

H 8.20422600 6.43068700 1.91541500

H 8.17119200 5.85923100 0.25230200

H 8.97633500 2.24903600 1.17633700

H 8.65280900 3.29258100 -0.20095100

H 9.85303100 4.57498200 1.54793500

H 8.63634800 4.13758800 2.73865600

C 3.32533500 4.79809700 -0.77867300

C 1.80968700 4.53344100 -0.88972800

C 3.61586400 6.30998100 -0.67611500

H 3.77290600 4.47529600 -1.72440500

C 1.20075500 5.30113500 -2.06674200

H 1.30843800 4.84432900 0.03501800

H 1.61208700 3.46512300 -1.00180300

C 2.97696600 7.08092700 -1.84049800

H 3.23468900 6.70970600 0.26956600

H 4.69496300 6.48627600 -0.67802000

C 1.47325000 6.80337700 -1.94906300

H 0.12895400 5.10515200 -2.12833000

H 1.64301900 4.92690800 -2.99665300

H 3.16029100 8.15423000 -1.72416200

H 3.46484900 6.77512200 -2.77457100

H 1.05008500 7.33723600 -2.80609100

H 0.96661000 7.19328900 -1.05666200

C 3.34158400 3.76763100 2.22311800

C 4.16946300 3.14906700 3.37130900

C 2.83799300 5.15964100 2.65805200

H 2.44860400 3.13776100 2.10027000

C 3.33414100 3.02353500 4.64975900

H 5.03403300 3.78965400 3.57761800

H 4.56591200 2.17319500 3.09696600

C 2.01144700 5.06890000 3.94926100

H 3.69233600 5.82887900 2.81828400

H 2.22961900 5.61498900 1.87415700

C 2.78668200 4.38805500 5.08339700

H 3.93588200 2.58270600 5.45103400

H 2.50317100 2.33209900 4.46364600

H 1.68711100 6.06790500 4.25905600

H 1.10097900 4.49304800 3.74062300

H 2.14874900 4.28262200 5.96655600

H 3.62670600 5.03039700 5.37844500

C -4.67313900 3.39837800 -0.67107100

C -5.11345700 4.50393600 -1.64994300

C -3.39755700 3.80014000 0.09411800

H -4.42190200 2.51939600 -1.27805100

C -3.97040500 4.91229300 -2.58818700

H -5.45017300 5.38753100 -1.09453400

H -5.96674400 4.16167700 -2.24303800

C -2.26508400 4.20158900 -0.85927800

H -3.61738300 4.64579700 0.75878200

H -3.07394100 2.97527200 0.73642600

C -2.71606500 5.31828700 -1.80800600

H -4.29396100 5.73017500 -3.24104500

H -3.72953800 4.06377300 -3.23687000

H -1.38641300 4.51827500 -0.28807300

H -1.96075500 3.32727900 -1.44654700

H -1.91243500 5.58067100 -2.50239400

H -2.92917000 6.22248300 -1.22263500

C -6.34172700 3.86506700 1.98691100

C -6.64512900 5.34441300 1.67891100

C -7.40766000 3.26972700 2.93019000

H -5.38361200 3.83726300 2.52775200

C -6.80406100 6.16789500 2.96431600

H -7.56728800 5.42129600 1.09232400

H -5.85003600 5.77444500 1.06360000

C -7.56436900 4.10020600 4.20986900

H -8.37411600 3.23418100 2.41211000

H -7.15026300 2.23632000 3.18094100

C -7.86917600 5.56792200 3.88914000

H -7.05498600 7.20502000 2.71835600

H -5.84240300 6.19288600 3.49245400

H -8.35333500 3.67703500 4.84036800

H -6.63372300 4.04258700 4.78821700

H -7.94422400 6.15223300 4.81168100

H -8.84858700 5.63181400 3.39711200

C -7.57630700 2.30573400 -0.53790900

C -8.30034900 1.03391500 -0.05177800

C -8.58260200 3.45858500 -0.72411100

H -7.16523100 2.07867200 -1.53011800

C -9.44497500 0.63831600 -0.99234400

H -8.70316300 1.20168100 0.95476100

H -7.58784800 0.21072400 0.04058500

C -9.71945400 3.06117000 -1.67529500

H -9.01249300 3.73119400 0.24679500

H -8.08035500 4.35391200 -1.10181000

C -10.43325000 1.79247300 -1.19478200

H -9.96497700 -0.24383800 -0.60414400

H -9.02394700 0.35027000 -1.96427500

H -10.43503000 3.88404600 -1.77420800

H -9.30113600 2.88511100 -2.67458400

H -11.21693100 1.50467000 -1.90283400

H -10.93461800 2.00430800 -0.24159100
